# Discovery and Rational Mutagenesis of Methionine Sulfoxide Reductase Biocatalysts To Expand the Substrate Scope of the Kinetic Resolution of Chiral Sulfoxides

**DOI:** 10.1021/acscatal.3c00372

**Published:** 2023-03-23

**Authors:** Silvia Anselmi, Alexandra T. P. Carvalho, Angela Serrano-Sanchez, Jose L. Ortega-Roldan, Jill Caswell, Iman Omar, Gustavo Perez-Ortiz, Sarah M. Barry, Thomas S. Moody, Daniele Castagnolo

**Affiliations:** † Department of Chemistry, University College London, 20 Gordon Street, WC1H 0AJ London, U. K.; ‡ Department of Biocatalysis and Isotope Chemistry, Almac, 20 Seagoe Industrial Estate, Craigavon BT63 5QD, U. K.; § Arran Chemical Company Limited, Unit 1 Monksland Industrial Estate, Athlone, Co., Roscommon N37 DN24, Ireland; ∥ School of Biosciences, University of Kent, Canterbury CT2 7NJ, U. K.; ⊥ Faculty of Natural, Mathematical and Engineering Sciences, Department of Chemistry, King’s College London, 7 Trinity Street, SE1 1DB London, U. K.

**Keywords:** sulfoxide, methionine reductase, MsrA, biocatalysis, mutagenesis

## Abstract

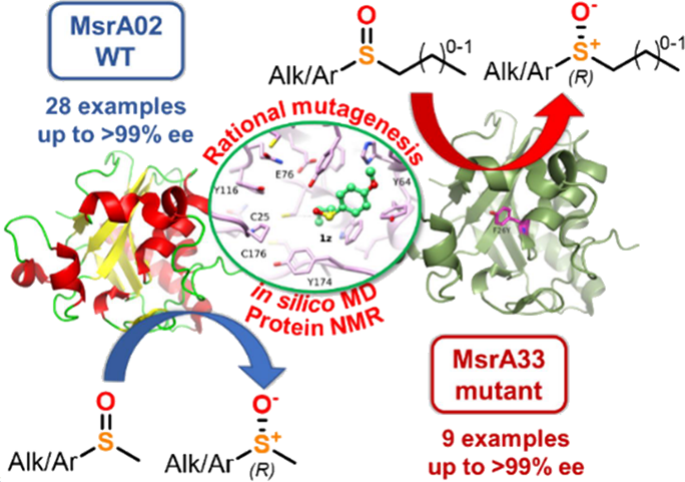

Methionine sulfoxide reductase A (MsrA) enzymes have recently found applications as nonoxidative biocatalysts in the enantioselective kinetic resolution of racemic sulfoxides. This work describes the identification of selective and robust MsrA biocatalysts able to catalyze the enantioselective reduction of a variety of aromatic and aliphatic chiral sulfoxides at 8–64 mM concentration with high yields and excellent ees (up to 99%). Moreover, with the aim to expand the substrate scope of MsrA biocatalysts, a library of mutant enzymes has been designed via rational mutagenesis utilizing *in silico* docking, molecular dynamics, and structural nuclear magnetic resonance (NMR) studies. The mutant enzyme MsrA33 was found to catalyze the kinetic resolution of bulky sulfoxide substrates bearing non-methyl substituents on the sulfur atom with ees up to 99%, overcoming a significant limitation of the currently available MsrA biocatalysts.

## Introduction

**Figure 1:**
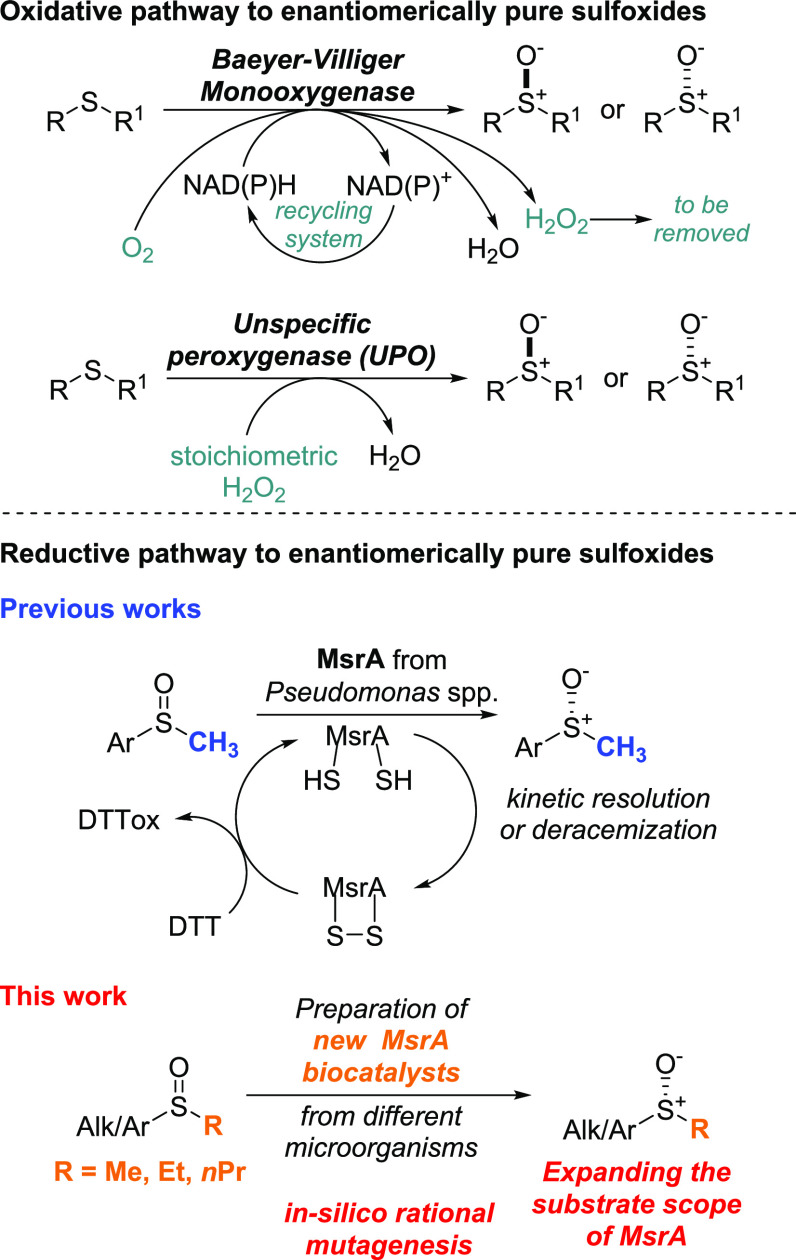
Biocatalytic approaches for the synthesis of enantiomerically pure sulfoxides.

Chiral sulfoxides are a ubiquitous class of organic compounds which find broad applications in organic and medicinal chemistry.^1^ Indeed, chiral sulfoxides can be used in organic chemistry as chiral ligands and auxiliaries for asymmetric reactions.^2^ They are also important as pharmaceutical ingredients, for example, in the blockbuster antacid esomeprazole,^3^ namely, the (*S*)-enantiomer of omeprazole, or the sleep disorder drug armodafinil.^4^ The chirality of sulfoxides has a key impact on their chemical and pharmacological properties, which has driven interest in the scientific community toward developing asymmetric strategies^5^ to access these molecules in enantiomerically pure form. The two most common methods to synthesize enantiomerically pure chiral sulfoxides involve (1) the formation of a new C–S bond by nucleophilic substitution of nonracemic sulfinyl substrates using Grignard reagents^2b,6^ and (2) the formation of the S–O bond through asymmetric oxidation of prochiral sulfides with chiral oxidants or auxiliaries.^2,7^ While catalytic C–S bond formation reactions are rare, several efficient oxidation systems have been developed for the preparation of enantiomerically pure sulfoxides. In the last decades, enzymes such as monooxygenases^1,8^ and peroxygenases^9^ have shown their potential as biocatalysts in the stereoselective oxidation of sulfides into sulfoxides, offering advantages over classic chemical oxidation methods due to the mild reaction conditions and sustainability of the processes used. However, although biocatalytic oxidative methodologies are highly efficient and stereoselective, their industrial applicability may be limited by some factors. Monooxygenases require expensive NAD(*P*)H to activate the FAD or FMN prosthetic groups and nonatom-economical systems to recycle NAD(*P*)H itself, while peroxygenases require the use of stoichiometric amounts of H_2_O_2_. Monooxygenases can also generate peroxide side products which, if not removed from the reaction mixtures, may cause overoxidation of the sulfoxide products and degradation of the enzyme (Figure 1). Recently, methionine sulfoxide reductase (Msr) enzymes have emerged as alternative nonoxidative biocatalysts able to catalyze the stereoselective reduction of racemic sulfoxides.^10^ Msrs are a large class of reductive enzymes found in many organisms,^11^ which selectively reduce the methionine sulfoxide (MetSO) residues found in proteins as a consequence of oxidative stress in cells, back to methionine (Met). Three subclasses of Msr enzymes have been identified to date, namely, MsrA and MsrB, which reduce, respectively, (*S*)-MetSO and (*R*)-MetSO residues in proteins, and the free (*R*)-Msr (frMsr), which reduces free (*R*)-MetSO amino acid.^12,13^ To date, out of the three subclasses, only a few MsrA enzymes from *Pseudomonas* sp. (*pm*MsrA, *pa*Msr, and *pm*Msr),^14−16^*E. coli*,^17a^ and a mammalian MsrA,^18^ as well as a MsrB from *Acidovorax* sp.,^17^ have shown potential as biocatalysts in the kinetic resolution (KR) of racemic aryl methyl sulfoxides.

The number of Msr biocatalysts currently available in the biocatalysis toolbox is still limited and additional investigations are required to expand their synthetic scope and fully disclose their industrial potential. Compared to oxidative biocatalysts, Msr enzymes have the advantage of not requiring any additional expensive cofactors and being regenerated with the cheap sacrificial co-substrate dithiothreitol (DTT).^18^ On the other hand, a current major limitation of available MsrA biocatalysts is their limited substrate scope as they are only able to reduce sulfoxides bearing a methyl, and in a few cases an ethyl,^16a^ substituent on the sulfur atom, while they are generally inactive on bulkier substrates. To date, only one example of a mutant MsrA enzyme with expanded substrate scope, identified via a high-throughput assay for directed evolution, has been reported.^16e^

Following up on our studies on the synthesis of chiral sulfur compounds via biocatalysis,^19^ herein, we report the preparation, screening, and development of novel MsrA biocatalysts from different microorganisms. Moreover, through a combination of in silico docking and molecular dynamics, protein NMR, and rational mutagenesis studies, a library of MsrA mutant enzymes has been rationally designed and prepared. This process resulted in a new mutant enzyme able to reduce stereoselectively sulfoxide substrates bearing non-methyl S-substituents, in turn allowing the expansion of the substrate scope of this class of biocatalysts. To the best of our knowledge, this is the first example of a rational mutagenesis study of Msr enzymes resulting in the successful development of a mutant biocatalyst with enhanced substrate scope.

## Results and Discussion

**Figure 2:**
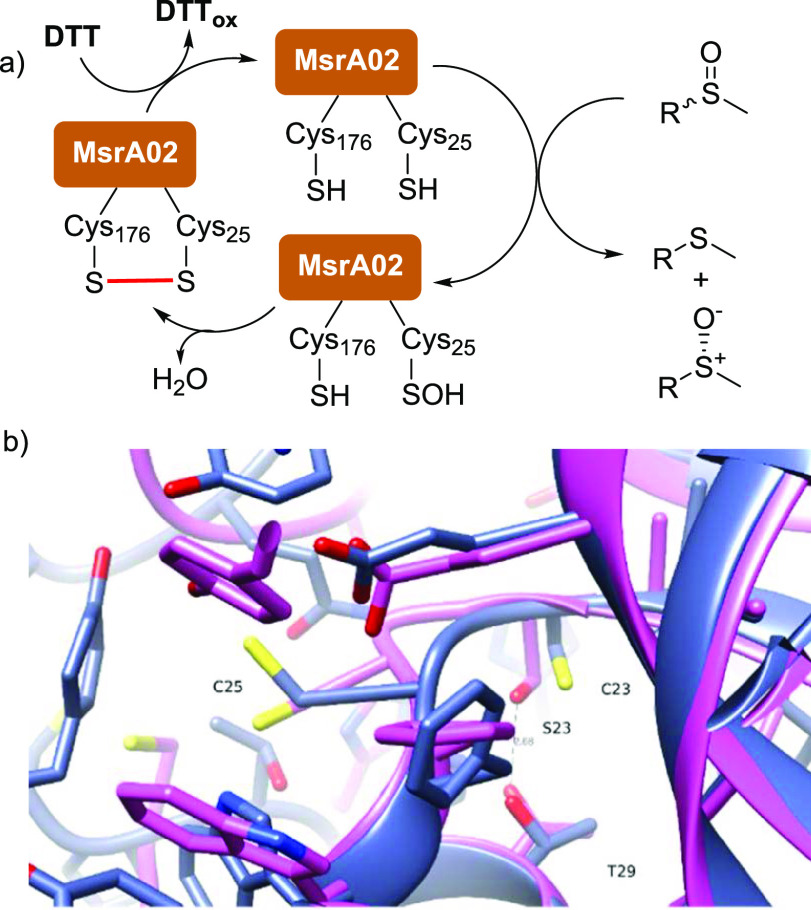
(a) Mechanism for the reduction of sulfoxides by MsrA02 biocatalysts; (b) comparison between the active sites of the wild-type (WT) MsrA02 enzyme (blue) and the C23S variant (pink). The serine hydroxide sidechain makes an H bond with threonine 29, significantly changing the loop containing the nucleophilic cysteine (C25).

First, a library of 15 Msr enzymes was cloned and overexpressed. Msr enzymes were selected from literature and homology searching in public databases to build a panel of diverse biocatalysts from different organisms as well as different Msr subclasses (Table 1). Msr encoding genes were cloned into pET28a and expressed in *the E. coli* BL21(DE3) expression strain. The gene expression was induced by the addition of 1 mM IPTG at 25 °C. After expression, the 15 Msr enzymes were prepared as lyophilized cell free extracts (CFE) and screened for the KR of the racemic sulfoxide **1a** to identify the best biocatalysts to afford the enantiomer **(*R*)-1a** in the highest yield and enantiomeric excess (ee). The initial biotransformation conditions for the screening were adopted from the literature.^16^ Each enzyme was resuspended in a 100 mM KPi buffer solution at pH = 8.0, together with 4.0 equiv of dithiothreitol (DTT), to regenerate the Msr enzymes (details on the role of DTT are reported below and in Figure 2a). An excess of DTT was initially used to promote full regeneration of the biocatalyst. Then, the substrate **1a** was dissolved in *i*PrOH (IPA), added to the enzyme mixture to initiate the reaction, and was left stirring at 30 °C for 24 h, after which the yields and ees of the enantiomer **(*R*)-1a**(20) were calculated using chiral HPLC (Table 1).

**Table 1:**
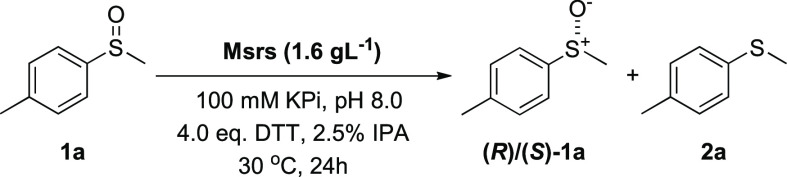
Screening of the Msr Panel Using Substrate **1a**

entry	Msr	Msr subclass	organism	**1a** yield^a^ %	(*R*)/(*S*)**1a** ee^b^ %	**1a** enant^b^
1	01	A	*Escherichia coli*	51	>99	(*R*)
2	02	A	*Saccharomyces cerevisiae*	50	>99	(*R*)
3	03	B	*Escherichia coli*	90	4	(*S*)
4	05	Free R	*Saccharomyces cerevisiae*	>99	<1	n.d.^c^
5	07	A	*Mycobacterium tuberculosis*	44	>99	(*R*)
6	08	AB hybrid	*Neisseria meningitidis*	44	>99	(*R*)
7	09	A	*Staphylococcus aureus*	55	>99	(*R*)
8	10	AB hybrid	*Thermococcus kodakarensis*	31	>99	(*R*)
9	11	A	*Streptomyces griseochromogenes*	54	>99	(*R*)
10	13	AB hybrid	*Streptococcus pneumoniae*	48	>99	(*R*)
11	15	AB hybrid	*Treponema denticola*	54	>99	(*R*)
12	16	A	*Klebsiella pneumoniae*	50	>99	(*R*)
13	17	A	*Salmonella schwarzengrund*	53	>99	(*R*)
14	18	A	*Serratia symbiotica*	>99	4	(*R*)
15	21	B	*Klebsiella oxytoca*	>99	1	n.d.^c^

a HPLC yields are reported. Yields were calculated using an Agilent Eclipse Plus C18 column and methyl phenyl sulfoxide as the internal standard.

b Determined by chiral HPLC using a Chiralpak IG column.

c Not determined.

Most MsrA enzymes showed excellent ees and conversions. Only MsrA18 (Table 1, entry 14), MsrB03, and MsrB21 (Table 1, entries 3 and 15) showed no activity. Interestingly, while frMsr05 from *Saccharomyces cerevisiae* showed no activity in the KR of **1a** (Table 1, entry 4), its MsrA variant, namely, MsrA02, from the same microorganism,^21^ furnished **(*R*)-1a** in high ee (>99%) and excellent HPLC yield (50%) (Table 1, entry 2). Since no MsrA biocatalyst from yeasts has been described to date, and due to the crystallographic data available in the literature,^21,22^ MsrA02 was selected for further optimization studies.

First, the optimal concentration of the biocatalyst, used as CFE, in the kinetic resolution of **1a** was investigated (Table 2). The concentration of **1a** was initially fixed at 8.0 mM and 4.0 equiv of DTT was used. The reactions (Table 2, entries 1–5) were carried out at 30 °C and stopped after 4 h when a 50% ^1^H-NMR yield of the enantiomer **(*R*)-1a** was observed, suggesting the completion of the KR reaction. The best concentration for MsrA02 was found at 1.0 gL^–1^, while lower concentrations led to poorer ees and yields. When the reaction was carried out in the absence of an enzyme, the sulfoxide **1a** was fully recovered as a racemate (Table 2, entry 5). Remarkably, biocatalytic transformation proved to tolerate concentrations of the substrate **1a** in the range of 32–64 mM (Table 2, entries 6–9), while at higher concentrations, **1a** was recovered as a racemic mixture (entry 9). Higher temperatures (37 °C) led to **1a** after 1 h with yield and ee (Table 2, entry 10) similar to those observed at 30 °C. Changing the cosolvent IPA to MeOH, EtOH or CH_3_CN did not affect the biotransformation outcome (Table 2, entries 11–14). Interestingly, when the reaction was carried out without any cosolvent or in the presence of DMSO, the sulfoxide **1a** was recovered as a racemate, indicating that no reduction took place (Table 2, entries 15–16). It is plausible that DMSO could act as a competitive substrate for MsrA02 or as an oxidant. Instead, in the absence of an organic cosolvent, **1a** showed poor solubility in aqueous buffer. The optimal amount of DTT used for the regeneration of MsrA02 was set at 1.1 equiv (compared to **1a**, Table 2, entry 17). Significantly, no biocatalytic transformation occurs in the absence of DTT, confirming its crucial role in the regeneration of MsrA02 (Table 2, entry 19 and Figure 2a).

**Table 2:**
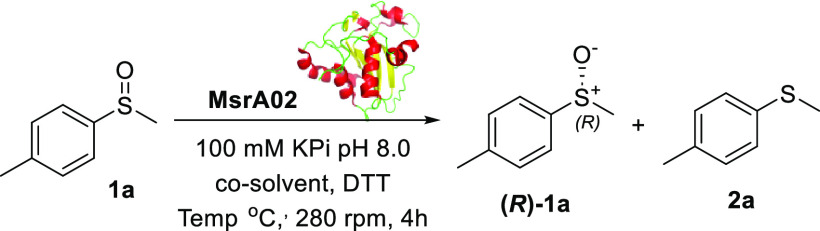
Optimization of the KR of **1a** with MsrA02

entry	MsrA02 (gL^–1^)^a^	**1a** (mM)	DTT (equiv)	cosolvent	temp °C	(*R*)-**1a** yield^b^ %	ee^c^ %
1	1.6	8.0	4.0	IPA	30	51 (51)^d^	98
2	1.0	8.0	4.0	IPA	30	54 (51)^d^	99
3	0.4	8.0	4.0	IPA	30	53 (52)^d^	98
4	0.1	8.0	4.0	IPA	30	71 (86)^d^	40
5		8.0	4.0	IPA	30	>99	<1
6	1.6	16	4.0	IPA	30	54	99
7	1.6	32	4.0	IPA	30	52	99
8	1.6	64	4.0	IPA	30	(48)^d^	99
9	1.6	128	4.0	IPA	30	54	5
10^e^	1.6	8.0	4.0	IPA	37	47	99
11	1.6	8.0	4.0	MeOH	30	46	>99
12	1.6	8.0	4.0	EtOH	30	50	>99
13	1.6	8.0	4.0	IPA	30	54	>99
14	1.6	8.0	4.0	CH_3_CN	30	49	>99
15	1.6	8.0	4.0	DMSO	30	90	5
16	1.6	8.0	4.0	Neat	30	>99	5
17	1.6	8.0	1.0	IPA	30	45	99
18	1.6	8.0	0.5	IPA	30	69 (62)^d^	64
19	1.6	8.0		IPA	30	>99	<1
20	1.0	32	1.1	IPA	30	53 (48)^f^	99
21	1.0	64	1.1	IPA	30	46	99
22	2.3^g^	32	1.1	IPA	30	(52)^d^	>99
23	10^h^	32	1.1	IPA	30	(52)^d^	>99
24^i^	1.6	8.0	4.0	IPA	30	52	>99

a MsrA02 used as CFE.

b ^1^H-NMR yields are reported.

c Determined by chiral HPLC using the Chiralpak IG column.

d HPLC yield is reported.

e 1 h reaction time.

f Isolated yield.

g Pure MsrA02 enzyme was used.

h Whole cell MsrA02 was used.

i Reaction time was 18 h.

The optimal reaction conditions were finally combined and set at 1 gL^–1^ of the biocatalyst, 32 mM of **1a**, 1.1 equiv of DTT, and 30 °C, leading to **(*R*)-1a** with 48% isolated yield and 99% ee (Table 2, entry 20). Remarkably, similar results were obtained when a higher concentration of the substrate (64 mM) was used (Table 2, entry 21), proving the robustness of this biotransformation. The use of MsrA02 as a whole cell biocatalyst (Table 2, entry 23) or as pure enzyme (Table 2, entry 22, see the Supporting Information for the purification procedure) led to enantiomerically pure **(*R*)-1a** in identical conversion and ee to the CFE, confirming that the observed reactivity was solely a result of MsrA02 and not from any other proteins present in the cell lysate. Finally, longer reaction times up to 18 h (Table 2, entry 24) proved to not affect the biotransformation outcome. With the best conditions for the MsrA02 biocatalyzed KR of **1a** in hand (Table 2, entry 20), the substrate scope of the reaction was then investigated. For some substrates where MsrA02 performed poorly, the biotransformation was also attempted with other biocatalysts selected from the initial screening (Table S3). All results are reported in Table 3. Isolated yields are reported for the reaction products.

**Table 3:**
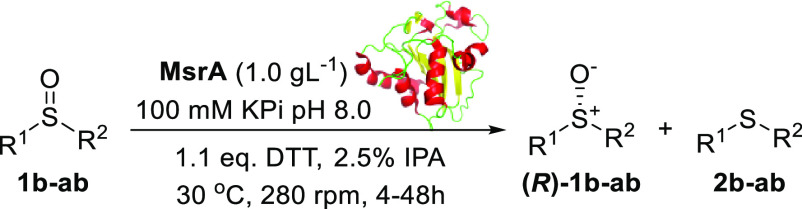
Substrate Scope of the KR of Sulfoxides **1b-ab**

entry	cmpd.	R^1^	R^2^	Msr	time (h)	isolated yield %^c^	ee %^b^
1	**1b**	Ph	Me	02	4	35	>99
2	**1c**	4-F-Ph	Me	02	6	26	>99
3	**1d**	4-Br-Ph	Me	02	6	41	>99
4	**1e**	4-Cl-Ph	Me	02	4	38	>99
5	**1f**	3-Cl-Ph	Me	02	6	40	>99
6	**1g**	2-Cl-Ph	Me	02	28	30	>99
7	**1h**	4-Ac-Ph	Me	02	4	50	>99
8	**1i**	4-MeO-Ph	Me	02	4	43	>99
9	**1j**	3-MeO-Ph	Me	02	6	35	>99
10	**1k**	3-Me-Ph	Me	02	6	48	>99
11	**1l**	4,2-Me-Ph	Me	02	81	41	>99
12	**1m**	2-Naph	Me	02^c^	28	n.d.^d^	74
				10	24	69^e^	30
				10^f^	24	45^e^	>99
13	**1n**	1-Naph	Me	02	24	n.d.^d^	16
14	**1o**	Bn	Me	02	24	29	94
15	**1p**	Dodecyl	Me	02	24	46	>99
16	**1q**	CH_3_CO(CH_2_)_2_	Me	02	48	40	>99
17	**1r**	PhSO(CH_2_)_2_	Me	02	8	39	>99^j^
18	**1s**	Ph	Et	02	48	47	90
19	**1t**^c^	4-Me-Ph	Et	02	48	36	98
20	**1u**^c^	4-Br-Ph	Et	02	48	55	80
21	**1v**^g^	Bn	Et	02	24	36	96
22	**1w**	Ph(CH_2_)_2_	Et	02	8	40	99
23	**1x**^g^	2-PyCH_2_	Et	02	24	n.d.^d^	54
				01	24	n.d.^d^	20
24	**1y**^h^	Ph	*n*Pr	02	24	n.d.^d^	<1
25	**1z**^h^	4-MeO-Ph	*n*Pr	02	24	n.d.^d^	<1
				01	24	n.d.^d^	10
26	**1aa**^i^	Ph	Vinyl	02	48	<5^e^	>99
27	**1ab**	Ph	Allyl	02	7d	n.d.^d^	16
				01	24	n.d.^d^	24

a Isolated yields after chromatographic purification of the (*R*)-sulfoxide.

b Determined by chiral HPLC using Chiralpak column IG or IC or Chiracel OD-H.

c 2.0 gL^–1^ CFE MsrA02.

d Not determined.

e Conversion determined by HPLC.

f 8 mM substrate, 1.0 gL^–1^.

g 10 gL^–1^ CFE MsrA02.

h 8 mM substrate, 10 gL^–1^ CFE MsrA02.

i 10 μM purified MsrA02.

j Two diastereoisomers, each one with >99% ee.

Sulfoxides **1b–l** were all resolved by MsrA02 with excellent ee (99%) and high isolated yields (Table 3, entries 1–11). Even if the maximum yield expected for these KR reactions was 50%, the isolated yield detected after chromatographic purification was, in a few cases, slightly lower due to the difficulties associated with the extraction of the sulfoxide compounds from the buffer media with organic solvents like DCM or AcOEt. Nevertheless, in most cases, the isolated yields obtained were well above 40%. The (*R*)-enantiomer of the 2-naphtyl derivative **1m** was obtained with high ee (74%) using MsrA02 (Table 3, entry 12). Interestingly, another AB hybrid Msr enzyme, namely, MsrA10 from *Thermococcus kodakarensis*, which was more active but less stereoselective on substrate **1a** (Table 1), proved to be highly effective in the KR of **1m** when used at 8 mM concentration, affording the (*R*)-enantiomer with excellent >99% ee and 45% conversion. On the other hand, the regioisomer 1-naphtyl derivative **1n**, proved to be a poor substrate for both MsrA02 and MsrA10 (Table 3, entry 13). The sulfoxides **1o–q** bearing benzyl or alkyl group on the sulfur atom were also reduced by MsrA02 (Table 3, entries 14–16), affording the corresponding (*R*)-enantiomers with high isolated yields (up to 46%) and excellent ee (up to 99%). Substrate **1r** bears two sulfoxide groups, and thus, it exists as two pairs of enantiomers, **(*R,S*)-1r** and **(*S,R*)-1r**, and **(*R,R*)-1r** and **(*S,S*)-1r** (Scheme S1). MsrA02 was able to reduce only the (*S*)-sulfoxide moiety adjacent to the methyl group, leaving the others unreacted. Thus, the diastereoisomers **(*R,S*)-1r** and **(*S,S*)-1r** were reduced by MsrA02 into the enantiomers **(*R*)-2r** and **(*S*)-2r**, while the diastereoisomers **(*S,R*)-1r** and **(*R,R*)-1r** were recovered in a 1:1 ratio, each one with excellent >99% ee (Table 3, entry 17).

Remarkably, MsrA02 showed excellent activity also on the substrates **1s–w** bearing an Et substituent on the sulfur atom with high yields and excellent ees (Table 3, entries 18–22). The pyridyl derivative **(*R*)-1x** was obtained with only 54% ee when MsrA02 was used. Attempts to find another suitable MsrA biocatalyst to resolve **1x** failed and only MsrA01 showed little enantioselectivity, affording the (*R*)-enantiomer with 20% ee (Table 3, entry 23). Similarly, the bulkier propyl derivatives **1y** and **1z** as well as allyl substrates **1ab** were not or poorly resolved by MsrA02 (Table 3, entries 24–25 and entry 27), while high ee (>99%) was detected for the smaller vinyl derivative **1aa** (Table 3, entry 26). However, **1aa** proved to be unstable and it was obtained in low HPLC yields.

### Mechanism of MsrA Biocatalytic Reduction

The substrate scope study of this biocatalytic transformation clearly showed the ability of MsrA02 to catalyze the KR of sulfoxides bearing a methyl and, remarkably, an ethyl substituent on the sulfur atom. However, bulkier substrates bearing a propyl or an allyl substituent were not reduced by MsrA02 or other MsrA wild-type (WT) enzymes. Thus, the possibility to mutate MsrA enzymes enabling them to catalyze the reduction of bulkier substrates was investigated. To design improved MsrA mutants via rational mutagenesis, the mechanism of the MsrA02 biocatalyzed reaction was first investigated in depth. The generally accepted mechanism for the reduction of MetSO and other sulfoxides by MsrA enzymes involves at least two catalytic cysteine (Cys) residues (Figure 2a).^23^ The first cysteine attacks and reduces the sulfoxide group forming a sulfenic acid intermediate (Cys-SOH), which then reacts with the second cysteine residue forming an intramolecular disulfide bond via dehydration. *In vivo*, the oxidoreductase thioredoxin (Trx) breaks the disulfide bond, leading to the regeneration of the MsrA enzymes, while *in vitro* Trx is replaced with the inexpensive sacrificial co-substrate DTT.

The biocatalyst MsrA02 contains five cysteine residues (Cys23, Cys25, Cys44, Cys68, and Cys176) which could be involved in the reduction of sulfoxides. According to the crystal structure analysis,^21^ the two residues involved in the catalytic cycle in MsrA02 are likely to be Cys25 and Cys176. To confirm the role of the five cysteines in the reduction of **1a**, single point mutations were carried out on MsrA02. Each cysteine was mutated to serine and all five MsrA02 mutants (MsrA02 C25S, C176S, C23S, C44S, and C68S) were then overexpressed and purified as N-terminal His-tagged fusion proteins. The mutants were assayed with sulfoxide **1a** as either CFE or pure enzymes (Table 4). Experiments were carried out at different concentrations of the biocatalysts. Mutants C25S and C176S dramatically affected the outcome of the sulfoxide reduction, and in both cases, **1a** was recovered as a racemate (Table 4, entries 2 and 5). This clearly confirms that these two Cys residues are involved in the catalytic cycle and in the sulfoxide reduction. Mutation C44S did not affect the biotransformation and **(*R*)-1a** was obtained with 99% ee, confirming that Cys44 has no role in the catalytic cycle (Table 4, entry 3). The mutation C68S also had no effect on the catalytic activity of the enzyme, as even at low concentrations (0.23 gL^–1^ of pure enzyme), >99% ee of **(*R*)-1a** was obtained (Table 4, entry 4). Interestingly, the mutation C23S, led to **(*R*)-1a** with poor ee, especially at low concentration of the enzyme (Table 4, entry 1).

**Table 4:**
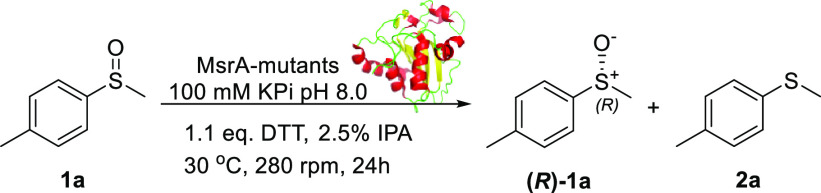
Single Point Mutation of MsrA02

entry	single point mutation	enzyme conc. (gL^–1^)^a^^,^^b^	(*R*)-**1a** ee%
1	C23S	1.0	26
		10	67
		40	80
2	C25S^c^	0.23	<1
		2.3	8
		9.2	21
3	C44S	1.0	9
		10	99
		40	>99
4	C68S^c^	0.23	>99
		2.3	>99
		9.2	>99
5	C176S	1.0	4
		10	26
		40	36
6	Wild type	1.0	>99

a Enzyme used as CFE.

b gL^–1^ of protein concentration calculated from 10, 100, and 400 μM.

c Purified enzyme was used.

Computational studies were carried out to show that even if Cys23 is not directly involved in the catalytic cycle or in the attack and reduction of the sulfoxide **1a**, the replacement of this cysteine with a serine alters the binding pocket. In the C23S mutant, the smaller serine hydroxide makes a new hydrogen bond with Thr29 (Figure 2b), leading to a significant change in the size and position of the loop containing the nucleophilic Cys25, which explains the poor ee observed.

**Figure 3:**
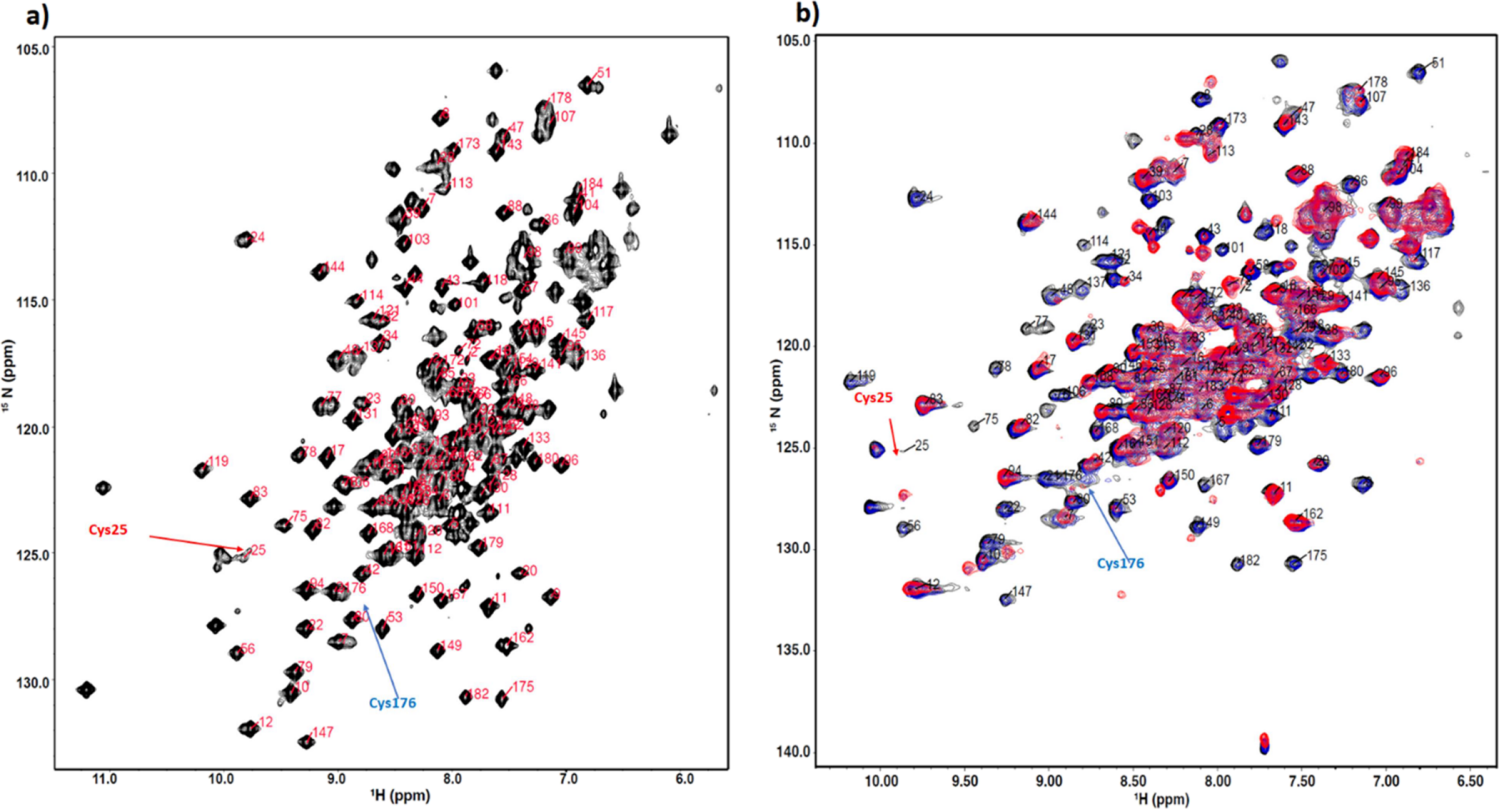
^15^N HSQC spectrum of MsrA02. (a) Assigned ^15^N HSQC of MsrA02 in its free, reduced form. (b) ^15^N SOFAST HMQC of MsrA02 collected at increasing 0 (black), 0.5 (blue), and 1 mM (red) of **1a**.

To further investigate and elucidate the dynamics of the catalytic cycle, an NMR structural study of the biocatalyst MsrA02 was carried out. ^15^N- and ^13^C-labeled MsrA02 enzymes were expressed and purified and the biocatalytic reduction of the sulfoxide **1a** was studied by ^15^N-^1^H NMR, allowing residue-specific information to be collected. Using a standard suit of triple resonance NMR experiments, around 70% of the backbone resonances were identified, allowing us to identify residues involved in the catalytic activity (Figure 3a).

**Figure 4:**
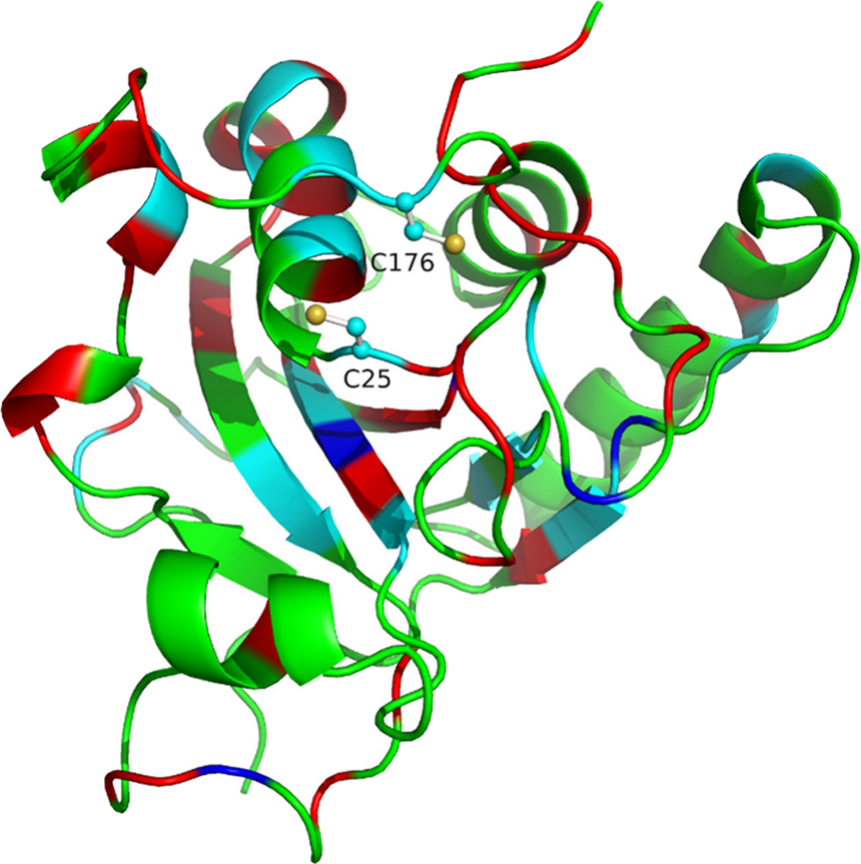
Cartoon representation of the MsrA02 structure (PDB: 3PIL) with the residues involved in the catalytic cycle of **1a** highlighted. Residues with resonances undergoing significant intensity losses upon the addition of 0.5 mM or 1 mM of **1a** are labeled in blue or red, respectively. Residues with resonances undergoing significant chemical shift differences between the free and bound forms are labeled in cyan. C25 and C176 sidechains are shown for clarity.

SOFAST HMQC experiments were subsequently collected at increasing substrate **1a** concentrations in the presence of DTT, resulting in the modulation of both intensities and chemical shift positions of a subset of NMR resonances following a slow exchange regime (Figure 3b). A comparison of the decay of intensities upon substrate addition at 0.5 and 1 mM show that at a lower concentration of **1a**, the most affected region of the protein is around Cys25, including the beta strand at positions 74–76 (Figure 4, blue), suggesting that the catalytic activity starts with the engagement of Cys25 with the substrate. At 1 mM of **1a**, large intensity changes extend from the Cys25 to the whole beta strand between residues 74–80 and the area around it, reaching Cys176 and its neighboring residues (Figure 4, red).

Such changes suggest that once the substrate is bound to Cys25, the area around Cys176 is involved in the catalytic cycle, supported by the changes occurring in the helical region between residues 161–172. The largest chemical shift changes between the free and bound state are also found in the patch surrounding Cys25, Cys176, and the central beta strand (Figure 4, cyan). The spectrum for the bound state of the protein shows broader peaks, indicating that substrate binding induces significant dynamics on the structure of MsrA02. These titration experiments present a slow exchange binding regime, characteristic of high affinity binding. The specificity of these spectral changes due to binding to substrate **1a** was confirmed by the addition of DTT, which reverts the spectrum of MsrA02 back to the apo form (Figure S2). This indicates that the chemical shift changes observed are specific to substrate **1a** and not caused by alterations of pH, salt concentrations, or any other effect.

### Rational Mutagenesis

The generation of mutant enzymes able to accept sulfoxide substrates bearing substituents on the sulfur atom other than a methyl group was then investigated. Molecular dynamics simulations were performed on the WT enzyme MsrA02.

**Figure 5:**
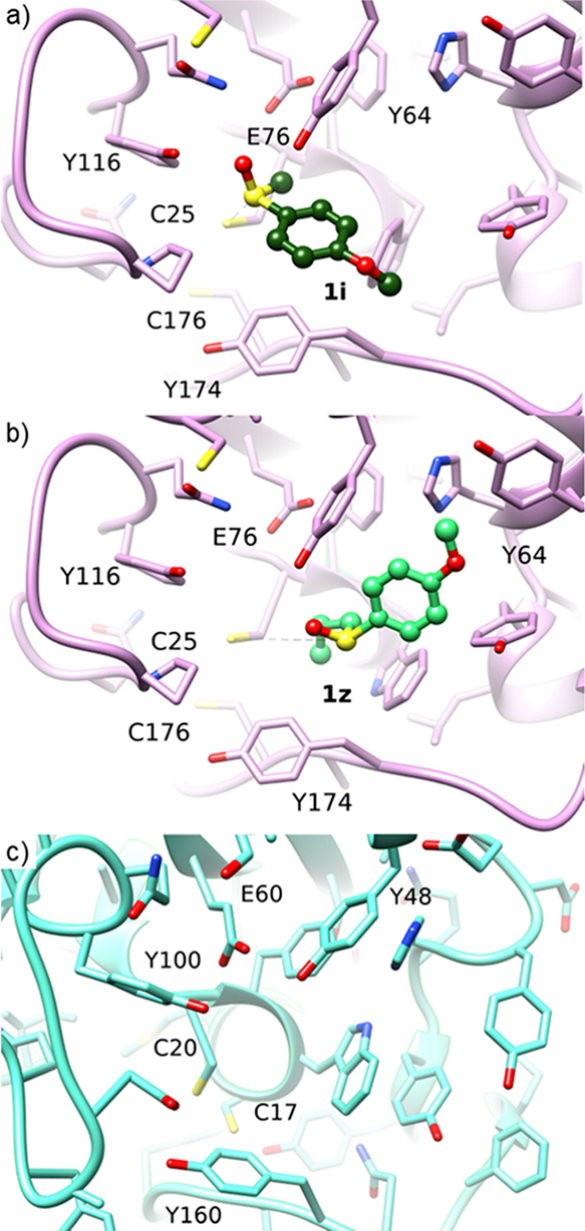
(a) docking of sulfoxide **1i** in MsrA02; the Tyr174 residue seems important to keep the Cys25 close to the sulfoxide group. (b) docking of the sulfoxide **1z** in MsrA02. (c) MsrA10 enzyme. The second cysteine residue of the enzymes is not in the C-terminal region.

The substrates **1i** and **1z** bearing, respectively, a methyl and a *n*-propyl substituent on the sulfur atom, were chosen for docking into the active site of WT MsrA02 (Figure 5). Initial MD studies show that, when bonded with substrate **1i**, Cys25 is in proximity (3.55 Å) to the sulfoxide group for nucleophilic attack (Figure 5a). Conversely, when complexed with the bulkier **1z**, the *n*-propyl group hinders the binding in sufficient proximity to Cys25 (6.6 Å), disfavoring the nucleophilic attack on the sulfoxide group (Figure 5b). This explains the lack of activity of MsrA02 on **1z**. Previous crystallographic studies on MsrA from *S. cerevisiae* and MetSO have shown that, in addition to Cys25 and Cys176, other essential residues for the reaction mechanism are Tyr64, Glu76, and Tyr116, as they bind and stabilize the oxygen of the sulfoxide, and Phe26, Trp27, which stabilize the methyl group of MetSO instead (Figure S1).^21,24−27^ Additionally, as shown in Figure 5, Tyr174 seems crucial to keep the Cys176 close to the adduct between the enzyme and the substrate to facilitate sulfide release and enzyme regeneration. Since the amino acids Phe26 and Trp27 are responsible for the interaction of the methyl group of MetSO with the MsrA02 binding pocket, we hypothesized that mutations of these residues would help accommodate larger alkyl substituents. Moreover, the mutation of other amino acid residues in the C-terminal and the α2-helix regions of MsrA02 were also explored (Table S1). Therefore, a series of nine mutant enzymes was computationally designed by modifying the WT MsrA02 amino acid residues to allow a shorter distance between the sulfoxide group of **1z** and the Cys25 residue. The design of appropriate mutants was complicated by the fact that in silico mutations in the C-terminal region of MsrA02 seemed deleterious for the stability of the enzyme. Hence, enzyme MsrA10, which showed good activity on the substrate **1m**, was also investigated for rational mutagenesis. In fact, since the second cysteine residue involved in the catalytic cycle of MsrA10 (corresponding to Cys176 in MsrA02 from a catalytic point of view) is not in the C-terminal region of the enzyme (Figure 5c), we hypothesized that mutations in this area would not affect the stability of the mutants.

**Figure 6:**
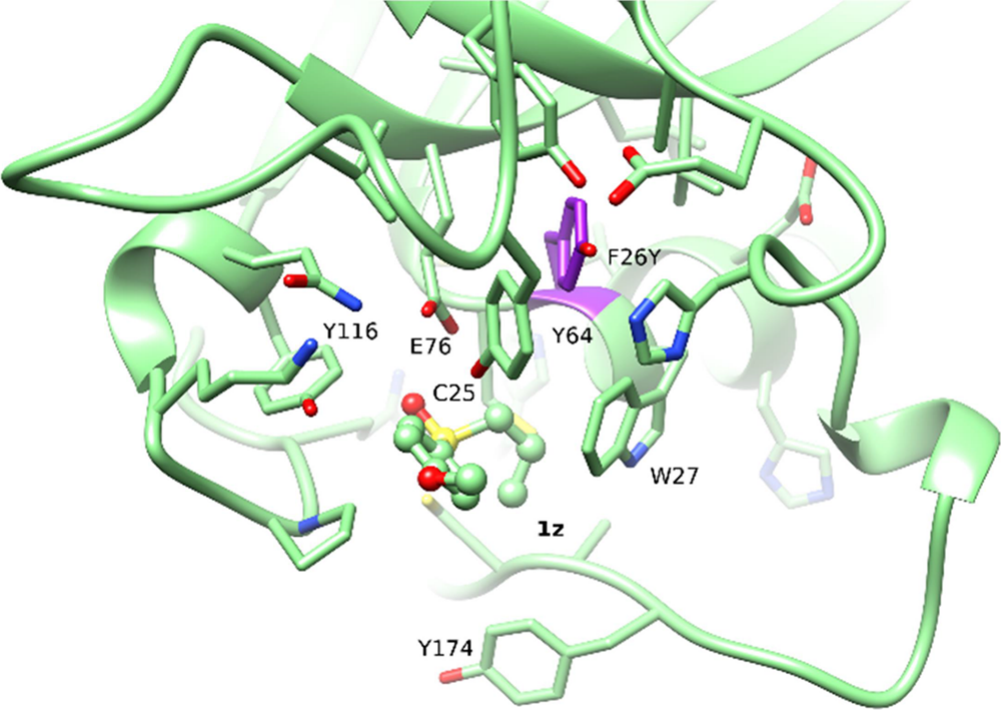
**1z** binding pose for the mutant MsrA33 (F26Y). In the WT enzyme, the substrate is far from Cys25, caused by steric hindrance between the propyl group of **1z** and the enzyme (Figure 5b). In the mutant, the substrate is in proximity with Cys25 favoring the nucleophilic attack.

Thus, three additional mutants of MsrA10 were designed and produced. Finally, seven new wild-type MsrA enzymes showing different C-terminal regions from MsrA02 were selected from the literature and homology searching in public databases and prepared for screening on substrate **1z**. The new WT enzymes and MsrA02 and MsrA10 mutants were cloned and expressed in *E. coli* under the same conditions as the original Msr enzyme panel and screened, as CFE, with the *n-*propyl substrate **1z** (Table S1). Among all the mutants, only mutant F26Y (MsrA33) was able to reduce **1z**, providing the (*R*)-enantiomer with 43% ee. Figure 6 shows that the F26Y mutation creates additional space for the *n*-propyl substituent of substrate **1z**, bringing Cys25 and the sulfoxide at 5.0 Å distance.

The biocatalyzed reduction of **1z** with the MsrA02-mutant MsrA33, as CFE biocatalyst, was then optimized (Table S2). Enantiomer **(*R*)-1z** was obtained in 42% HPLC yield and 99% ee when the biotransformation was carried out for 48 h using 8 mM of racemic **1z**, 40 gL^–1^ of MsrA33, and 4 equiv DTT^28^ (Table S2, entry 16). The substrate scope of the biocatalytic reduction of various sulfoxides with the mutant MsrA33 was finally investigated. The results are reported in Table 5. All the sulfoxides **1z**, **1y**, and **1ac–af** bearing an *n-*propyl substituent on the sulfur atom were obtained with good-high conversion (Table 5, entries 1–6). Remarkably, the (*R*)-enantiomers of the derivatives **1z**, **1ac**, and **1ad** were obtained with excellent ees (up to 99%). The bulkier sulfoxide **1ad** bearing an *n*-butyl substituent was also reacted with MsrA33, but lower ee was detected for **(*R*)-1ad**. On the other hand, the ethyl-substituted sulfoxides **1u** and **1x** (Table 5, entries 8–9), which were poorly reduced by MsrA02, were formed with good conversion and excellent ee (>99 and 96%).

**Table 5:**
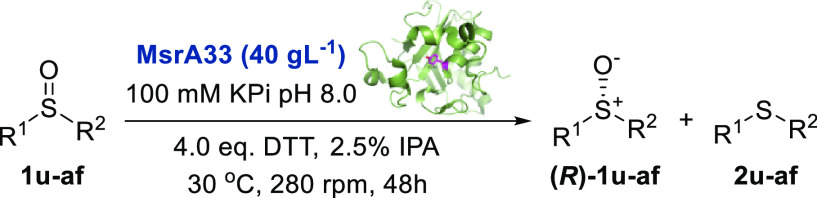
Scope of the MsrA33 Biocatalyzed Deracemization of Sulfoxides

entry	substrate	R^1^	R^2^	(*R*)-sulfoxide conv. %^a^	ee %^b^
1	**1z**	4-MeOPh	*n*Pr	42	>99
2	**1y**	Ph	*n*Pr	37	80
3	**1ac**	4-MePh	*n*Pr	40	99
4	**1ad**	4-ClPh	*n*Pr	46	92
5	**1ae**	4-BrPh	*n*Pr	56	76
6	**1af**	3-MeOPh	*n*Pr	51	64
7	**1ad**	4-MeOPh	*n*Bu	87	12
8	**1u**	4-BrPh	Et	39	>99
9	**1x**	2-PyCH_2_	Et	44	96

a Conversions determined by reverse-phase HPLC.

b Determined by chiral HPLC using Chiralpak column IG, IC, ID, or Chiracel OD-H.

## Conclusions

MsrAs are a promising class of enzymes able to catalyze the stereoselective reduction of chiral sulfoxides into enantiomerically pure products. The use of reductive instead of oxidative enzymes in the synthesis of enantiomerically pure sulfoxides offers substantial advantages from a synthetic point of view, such as the use of cheap cosubstrate DTT in place of the expensive cofactor NAD(*P*)H and related recycling systems or avoiding peroxide reagents which can lead to overoxidation byproducts. Thus, the discovery and preparation of new MsrA enzymes is an important development as it expands the biocatalysis toolbox and it provides an alternative biocatalytic strategy to access enantiomerically pure sulfoxides especially from substrates bearing multiple oxidation sites, which are incompatible with existing oxidizing biocatalysts.

To date, only few MsrA biocatalysts have been described in the literature and little is still known on their potential and industrial application. This work has expanded the range of MsrA enzymes suitable for biocatalytic applications, leading to the identification of MsrA02 from *Saccharomyces cerevisiae*. MsrA02 proved to be a robust and efficient biocatalyst in the KR of a large variety of aromatic and aliphatic racemic sulfoxides and to work well at high substrate concentrations as CFE, pure enzyme or whole cell biocatalyst. Moreover, in this work, a study of the catalytic mechanism of the biotransformation has been carried out, through mutagenesis and structural biology NMR studies, highlighting the amino acid residues and the dynamics involved in the reduction of sulfoxides. Such studies have led to the *in silico* design and preparation of a novel MsrA mutant enzyme, which is remarkable in its ability to catalyze the reduction of sulfoxides bearing a diverse range of non-methyl substituents on the sulfur atom. This development significantly expands the substrate scope of MsrAs and thus overcomes a major limitation of currently available MsrA biocatalysts. To the best of our knowledge, this is the first example of rational mutagenesis of MsrA enzymes, and it can pave the way to exploit and expand the scope and application of such biocatalysts both in academia and industry.

## Supporting Information Available

Detailed experimental procedure; compound characterization data; copies of NMR spectra of sulfoxides; and copies of HPLC traces of biocatalytic reaction products (PDF)

## Abbreviations

Msr: methionine sulfoxide reductase
MetSO: methionine sulfoxide
KR: kinetic resolution
FAD: flavin adenine dinucleotide
FMN: flavin mononucleotide
WT: wild type
CFE: cell free extract
