# Supplementary material for: Discovery and Rational Mutagenesis of Methionine Sulfoxide Reductase Biocatalysts To Expand the Substrate Scope of the Kinetic Resolution of Chiral Sulfoxides
Source: ACS Catal. 2023 Mar 23;13(7):4742–51. doi: 10.1021/acscatal.3c00372 (PMC10088026; doi:10.1021/acscatal.3c00372)
Supplement: Supplementary file 1 — cs3c00372_si_001.pdf [file cs3c00372_si_001.pdf]

Discovery and rational mutagenesis of methionine sulfoxide reductase (MsrA) biocatalysts to expand the substrate scope of the kinetic resolution of chiral sulfoxides.

Silvia Anselmi,[a] Alexandra T. P. Carvalho,[b] Angela Serrano-Sanchez,[d] Jose L. Ortega-Roldan,[d] Jill Caswell,[b] Iman Omar,[a,e] Gustavo Perez-Ortiz,[e] Sarah M. Barry,[e] Thomas S. Moody[b,c,] and Daniele Castagnolo[a,*]*

[a]Department of Chemistry, University College London, 20 Gordon Street, WC1H 0AJ, London, United Kingdom; [b]Department of Biocatalysis & Isotope Chemistry, Almac, 20 Seagoe Industrial Estate, Craigavon, BT63 5QD, United Kingdom; [c]Arran Chemical Company Limited, Unit 1 Monksland Industrial Estate, Athlone, Co. Roscommon, Ireland; [d]School of Biosciences, University of Kent, Canterbury, CT2 7NJ, United Kingdom; [e]Department of Chemistry, King's College London, 7 Trinity Street, SE1 1DB, London, United Kingdom.

[*]Corresponding Authors: d.castagnolo@ucl.ac.uk; tom.moody@almacgroup.com.

Supporting information

Table of contents

1 General S3
2 Biocatalysis S4
2.1 Preparation of Msr WT enzymes panel S4
2.2 Purification of *S. cerevisiae* MsrA02 from CFE S6
2.3 General procedure for the kinetic resolution of substrates 1a-ab using MsrA02 .... S7
2.4 Preparation of C25S and C68S MsrA02 .... S8
2.5 Activity assay of C25S and C68S MsrA02 .... S9
2.6 Activity assay of C23S, C44S and C176S MsrA02 .... S9
2.7 Identification of other Msrs for the kinetic resolution of bulkier sulfoxides S9
2.8 Screening of MsrA31-49 mutants and new WT MsrAs S9
2.9 Optimisation of the reaction conditions using MsrA33 .... S11
2.10 General procedure for the kinetic resolution of sulfoxides using MsrA33 .... S11
2.11 Additional screening of MsrA enzymes on bulky substrates S12
2.12 Mechanism for the reduction of MetSO by MsrA02 .... S13
3 Computational Methods S14
3.1 Modelling S14
3.2 Molecular Docking S14
3.3 Molecular Dynamics Simulations S14
4 NMR studies S15
4.1 Production of N^{15} and C^{13} labelled MsrA02 and MsrA02_C25S S15
4.2 NMR spectroscopy S15
5 Chemistry S16
5.1 General procedure for the synthesis of racemic sulfoxides S16
6 Characterisation of compounds S17
6.1 Characterisation data of the substrate sulfoxides 1a-ag and sulfide 2r S17
6.2 Isolated yields and optical rotation data of (*R*)-sulfoxides S25
6.3 Copies of NMR spectra S26
6.4 HPLC analysis and traces S57
7 References S91

1 General

Reagents and solvents were used as supplied from the vendor without further purification. Thin layer chromatography plates (Merk, silica gel 60 F_{254}, aluminium backed) were viewed under UV light and stained using $KMnO_4$ developed using heat. $MgSO_4$ (Sigma Aldrich, anhydrous ≥ 98.0 %) was used as the drying agent. Column chromatography was performed on silica gel for flash chromatography (Sigma Aldrich, 40-63 µm particle size, 60 Å pore size). Microwave irradiations were conducted using a CEM Discover Synthesis Unit. Products were characterised by ^{1}H NMR, ^{13}C NMR and ^{19}F NMR spectra where applicable obtained from one of the following: a) Bruker (Germany) Ascend400 Spectrometer (δ_H 400 MHz, δ_C 101 MHz, δ_F 376 MHz) at 300 K; b) Bruker (Germany) Avance III 400 (δ_H 400 MHz, δ_C 101 MHz) at 300 K; c) Bruker (Germany) Avance Neo 500 (δ_H 500 MHz, δ_C 126 MHz) at 300 K. Chemical shifts are reported in ppm relative to the reference peaks of the solvents: $CDCl_3$ (^{1}H NMR 7.26 and ^{13}C NMR 77.16) unless stated otherwise. Coupling constants (*J*) are reported in Hz, multiplicities are specified as singlet (s), doublet (d), triplet (t), doublet of doublet (dd), doublet of quartet (dq), doublet of doublet of doublet (ddd), triplet of doublet (td), multiplet (m).

HPLC/UPLC and LC-MS analysis was carried out using one of the following: a) Agilent series 1100 LC/MSD system coupled with UV detector at l = 254 nm, 2 = 240 nm and an atmospheric pressure chemical ionization (APCI); b) Agilent series 1250 UPLC system coupled with UV detector at l = 254 nm, 2 = 240 nm. The columns used were Chiralpak IC® (0.5µm, 4.6mm X 250mm), Chiralpak IG® (5µm, 4.6mm X 250mm), Chiralpak ID® (0.5µm, 4.6mm X 250mm), and Chiracel OD-H (0.5µm, 4.6mm X 250mm) supplied by Daicel. Hexane, isopropanol (IPA) and ethanol (EtOH) were used as an isocratic mobile phase system for all columns.

Mass spectra were acquired in positive mode scanning over the mass range of 50 – 1500. The following ion source parameters were used: drying gas flow, 12 mL/min; nebulize pressure, 35 psi; and drying gas temperature, 350 ºC.

Protein purification was performed on an ÄKTA Fast Protein Liquid Chromatography (FPLC) Instrument (GE Healthcare) with a UV detector set at 280 nm. Purification was carried out a 5 mL His-Trap™ Fast Flow nickel affinity column (GE) and a HiLoadTM 16/600 Superdex 200pg size exclusion chromatography column (GE) where appropriate. All buffers were adjusted using 5M NaOH or 5M HCl.

2 Biocatalysis

2.1 Preparation of Msr WT enzymes panel

The MSR genes were synthesised in pET28a(+) and transformed into BL21(DE3) *E. coli* and plated on nutrient agar plates containing 50 µg/mL kanamycin which were incubated overnight. Single colonies for each MSR gene were used to inoculate a 10 mL culture tube containing LB media (10 g tryptone, 5 g yeast extract and 10 g NaCl per litre) which was incubated at 37 °C overnight with agitation. This 10 mL primary culture was then used to inoculate a 1 L culture of LB media containing kanamycin in a 2 L shake flask. The culture was incubated at 37 °C in an orbital shaker until OD_{600} reached ~0.6. At this point the incubation temperature was reduced to 25 °C and 1 mM IPTG added to induce protein expression overnight. Following the overnight incubation, the culture was centrifuged (18,000 rpm, 45 min, 4 ºC) to pellet the bacterial material, and the pellet resuspended in 0.1 M Potassium Phosphate, pH 7.4. The pellet was then subjected to sonication (10 secs on, 10 sec off for 6 cycles) to lyse the *E. coli* cells. This lysate was centrifuged for 15 min at 4000 rpm to pellet the cellular debris. The supernatant was retained and lyophilised to generate freeze dried cell free extract (CFE) to be used in all subsequent experiments.

Enzyme	Type	Uniprot code	Sequence
Msr01	msrA	P0A744	MSLFDKKHLVSPADALPGRNTPMPVA TLHAVNGHSMTNVPDGMEIAIFAMGCF WGVERLFWQLPGVYSTAAGYTGGYTPNP TYREVCSGDTGHAEAVRIVYDPSVISYEQ LLQVFWENHDPAQGMRQGNDHGTQYRSAI YPLTPEQDAAARASLERFQAAMLAADDD RHITTEIANATPFYYAEDDHQQYLHKNPY GYCGIGGIGVCLPPEA
Msr02	msrA	B3LS55	MSSLISKTIKYDPAKDKLITLACGCFWGTE HMYRKYLNDRIVDCKVGYANGEESKKDS PSSVSYKRVCGGDTDFAEVLQVSYNPKVIT LRELTDFFFRIHDPTTSNSQGPDKGTQYRSG LFAHSDADLKELAKIKEEWQPKWGNKIAT VIEPIKNFYDAEEYHQLYLDKNPQGYACPT HYLREM
Msr03	msrB	P0A747	MANKPSAEELKKNLSEMQFYVTQNHGTEP PFTGRLLHNKRDGVYHCLICDAPLFHSQTK YDSGCGWPSFYEPVSEESIRYIKDLSHGMQ RIEIRCGNCDAHLGHVFPDGPQPTGERYCV NSASLRFTDGENGEEING
Msr05	free R msr	P36088	MGSSTGFHHADHVNYSSNLNKEEILEQLLL SYEGLSDGQVNWVCNLSNASSLIWHAYKSL AVDINWAGFYVTQASEENTLILGPFQGKVA CQMIQFGKGVCGTAASTKETQIVPDVNKYP GHIACDGETKSEIVVPIISNDGKTLGVIDIDC LDYEGFDHVDKEFLEKLAKLINKSCVFK
Msr07	msrA	P9WJM5	MTSNQKAILAGGCFWGLQDLIRNQPGVVST RVGYSGGNIPNATYRNHGTHAEAVEIIFDPV TDYRTLLEFFFQIHDPTTKDRQGNDRGTSYR

			SAIFYFDEQQKRIALDTIADVEASGLWPGKV VTEVSPAGDFWEAEPEHQDYLQRYPNGYTC HFVRPGWRLPRRTAESALRASLSPELGT
Msr08	msrA msrB hybrid	Q9JWM8	MKHRTFFSLCAKFGCLLALGACSPKIVDAGA ATVPHTLSTLKTADNRPASVYLKKDKPTLIKF WASWCPLCLSELGQTEKWAQDAKFSSANLIT VASPGFLHEKKDGDFQKWYAGLNYPKLPVVT DNGGTIAQSLNISVYPSWALIGKDGDVQRIVK GSINEAQALALIRDPNADLGSLKHSFYKPDTQ KKDSKIMNTRTIYLAGGCFWGLEAYFQRIDGV VDAVSGYANGNTKNPSYEDVSYRHTGHAETV KVTYDADKLSLDDILQYFFRVVDPTSLNKQGN DTGTQYRSGVYYTDPAEKAVIAAALKREQQK YQLPLVVENEPLKNFYDAEEYHQDYLIKNPNG YCHIDIRKADEPLPGKTKTAPQGKGFDAATYK KPSDAELKRTLTEEQYQVTQNSATEYAFSHEY DHLFKPGIYVDVVSGEPLFSSADKYDSGCGWP SFTRPIDAKSVTEHDDFSYNMRRTEVRSHAAD SHLGHVFPDGPRDKGGLRYCINGASLKFIPLEQ MDAAGYGALKSKVK
Msr09	msrA2	P0A086	MTKEYATLAGGCFWCMVKPFTSYPGIKSV VSGYSGGHVDNPTYEQVCTNQTGHVEAVQ ITFDPEVTSFENILDIYFKTFDPTDDQGQFF DRGESYQPVIFYHDEHQKKAAEFKKQQLN EQGIFKKPVITPIKPYKNFYPAEDYHQDYYK KNPVHYYQYQRGSGRKAFIESHWGNQNA
Msr10	msrA msrB hybrid	Q5JHZ2	MGGIKIEPKTAIFAGGCFWCMEEAFERLPGVIEA ISGYTGGWVENPTYELVSTGETGHREAVKVIYD PSKISYERLLDVFWRNIDPTDPGGQFADRGEQYK TAIFYLDEEQRELAEESKRRLELSGIFDEPIATEIL PAKEFYPAEDYHQGYYFRFEANYKGYKLYSGRL GFIKSVWEKNRHFRLFPEREGYWLGYVKPSDAE LKRQLTPLQYRVTQLGDTEEPFHNEYWNNHEEG IYVDVVSGEPLFSLLDKYDSGTGWPSFTKPLEEW AVVEAGECEGFLCGREVRSRFAGSHLGHVFDEPT PTGKRYCINSAALRFIPRDELRRYGYTAYEGIFK
Msr11	msrA	A0A1B1B0V5	MTVGTERAVLAGGCFWGMQDLIRKQPGVVSTR VGYSGGDTPNATYRNHGDHAEAIEILFDPSATNY RDILEFFFQIHDPTTKDRQGNDLGRSYRSAIYYTD EEQHRVALDTIADVEASGLWPGKVVTEVEPVGD FWEAEPEHQDYLKRYPNGYTCHFPRPNWKLPRR TSSSTN
Msr13	msrA msrB hybrid	P0A3R0	MAEIYLAGGCFWGLEEYFSRISGVLETSVGYANG QVETTNYQLLKETDHAETVQVIYDEKEVSLREILL YYFRVIDPLSINQQGNDRGRQYRTGIYYQDEADLP AIYTVVQEQERMLGRKIAVEVEQLRHYILAEDYH QDYLRKNPSGYCHIDVTDADKPLIDAANYEKPSQ EVLKASLSEESYRVTQEAATEAPFTNAYDQTFEEG IYVDITTGEPLFFAKDKFASGCGWPSFSRPISKELIH YYKDLSHGMERIEVRSRSGSAHLGHVFTDGPRELG GLRYCINSASLRFVAKDEMEKAGYGYLLPYLNK
Msr15	msrA msrB hybrid	M2BFA6	MKNKNILFHLSFLMMIVLFLSCTKAQAEQKLPIG GKGMIKEIYLAGGCFWGVEGYFRQIPGVKETDTG YANGKNDSANYKGLHQSDHAETVKIVYDSSVVSL QELLAHYFRIIDPTSLNKQGNDAGRQYRTGIYYVD

			DSMIKEINSFVKFMQKKYSRPIVVEVEKLKHFILAE DYHQDYLQKNPGGYCHIDLTLALKPLYDESKFKVP SKEELKKSLKPIQFSVTQEKATERPFTSEYDKFDDE GIYVDITTGKPLFSSLNKYDAGCGWPSFTKAITTQA LQYLEDKSLGMNRTEVISKTGGAHLGHVFDDGPAD AGGLRYCINGAALRFIPYDKMEKEGYGDYLPYVK PTGTF
Msr16	msrA	B5Y2Y3	MSLFDKTHLVAQADALPGRNTPMPVATLHAVN GHSMTNVPAGMEVALFAMGCFWGVERLFWQLP GVYSTAAGYTGGYTPNPTYREVCSGQTGHAEAV RVVYDPQVISYEQLLQVFWENHDPAQGMRQGND HGTQYRSAIYPLTPEQTAAAKASLARFQAAMNDA HDTRHITTEIATAKPFYYAEDDHQQYLYKNPHGY CGIGGIGVCLPPQ
Msr17	msrA	B4TT49	MSLFDKKHLVTQADALPGRNTPMPIATLHAVNE HSMTNVPAGMEIAYFAMGCFWGVERLFWQLPG VYSTAAGYAGGYTPNPTYREVCSGQTGHAEAVR IVYDPAVIRYEQLLQIFWENHDPTQGMQQGNDH GTQYRSAIYPLTPEQSAAAHASRERFQSAMAAAG NHRPITTEITHATPFYYAEDEHQQYLHKNPYGYC GIGGIGVCLPPDA
Msr18	msrA	E9CMS8	MVPFFNKPLTADGGNVLPGRTTPMPVTTLNVVT GHSMTQVPSGMEVAIFTMGCFWGVERLFWQQP GVYSTAAGYSGGYTPNPTYSEVCSSQTGHAEVV RVVFDPKIISYKQLLQMFWENHDPTQGMRQGGD IGTQYRSAIFTLNQEQHTEAERSQQRFQQAMEAA GDKRIITTDVVPALPFYYAEDEHQQYLYKNPQGY CGLGGIGICLPPQG
Msr19	msrA	B2HLR5	MTSPQKAILAGGCFWGMQDLIRKQPGVIATRVGY SGGDVANATYRNHGTHAEAVEIIFDPQATDYRTLL EFFFQIHDPTTPNRQGNDRGTSYRSAIYYLDDEQKR VALDTIADVEASGLWPGKVVTEVSPAGDFWEAEPE HQDYLQRYPSGYTCHFIRPGWKLPRRATAGQ
Msr21	msrB	A0A1Z3MMZ9	MKFEKSQAAVDRLTPEQRRITQDSGTERPF TGEYNDNKEPGIYVDIVSGEPLFASTDKFDS GTGWPSFTKPIVSANVNEVRDSAHGMVRT EVRSVHADSHLGHVFPDGPSDRGGLRYCIN SASLRFIPRDEMESEGYGEYLDQVEEA

2.2 Purification of *S. cerevisiae* MsrA02 from CFE

1.6 g of MsrA02 CFE (Almac) was resuspended in Buffer A (20 mM Tris base pH 8.0, 100 mM NaCl, 20 mM imidazole and 10% *v/v* glycerol and 1 mM DTT) and was centrifuged at 4000 rpm for 20 minutes. A 5 mL His-Trap™ Fast Flow nickel affinity column was washed with water (7 CV) and equilibrated with Buffer A (7 CV). The filtered supernatant was loaded onto the nickel affinity column and the column was flushed with Buffer A (1.6 CV). The protein was eluted with Buffer B (20 mM Tris base pH 8.0, 100 mM NaCl, 200 mM imidazole and 10% *v/v* glycerol), the fractions of the purification were analysed by SDS-Page (expected molecular weight including the 6xHis tag of 23.3 kDa). The fractions containing the protein of interest were pooled and concentrated, and buffer exchanged in Buffer C (20 mM Tris base pH 8.0, 100 mM NaCl and 10% *v/v* glycerol) by centrifugation on

ultrafiltration unit (10 KDa MWCO, Cytiva, Sweden). When the volume reached approximately 1.5 mL, it was mixed with an equal amount of Buffer D (20 mM Tris base pH 8.0, 100 mM NaCl and 30% *v/v* glycerol), divided into 100 µL aliquots, snap frozen and stored at -80 °C. The final concentration was assessed through Bradford method.

2.3 General procedure for the kinetic resolution of substrates 1a-ab using MsrA02

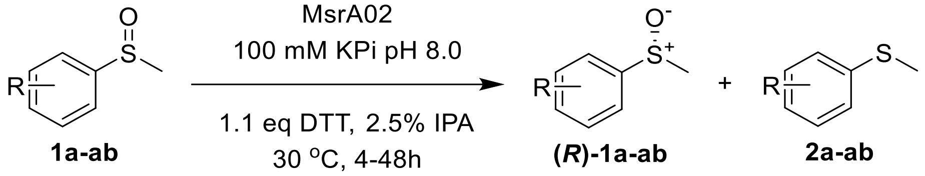

315 µL of a 1.28 M stock solution in IPA of the relevant racemic substrate **1a-ab** (32 mM final concentration), was added to 12.3 mL 100 mM KPi buffer pH 8.0 containing DTT (35.2 mM, 1.1 eq.) and MsrA02 to initiate the reaction. The reaction was monitored by taking 10 µL aliquots, which were extracted with EtOAc (3x20 µL) and injected into the appropriate HPLC chiral column. Upon completion, the reaction was extracted with EtOAc (5x5 mL) by centrifugation (5300 rpm, 5 min, 4 °C). The organic phase was dried over $MgSO_4$ and evaporated under reduced pressure and the resulting crude was purified by flash column chromatography using a mixture of EtOAc and hexane to afford the resulting enantiopure (*R*)-sulfoxide. Compounds were purified on neutral alumina (Al_3O_2) and the final enantiomeric excess was determined using Chiralpak IC or IG chiral columns. The case of substrate **1r** is highlighted in the scheme below.

Scheme S1. Schematic representation of the MsrA02 biocatalysed reduction of substrate **1r**

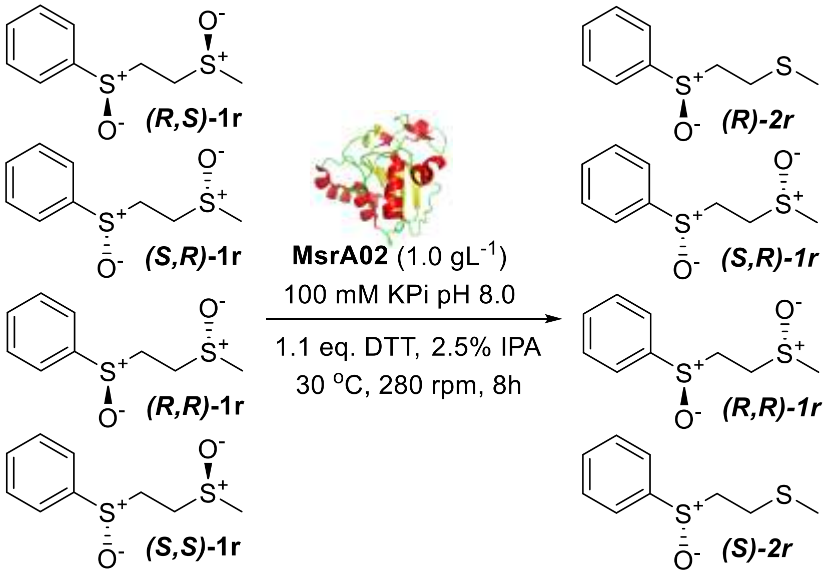

2.4 Preparation of C25S and C68S MsrA02

2.4.1 General considerations

The plasmid encoding WT MsrA02 gene (pET28a-Msr-XhoI-NdeI) was obtained by Almac. PCR kit QuikChange II was purchased by Agilent Technologies and the primers were purchased from Merk Life Science.

Desired point mutation	Nucleotide change	Forward primer (5'-3')	Reverse primer (3'-5')
C25S	TGC→T**C**C	CACCCTGGCGTGCGG TT**C**CTTTTGGGGCSC CGAGCACATGTACCG	GACCGCACGCCAA**G**G AAAACCCCGTGGCTC GTGTAC
C68S	TGC→T**C**C	AGCTATAAGCGT GTTT**C**CGGTGGC GACACC	TCGATATTCGCA CAAA**G**GCCACCG CTGTGG

2.4.2 Site directed mutagenesis

Site directed mutagenesis was performed by PCR according to Quick Change II manufacturer's instructions using pET28a-Msr-XhoI-NdeI as template. After PCR, the template of the reaction was digested with DpnI (1 hour 1 uL, Thermofisher). On completion, 2 uL of the reaction mixture were used to transform 50 uL of Top10 competent *E. coli* cells. Single colonies were selected and incubated overnight in 15 mL of LB media with 50 $mgmL^{-1}$ kanamycin. The DNA was extracted and purified with the QIAprep® Spin Minikit (QIAGEN). The resulting DNA was analysed by agarose gel after incubation with XhoI, BglI and BssSI digestive enzymes. The mutated sequence was confirmed by sequencing (Genewiz).

2.4.3 Gene expression and purification

The purified MsrA02_C25S or C68S plasmids were transformed into T7 competent cells and the resulting cell mixture was plated on LB agar (50 μgmL^{-1} kanamycin) and incubated overnight at 35 °C. A single colony of transformed cells was used to inoculate 100 mL preculture in LB media with kanamycin (50 μgmL^{-1}) and incubated overnight at 37 °C and 220 rpm. The overnight precultures were used to inoculate 2x1L cultures in LB media with kanamycin (50 μgmL^{-1}). When the OD_{600} reached 0.6-0.8 the expression of the protein was induced with 0.5 mM isopropyl-β-D-thiogalactopyranoside (IPTG) and incubated overnight. Cells were harvested by centrifugation (18,000 rpm, 45 min, 4 °C), resuspended in cell lysis buffer (Buffer A, pepstatin, protease cocktail, lysozyme, DNAase, 1 mM DTT) and lysed using a IXT4A (Constant System LTD) cell disruptor (25 psi, 4 °C). After centrifugation (20,000 rpm, 1h, 4 °C), the CFE was loaded onto a His-Trap™ Fast Flow nickel affinity column and purified following the procedure described in Section 2.2. The final concentration was assessed through Bradford method.

2.5 Activity assay of C25S and C68S MsrA02

Reactions were run in duplicates in a final volume of 1.0 mL in microcentrifuge tubes. C25S or C68S MsrA02 was used directly from the previously prepared stock and was diluted to three different concentrations (10, 100 and 400 mM) in 100 mM KPi buffer (pH8.0) containing 8.8 mM DTT. 25 µL of a 320 mM stock solution of **1a** in IPA were added to the reactions and the microcentrifuge tubes were shaken at 30 °C and 280 rpm for 18 h. The reactions were extracted using 3x250 µL EtOAc and the enantiomeric excess was determined using a Chiralpak IG chiral column.

2.6 Activity assay of C23S, C44S and C176S MsrA02

Reactions were run in duplicates in a final volume of 500 µL. The CFE of the mutants C23S, C44S and C176S of MsrA02 were provided in 96-well plates containing 10mg of lyophilised powder each. Each mutant was redissolved in 100 mM KPi buffer (pH 8.0) to 1.0, 10 and 40 mg/mL final concentrations. Then, 12.5 µL 352 mM DTT in buffer and 12.5 µL of a 320 mM stock solution of **1a** in IPA was added to each well to initiate the reaction. The plate was incubated for 18 h at 30 °C and 1000 rpm. 500 µL of CH_3CN was added to each well to quench the reaction and the plates were centrifuged for 20 min at 4000 rpm and 4 °C to get rid of cell debris. To determine the activity of the mutants, 5 µL aliquots were injected into a Daicel Chiralpak IC column and the enantiomeric excess was determined.

2.7 Identification of other Msrs for the kinetic resolution of bulkier sulfoxides

The CFE of 13 Msrs and EVC was provided by Almac in 96-well plates as 10 mg of lyophilised powder. Each well was resuspended in 950 µL 100 mM KPi buffer (pH 8.0) and split into two wells of a fresh 96-well plate (475 µL each). Then, 12.5 µL 352 mM DTT in buffer and 12.5 µL of a 320 mM sulfoxide solution in IPA or CH_3CN were added to each well to initiate the reaction. The plate was shaken for 18 h at 30 °C and 1000 rpm. 500 µL of CH_3CN were added to the wells to quench the reaction and the plates were centrifuged for 20 min at 4000 rpm and 4 °C to get rid of cell debris. To determine the final concentration of the remaining sulfoxide, 1 µL aliquots were injected into a UPLC Agilent Eclipse Plus C18 column and analysed with the following method: 0.5 mL/min, 25 °C; *T0*, water:CH_3CN 90:10; *T4min*, water:CH_3CN 40:60, hold for 1.5 min; *T6min*, water:CH_3CN 90:10, hold for 1 minute. Total time 7 minutes.

2.8 Screening of MsrA31-49 mutants and new WT MsrAs

In this new panel of enzymes, the MsrA mutants were generated from the *in silico* studies while the new WT were selected from the literature.

Table S1. Screening of mutant and new WT enzymes for the reduction of bulky substrates.

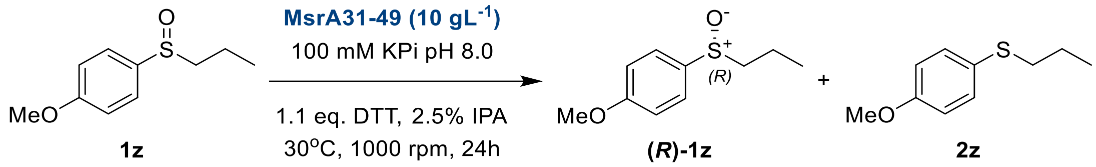

Entry	Mutant MsrA code	WT MsrA	WT residue	Mutant residue	Position	ee[a]%
1	MsrA31	02	Cys68	Thr	α2-helix	34
2	MsrA32	02	Trp27	Phe	Pocket	18
3	MsrA33	02	Phe26	Tyr	Pocket	46
4	MsrA34	02	Gly28	Cys	α1-helix	<1
5	MsrA35	02	Leu167	Phe	C-terminal loop	13
6	MsrA36	02	Tyr174	Lys	C-terminal loop	<1
7	MsrA37	02	Tyr174	Leu	C-terminal loop	<1
8	MsrA38	02	H179	Arg	C-terminal loop	<1
9	MsrA39	02	Gly28 Tyr174	Cys Lys	Pocket and C-terminal loop	<1
10	MsrA40	10	Ser52	Thr	α2-helix	<1
11	MsrA41	10	Phe18	Tyr	Pocket	<1
12	MsrA42	10	Tyr169	Lys	C-terminal loop	<1
**Entry**	**New WT Msr**				**Organism**	
13	MsrA43				Unspecified bacterium	<1
14	MsrA44				*S. piezotolerans*	<1
15	MsrA45				*B. solimangrovi*	<1
			**Table S1. continued**			
16	MsrA46				*Archaeon*	<1
17	MsrA47				*C. Aenigmarchaeota archaeon*	6
18	MsrA49				*Lentisphaerae* bacterium	<1

[a] Determined by chiral HPLC using a Chiracel IC column.

2.9 Optimisation of the reaction conditions using MsrA33

In the table below are shown the results of the optimisation experiments for the reduction of **1z** using MsrA33.

Table S2. Optimisation of conditions of the reduction of **1z** with MsrA33.

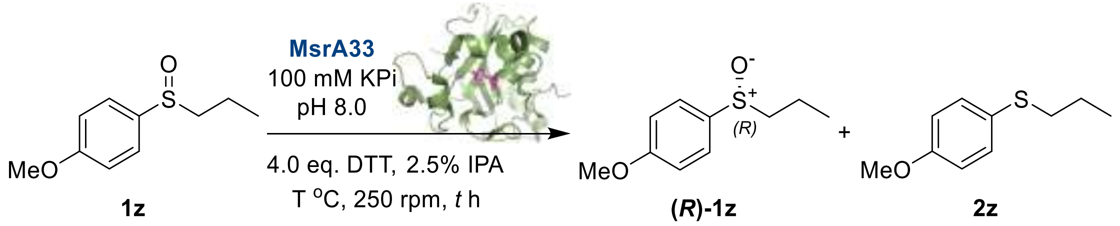

Entry	MsrA33 conc. / gL^{-1}	1z conc. / mM	T / °C	Time / h	(*R*)-1z ee[a,b] %
1	1.0	8.0	30	24	4
2	1.0	32	30	24	<1
3	10	32	30	24	6
4	10	8.0	30	24	40
5	10	8.0	30	48	66
6	10	8.0	30	72	66
7	10	4.0	30	24	50
8	10	4.0	30	48	70
9	10	2.0	30	24	54
10	10	2.0	30	48	50
11	10	1.0	30	24	28
12	10	1.0	30	48	28
13	20	8.0	30	24	78
14	20	8.0	30	48	82
15	40	8.0	30	24	92
16	**40**	**8.0**	**30**	**48**	**>99 (42)**[c]
17	20	8.0	37	24	6

[a] Determined by chiral HPLC using a Chiracel IC column. [b] Reactions run in duplicates. [c] HPLC yield is reported. Calculated using an Agilent Eclipse Plus C18 column and methyl *p*-tolyl sulfoxide as internal standard.

2.10 General procedure for the kinetic resolution of sulfoxides using MsrA33

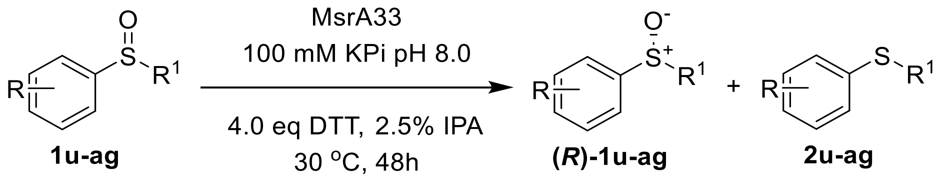

25 µL of a 320 mM stock solution in IPA of the relevant racemic substrate **1u-ag** (32 mM final concentration), was added to 975 µL 100 mM KPi buffer pH 8.0 containing DTT (16 mM, 4.0 eq.) and MsrA33 (40 gL^{-1}) to initiate the reaction. The reaction was shaken at 30 °C for 48 h. Upon completion, a 30 µL aliquot was extracted with EtOAc (3x50 µL), centrifuged (12500 rpm, 5 min) and the collected organic layers were analysed by normal phase HPLC using Chiralpak IC, IG or ID chiral columns to determine the enantiomeric excess. The remaining reaction mixture was quenched with 500 µL of CH_3CN and 25 uL of compound **1b** was added as internal standard. The conversion was calculated by reversed phase HPLC using an Agilent Eclipse Plus C18 column.

2.11 Additional screening of MsrA enzymes on bulky substrates

The sulfoxides **1m**, **1n**, **1x**, **1z**, **1aa** and **1ab**, which were poorly converted by MsrA02, were screened with addiotnal MsrA enzymes from Table 1. In all cases poor conversions were observed.

Table S3. Further screening of MsrA enzymes with bulkier sulfoxides.

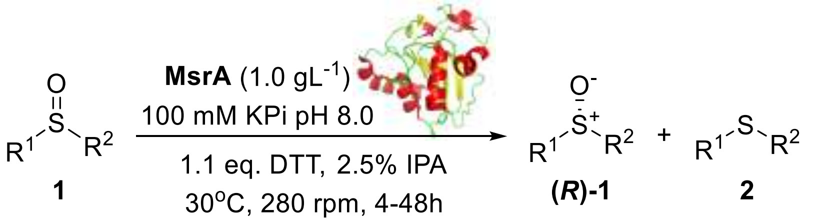

Substrate	R[1]	R[2]	Msr	ee[a] %
**1m**	2-Naph	Me	01	9
			08	9
			16	32
**1n**	1-Naph	Me	01	<1
			08	<1
			10	<1
			16	10
**1x**	2-PyCH$_2$	Et	08	8
			10	<1
			16	14
**1z**	4-MeOPh	*n*Pr	08	<1
			10	<1
			16	4
**1aa**	Ph	Vinyl	01	n.d.[c]
			08	
			10	
			16	
**1ab**	Ph	Allyl	08	4
			10	<1
			16	18

[a]Determined by chiral HPLC using Chiralpak column IG or IC. [b]8 mM substrate. [c]n.d. = not determined.

2.12 Mechanism for the reduction of MetSO by MsrA02

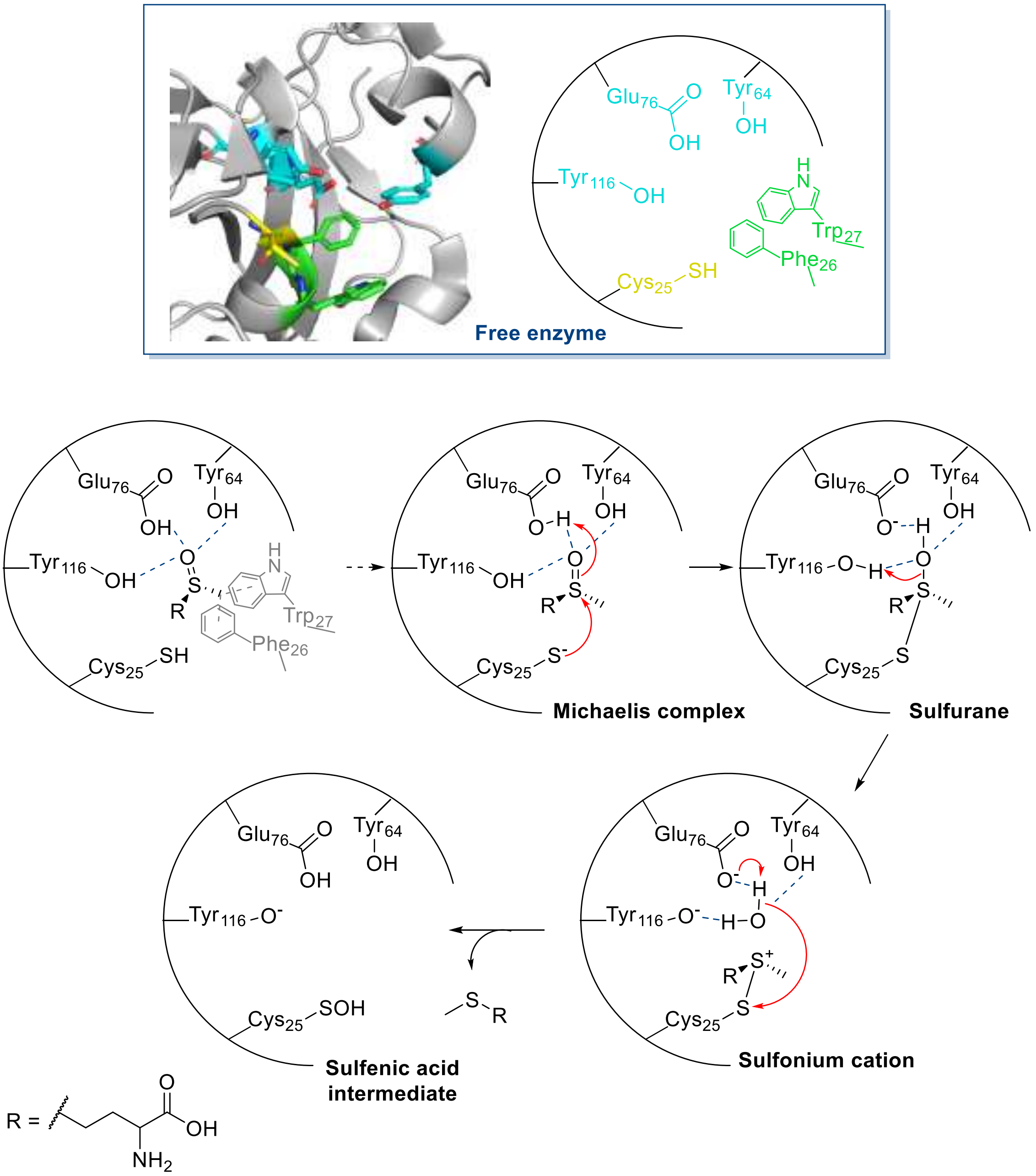

Figure S1. Mechanism of MetSO reduction and sulfenic acid formation.

3 Computational Methods

3.1 Modelling

The crystal structure of MsrA02 from *Streptomyces cerevisiae* (pdb code 3PIL)[1] was used as the initial structure. The acetate molecule was deleted, and hydrogens were added with Molprobity.[2] For the variants, single point mutations were introduced using a rotamer library. Afterwards, the structures were geometry optimized and subjected to molecular dynamics simulations. GROMACS[3] cluster analysis was conducted to select the most representative structures from the simulations

The substrates **1a** and **1q** were optimized with the exchange-correlation functional B3LYP,[4] the 6-31g(d) basis set and Polarizable Continuum Model (PCM).[5] The point charges were calculated resorting to the RESP methodology, from a single point calculation using Hartree-Fock with the 6-31g(d) basis set.

3.2 Molecular Docking

Molecular docking was performed using Autodock4.2 with the Lamarckian genetic algorithm (LGA)[6] using a grid around the sulphur atom of the nucleophilic cysteine. A total of 1000 LGA runs were carried out per system. The population was 300, the GA elitism=1, the maximum number of generations was 27000 and the maximum number of energy evaluations was 2500000. The top ranked structure corresponds to lowest binding energy structure of the most populated cluster with the lowest mean binding energy.

3.3 Molecular Dynamics Simulations

Molecular dynamics (MD) simulations were performed with GROMACS[3] with the amber parm99SB[7] force field. One initial energy minimization was performed, followed by two equilibration steps to slowly heat the system from 0 to 300 K (in canonical and isothermal-isobaric ensembles, respectively). Temperature and pressure coupling were 300 K and 1 bar, respectively and periodic boundary conditions (PBC) were used. Production runs were performed in an isothermal-isobaric ensemble. For each enzyme 3 replicas of the production simulations (100 ns) were carried out at 300 K The time step was set to 2 fs and LINCS[8] constraints were applied to all bonds involving hydrogen atoms. The particle mesh Ewald (PME) method[9] was used to calculate electrostatic interactions.

4 NMR studies

4.1 Production of N^{15} and C^{13} labelled MsrA02 and MsrA02_C25S

The purified MsrA02 and MsrA02_C25S plasmids were transformed into T7 competent cells and the resulting cell mixture was plated on LB agar (50 μgmL^{-1} kanamycin) and incubated overnight at 35 ^{o}C. A single colony of transformed cells was used to inoculate 1.0 mL SOC media with kanamycin (50 μgmL^{-1}) and incubated at 30 ^{o}C and 220 rpm until cloudy. Then 150 µL of the SOC media was used to inoculate 50 mL M9 media (100 mM NaH_2PO_4, 100 mM KH_2PO_4, 100 mM NaCl, 1 mM $MgSO_4$, 0.1 mM $CaCl_2$, 1 mM Thiamine-HCl, 22 mM ^{13}C glucose, 19 mM $^{15}NH_4Cl$) with kanamycin (50 μgmL^{-1}) and incubated overnight at 37 ^{o}C. The overnight M9 preculture was used to inoculate 500 mL of M9 media and incubated at 30 ^{o}C. When OD_{600} reached 0.6-0.8 the expression of the protein was induced with 0.5 mM isopropyl-β-D-thiogalactopyranoside (IPTG) and incubated overnight. Cells were harvested by centrifugation (18,000 rpm, 45 min, 4 ^{o}C), resuspended in cell lysis buffer (Buffer A, pepstatin, protease cocktail, lysozyme, DNAase, 1 mM DTT) and lysed using a IXT4A (Constant System LTD) cell disruptor. After centrifugation (20,000 rpm, 1h, 4 ^{o}C), the CFE was loaded onto a His-Trap™ Fast Flow nickel affinity column and purified following the procedure described in Section 2.2. The final concentration was assessed through Bradford method.

4.2 NMR spectroscopy

Spectra were acquired on a Bruker Avance III spectrometer at a proton frequency of 600MHz using a QCIP cryoprobe at 25 C. NMR spectra were processed using NMRPipe[10] and analyzed using NMRView.[11] Backbone ^{1}HN, ^{15}N, and ^{13}C, assignments were obtained from triple resonance HNCA, HN(CO)CA, CBCA(CO)NH, HNCACB, and HNCO spectra collected on ^{15}N- and ^{13}C- labelled MsrA02 enzyme. Backbone resonances are deposited on the BMRB (51733).

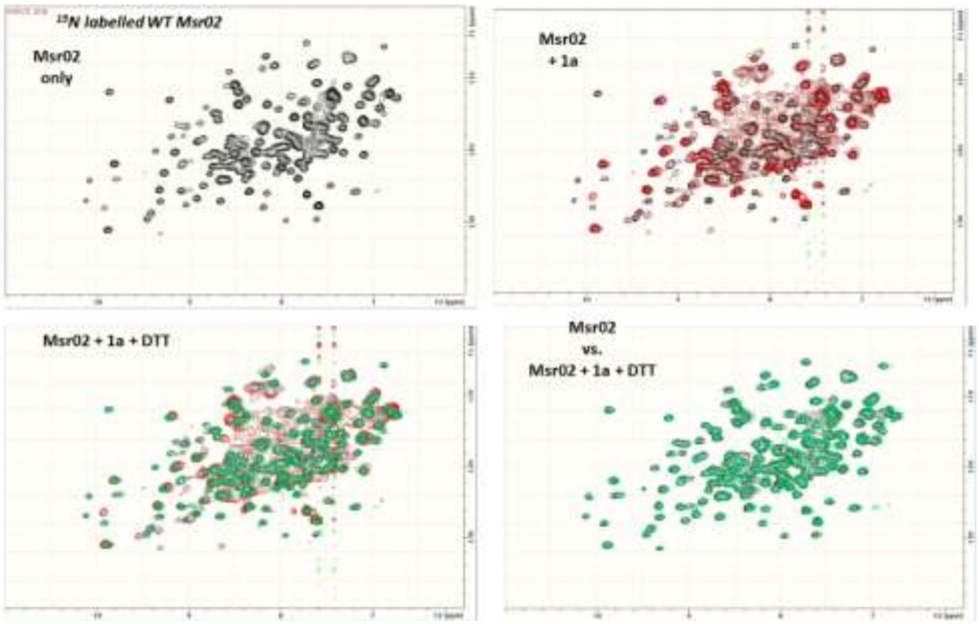

Figure S2. NMR experiments with ^{15}N labelled MsrA02 and **1a**

5 Chemistry

5.1 General procedure for the synthesis of racemic sulfoxides

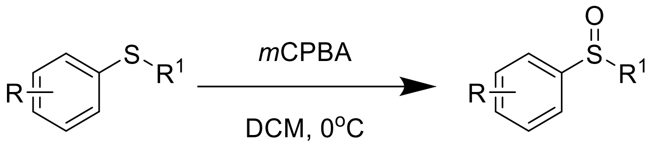

2a-ag **1a-ag**

Sulfides **2a-l**, **2o**, and **2q** were commercially available. Sulfides **2m-n**, **2r-z** and **2ab-ag** were synthesised according to the literature.[12–15] Racemic sulfoxides were synthesised as follows: the appropriate sulfide (2.0 mmol, 1.0 eq.) was dissolved in DCM (10 mL) and stirred at 0 °C. *m*CPBA (2.2 mmol, 1.1 eq) in DCM (10 mL) was added dropwise using a dropping funnel and the reaction was monitored by TLC for 1-24h until completion. The reaction was dried under reduced pressure and purified by flash column chromatography using an appropriate eluent mixture of EtOAc and hexane to afford the resulting sulfoxide.

6 Characterisation of compounds

6.1 Characterisation data of the substrate sulfoxides 1a-ag and sulfide 2r

6.1.1 1-Methyl-4-(methylsulfinyl)benzene (1a)

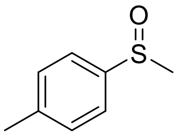

Commercially available.

6.1.2 (Methylsulfinyl)benzene (1b)

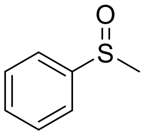

Commercially available.

6.1.3 1-Fluoro-4-(methylsulfinyl)benzene (1c)[16,17]

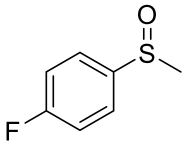

Colourless oil (102 mg, 92% yield). ^1H NMR (400 MHz, Chloroform-*d*) δ 7.70 – 7.60 (m, 2H), 7.25 – 7.17 (m, 2H), 2.71 (s, 3H). ^{13}C NMR (101 MHz, $CDCl_3$) δ 165.7, 163.2, 141.3, 126.0, 125.9, 117.0, 116.7, 44.3, 44.3. ^{19}F NMR (376 MHz, $CDCl_3$) δ -108.62.

6.1.4 1-Bromo-4-(methylsulfinyl)benzene (1d)[18]

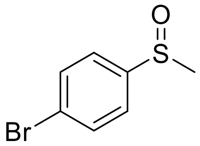

White crystalline solid (193 mg, 90% yield). ^1H NMR (400 MHz, Chloroform-*d*) δ 7.68 (d, J = 8.6 Hz, 2H), 7.53 (d, J = 8.6 Hz, 2H), 2.72 (s, 3H). ^{13}C NMR (101 MHz, $CDCl_3$) δ 145.1, 132.8, 125.6, 125.3, 44.2.

6.1.5 1-Chloro-4-(methylsulfinyl)benzene (1e)[18]

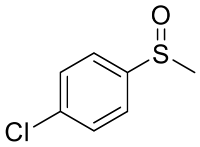

White solid (359 mg, 99% yield). ^{1}H NMR (400 MHz, Chloroform-*d*) δ 7.62 – 7.57 (m, 2H), 7.54 – 7.49 (m, 2H), 2.72 (d, *J* = 0.6 Hz, 3H). ^{13}C NMR (101 MHz, $CDCl_3$) δ 144.1, 137.1, 129.5, 124.8, 43.9.

6.1.6 1-Chloro-3-(methylsulfinyl)benzene (1f)[19]

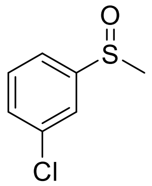

Pale yellow oil (362 mg, 99% yield). ^{1}H NMR (400 MHz, Chloroform-*d*) δ 7.68 – 7.65 (m, 1H), 7.52 – 7.45 (m, 3H), 2.74 (s, 3H). ^{13}C NMR (101 MHz, $CDCl_3$) δ 148.1, 135.9, 131.3, 130.7, 123.8, 121.8, 44.2.

6.1.7 1-Chloro-2-(methylsulfinyl)benzene (1g)[16]

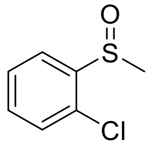

Pale yellow oil (125 mg, 57% yield). ^{1}H NMR (500 MHz, $CDCl_3$) δ 7.97 (dd, *J* = 7.8, 1.7 Hz, 1H), 7.54 (td, *J* = 7.5, 1.3 Hz, 1H), 7.45 (td, *J* = 7.6, 1.6 Hz, 1H), 7.40 (dd, *J* = 8.0, 1.3 Hz, 1H), 2.83 (s, 3H). ^{13}C NMR (126 MHz, $CDCl_3$) δ 143.8, 132.1, 130.0, 129.9, 128.3, 125.5, 41.8.

6.1.8 (4-(Methylsulfinyl)phenyl)ethan-1-one (1h)[18,20]

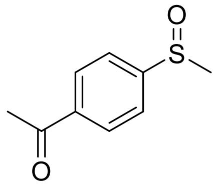

White amorphous solid (434 mg, 87% yield). ^{1}H NMR (400 MHz, Chloroform-*d*) δ 8.09 – 7.98 (m, 1H), 7.73 – 7.64 (m, 1H), 2.70 (s, 1H), 2.58 (s, 1H). ^{13}C NMR (101 MHz, $CDCl_3$) δ 197.0, 150.9, 139.0, 129.1, 123.7, 43.8, 26.8.

6.1.9 Methoxy-4-(methylsulfinyl)benzene (1i)[18]

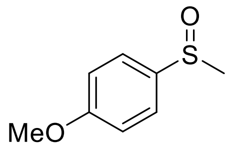

Off white solid (241 mg, 73% yield). ^{1}H NMR (400 MHz, Chloroform-*d*) δ 7.63 – 7.57 (m, 2H), 7.06 – 6.99 (m, 2H), 3.85 (s, 3H), 2.71 (s, 3H). ^{13}C NMR (101 MHz, $CDCl_3$) δ 162.2, 136.6, 125.7, 115.0, 55.7, 44.1.

6.1.10 Methoxy-3-(methylsulfinyl)benzene (1j)[20]

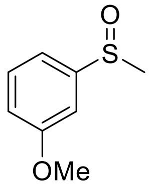

Colourless oil (386 mg, 77% yield). ^{1}H NMR (400 MHz, Chloroform-*d*) δ 7.44 – 7.38 (m, 1H), 7.27 – 7.24 (m, 2H), 7.13 (ddd, *J* = 7.7, 1.6, 0.9 Hz, 1H), 7.02 (ddd, *J* = 8.3, 2.6, 0.9 Hz, 1H), 3.87 (s, 3H), 2.73 (s, 3H). ^{13}C NMR (101 MHz, $CDCl_3$) δ 160.7, 147.3, 130.5, 117.6, 115.7, 108.1, 55.8, 44.2.

6.1.11 1-Methyl-3-(methylsulfinyl)benzene (1k)[18]

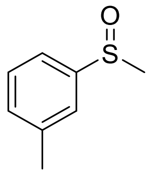

Colourless oil (312 mg, 88% yield). ^{1}H NMR (400 MHz, Chloroform-*d*) δ 7.49 (dt, *J* = 1.5, 0.8 Hz, 1H), 7.43 – 7.38 (m, 2H), 7.33 – 7.27 (m, 1H), 2.72 (s, 3H), 2.43 (t, *J* = 0.8 Hz, 3H). ^{13}C NMR (101 MHz, $CDCl_3$) δ 145.4, 139.5, 131.7, 129.0, 123.6, 120.5, 43.8, 21.3.

6.1.12 2,4-Dimethyl-1-(methylsulfinyl)benzene (1l)[21]

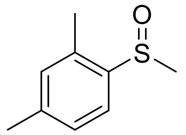

Colourless oil (275 mg, 83% yield). ^{1}H NMR (500 MHz, $CDCl_3$) δ 7.83 (d, *J* = 8.0 Hz, 1H), 7.25 (dd, *J* = 8.1, 1.8 Hz, 1H), 7.01 (d, *J* = 1.8 Hz, 1H), 2.66 (s, 3H), 2.36 (s, 3H), 2.34 (s, 3H). ^{13}C NMR (126 MHz, $CDCl_3$) δ 141.2, 141.0, 134.0, 131.6, 128.3, 123.3, 42.4, 21.3, 18.1.

6.1.13 (Methylsulfinyl)naphthalene (1m)[18]

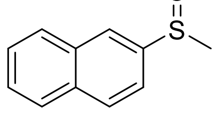

White crystals (170 mg, 57% yield). ^{1}H NMR (400 MHz, $CDCl_3$) δ 8.22 (d, *J* = 0.6 Hz, 1H), 7.99 (d, *J* = 0.8 Hz, 1H), 7.97 – 7.89 (m, 2H), 7.67 – 7.55 (m, 3H), 2.80 (s, 3H). ^{13}C NMR (101 MHz, $CDCl_3$) δ 129.8, 128.7, 128.2, 128.0, 127.5, 124.2, 119.6, 44.0. 3 quat carbons

6.1.14 (Methylsulfinyl)naphthalene (1n)[22]

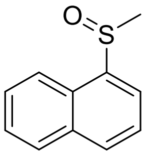

White amorphous solid (106 mg, 85% yield). ^{1}H NMR (400 MHz, $CDCl_3$) δ 8.17 (dd, *J* = 7.3, 1.2 Hz, 1H), 7.99 – 7.86 (m, 3H), 7.65 (dd, *J* = 8.2, 7.3 Hz, 1H), 7.61 – 7.51 (m, 2H), 2.82 (s, 3H). ^{13}C NMR (101 MHz, $CDCl_3$) δ 141.6, 133.5, 131.3, 129.2, 128.6, 127.3, 126.8, 125.8, 122.2, 121.4, 43.0.

6.1.15 ((Methylsulfinyl)methyl)benzene (1o)[18]

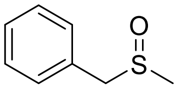

Off white solid (170 mg, 57% yield). ^{1}H NMR (400 MHz, $CDCl_3$) δ 7.43 – 7.33 (m, 3H), 7.29 (dd, *J* = 7.6, 1.8 Hz, 2H), 4.07 (d, *J* = 12.8 Hz, 1H), 3.93 (d, *J* = 12.8 Hz, 1H), 2.46 (s, 3H). ^{13}C NMR (101 MHz, $CDCl_3$) δ 130.2, 129.8, 129.1, 128.6, 60.5, 37.4 ppm. MS (APCI): m/z = 155.1 $[M+H]^+$.

6.1.16 (Methylsulfinyl)dodecane (1p)

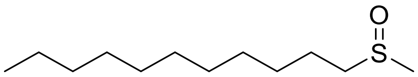

Commercially available.

6.1.17 (Methylsulfinyl)butan-2-one (1q)

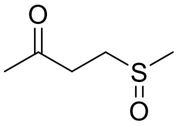

Pale yellow oil (312 mg, 99% yield). ν_{max}/cm^{-1}: 2996, 2913, 1709. ^{1}H NMR (500 MHz, $CDCl_3$) δ 3.08 – 3.00 (m, 1H), 2.97 (td, *J* = 7.2, 2.6 Hz, 2H), 2.85 – 2.76 (m, 1H), 2.57 (s, 2H), 2.23 (d, *J* = 0.9 Hz, 3H). ^{13}C NMR (126 MHz, $CDCl_3$) δ 205.3, 47.7, 39.1, 35.6, 30.0. HRMS (ESI) m/z calcd. for $C_5H_{11}O_2{}^{32}S^+$ $[M+H]^+$ 135.0474; found 135.0475.

6.1.18 ((2-(Methylsulfinyl)ethyl)sulfinyl)benzene (1r)

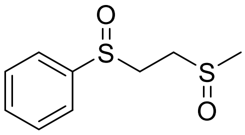

White crystalline solid (166 mg, 85% yield). ν_{max}/cm^{-1}: 2911, 1421, 1019. ^{1}H NMR (500 MHz, $CDCl_3$) δ 7.64 – 7.48 (m, 10H), 3.43 – 3.32 (m, 2H), 3.23 (ddd, *J* = 13.1, 10.4, 5.3 Hz, 1H), 3.10 – 2.97 (m, 3H), 2.97 – 2.88 (m, 1H), 2.66 (ddd, *J* = 13.1, 10.4, 4.9 Hz, 1H), 2.59 (s, 3H), 2.56 (s, 3H). ^{13}C NMR

(126 MHz, $CDCl_3$) δ 142.6, 142.4, 131.6, 131.6, 129.6, 124.1, 124.0, 48.8, 48.5, 45.5, 45.4, 39.0, 39.0. HRMS (ESI) m/z calcd. for $C_9H_{13}O_2{}^{32}S^{2+}$ $[M+H]^+$ 217.0352; found 217.0348.

6.1.19 (Ethylsulfinyl)benzene (1s)[23]

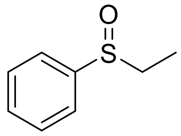

Yellow oil (91 mg, 82% yield). ^{1}H NMR (400 MHz, $CDCl_3$) δ 7.66 – 7.57 (m, 2H), 7.57 – 7.44 (m, 2H), 2.91 (dq, *J* = 13.3, 7.4 Hz, 1H), 2.78 (dq, *J* = 13.3, 7.4 Hz, 1H), 1.20 (t, *J* = 7.4 Hz, 3H). ^{13}C NMR (101 MHz, $CDCl_3$) δ 143.4, 131.1, 129.3, 124.4, 50.4, 6.1.

6.1.20 (Ethylsulfinyl)-4-methylbenzene (1t)[14]

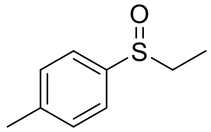

Colourless oil (71% yield). ^{1}H NMR (400 MHz, $CDCl_3$) δ 7.51 – 7.43 (m, 2H), 7.29 (d, *J* = 7.9 Hz, 2H), 2.84 (dq, *J* = 13.2, 7.4 Hz, 1H), 2.73 (dq, *J* = 13.2, 7.4 Hz, 1H), 2.39 (s, 3H), 1.16 (t, *J* = 7.4 Hz, 3H). ^{13}C NMR (101 MHz, $CDCl_3$) δ 141.4, 140.2, 129.9, 124.3, 50.4, 21.5, 6.1.

6.1.21 1-Bromo-4-(ethylsulfinyl)benzene (1u)[24]

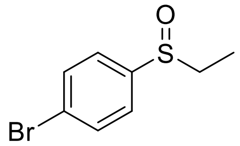

Colourless oil (275 mg, 92% yield). ^{1}H NMR (400 MHz, $CDCl_3$) δ 7.68 – 7.60 (m, 2H), 7.51 – 7.42 (m, 2H), 2.88 (dq, *J* = 13.3, 7.4 Hz, 1H), 2.72 (dq, *J* = 13.3, 7.4 Hz, 1H), 1.18 (t, *J* = 7.4 Hz, 3H). ^{13}C NMR (101 MHz, $CDCl_3$) δ 142.6, 132.5, 125.9, 125.5, 50.4, 5.9.

6.1.22 ((Ethylsulfinyl)methyl)benzene (1v)[14,19]

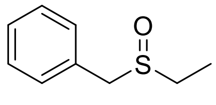

White solid (198 mg, 66% yield). ^{1}H NMR (500 MHz, $CDCl_3$) δ 7.40 – 7.32 (m, 3H), 7.31 – 7.27 (m, 2H), 4.02 (d, *J* = 12.9 Hz, 1H), 3.93 (d, *J* = 13.0 Hz, 1H), 2.72 – 2.49 (m, 2H), 1.33 (t, *J* = 7.5 Hz, 3H). ^{13}C NMR (126 MHz, $CDCl_3$) δ 130.2, 130.1, 129.1, 128.5, 57.9, 44.3, 6.7.

6.1.23 (2-(Ethylsulfinyl)ethyl)benzene (1w)

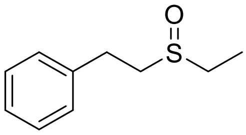

Pale yellow oil (258 mg, 86% yield). v_{max}/cm^{-1}: 2930, 1452, 1014. ^1H NMR (400 MHz, $CDCl_3$) δ 7.38 – 7.28 (m, 2H), 7.28 – 7.20 (m, 3H), 3.21 – 3.01 (m, 2H), 3.02 – 2.82 (m, 2H), 2.78 – 2.67 (m, 2H), 1.33 (tdd, *J* = 7.6, 1.7, 0.9 Hz, 3H). ^{13}C NMR (101 MHz, $CDCl_3$) δ 139.1, 128.9, 128.7, 126.9, 53.2, 45.9, 29.0, 6.8. HRMS (ESI) m/z calcd. for $C_{10}H_{15}O^{32}S^+$ $[M+H]^+$ 183.0838; found 183.0839.

6.1.24 2-((Ethylsulfinyl)methyl)pyridine (1x)

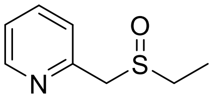

Colourless oil (242 mg, 81% yield). v_{max}/cm^{-1}: 2970, 2930, 1433, 1041. ^1H NMR (400 MHz, $CDCl_3$) δ 8.62 – 8.56 (m, 1H), 7.70 (td, *J* = 7.7, 1.8 Hz, 1H), 7.37 (d, *J* = 7.8 Hz, 1H), 7.30 – 7.22 (m, 1H), 4.17 (d, *J* = 12.8 Hz, 1H), 4.07 (d, *J* = 12.8 Hz, 1H), 2.78 (dq, *J* = 13.2, 7.6 Hz, 1H), 2.66 (dq, *J* = 13.2, 7.5 Hz, 1H), 1.34 (t, *J* = 7.5 Hz, 3H). ^{13}C NMR (101 MHz, $CDCl_3$) δ 151.0, 149.9, 137.01, 125.5, 123.2, 45.0, 6.8. HRMS (ESI) m/z calcd. for $C_8H_{12}ON^{32}S^+$ $[M+H]^+$ 170.0634; found 170.0631.

6.1.25 (Propylsulfinyl)benzene (1y)[25]

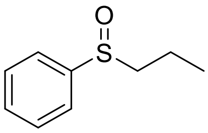

Yellow oil (366 mg, 99% yield). ^1H NMR (500 MHz, $CDCl_3$) δ 7.66 – 7.59 (m, 2H), 7.55 – 7.46 (m, 3H), 2.86 – 2.71 (m, 2H), 1.87 – 1.74 (m, 1H), 1.73 – 1.60 (m, 1H), 1.05 (t, *J* = 7.4 Hz, 3H). ^{13}C NMR (126 MHz, $CDCl_3$) δ 144.3, 131.3, 129.6, 124.6, 59.6, 16.3, 13.6.

6.1.26 1-Methoxy-4-(propylsulfinyl)benzene (1z)[26]

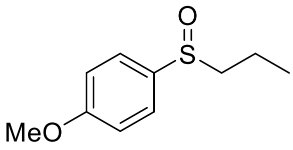

Yellow oil (288 mg, 95% yield). ^1H NMR (400 MHz, $CDCl_3$) δ 7.61 – 7.50 (m, 2H), 7.01 (dd, *J* = 8.7, 1.4 Hz, 2H), 3.84 (s, 1H), 2.87 – 2.63 (m, 2H), 1.79 – 1.57 (m, 2H), 1.03 (td, *J* = 7.5, 1.3 Hz, 3H). ^{13}C NMR (101 MHz, $CDCl_3$) δ 162.1, 135.0, 126.1, 114.9, 59.5, 55.6, 16.1, 13.4.

6.1.27 (Vinylsulfinyl)benzene (1aa)

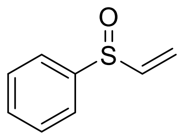

Commercially available

6.1.28 (Allylsulfinyl)benzene (1ab)[23]

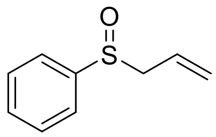

Yellow oil (58% yield). ^{1}H NMR (400 MHz, $CDCl_3$) δ 7.64 – 7.56 (m, 2H, *Ar*), 7.49 (m, 3H, *Ar*), 5.63 (m, 1H), 5.31 (dd, *J* = 10.2, 1.4 Hz, 1H), 5.17 (dd, *J* = 17.0, 1.3 Hz, 1H), 3.62 – 3.42 (m, 2H) ppm. ^{13}C NMR (101 MHz, $CDCl_3$) δ 142.9, 131.1, 129.1 (2C), 125.3, 124.3 (2C), 123.9, 60.9 ppm.

6.1.29 1-Methyl-4-(propylsulfinyl)benzene (1ac)[13]

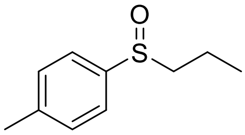

Colourless oil (284 mg, 85% yield). ^{1}H NMR (500 MHz, $CDCl_3$) δ 7.54 – 7.48 (m, 2H), 7.34 – 7.29 (m, 2H), 2.84 – 2.67 (m, 2H), 2.41 (s, 3H), 1.83 – 1.71 (m, 1H), 1.72 – 1.58 (m, 1H), 1.03 (t, *J* = 7.4 Hz, 3H). ^{13}C NMR (126 MHz, $CDCl_3$) δ 141.5, 140.9, 130.0, 124.2, 59.4, 21.5, 16.1, 13.4.

6.1.30 1-Chloro-4-(propylsulfinyl)benzene (1ad)[25]

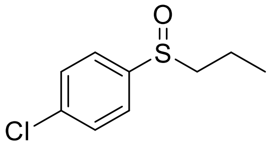

Colourless oil (183 mg, 61% yield). ^{1}H NMR (500 MHz, $CDCl_3$) δ 7.58 – 7.54 (m, 2H), 7.52 – 7.47 (m, 2H), 2.83 – 2.69 (m, 2H), 1.87 – 1.73 (m, 1H), 1.72 – 1.58 (m, 1H), 1.05 (t, *J* = 7.4 Hz, 3H). ^{13}C NMR (126 MHz, $CDCl_3$) δ 142.6, 137.3, 129.7, 125.6, 59.4, 15.9, 13.4.

6.1.31 1-Bromo-4-(propylsulfinyl)benzene (1ae)

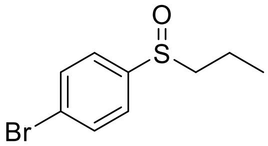

Colourless oil (278 mg, 99% yield). ν_{max}/cm^{-1}: 2963, 1470, 1005. ^{1}H NMR (500 MHz, $CDCl_3$) δ 7.65 (d, *J* = 8.4 Hz, 2H), 7.49 (d, *J* = 8.5 Hz, 2H), 2.82 – 2.68 (m, 2H), 1.87 – 1.73 (m, 1H), 1.71 – 1.58 (m,

1H), 1.04 (t, *J* = 7.4 Hz, 3H). ^{13}C NMR (126 MHz, $CDCl_3$) δ 143.3, 132.5, 125.8, 125.5, 59.3, 15.9, 13.4. HRMS (ESI) m/z calcd. for $C_9H_{12}BrO^{32}S^+$ $[M+H]^+$ 246.9714; found 246.9785, 248.9758.

6.1.32 1-Methoxy-3-(propylsulfinyl)benzene (1af)

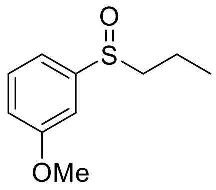

Pale yellow oil (232 mg, 77% yield). v_{max}/cm^{-1}: 2963, 1479, 1025. ^{1}H NMR (400 MHz, $CDCl_3$) δ 7.39 (td, *J* = 7.9, 1.4 Hz, 1H), 7.23 (dd, *J* = 2.6, 1.5 Hz, 1H), 7.10 (dt, *J* = 7.6, 1.2 Hz, 1H), 7.00 (ddd, *J* = 8.3, 2.5, 1.1 Hz, 1H), 3.86 (s, 2H), 2.86 – 2.68 (m, 2H), 1.87 – 1.73 (m, 1H), 1.73 – 1.58 (m, 1H), 1.09 – 1.00 (m, 3H). ^{13}C NMR (101 MHz, $CDCl_3$) δ 160.6, 145.6, 130.2, 117.5, 116.2, 108.6, 59.4, 55.7, 16.1, 13.4. HRMS (ESI) m/z calcd. for $C_9H_{15}O_2{}^{32}S^+$ $[M+H]^+$ 199.0787; found 199.0784.

6.1.33 1-Methoxy-4-(butylsulfinyl)benzene (1ag)[27]

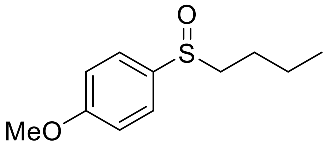

Yellow oil (195 mg, 65% yield). ^{1}H NMR (500 MHz, $CDCl_3$) δ 7.56 (d, *J* = 8.8 Hz, 2H), 7.02 (d, *J* = 8.8 Hz, 2H), 3.85 (s, 3H), 1.73 – 1.52 (m, 2H), 1.51 – 1.34 (m, 2H), 0.91 (t, *J* = 7.3 Hz, 3H). ^{13}C NMR (126 MHz, $CDCl_3$) δ 162.0, 135.0, 126.1, 114.9, 57.3, 55.6, 24.4, 22.0, 13.8.

6.1.34 Methyl(2-(phenylsulfinyl)ethyl)sulfane (2r)

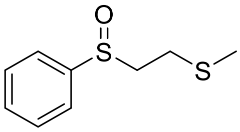

Yellow oil (32 mg, 49% yield). v_{max}/cm^{-1}: 3397, 2911, 1440, 1036. ^{1}H NMR (500 MHz, $CDCl_3$) δ 7.63 – 7.57 (m, 2H), 7.54 – 7.46 (m, 3H), 3.09 – 2.94 (m, 2H), 2.87 (ddd, *J* = 13.4, 9.7, 6.3 Hz, 1H), 2.64 – 2.58 (m, 1H), 2.09 (s, 3H). ^{13}C NMR (126 MHz, $CDCl_3$) δ 143.2, 131.3, 129.5, 129.4, 124.1, 73.4, 56.5, 26.3, 15.7. HRMS (ESI) m/z calcd. for $C_9H_{13}O_2{}^{32}S^{2+}$ $[M+H]^+$ 201.0399; found 201.0402.

6.2 Isolated yields and optical rotation data of (*R*)-sulfoxides

Table S4. Isolated yields and optical rotation data

Compound	Mass / mg	Yield %	Optical rotation / $[\alpha]_D^{27}$
**(*R*)-1a**	11.6	48	+169.8 (c 1.0, in $CHCl_3$) [14]
**(*R*)-1b**	18	35	+80.4 (c 1.0, in $CHCl_3$) [28]
**(*R*)-1c**	18	26	+46.8 (c 1.0, in $CHCl_3$) [17]
**(*R*)-1d**	28	41	+160.2 (c 1.0, in $CHCl_3$) [14]
**(*R*)-1e**	17	38	+82.8 (c 1.0, in $CHCl_3$) [29]
**(*R*)-1f**	20	40	+65.0 (c 1.0, in $CHCl_3$) [30]
**(*R*)-1g**	9	30	+125.2 (c 1.0, in $CHCl_3$) [30]
**(*R*)-1h**	35	50	+155.4 (c 1.0, in $CHCl_3$) [15]
**(*R*)-1i**	30	43	+144.2 (c 1.0, in $CHCl_3$) [14]
**(*R*)-1j**	24	35	+94.0 (c 1.0, in $CHCl_3$) [31]
**(*R*)-1k**	32	46	+88.6 (c 1.0, in $CHCl_3$) [14]
**(*R*)-1l**	29	41	+104.4 (c 1.0, in $CHCl_3$)
**(*R*)-1m**	40	55	+88.6 (c 1.0, in $CHCl_3$) [17]
**(*R*)-1n**	61	87	+58.4 (c 3.0, in $CHCl_3$) [32]
**(*R*)-1o**	20	29	+64.6 (c 1.0, in $CHCl_3$) [14]
**(*R*)-1p**	32	46	-51.6 (c 1.0, in $CHCl_3$) [33]
**(*R*)-1r**	34	49	-96.0 (c 1.0, in $CHCl_3$)
**(*R*)-1s**	33	47	+137.6 (c 1.0, in $CHCl_3$) [28]
**(*R*)-1t**	11	37	+123.3 (c 1.0, in $CHCl_3$) [14]
**(*R*)-1u**	18	55	+94.6 (c 1.0, in $CHCl_3$) [28]
**(*R*)-1v**	25	36	+76.0 (c 1.0, in $CHCl_3$) [14]
**(*R*)-1w**	28	40	-44.8 (c 1.0, in $CHCl_3$)

6.3 Copies of NMR spectra

6.3.1 1-Fluoro-4-(methylsulfinyl)benzene (1c)

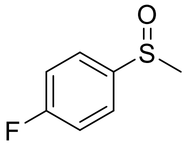

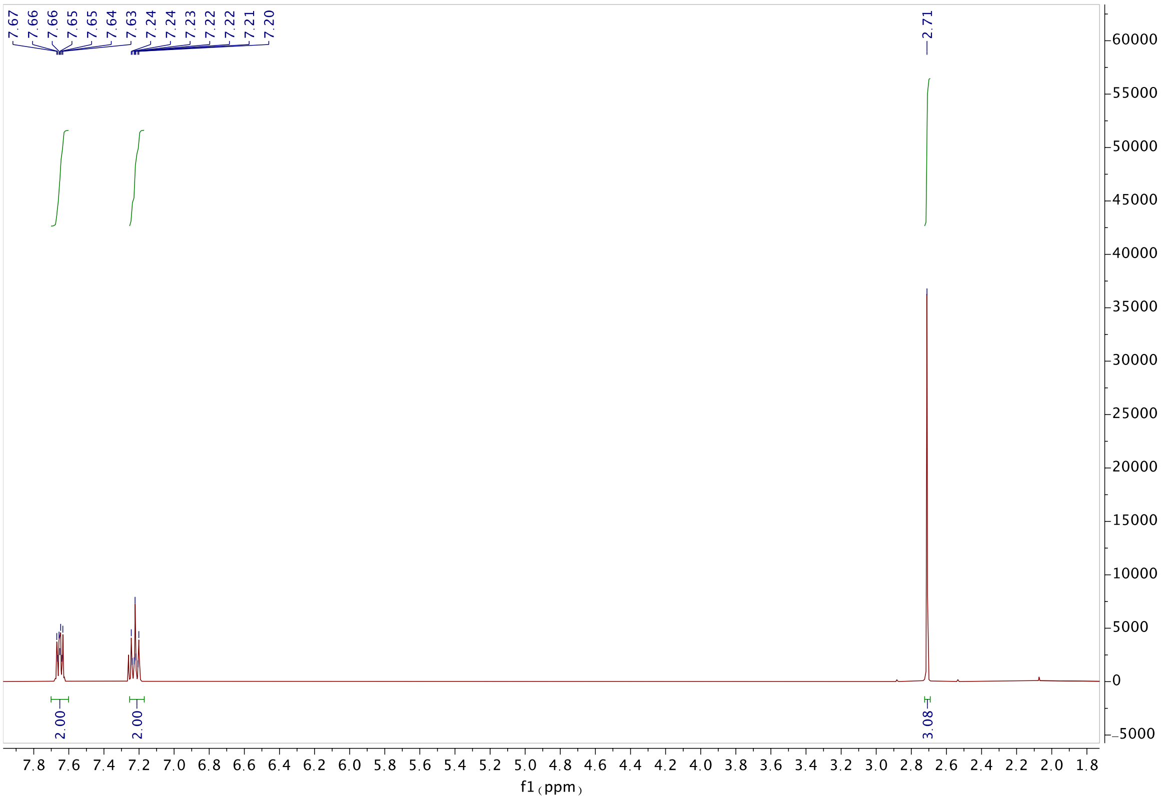

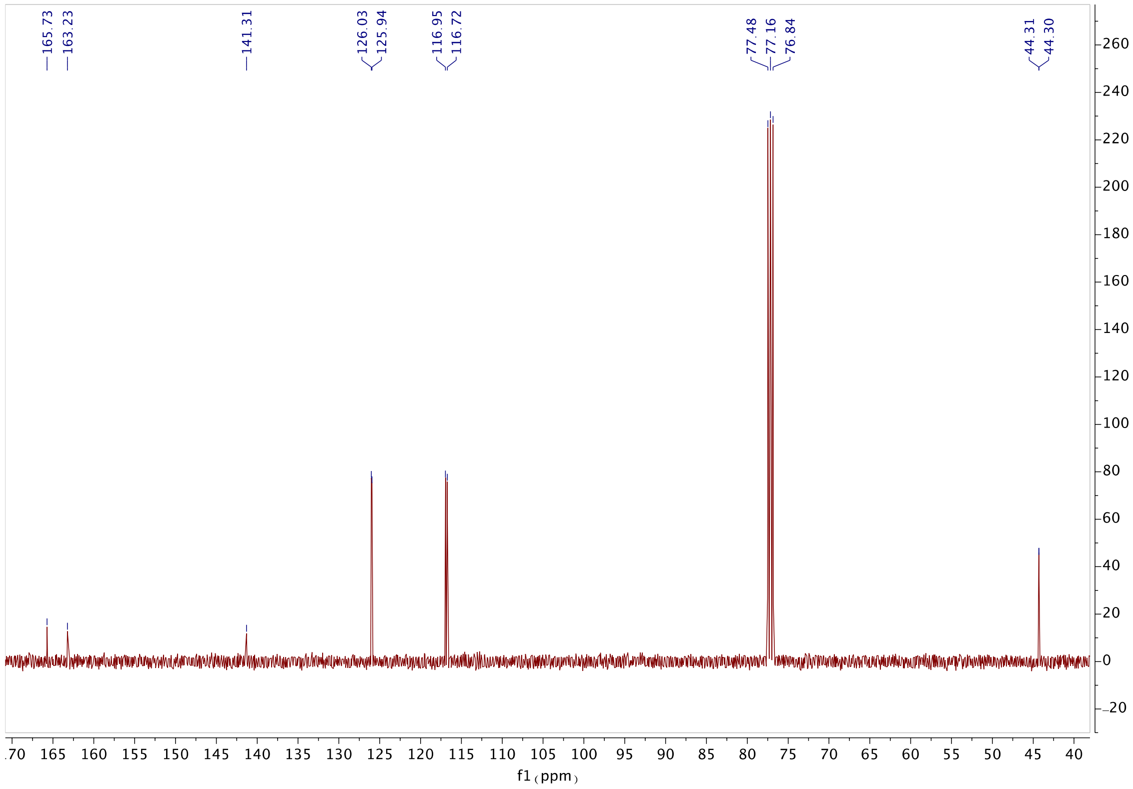

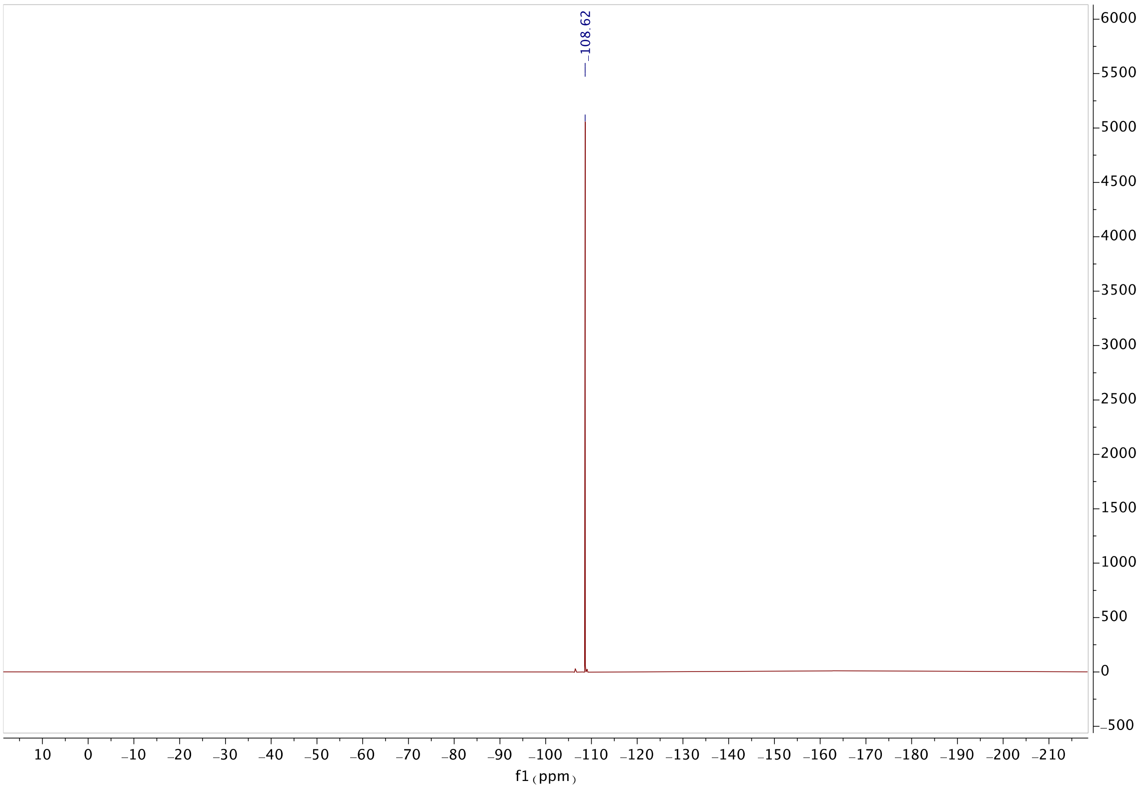
-108.62
f1 (ppm)

6.3.2 1-Bromo-4-(methylsulfinyl)benzene (1d)

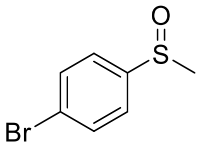

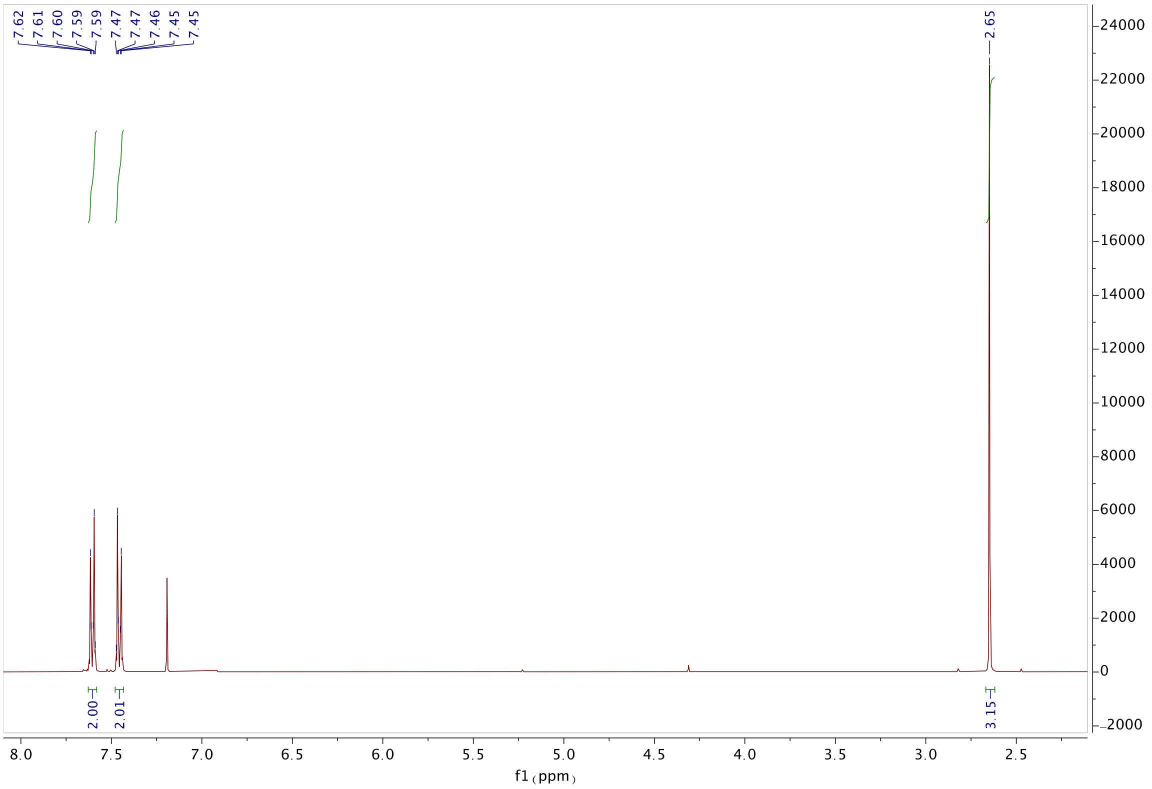

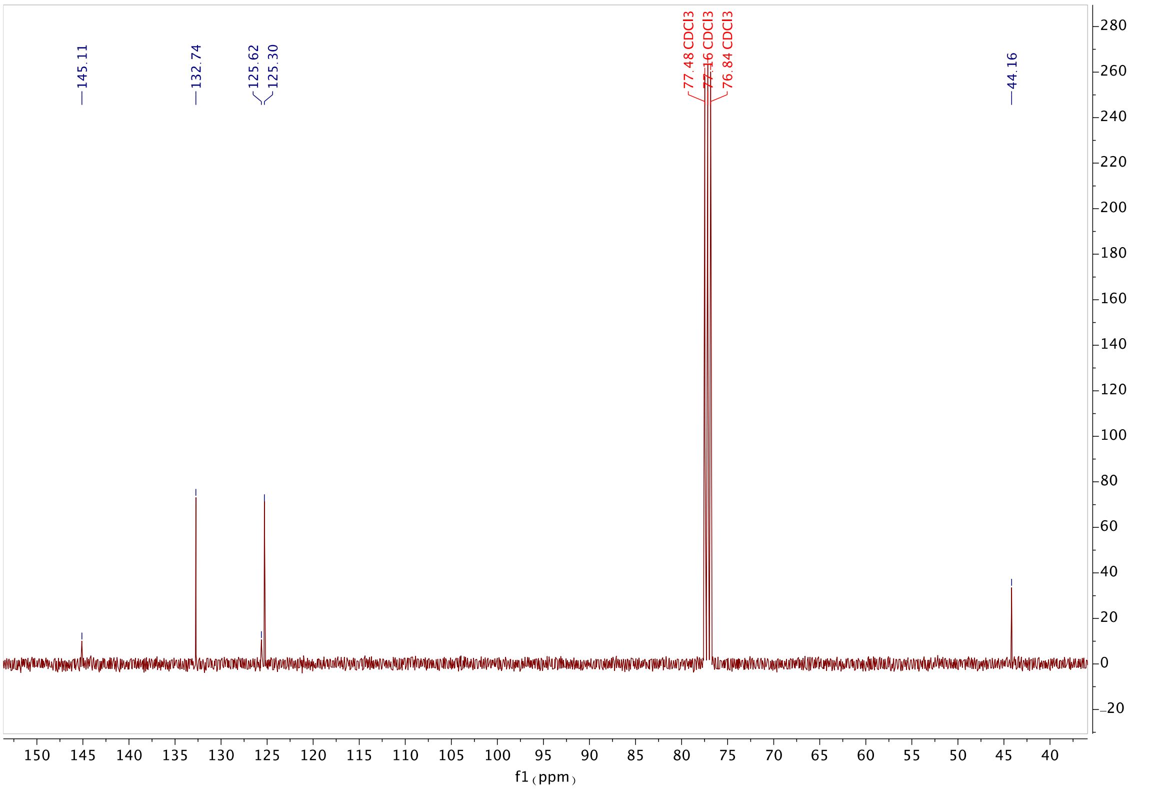

6.3.3 1-Chloro-4-(methylsulfinyl)benzene (1e)

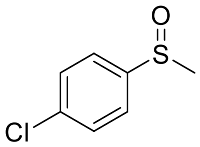

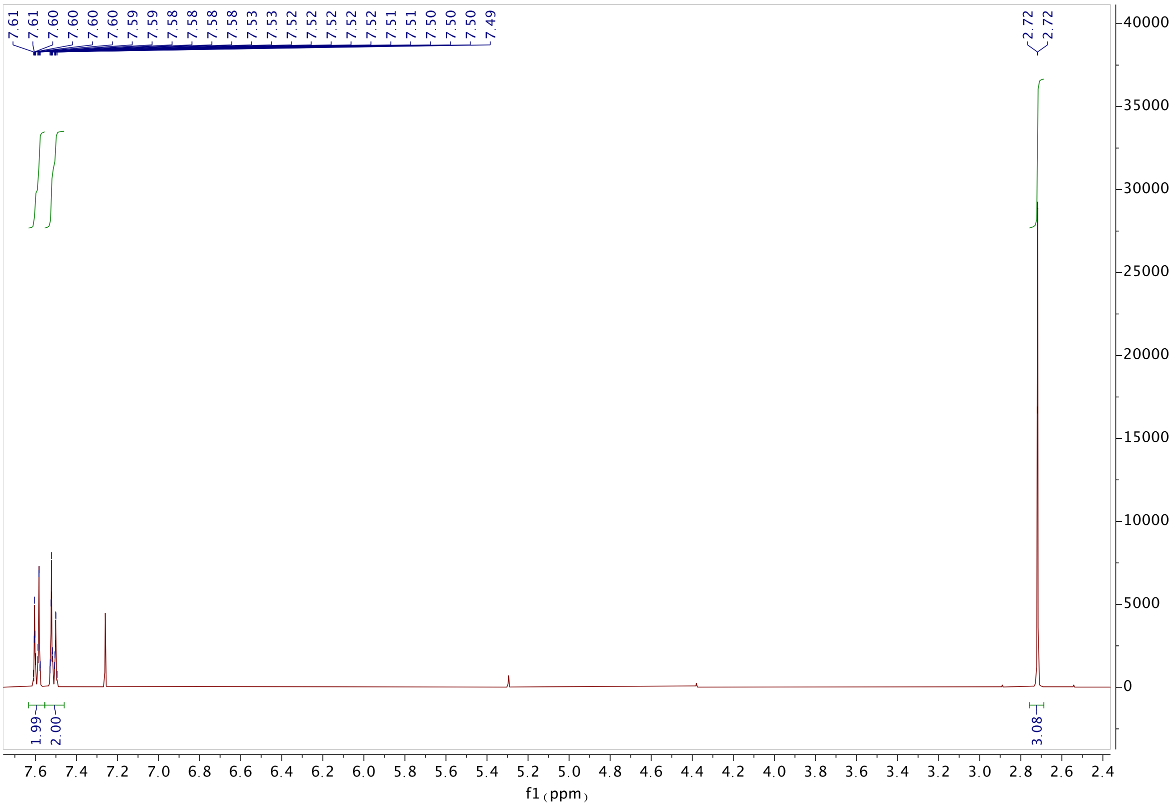

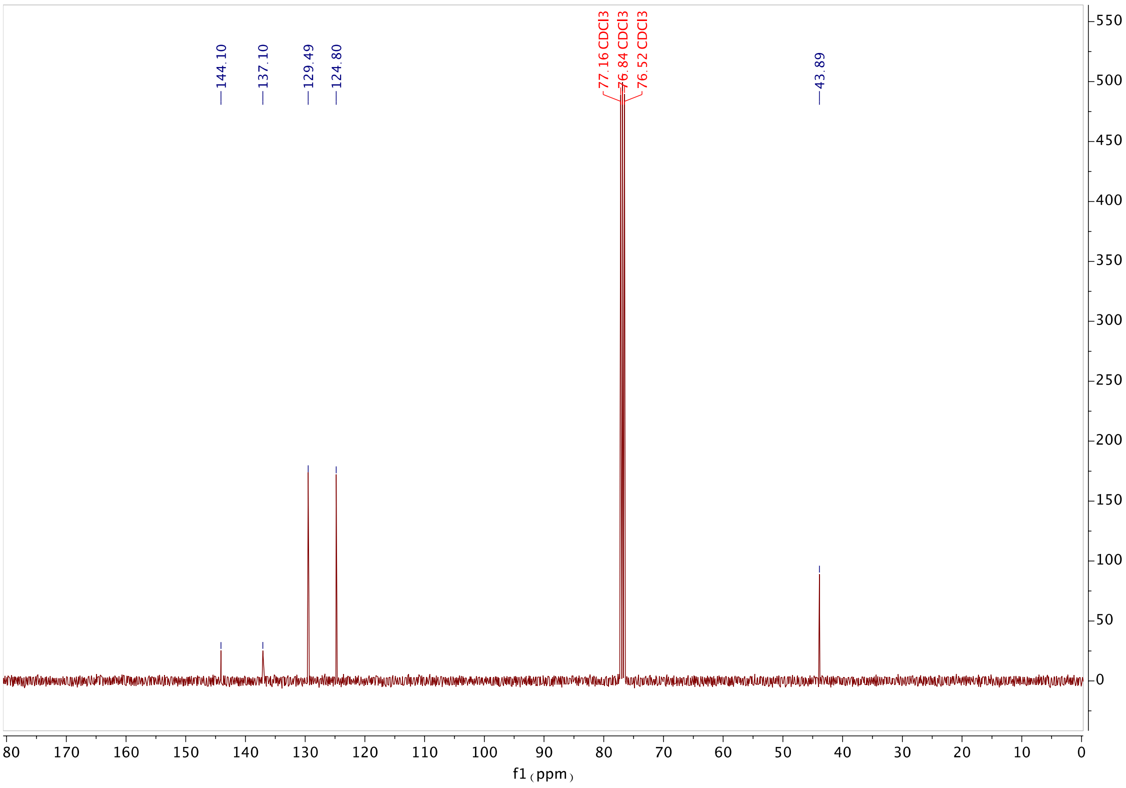

6.3.4 1-chloro-3-(methylsulfinyl)benzene (1f)

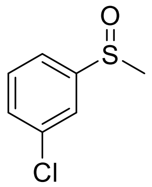

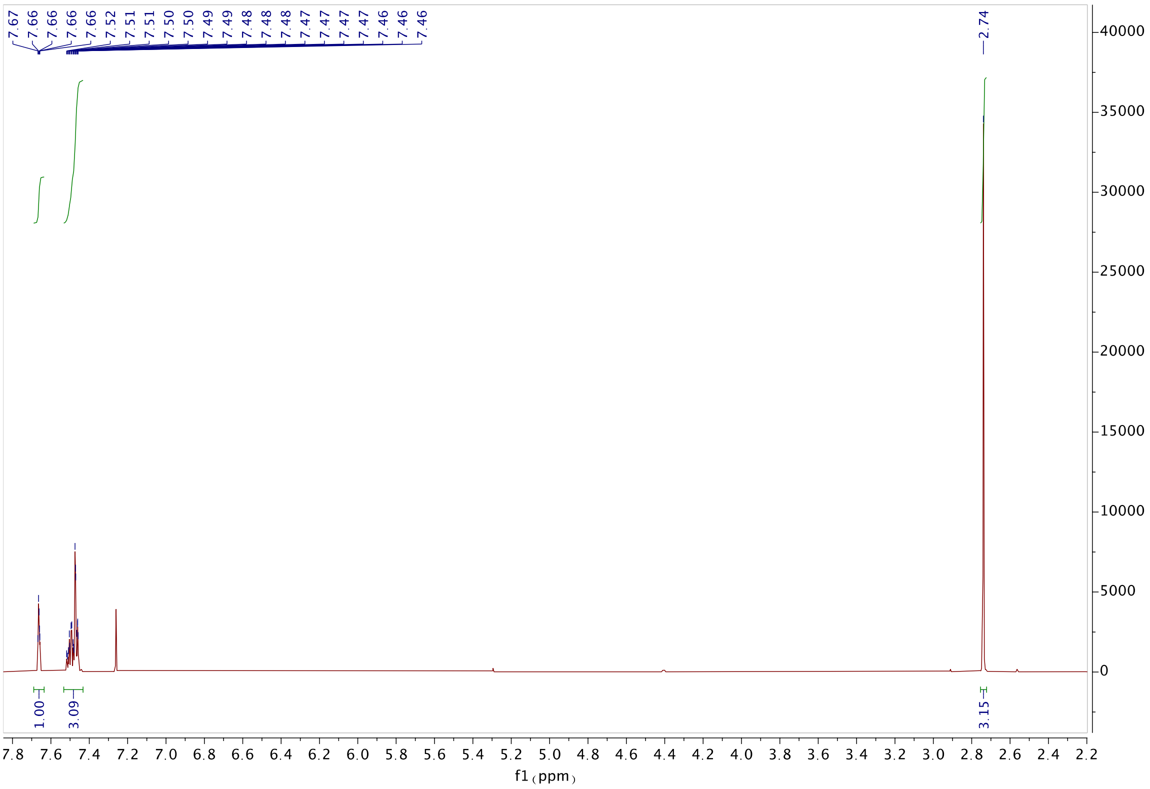

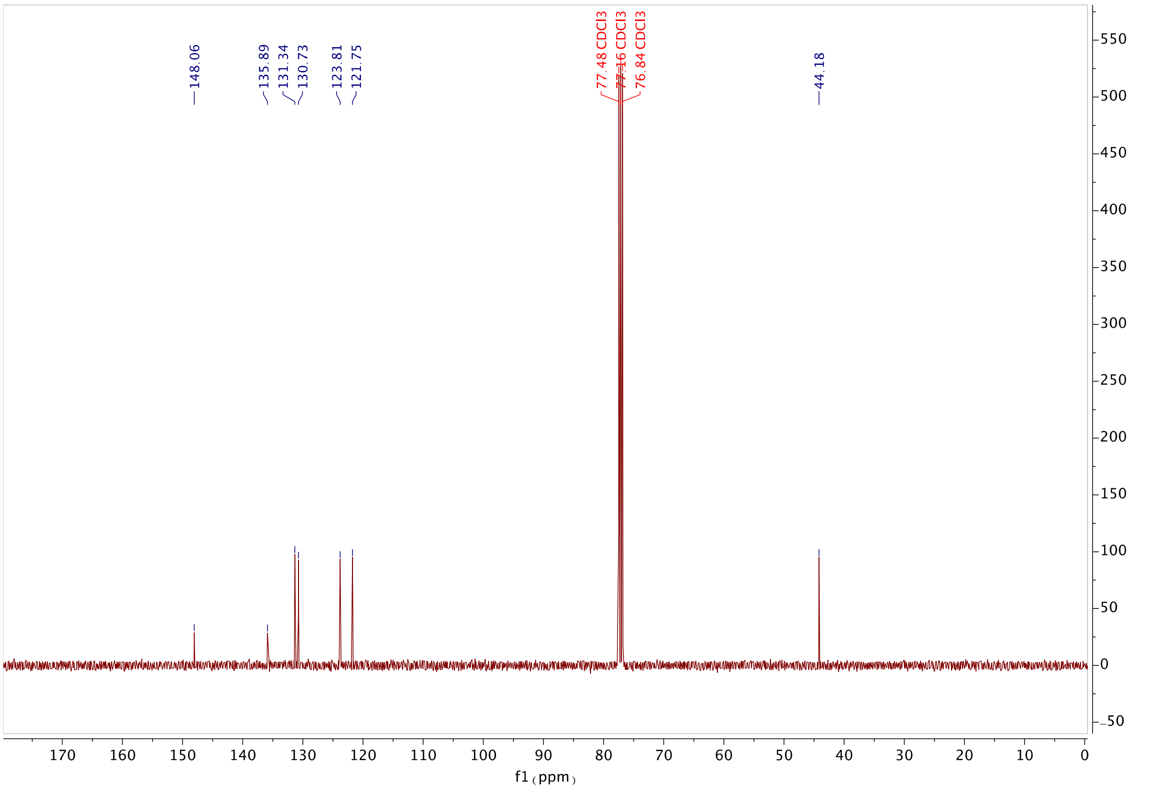

6.3.5 1-Chloro-2-(methylsulfinyl)benzene (1g)

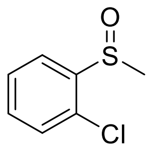

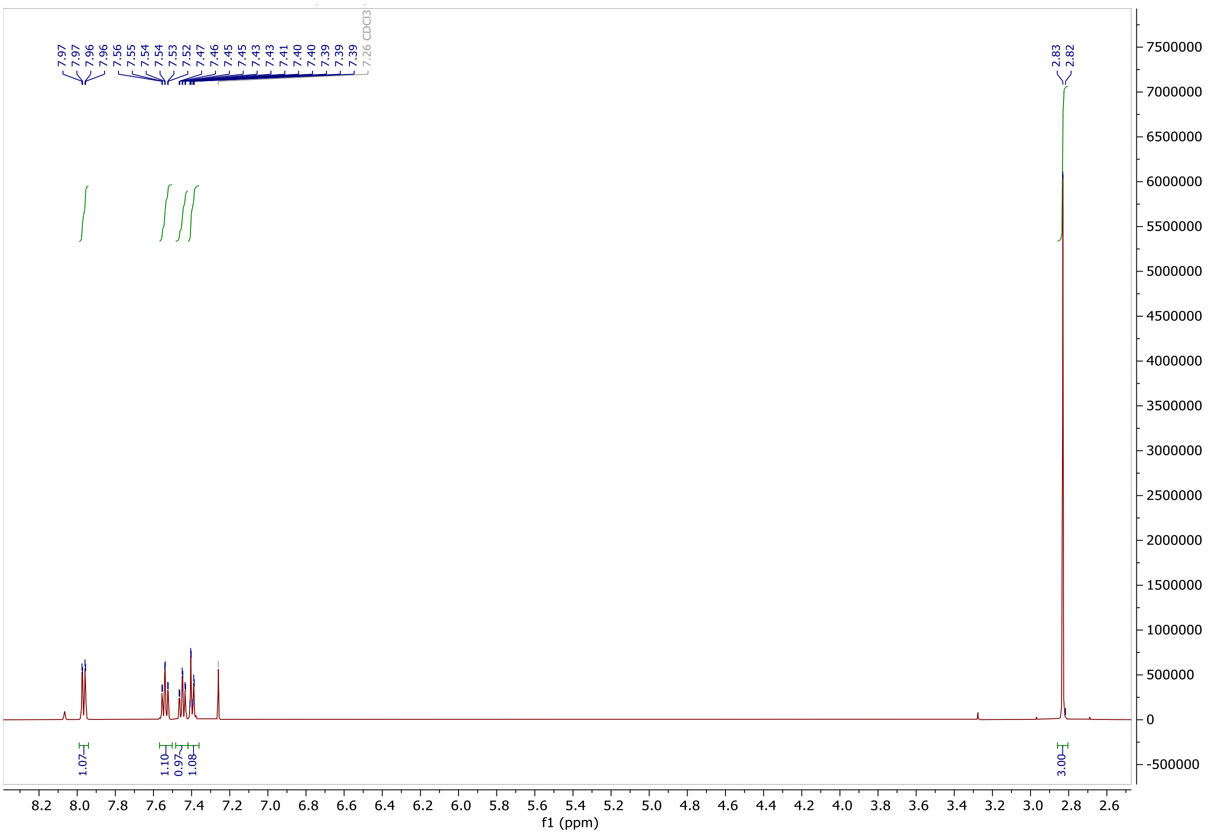

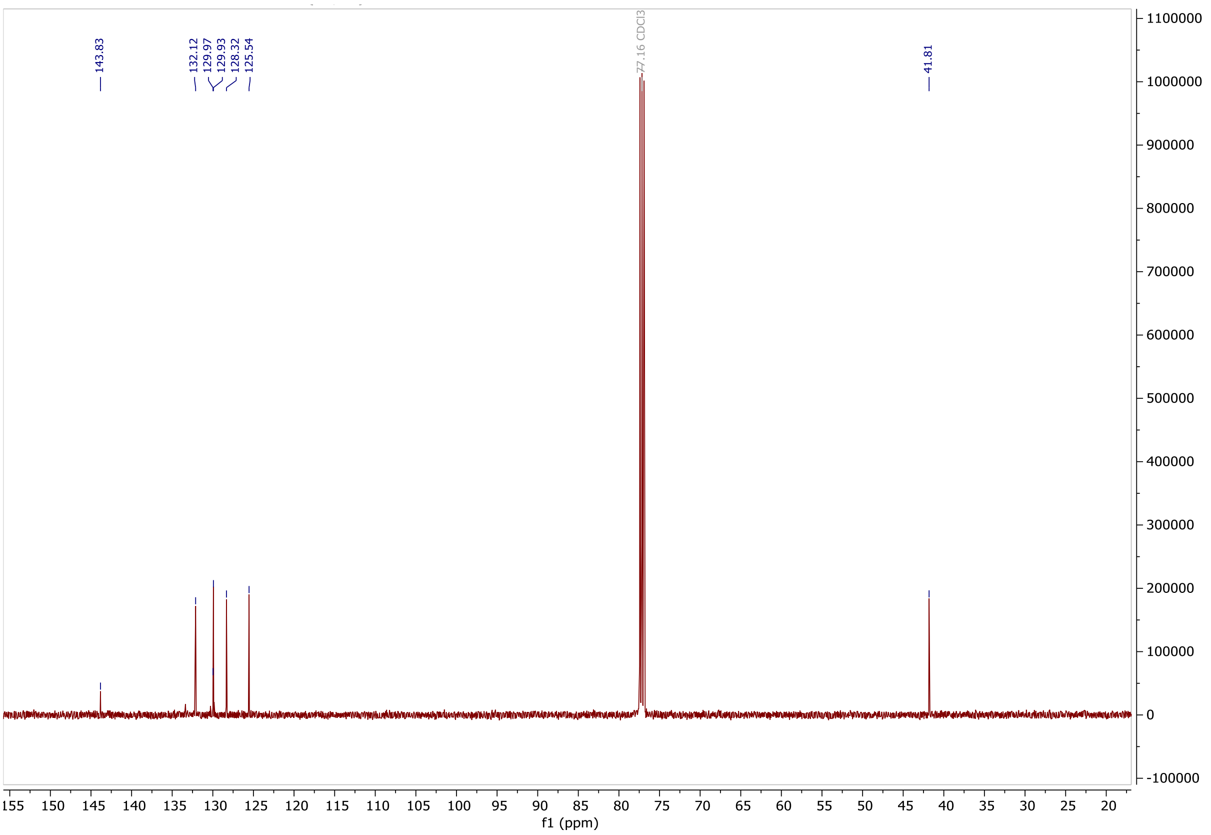

6.3.6 1-(4-(Methylsulfinyl)phenyl)ethan-1-one (1h)

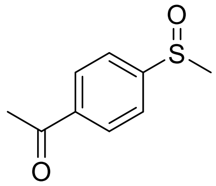

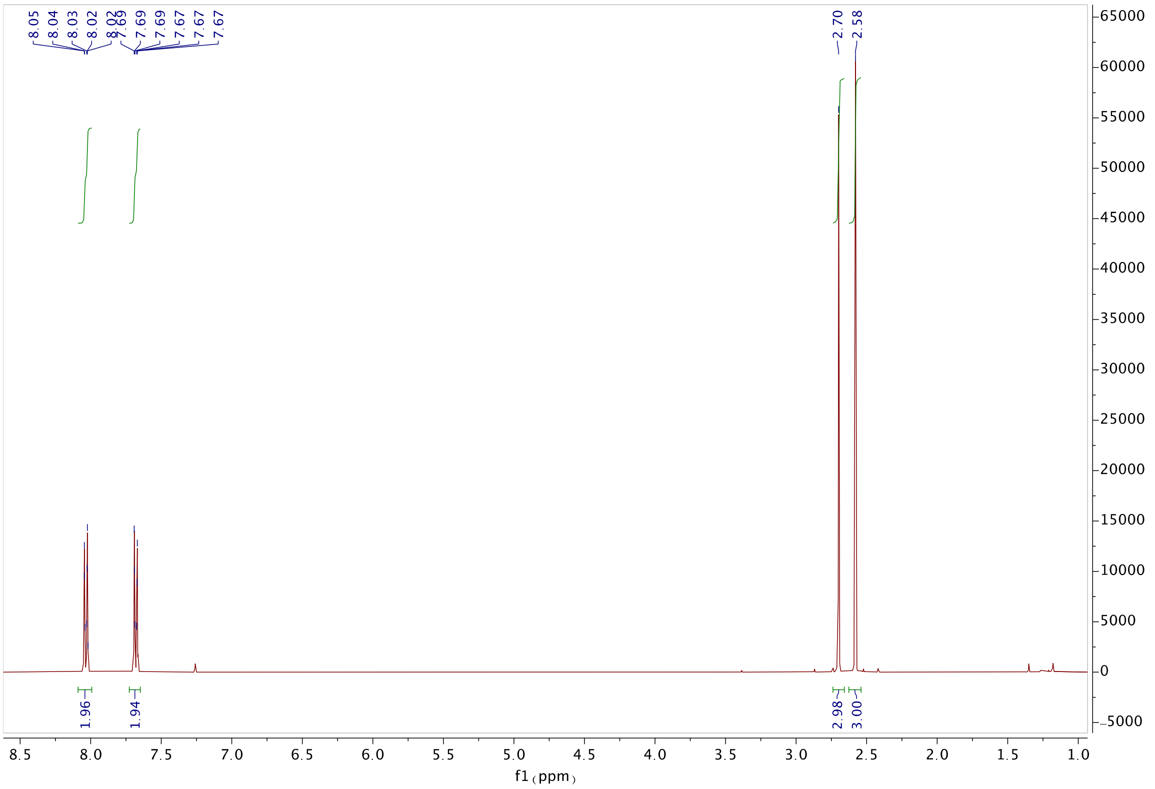

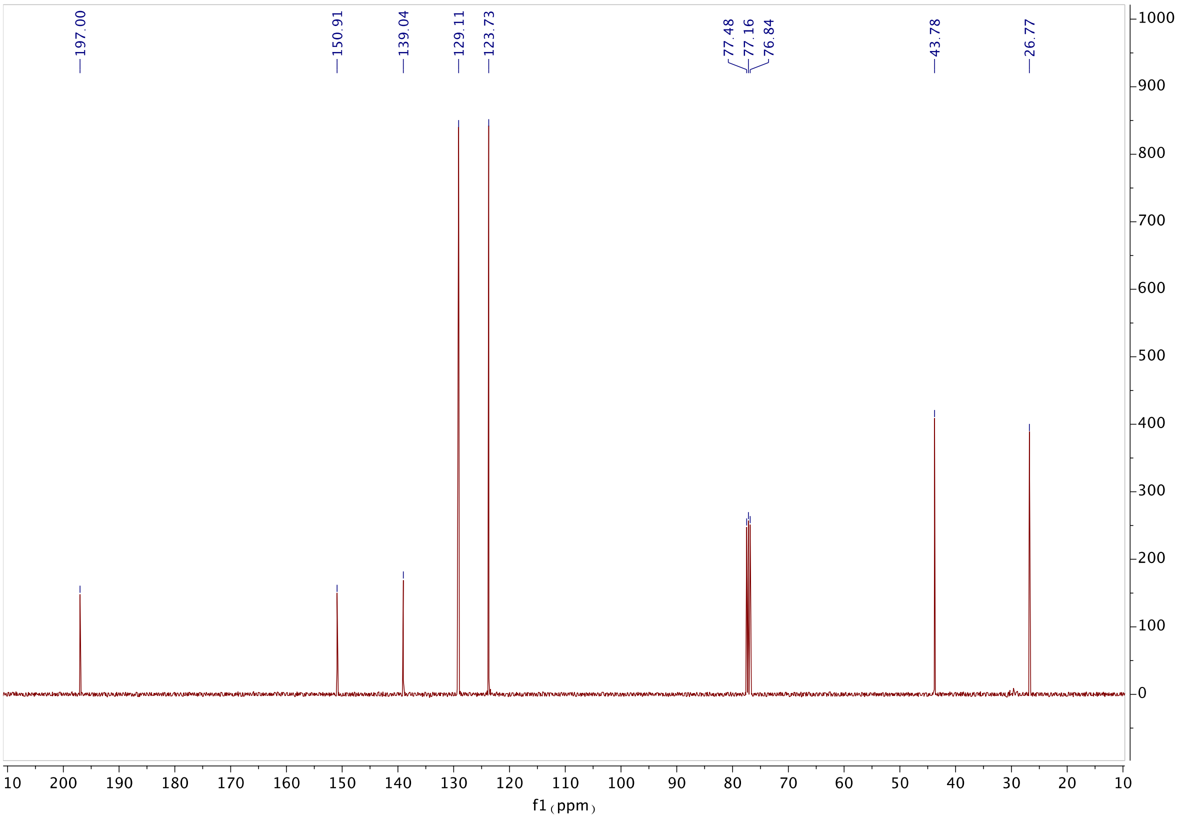

6.3.7 1-Methoxy-4-(methylsulfinyl)benzene (1i)

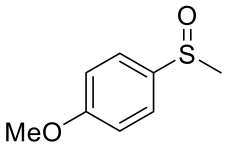

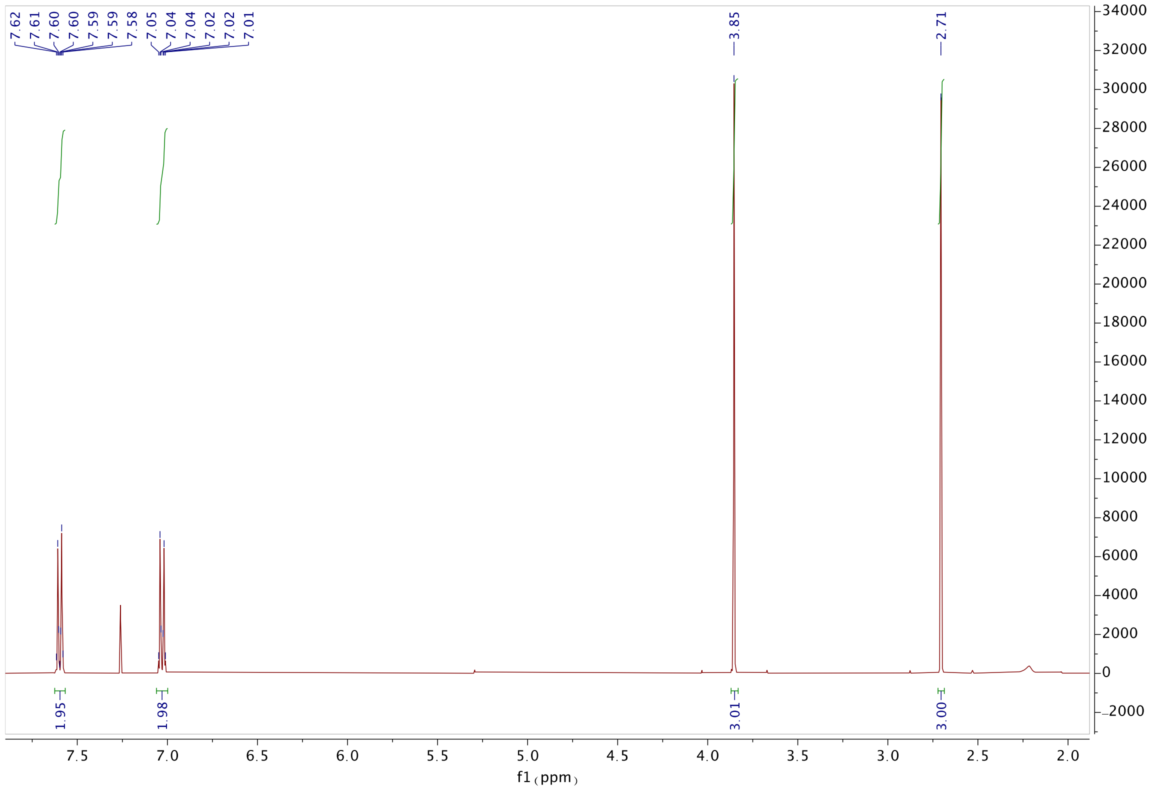

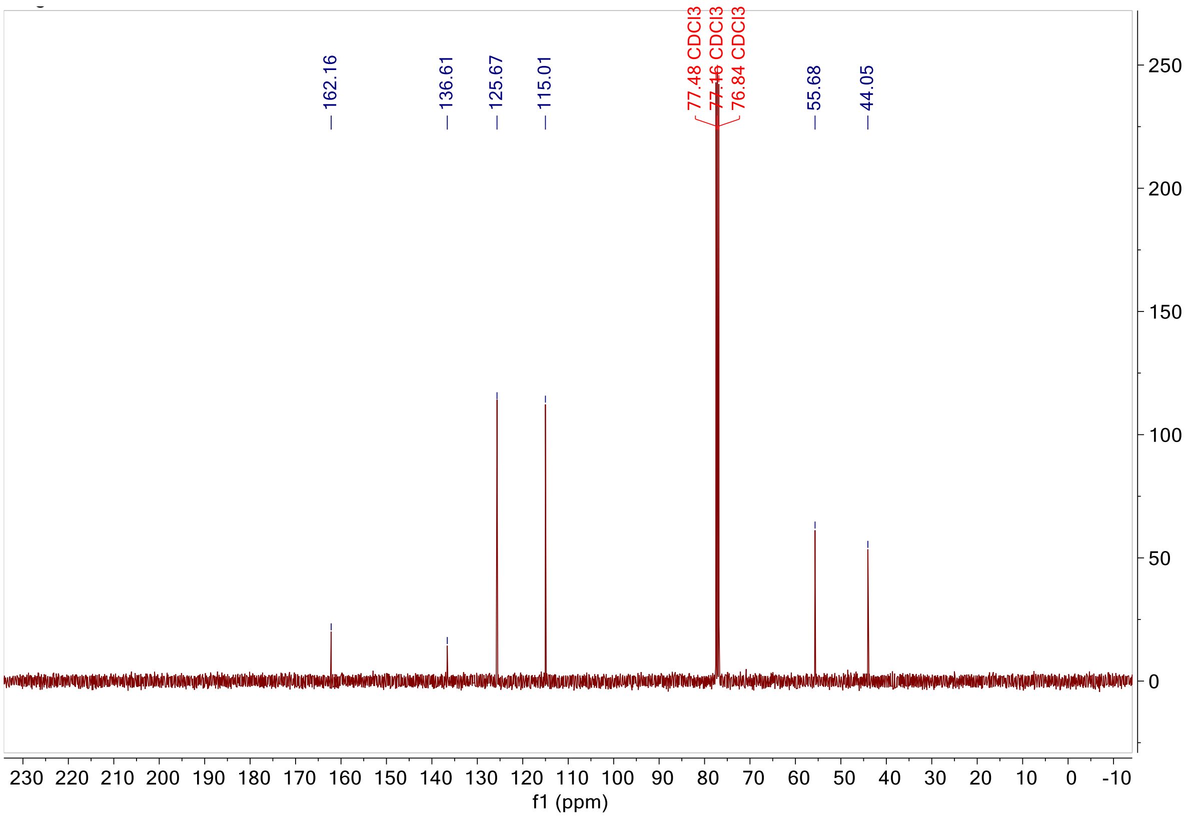

6.3.8 1-Methoxy-3-(methylsulfinyl)benzene (1j)

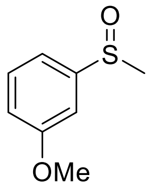

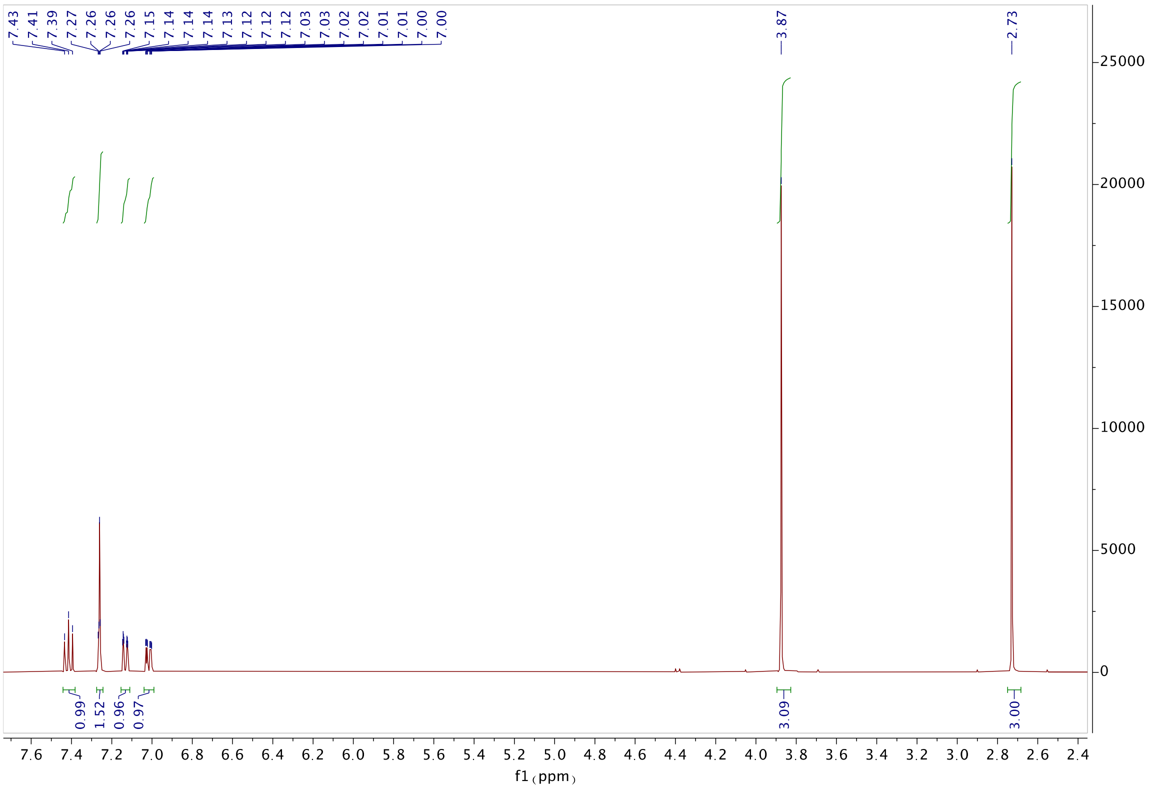

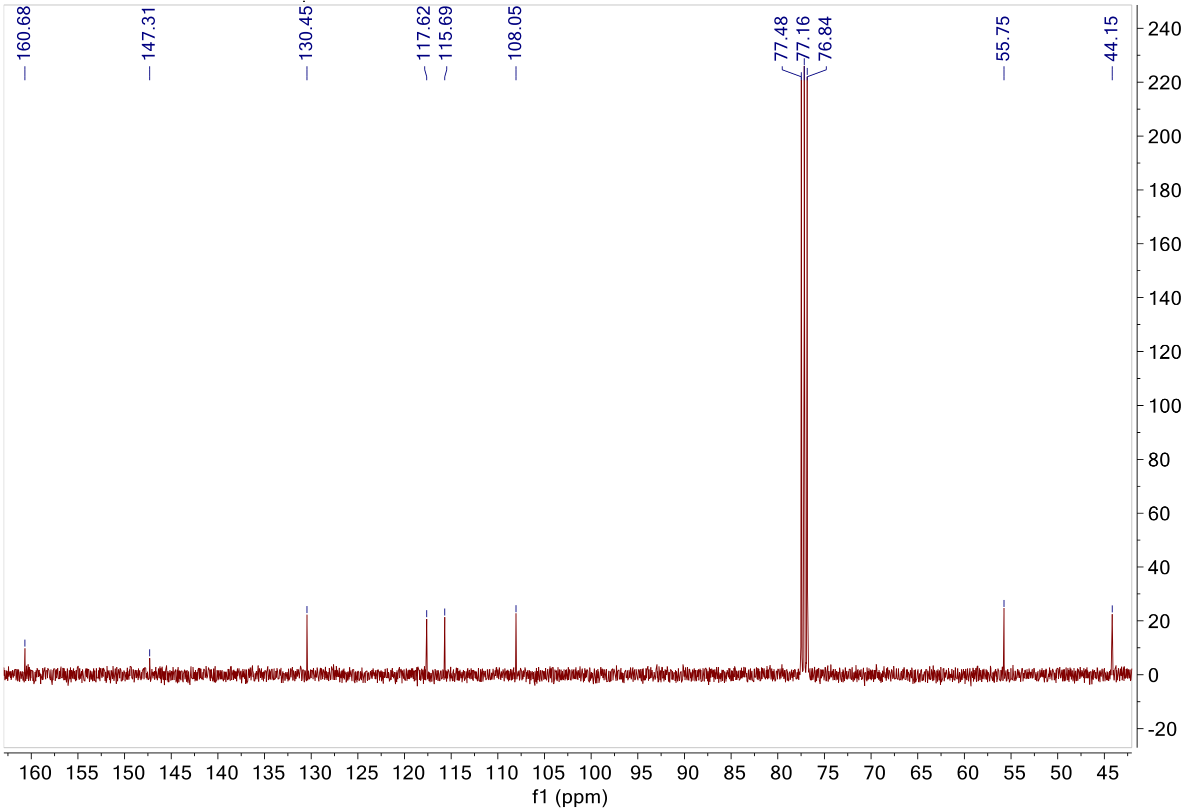

6.3.9 1-Methyl-3-(methylsulfinyl)benzene (1k)

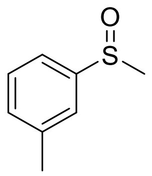

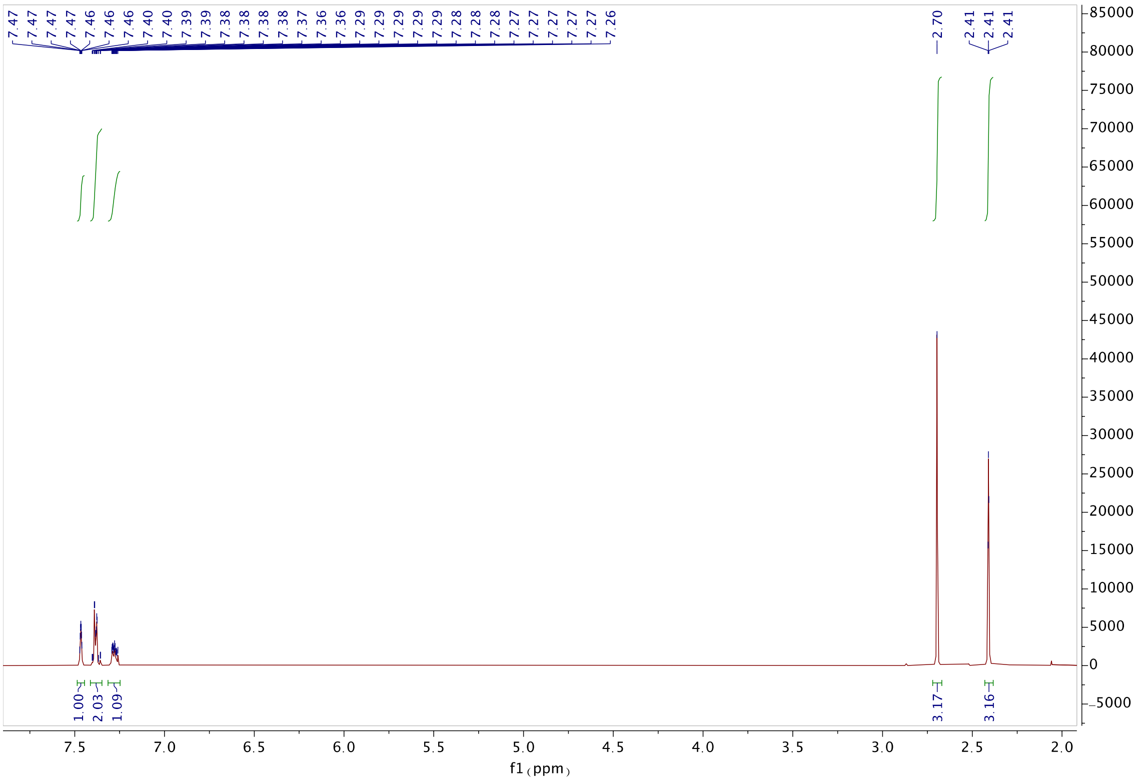

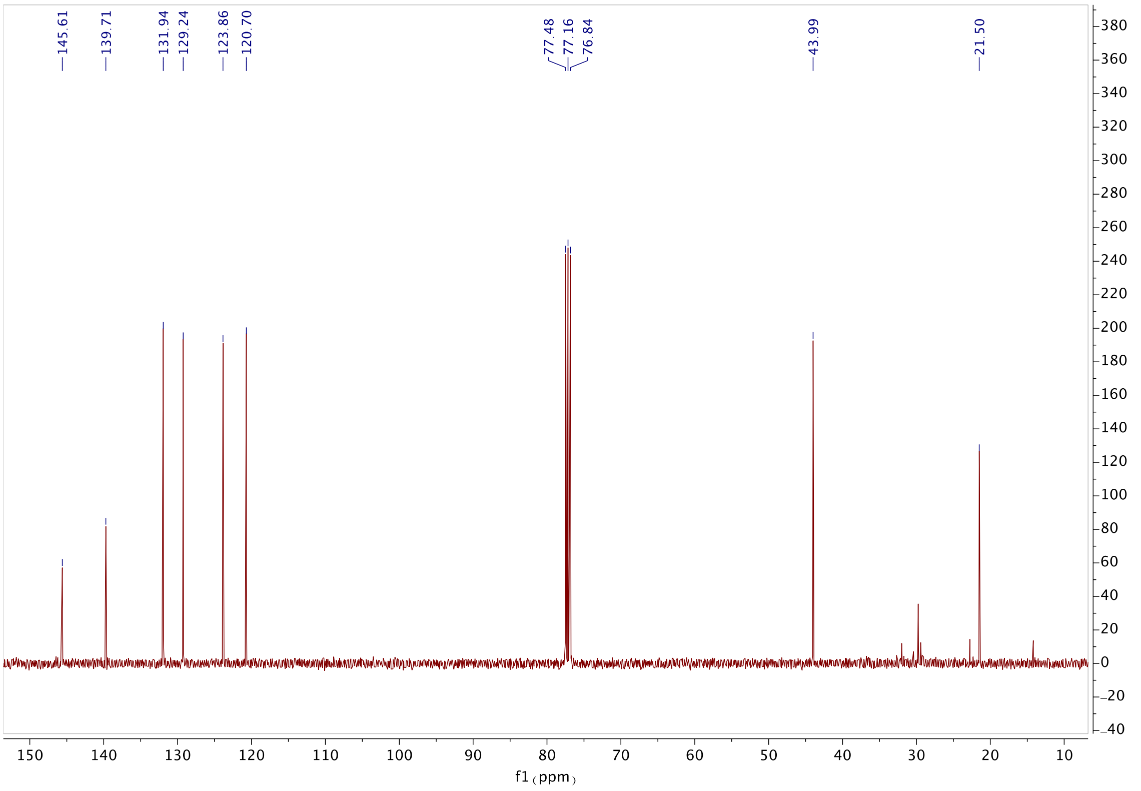

6.3.10 2,4-Dimethyl-1-(methylsulfinyl)benzene (1l)

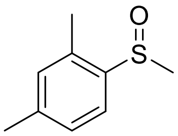

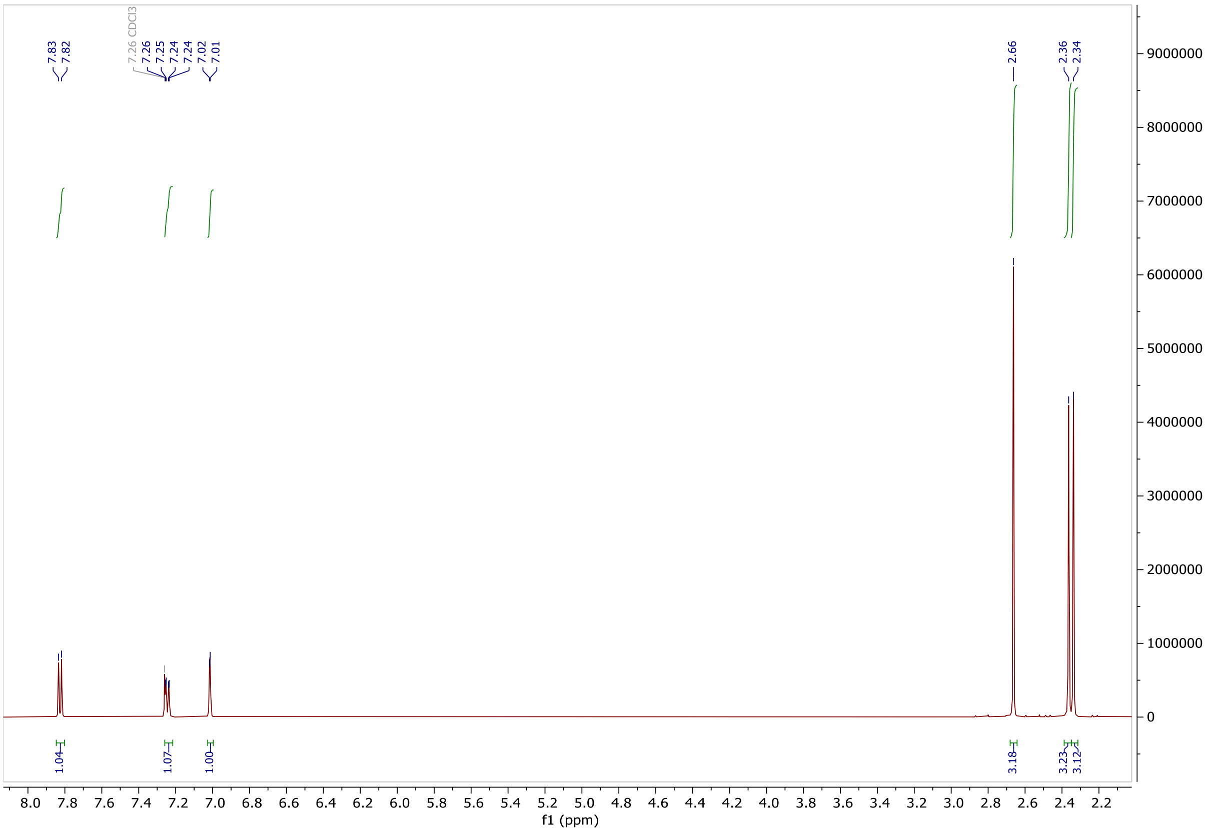

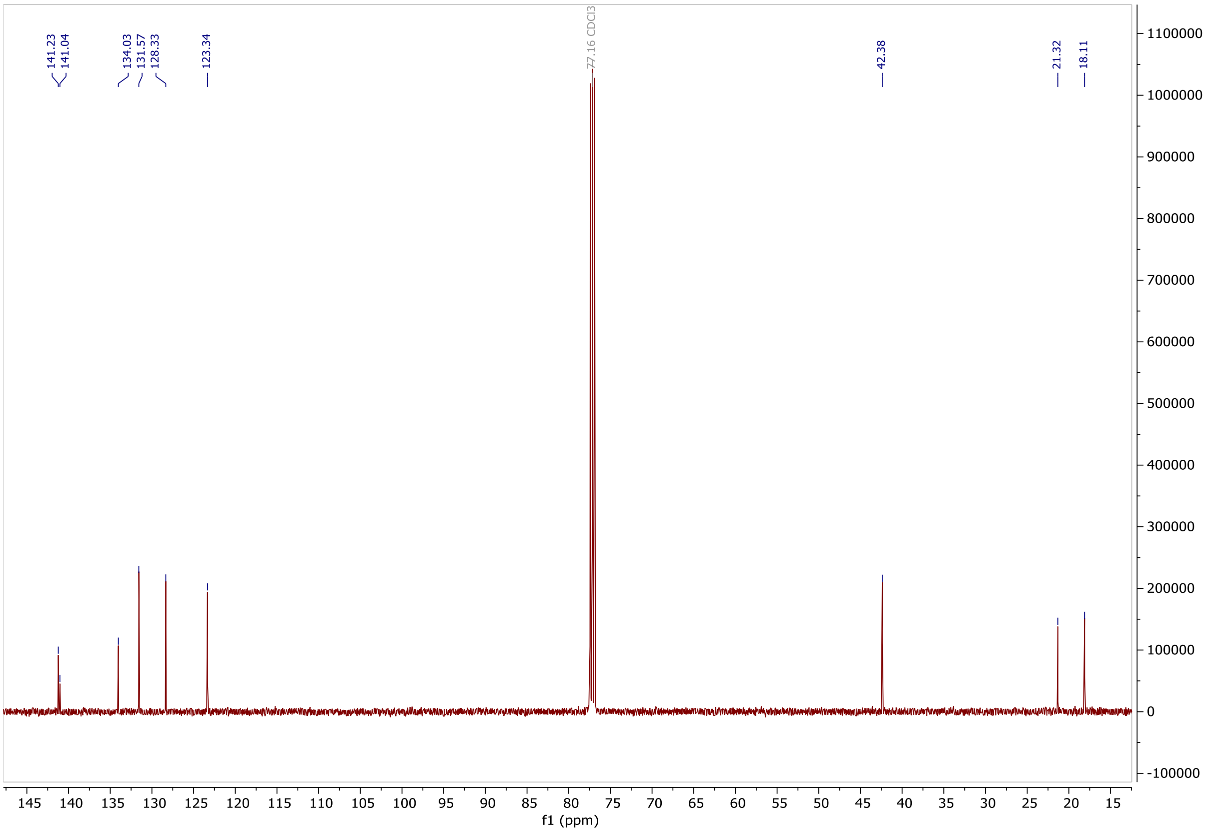

6.3.11 2-(Methylsulfinyl)naphthalene (1m)

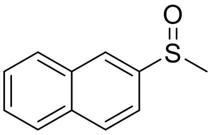

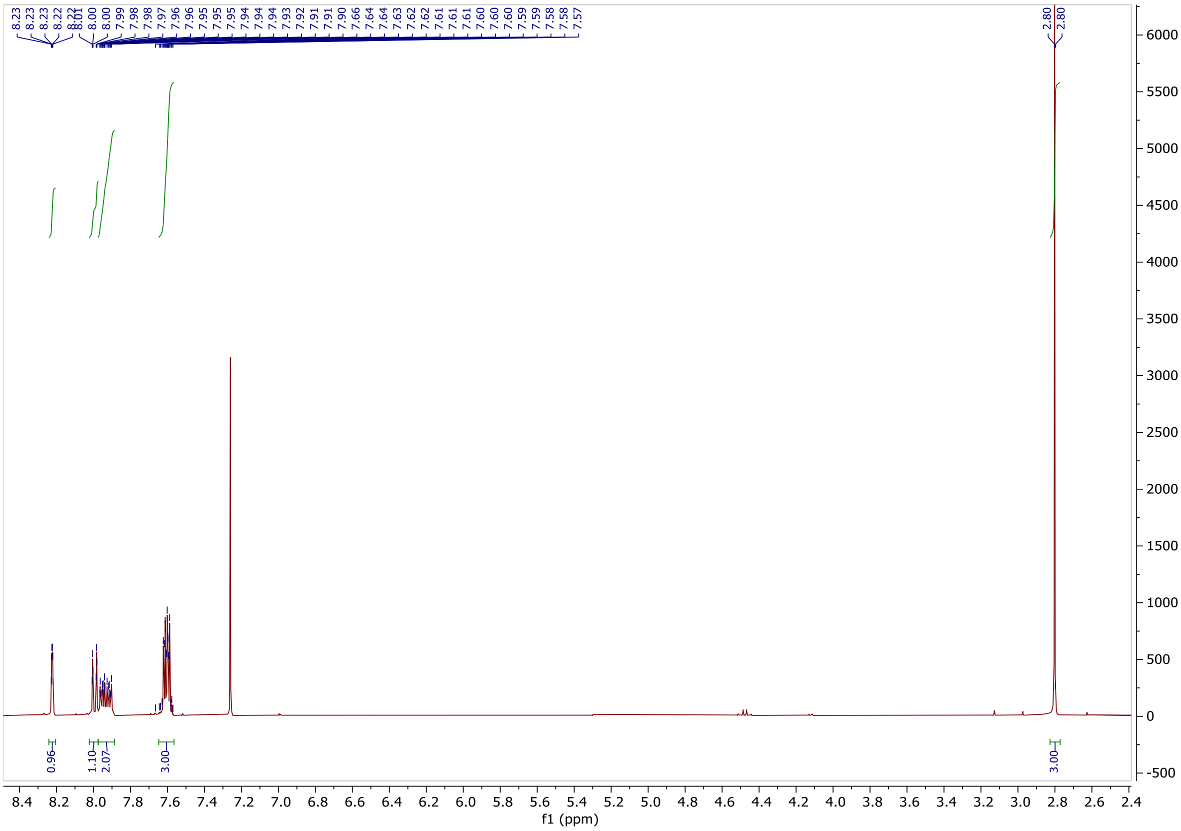

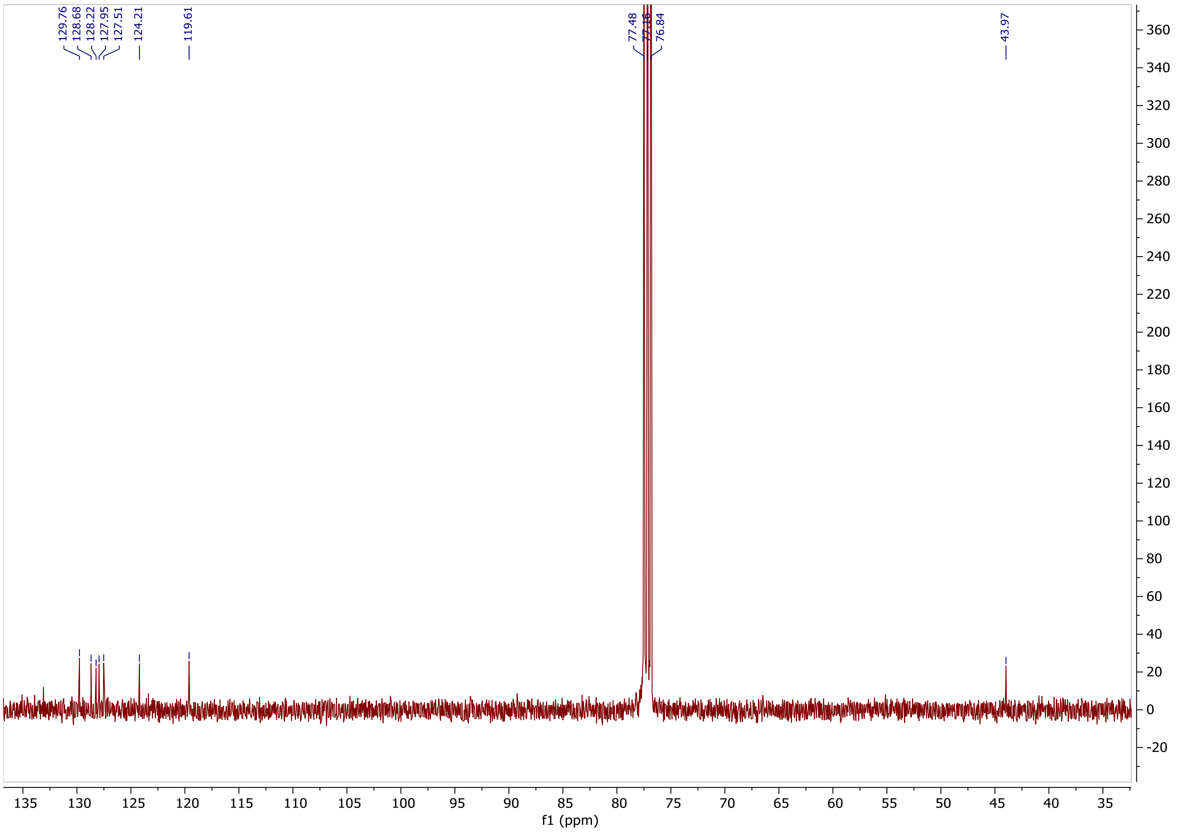

6.3.12 1-(methylsulfinyl)naphthalene (1n)

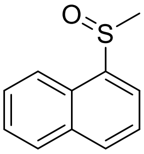

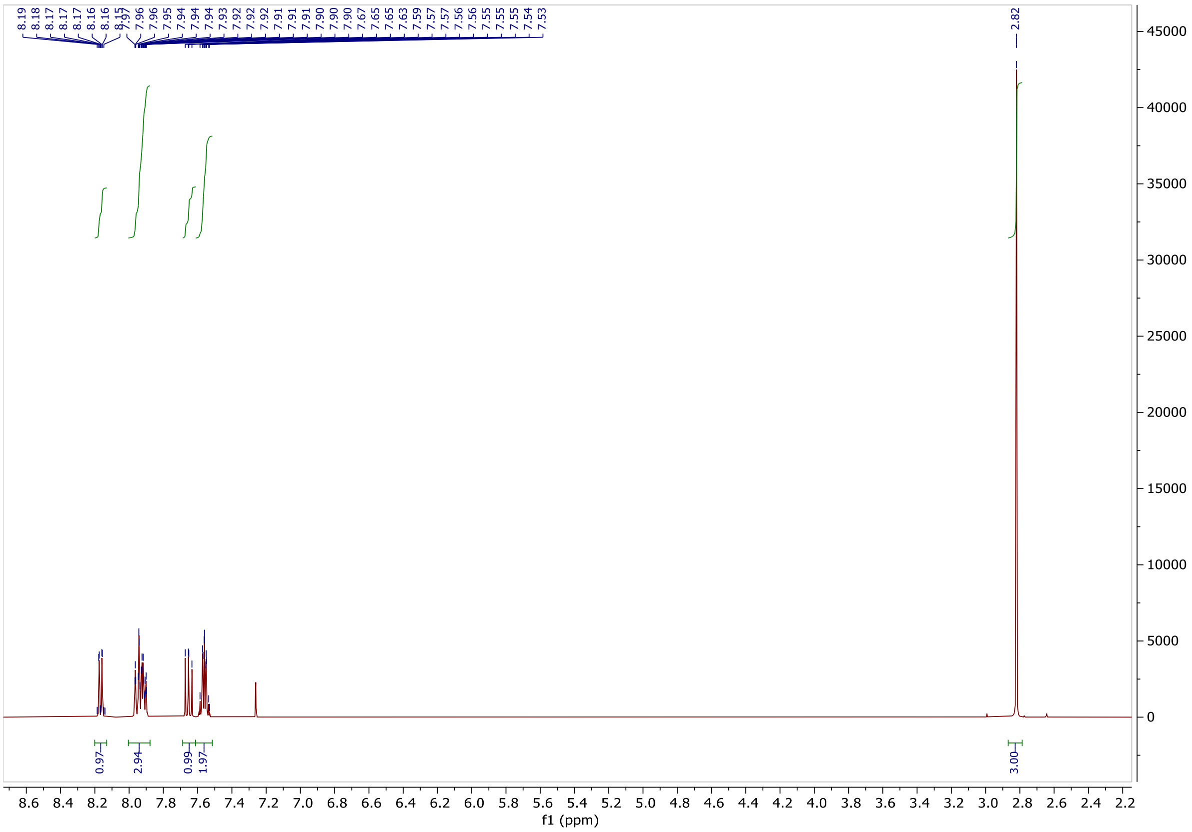

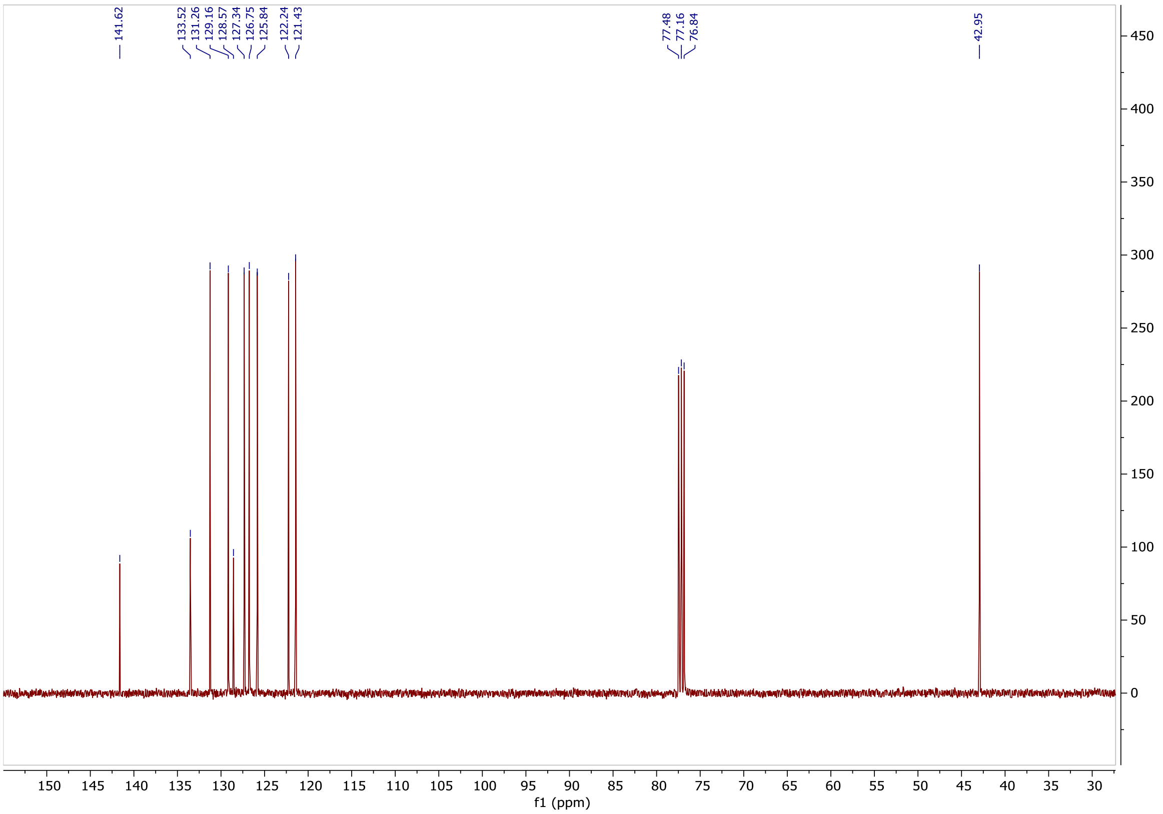

6.3.13 ((Methylsulfinyl)methyl)benzene (1o)

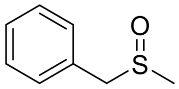

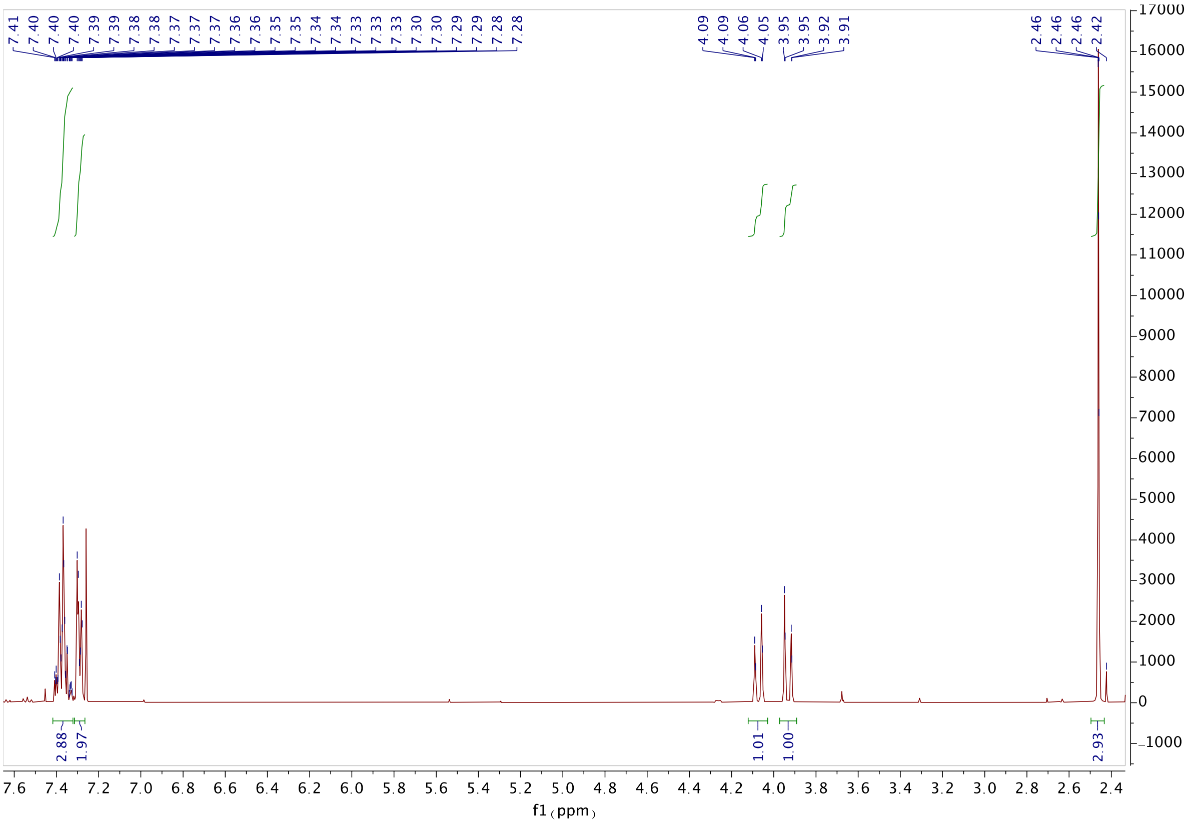

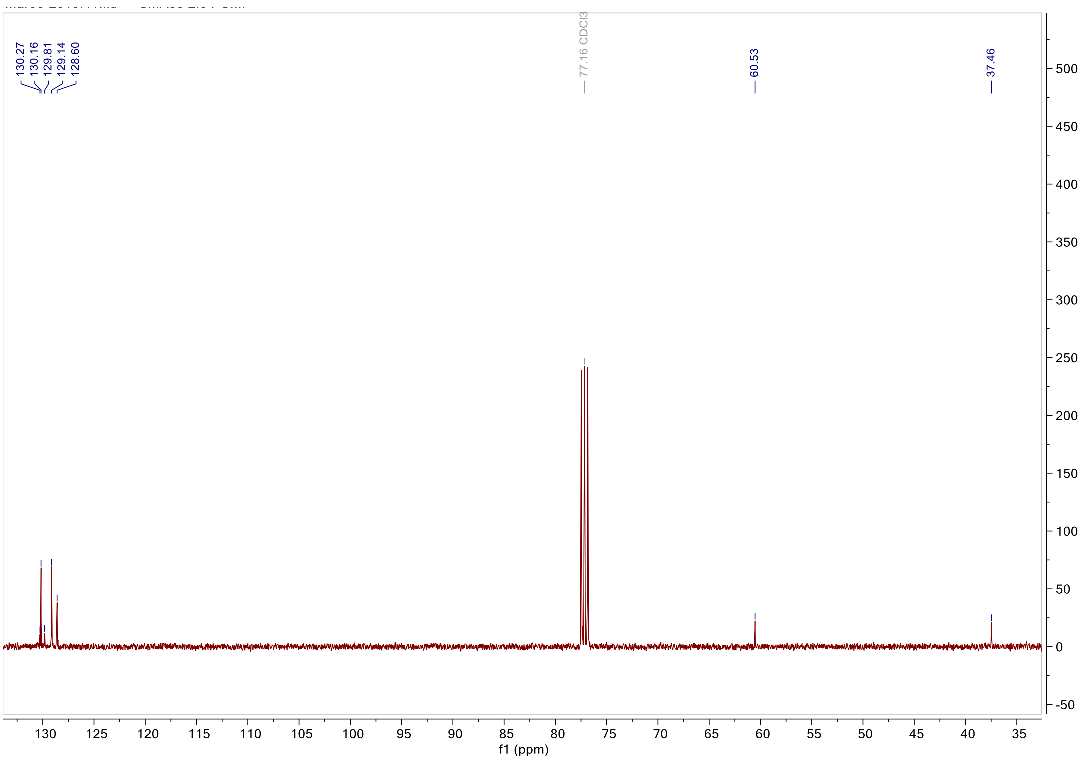

6.3.14 4-(Methylsulfinyl)butan-2-one (1q)

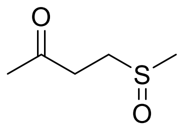

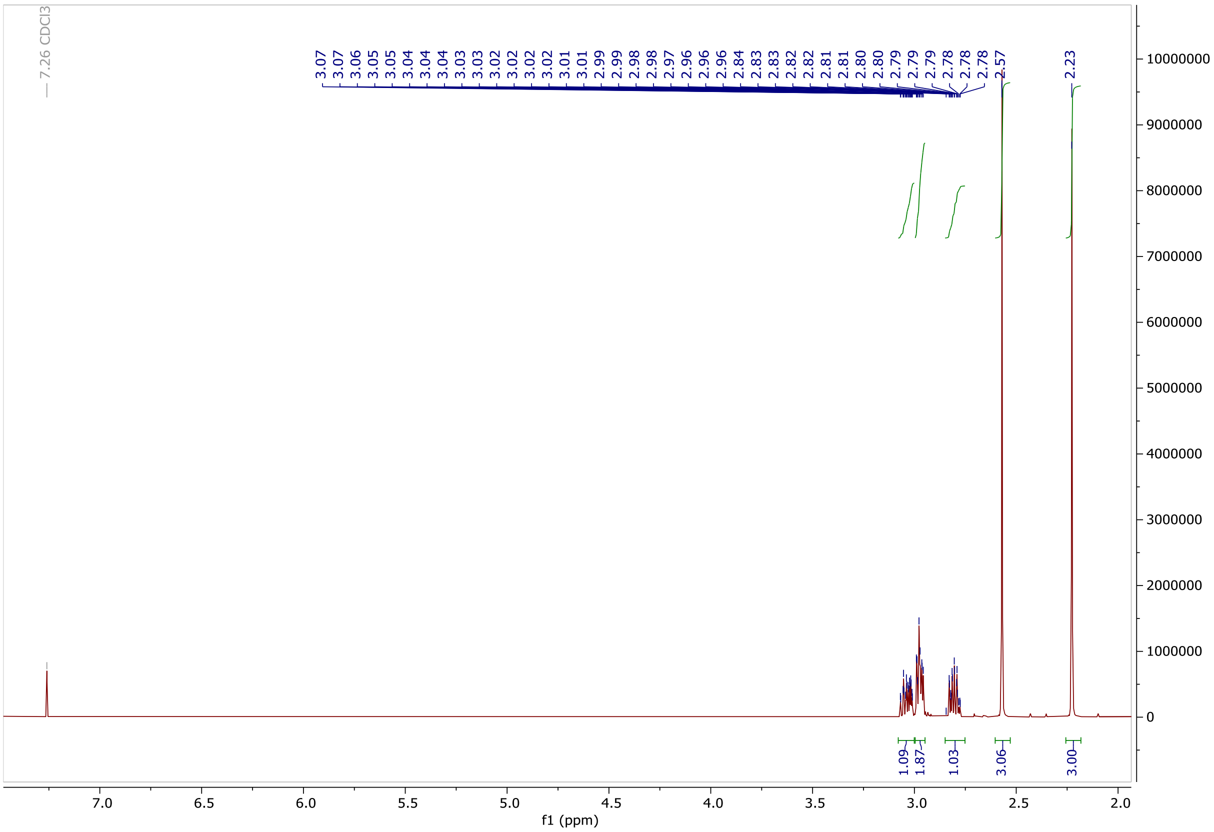

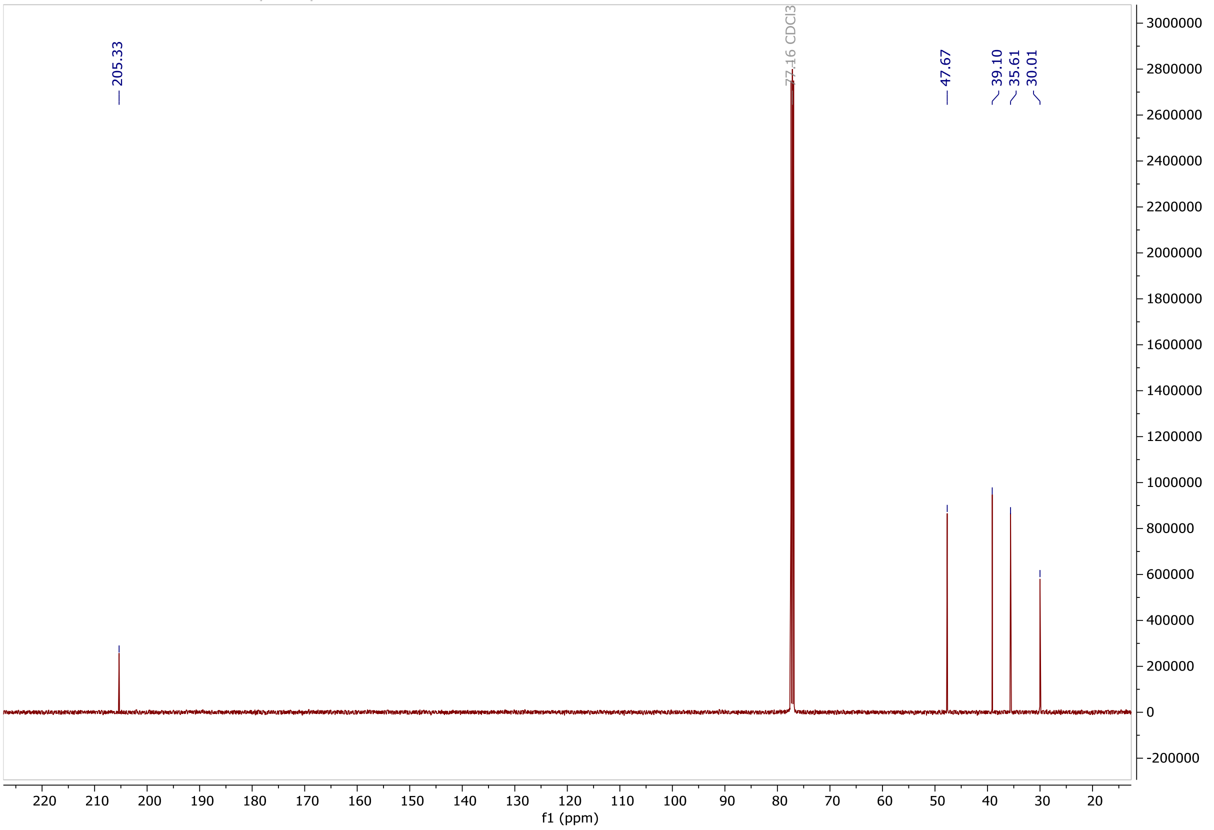

6.3.15 ((2-(Methylsulfinyl)ethyl)sulfinyl)benzene (1r)

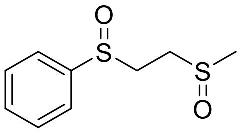

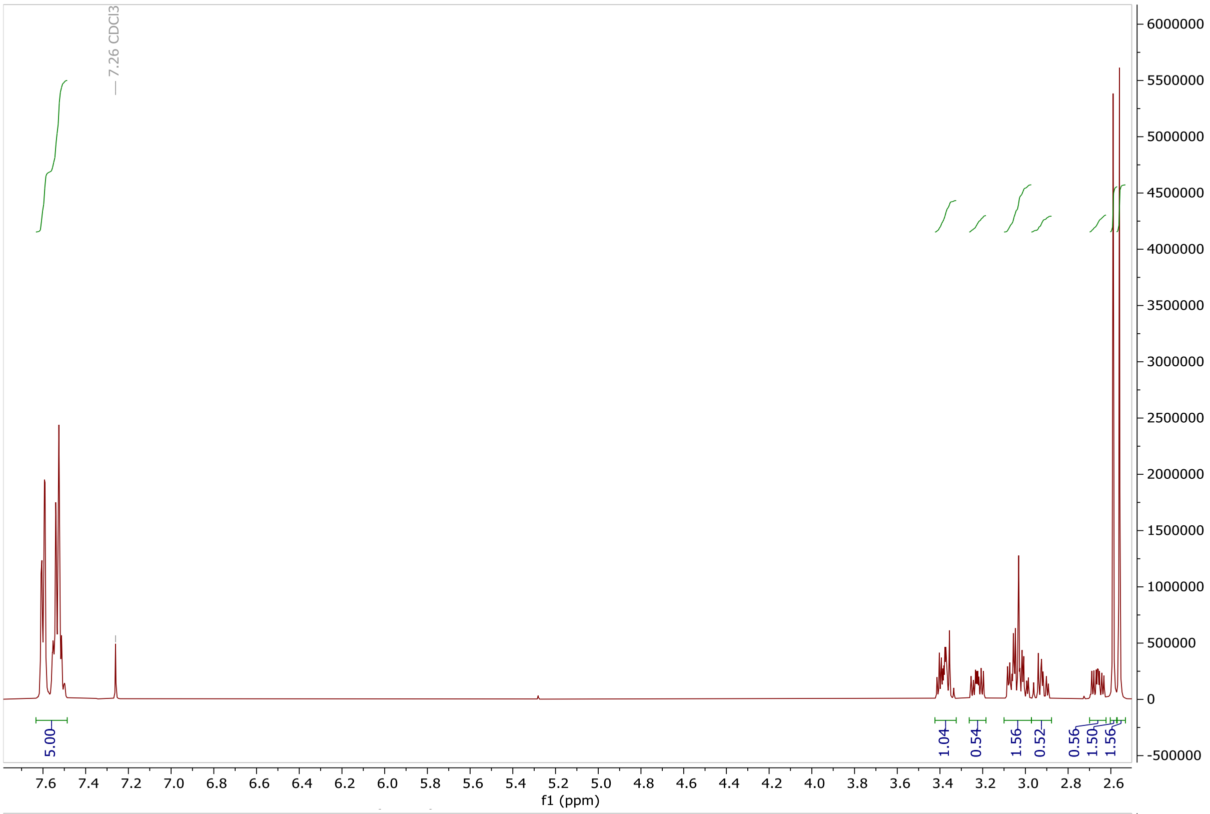

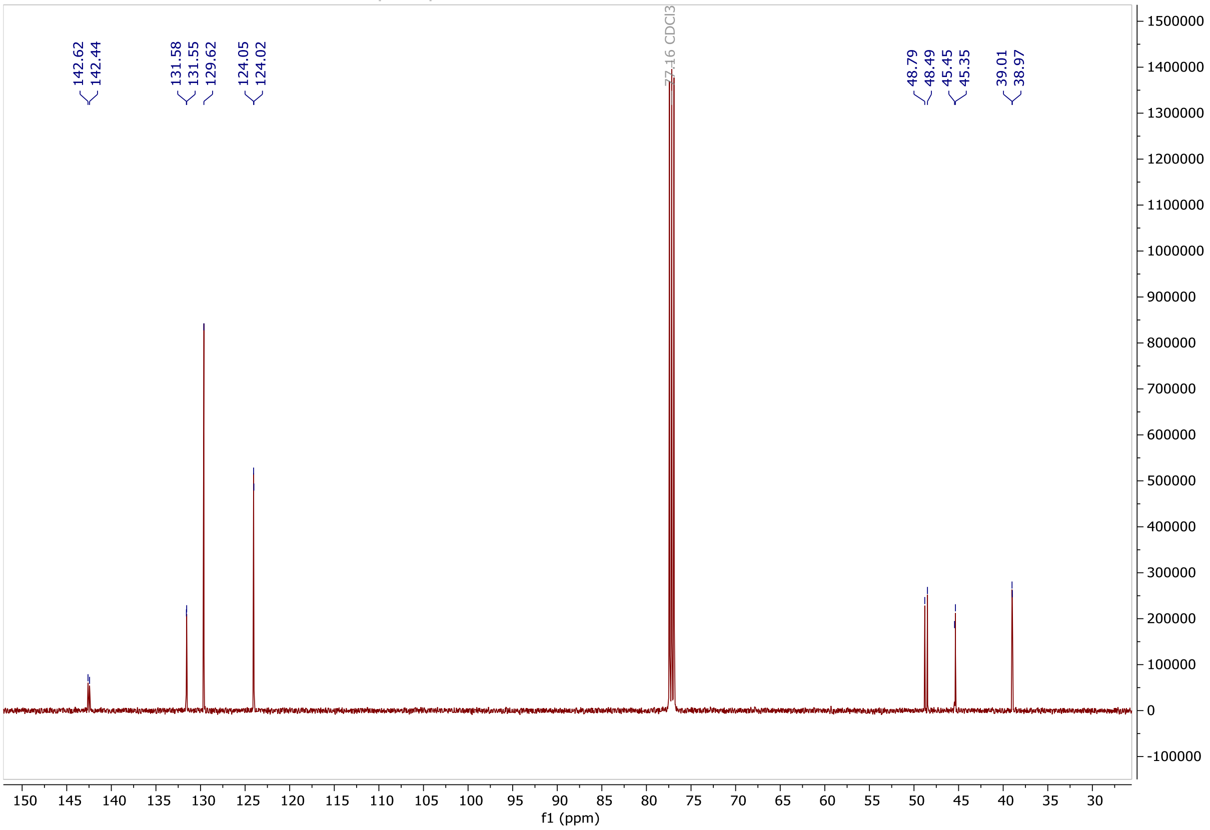

6.3.16 (Ethylsulfinyl)benzene (1s)

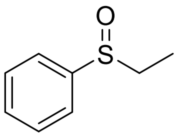

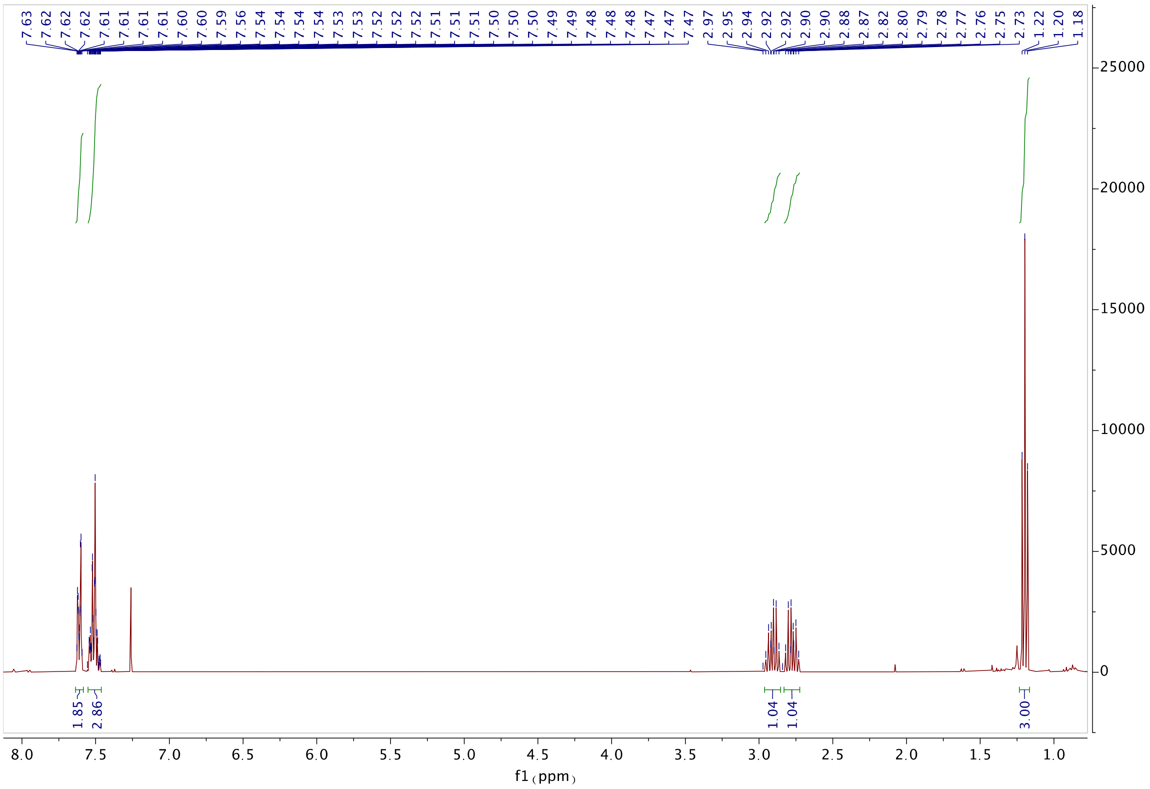

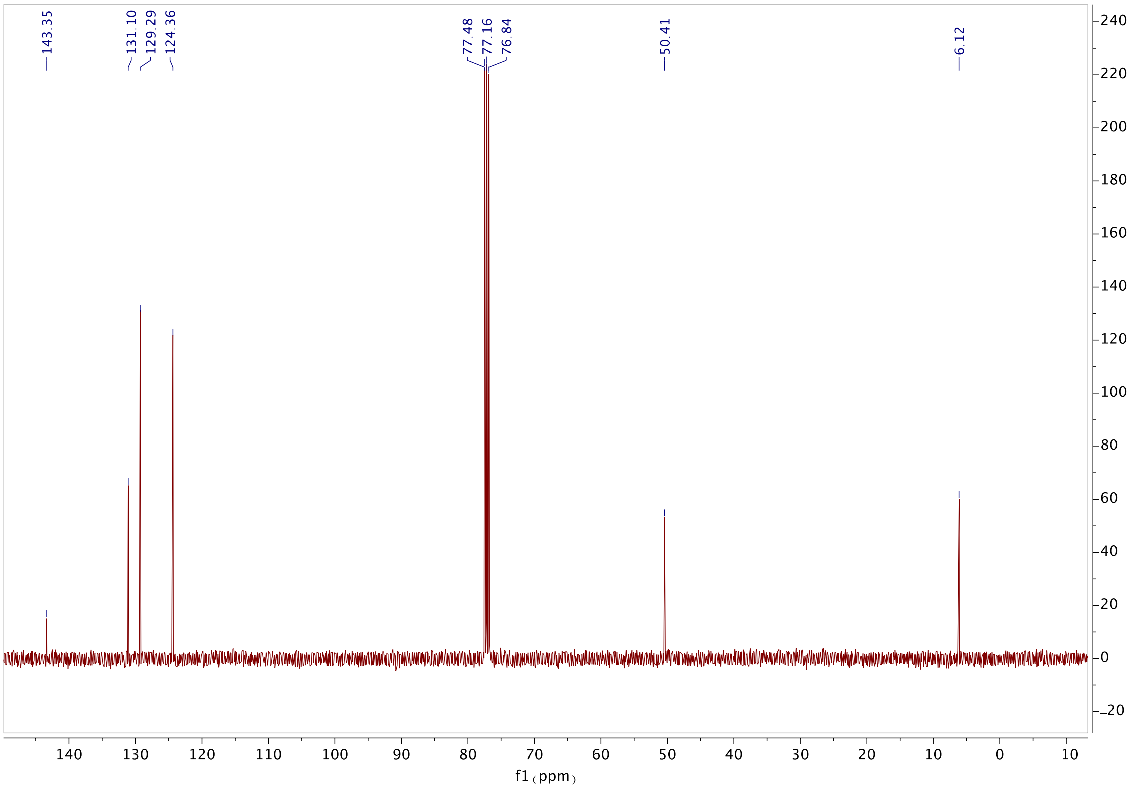

6.3.17 1-(Ethylsulfinyl)-4-methylbenzene (1t)

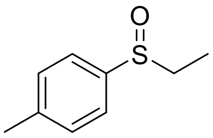

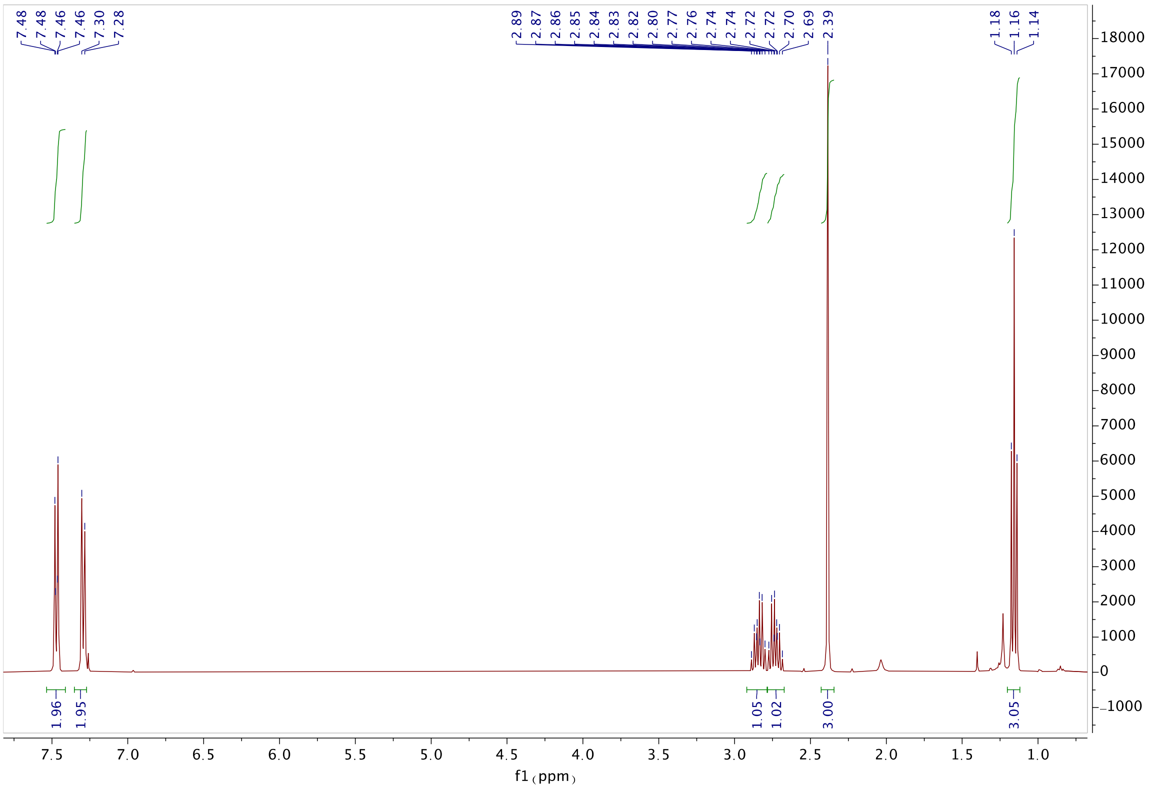

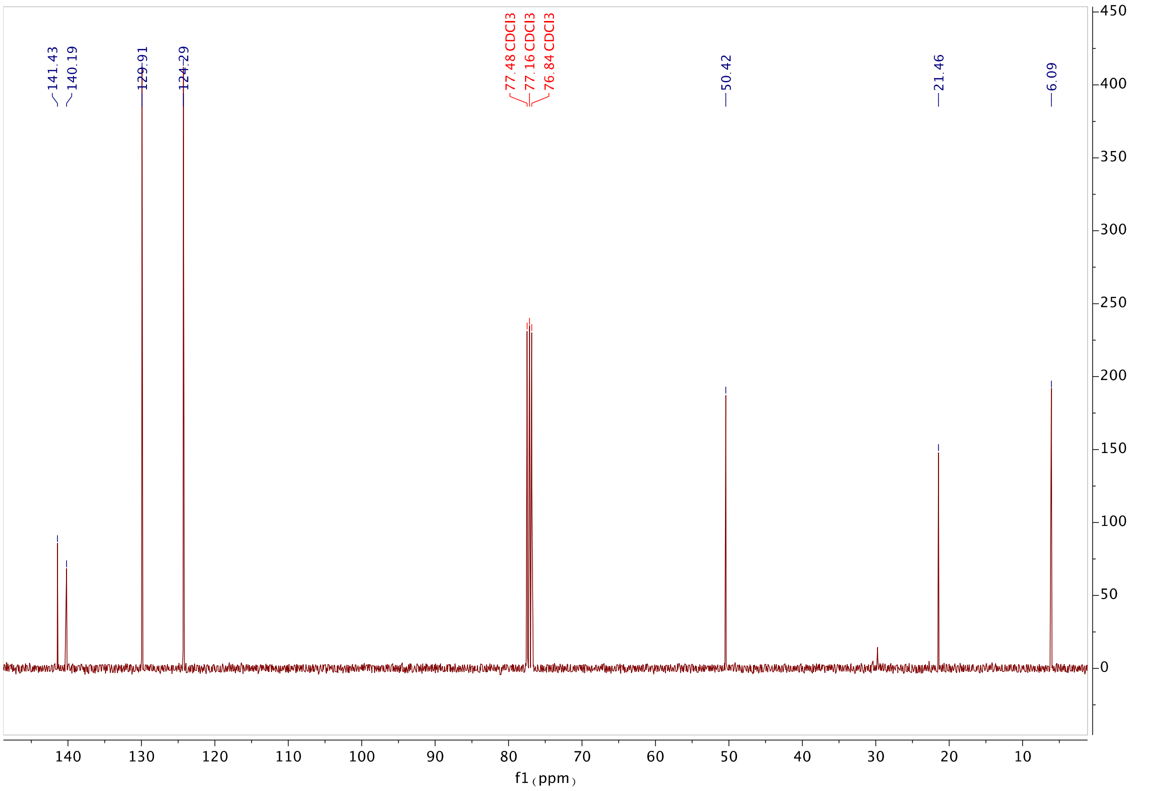

6.3.18 1-Bromo-4-(ethylsulfinyl)benzene (1u)

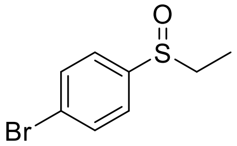

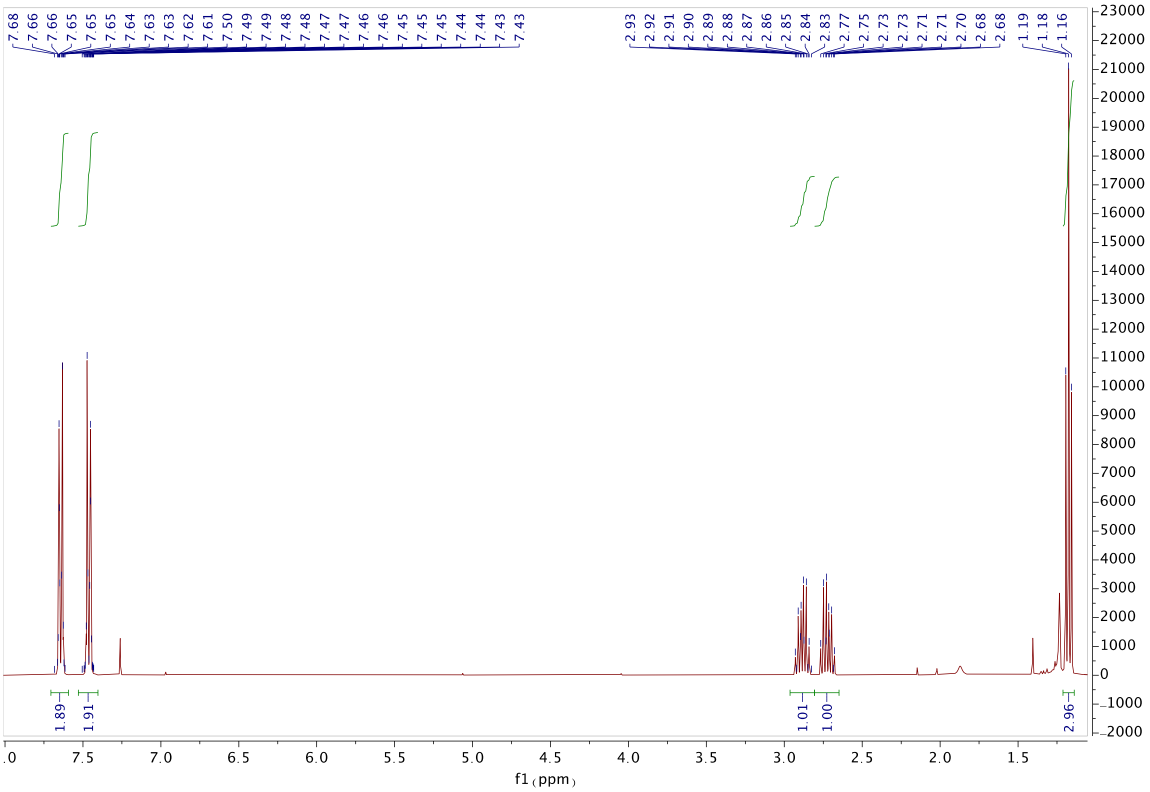

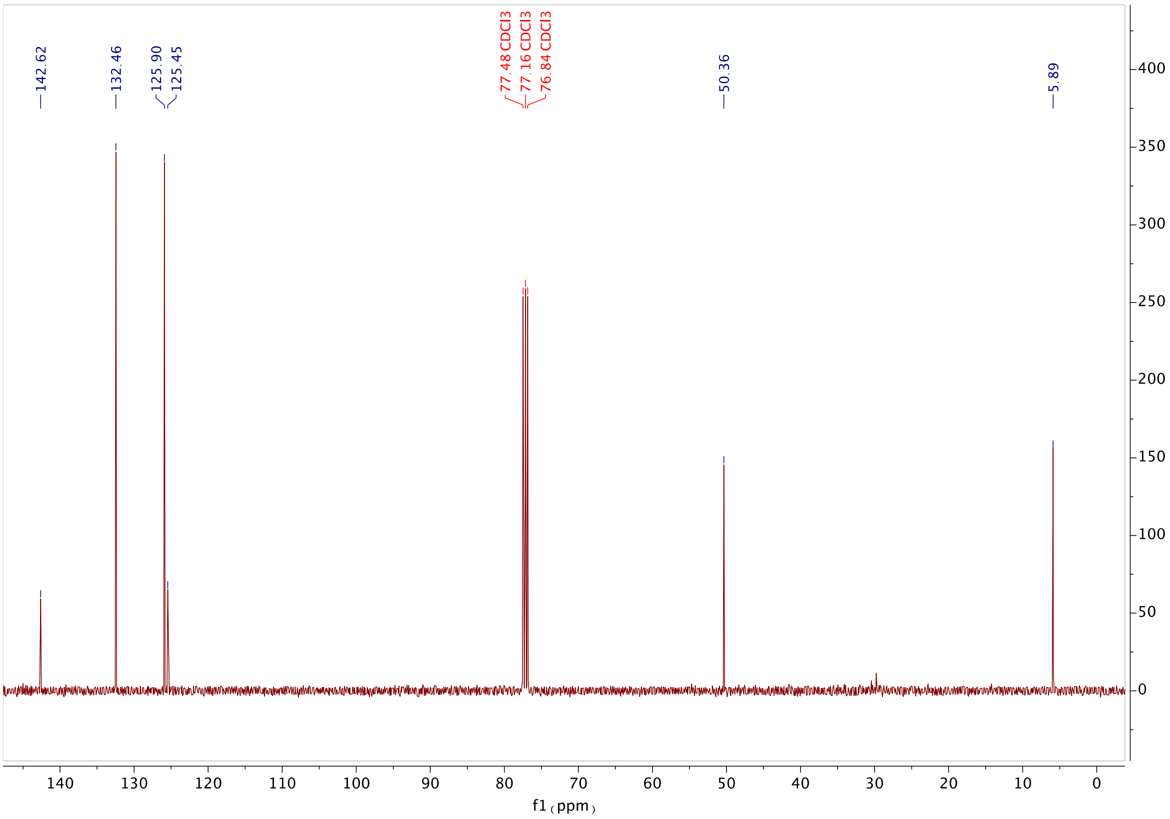

6.3.19 ((Ethylsulfinyl)methyl)benzene (1v)

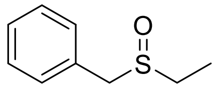

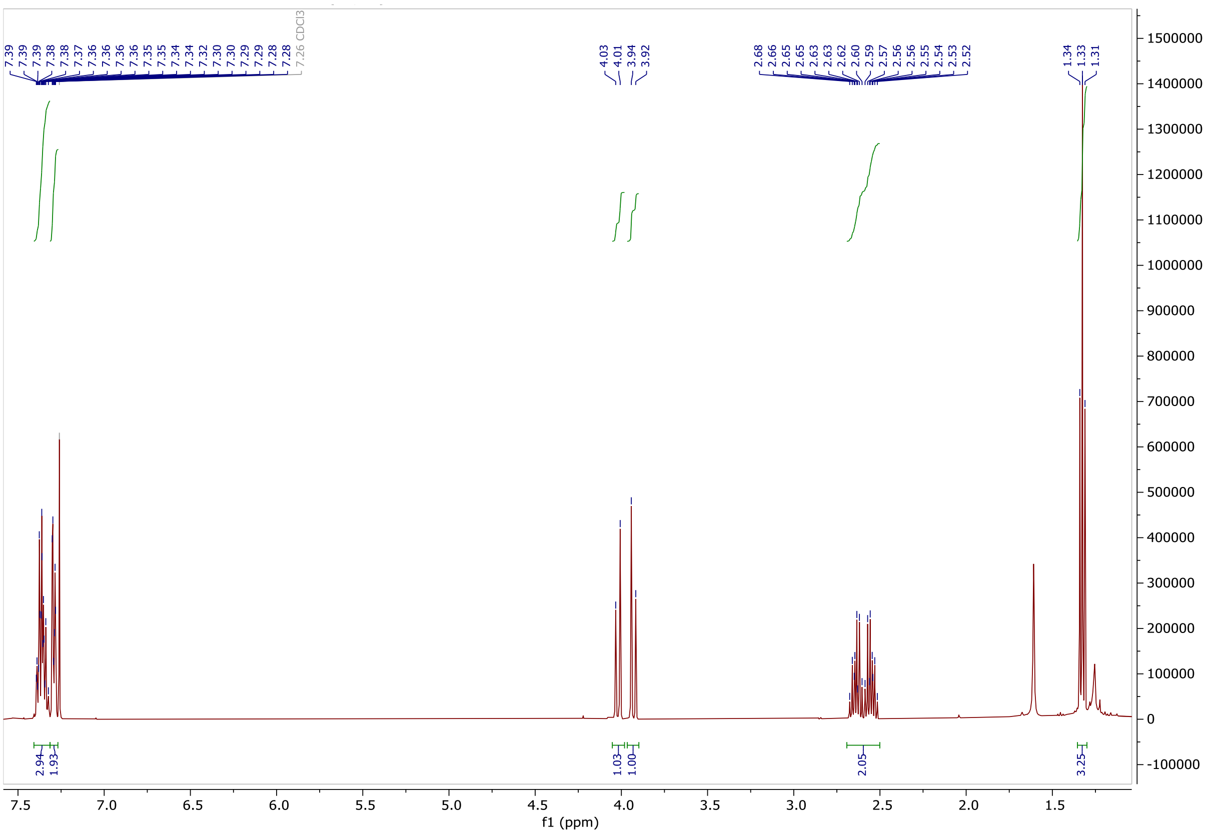

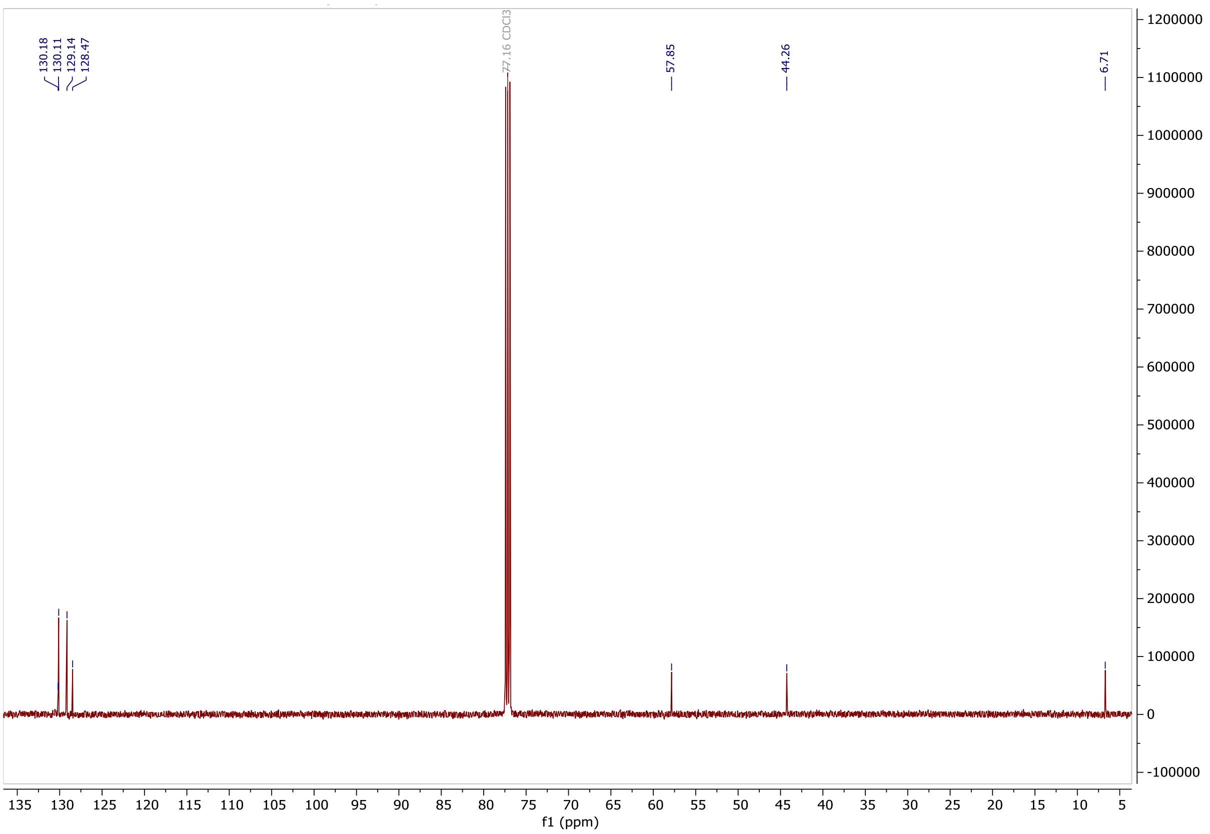

6.3.20 (2-(Ethylsulfinyl)ethyl)benzene (1w)

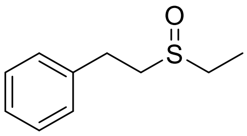

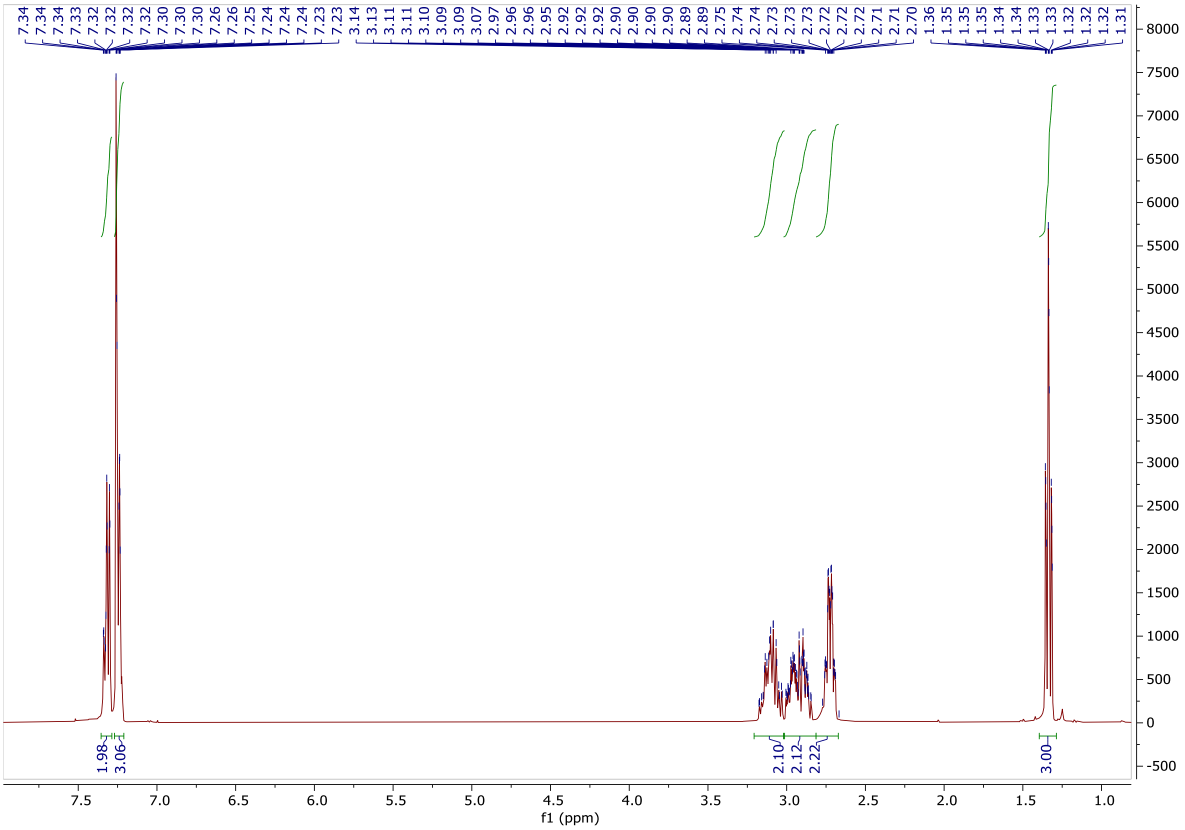

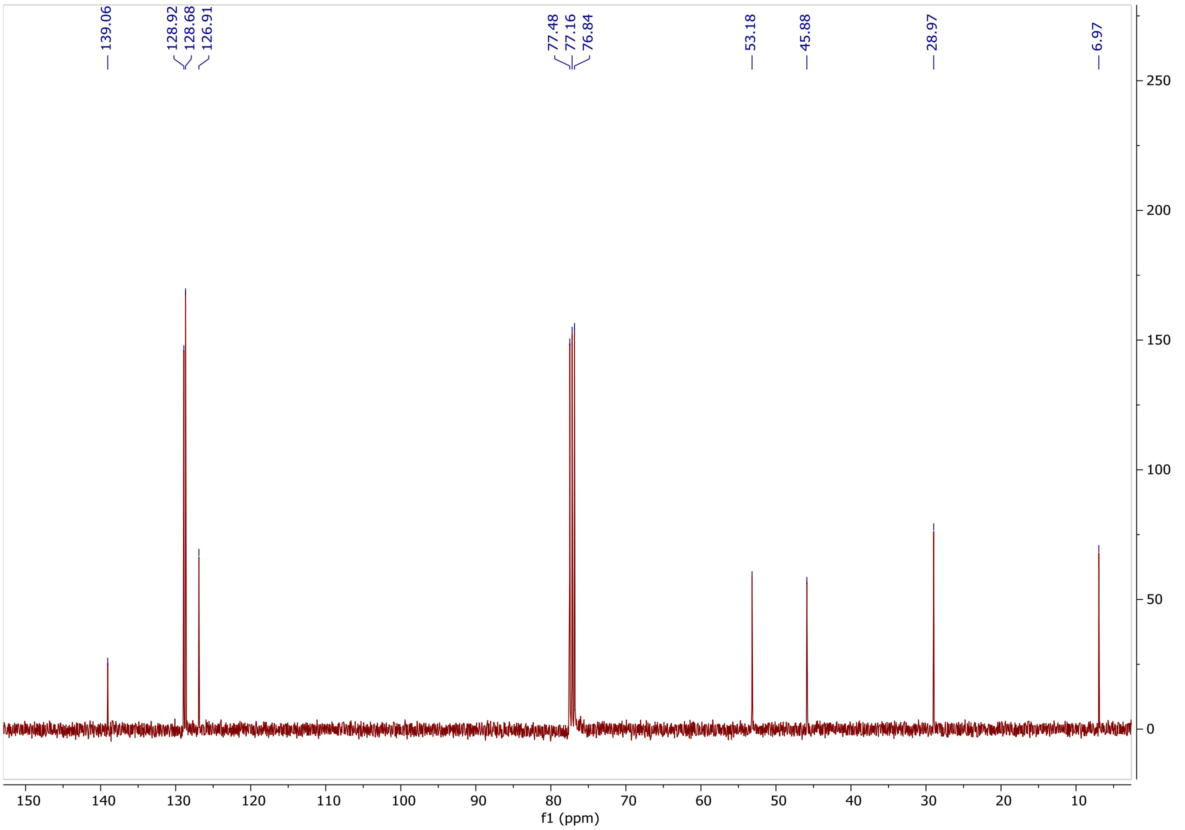

6.3.21 2-((Ethylsulfinyl)methyl)pyridine (1x)

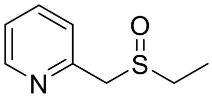

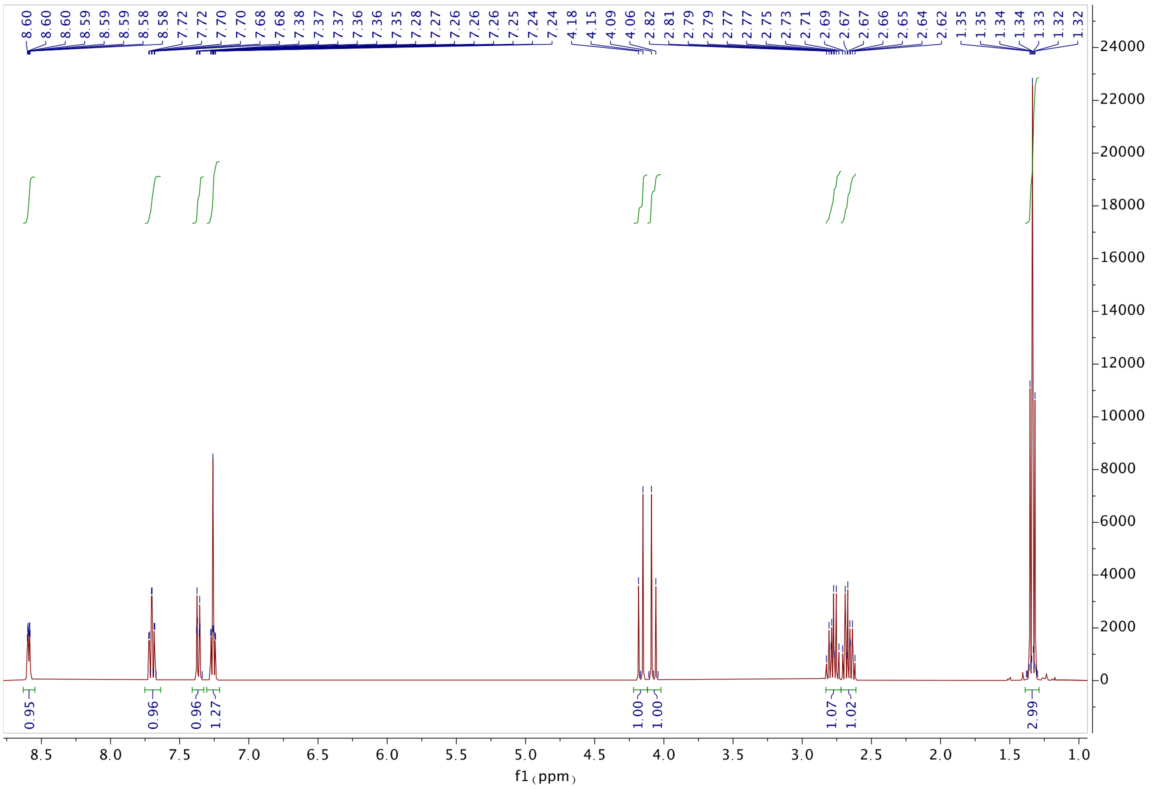

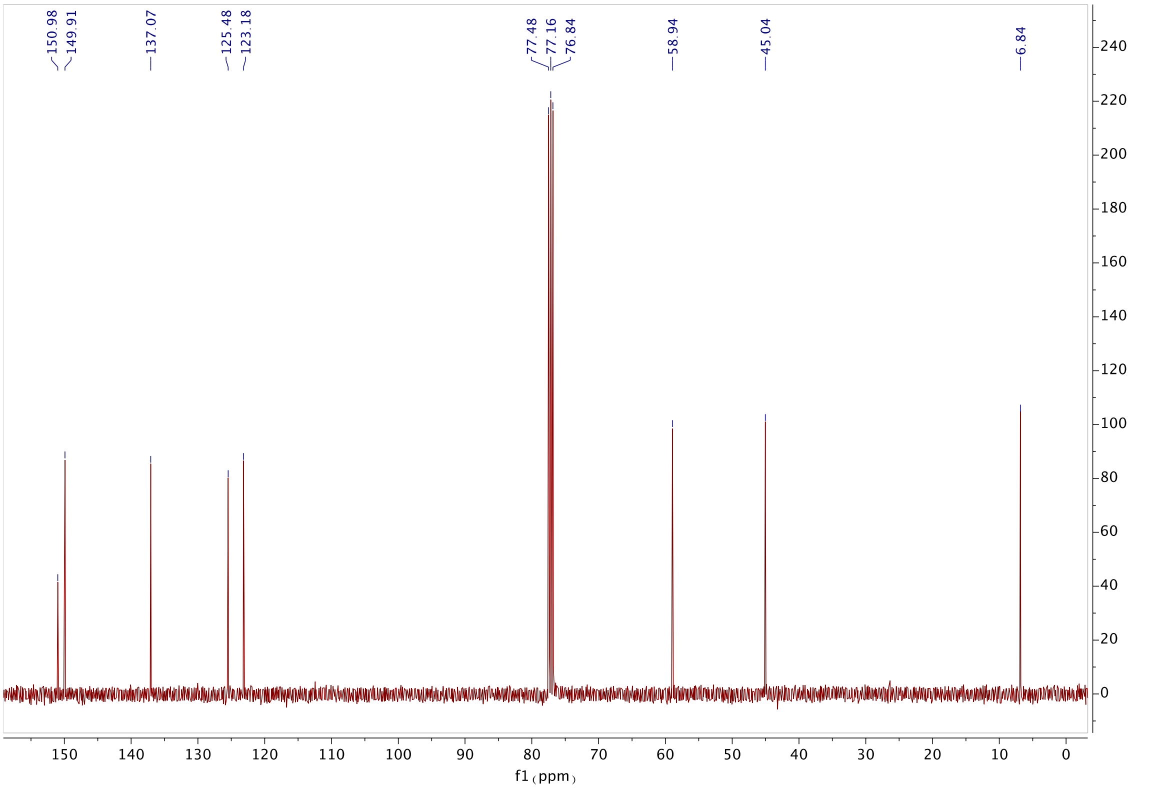

6.3.22 (Propylsulfinyl)benzene (1y)

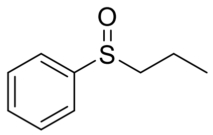

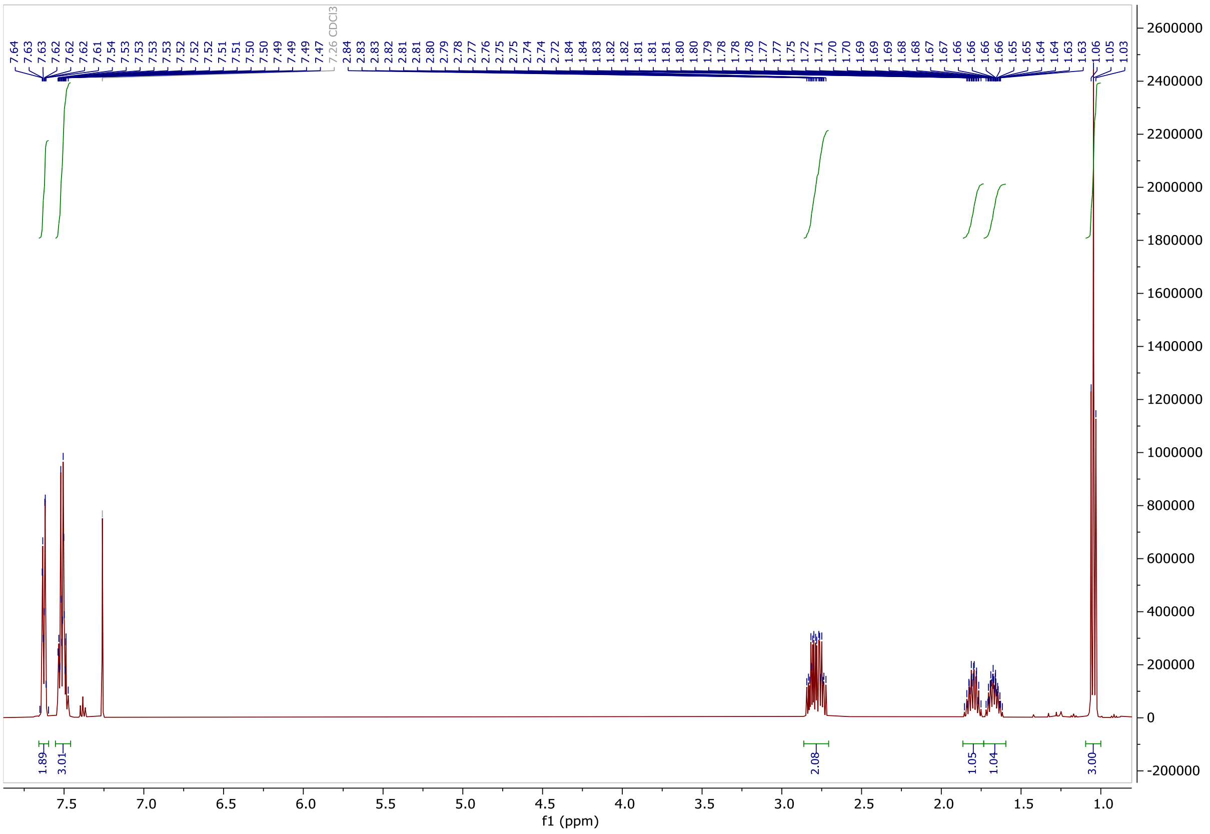

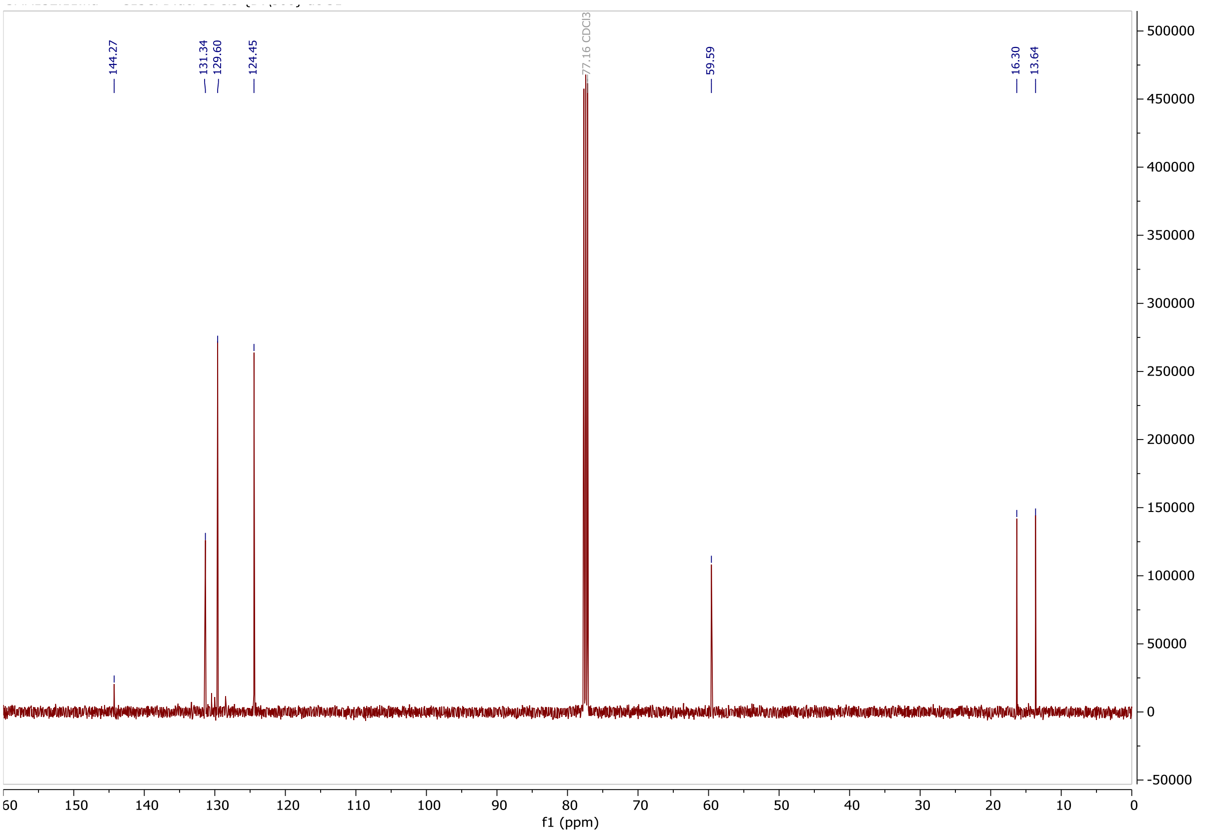

6.3.23 1-Methoxy-4-(propylsulfinyl)benzene (1z)

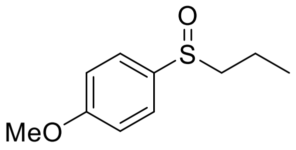

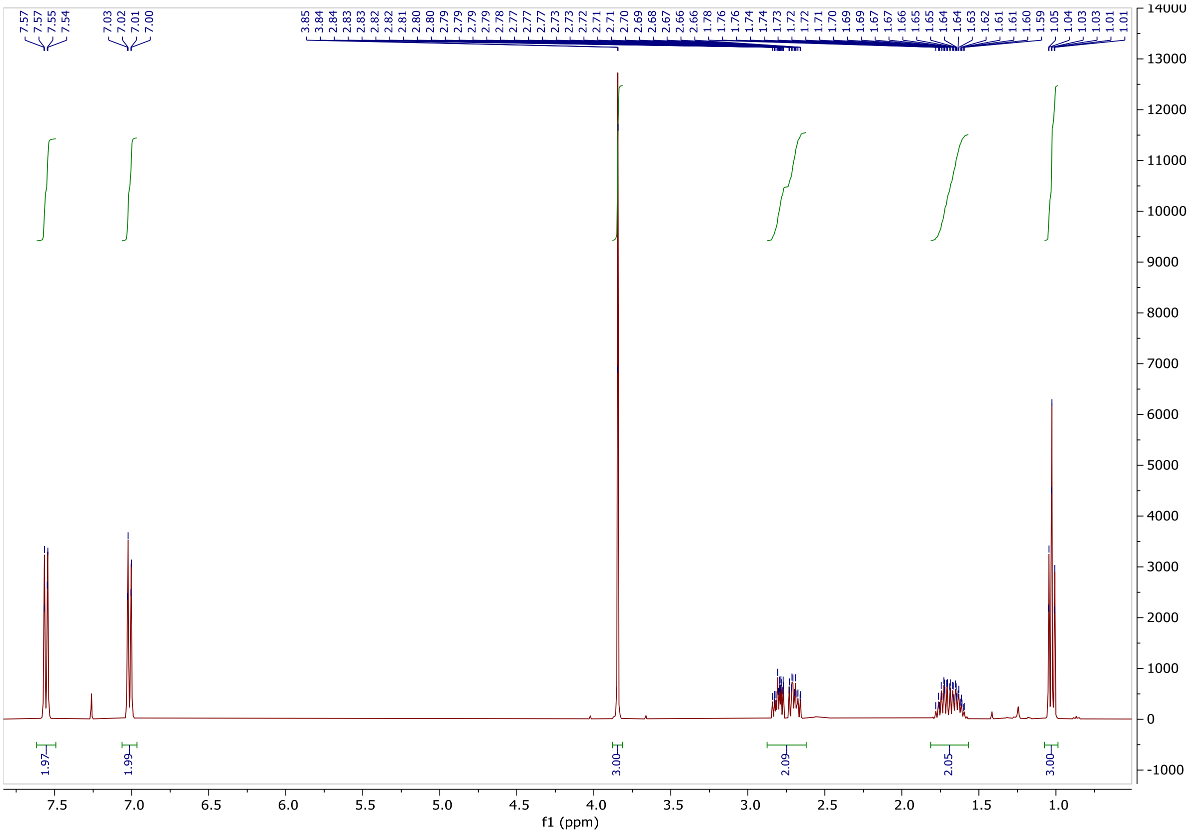

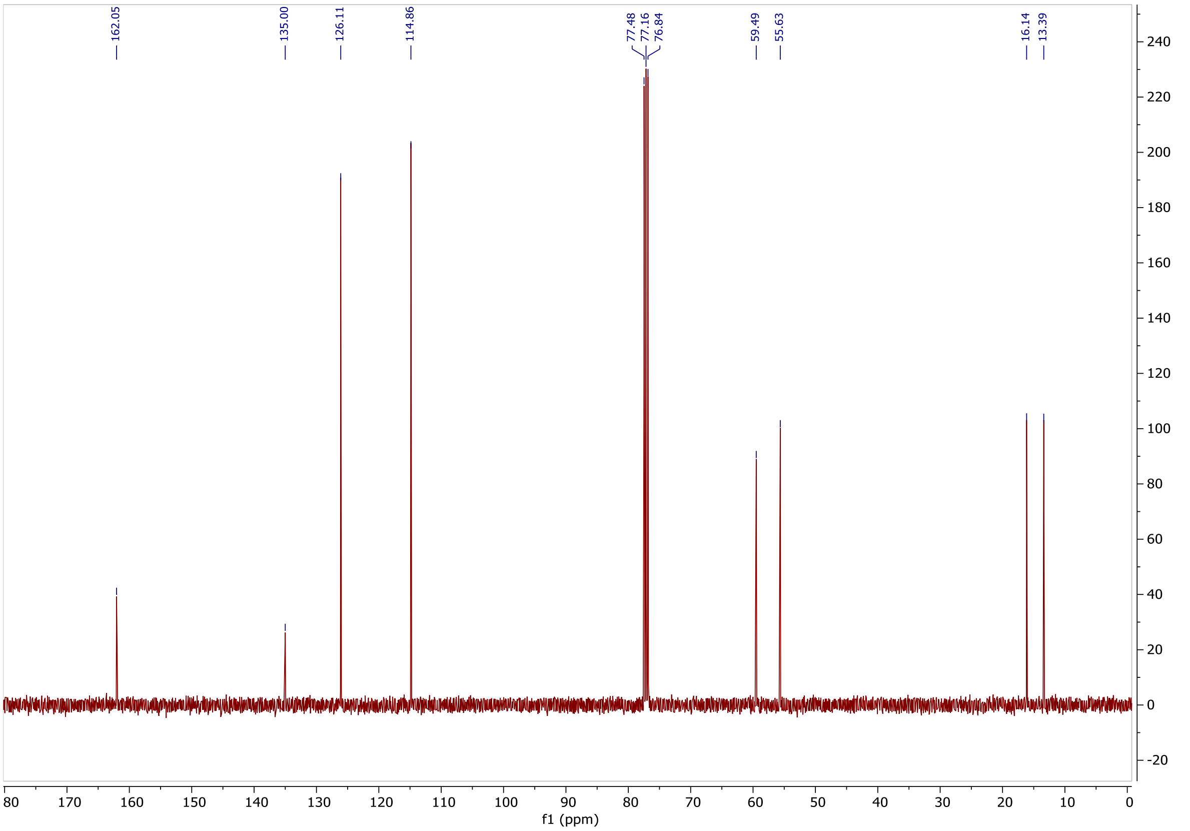

6.3.24 (Allylsulfinyl)benzene (1ab)

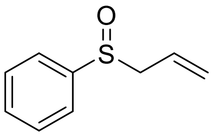

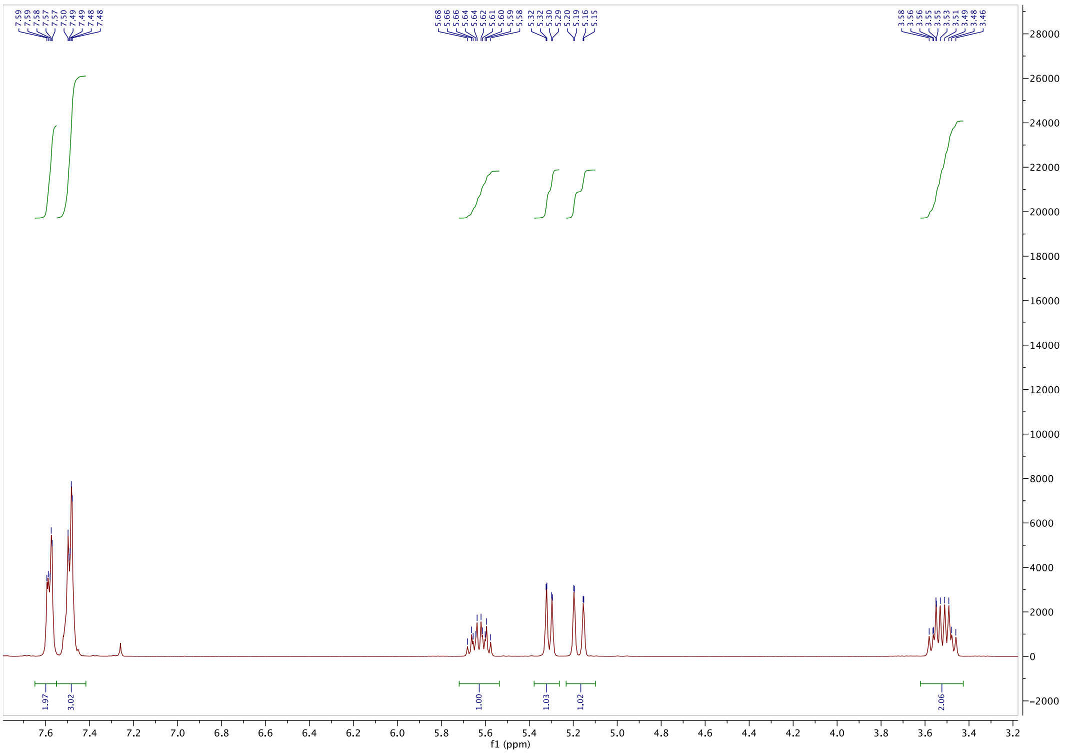

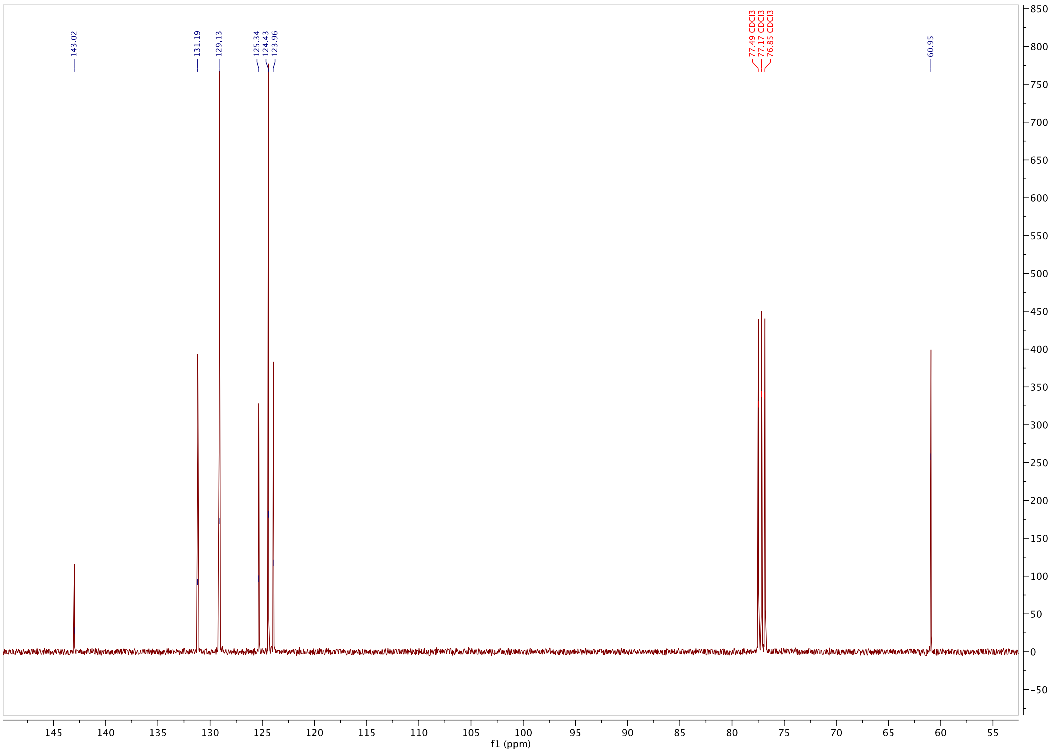

6.3.25 1-Methyl-4-(propylsulfinyl)benzene (1ac)

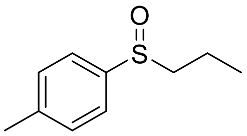

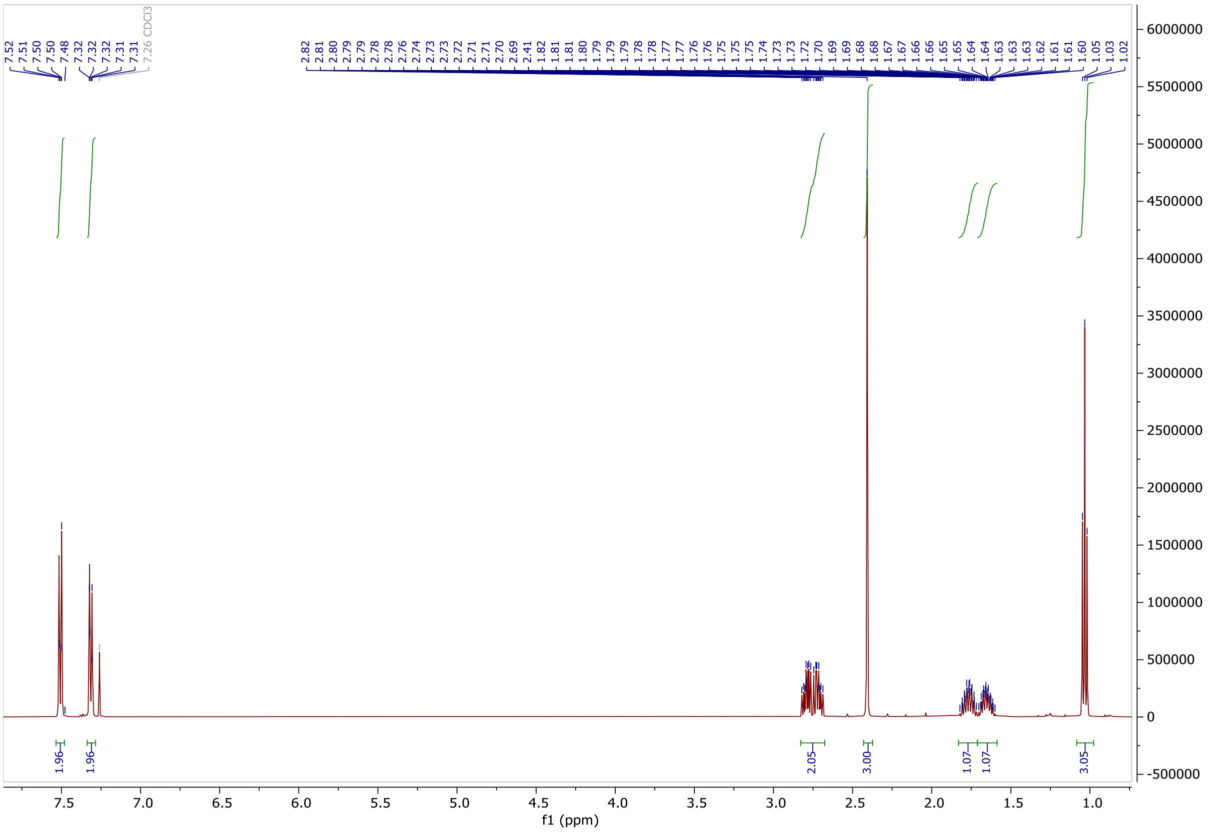

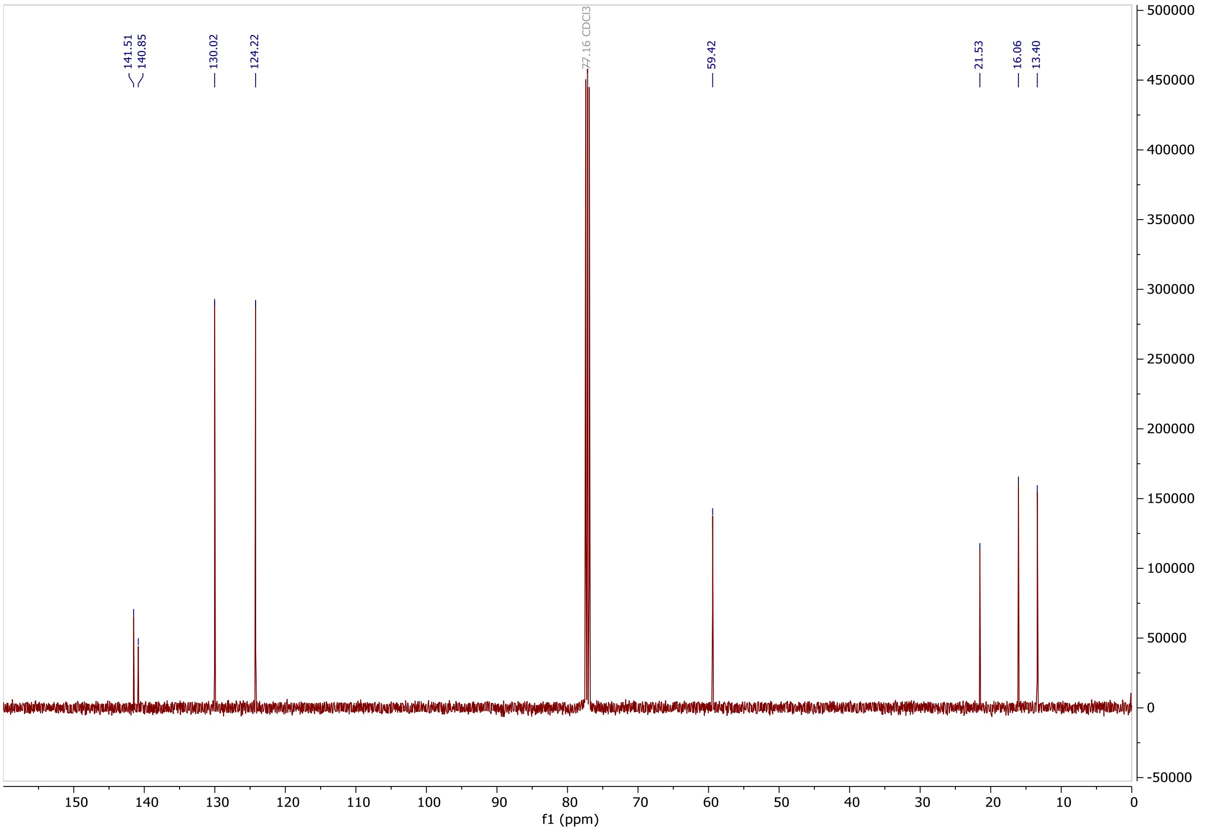

6.3.26 1-Chloro-4-(propylsulfinyl)benzene (1ad)

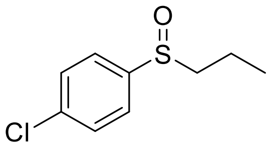

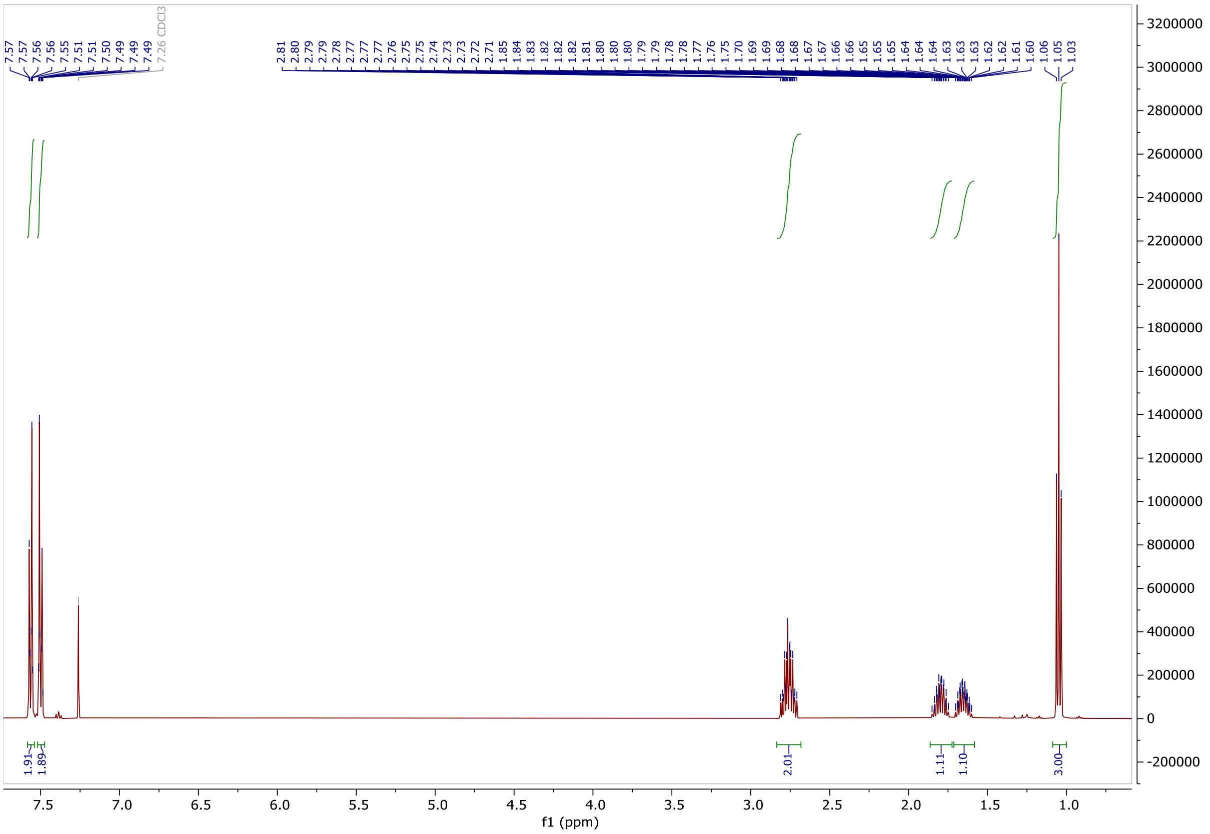

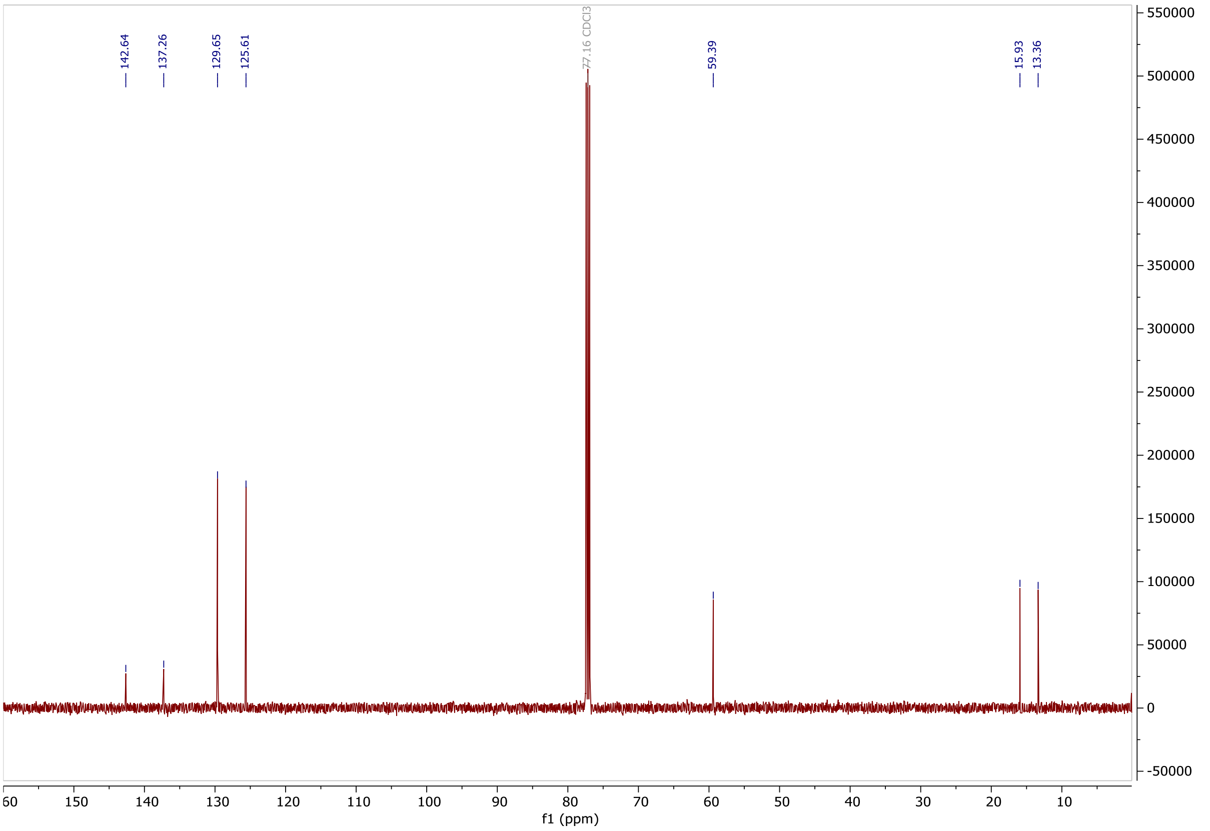

6.3.27 1-Bromo-4-(propylsulfinyl)benzene (1ae)

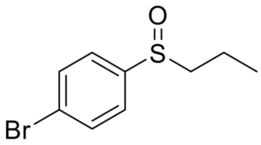

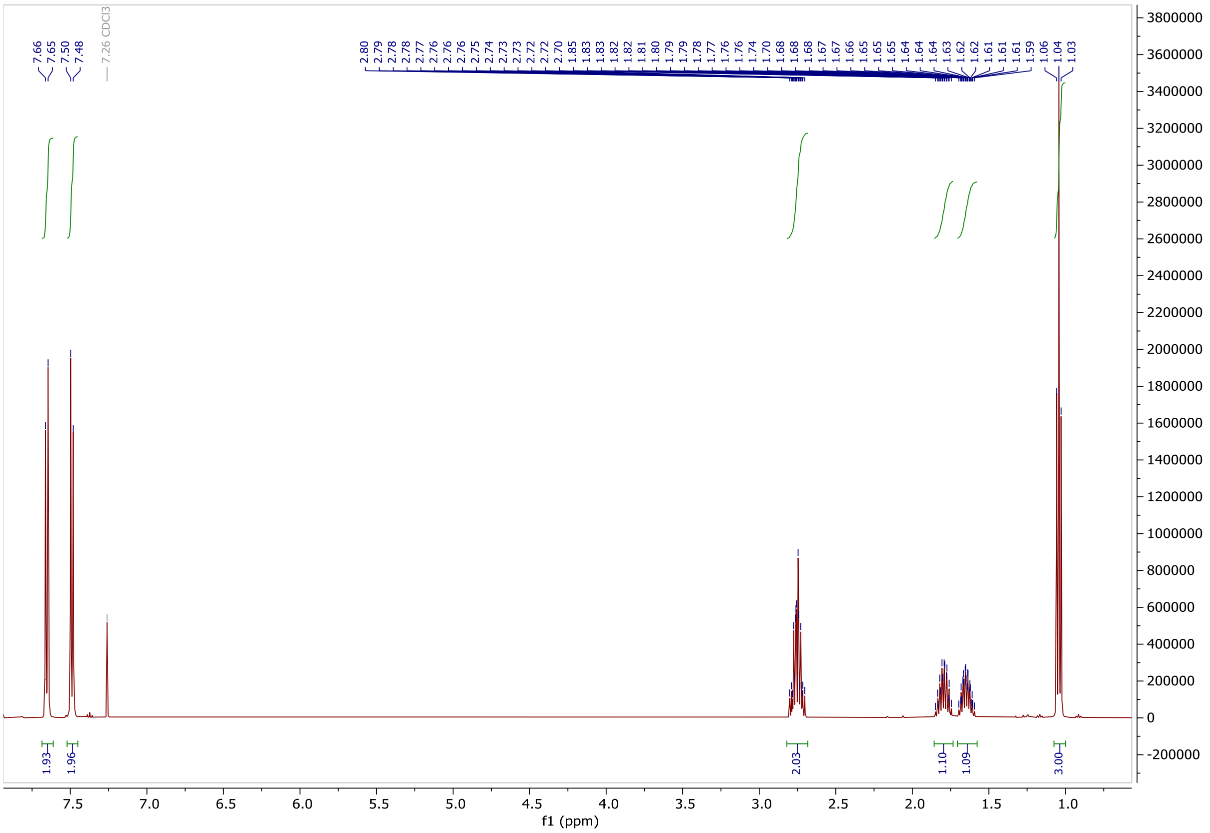

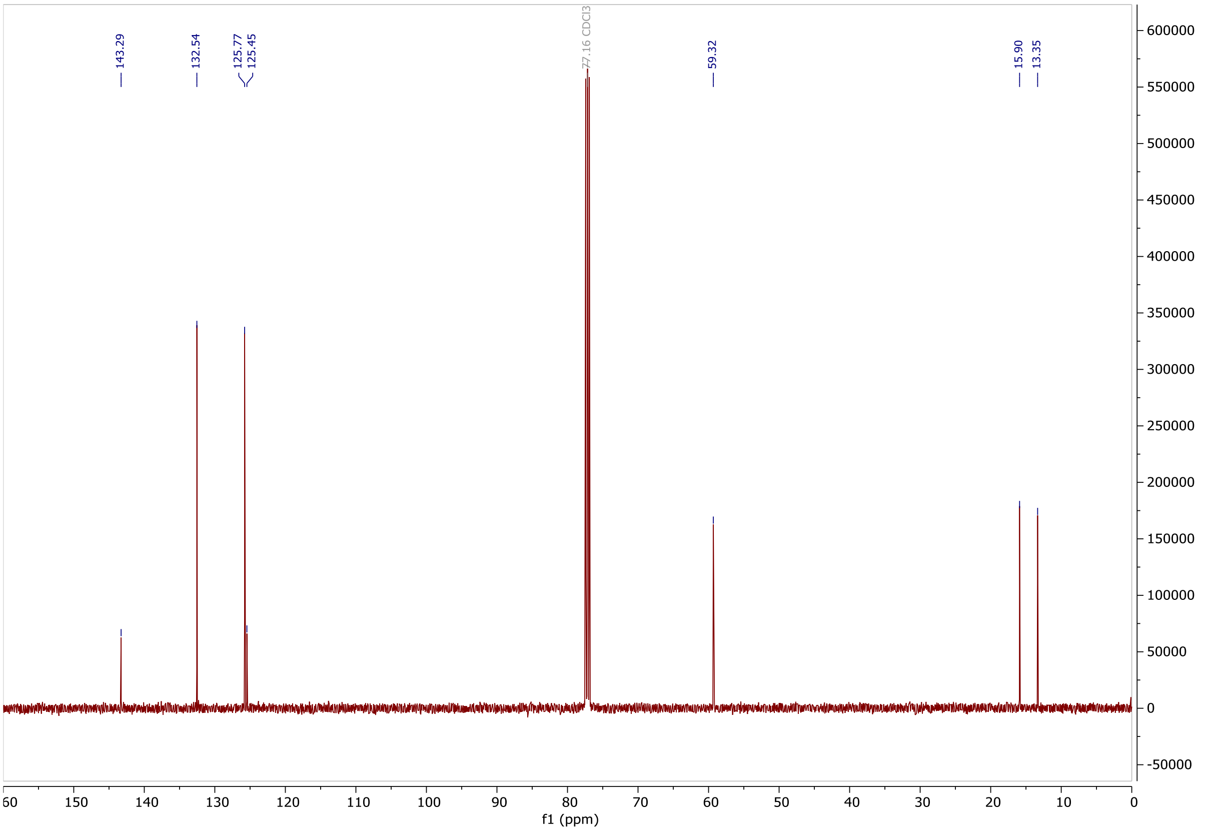

6.3.28 1-Methoxy-3-(propylsulfinyl)benzene (1af)

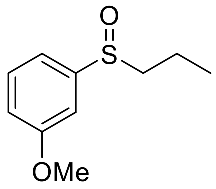

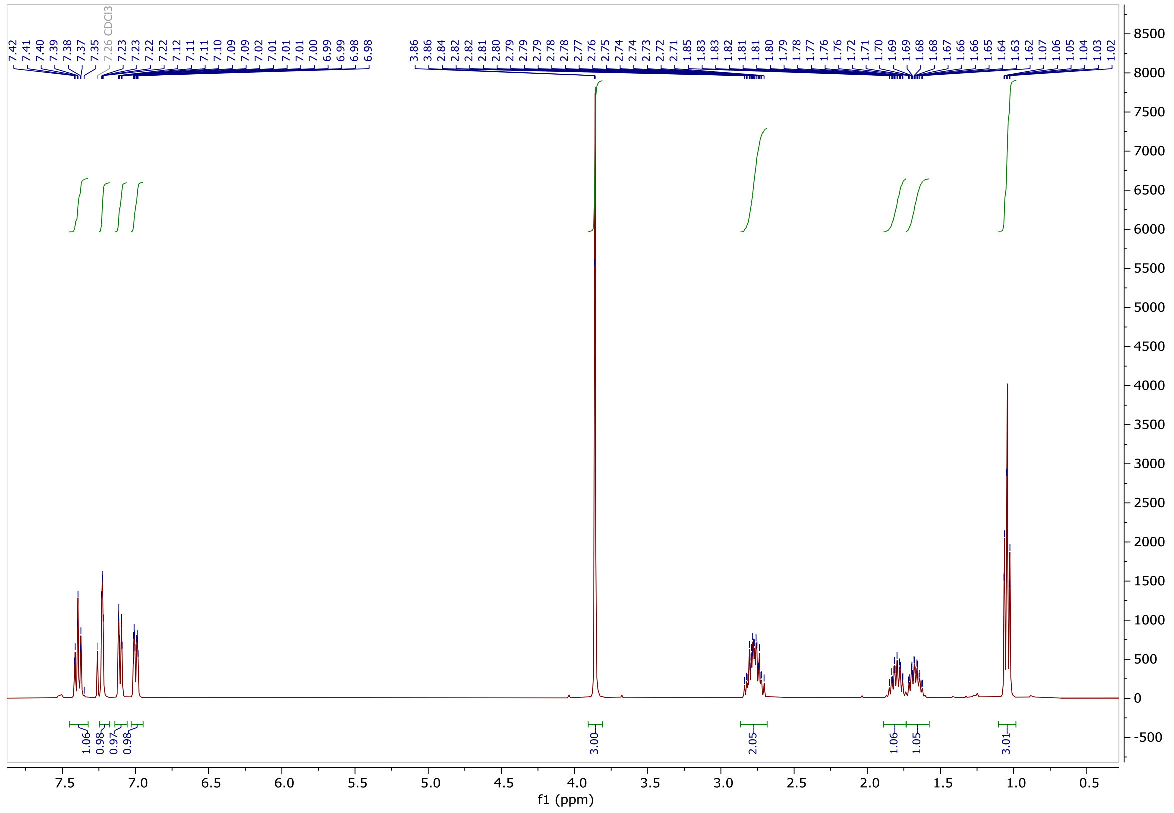

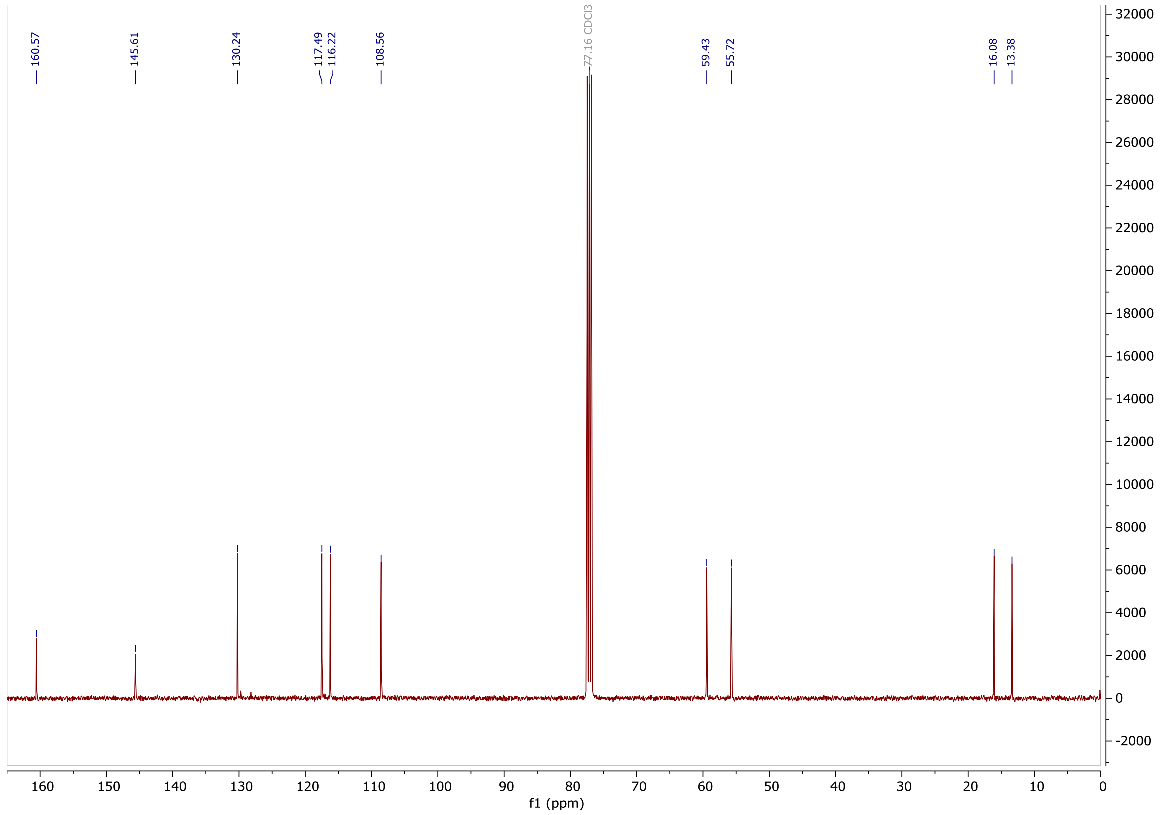

6.3.29 1-Methoxy-4-(butylsulfinyl)benzene (1ag)

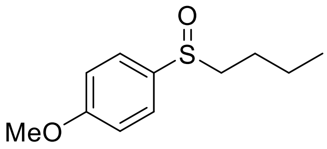

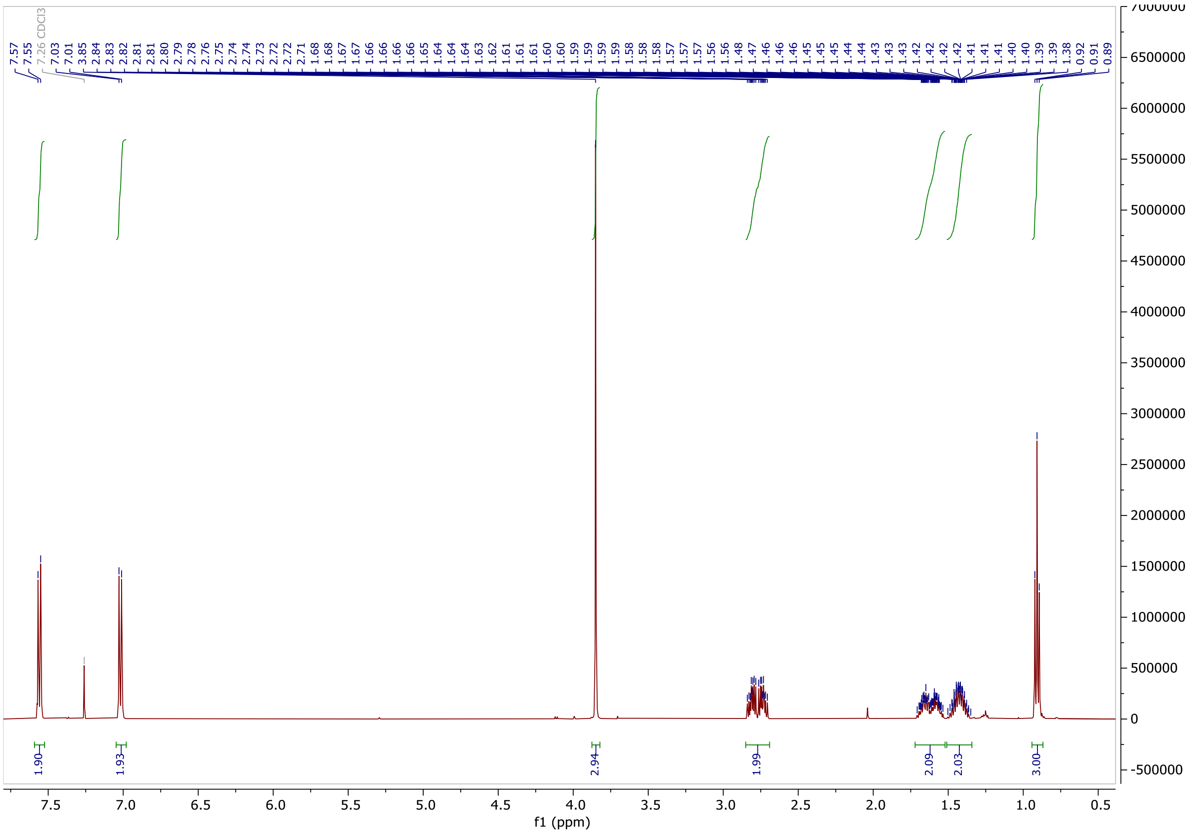

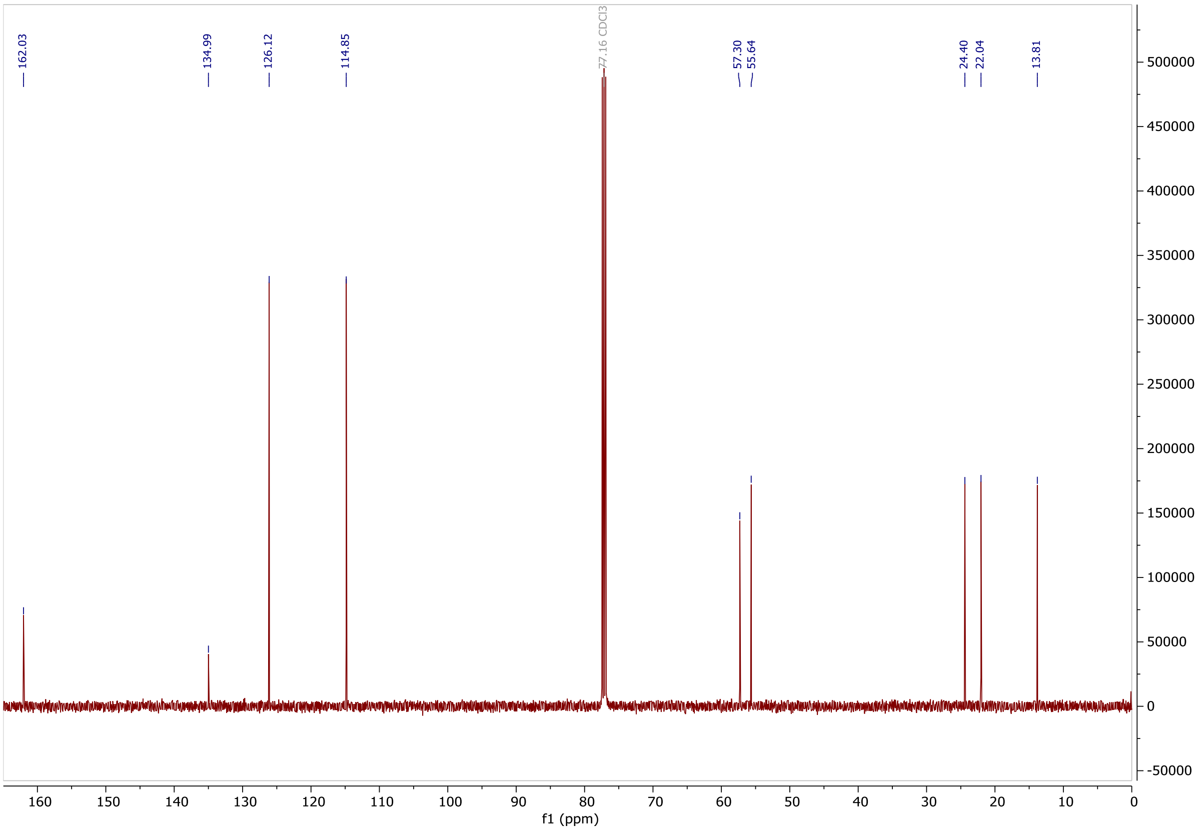

6.3.30 Methyl(2-(phenylsulfinyl)ethyl)sulfane (2r)

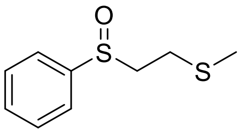

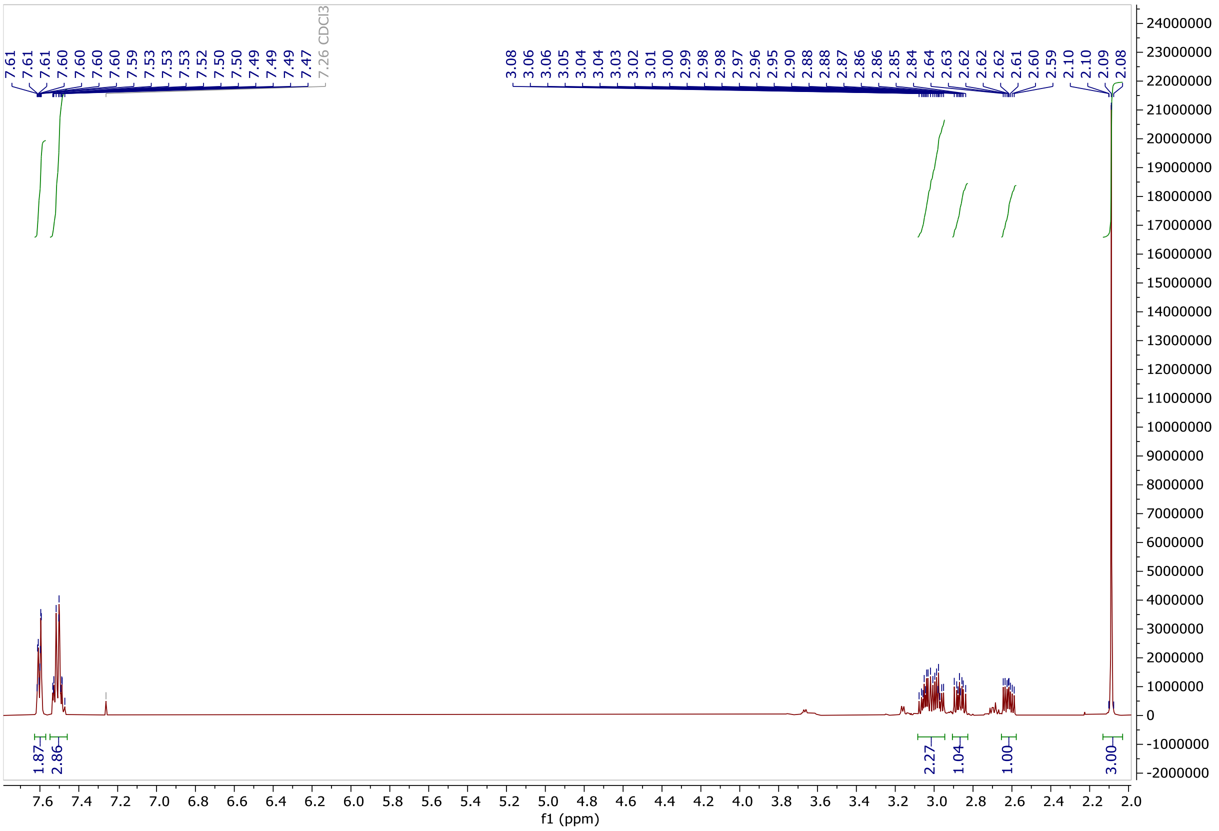

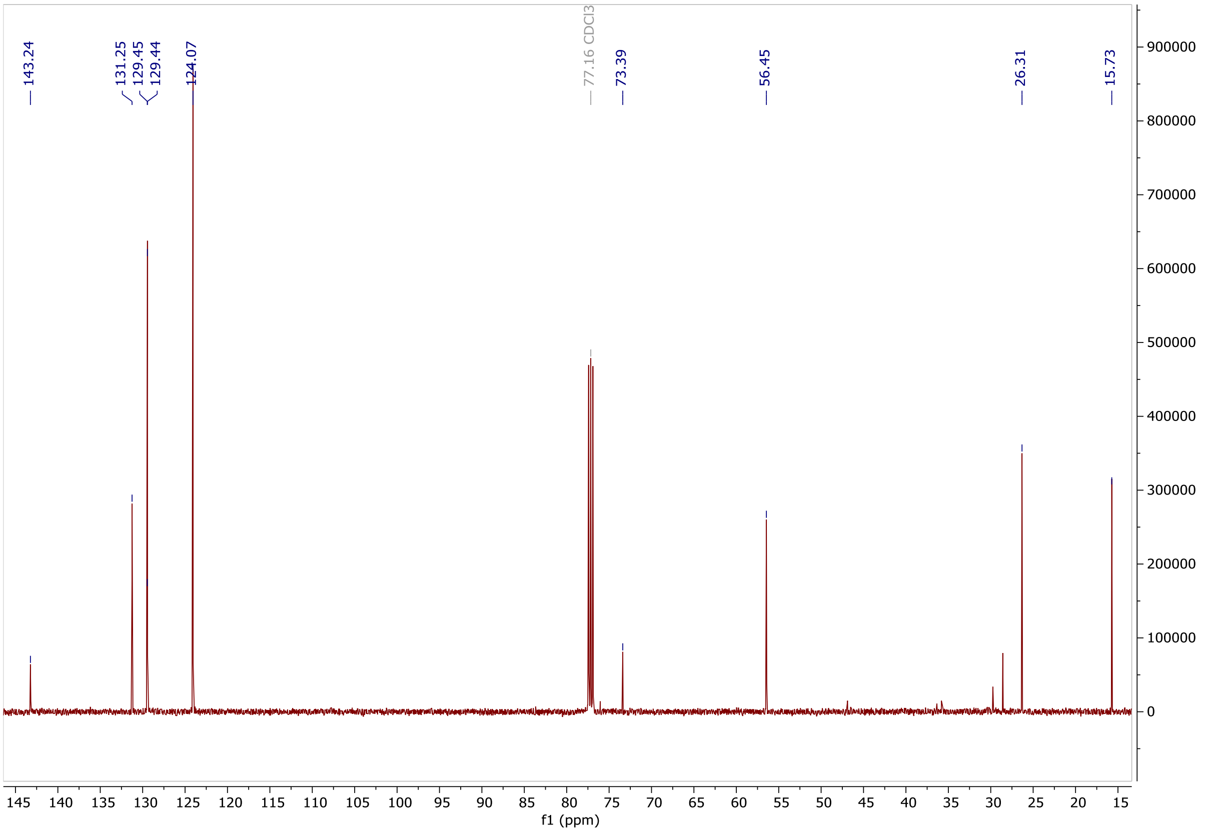

6.4 HPLC analysis and traces

In order to determine the enantiomeric excess of the sulfoxides, Daicel Chiralpak chiral columns IC (0.46 cm x 25 cm), IG (0.46 cm x 25 cm), ID (0.46 cm x 25 cm) and Daicel Chiracel OD-H (0.46 cm x 25 cm) were used in normal phase. All the methods described ran at 1.0 mLmin^{-1}, 25 ^{o}C with an isocratic eluent. Detection wavelengths were set at 254 and 240 nm for all compounds and 210 nm for compounds **1p** and **1q**.

Compound	Column	Eluent system	Retention time
**1a**	IG	*n*-hexane:IPA 8:2	14.2 (R), 15.2 (S)
**1b**	IC	*n*-hexane:IPA 8:2	20.2 (R), 22.8 (S)
**1c**	IC	*n*-hexane:IPA 8:2	16.5 (R), 17. (S)
**1d**	IC	*n*-hexane:IPA 8:2	16.8 (R), 17.9 (S)
**1e**	IC	*n*-hexane:IPA 8:2	16.1 (R), 17.3 (S)
**1f**	IG	*n*-hexane:IPA 8:2	11.5 (R), 13.3 (S)
**1g**	IG	*n*-hexane:IPA 8:2	11.5 (S), 13.5 (R)
**1h**	IC	*n*-hexane:IPA 6:4	25.4 (S), 27.3 (R)
**1i**	IG	*n*-hexane:IPA 8:2	25.4 (R), 27.0 (S)
**1j**	IG	*n*-hexane:IPA 8:2	16.9 (R), 19.3 (S)
**1k**	IG	*n*-hexane:IPA 8:2	11.7 (S), 12.3 (R)
**1l**	IG	*n*-hexane:IPA 8:2	14.8 (R), 16.8 (S)
**1m**	IG	*n*-hexane:IPA 8:2	19.1 (R), 21.0 (S)
**1n**	IC	*n*-hexane:EtOH 8:2	13.6 (R), 15.7 (S)
**1o**	IC	*n*-hexane:IPA 7:3	16.7 (S), 17.5 (R)
**1p**	IC	*n*-hexane:IPA 9:1	30.5 (R), 32.3 (S)
**1q**	IG	*n*-hexane:IPA 6:4	8.4 (R), 9.0 (S)
**1r**	IC	*n*-hexane:EtOH 1:1	12.9, 14.0, 16.8, 20.5
**1s**	IC	*n*-hexane:IPA 8:2	17.2 (R), 20.0 (S)
**1t**	IG	*n*-hexane:IPA 8:2	14.4 (R), 15.7 (S)
**1u**	IC	*n*-hexane:IPA 8:2	14.6 (R), 15.5 (S)
**1v**	IC	*n*-hexane:IPA 8:2	21.3 (S), 23.5 (R)
**1w**	IG	*n*-hexane:IPA 8:2	13.2 (R), 17.3 (S)
**1x**	IC	*n*-hexane:IPA 6:4	15.2 (R), 16.9 (S)
**1y**	OD-H	*n*-hexane:EtOH 9:1	6.9 (R), 7.6 (S)
**1z**	IC	*n*-hexane:IPA 8:2	29.3 (S), 31.2 (R)
**1aa**	IG	*n*-hexane:IPA 95:5	11.7 (R), 12.4 (S)
**1ab**	IC	*n*-hexane:IPA 8:2	15.8 (R), 17.0 (S)
**1ac**	IC	*n*-hexane:IPA 8:2	19.4 (S), 21.9 (R)
**1ad**	IG	*n*-hexane:EtOH 9:1	17.2 (S), 18.0 (R)
**1ae**	IG	*n*-hexane:IPA 8:2	11.4 (R), 12.0 (R)
**1af**	ID	*n*-hexane:IPA 9:1	14.7 (R), 15.9 (S)
**1ag**	IC	*n*-hexane:IPA 8:2	28.3 (S), 30.8 (R)

6.4.1 1-Methyl-4-(methylsulfinyl)benzene (1a)

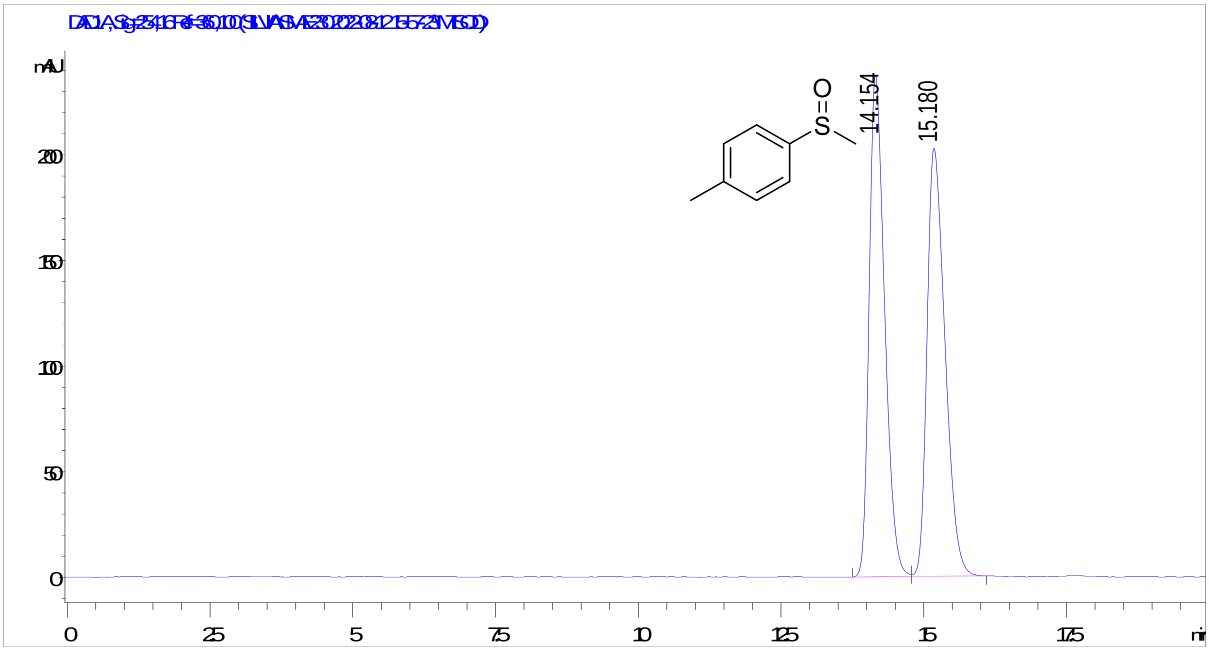

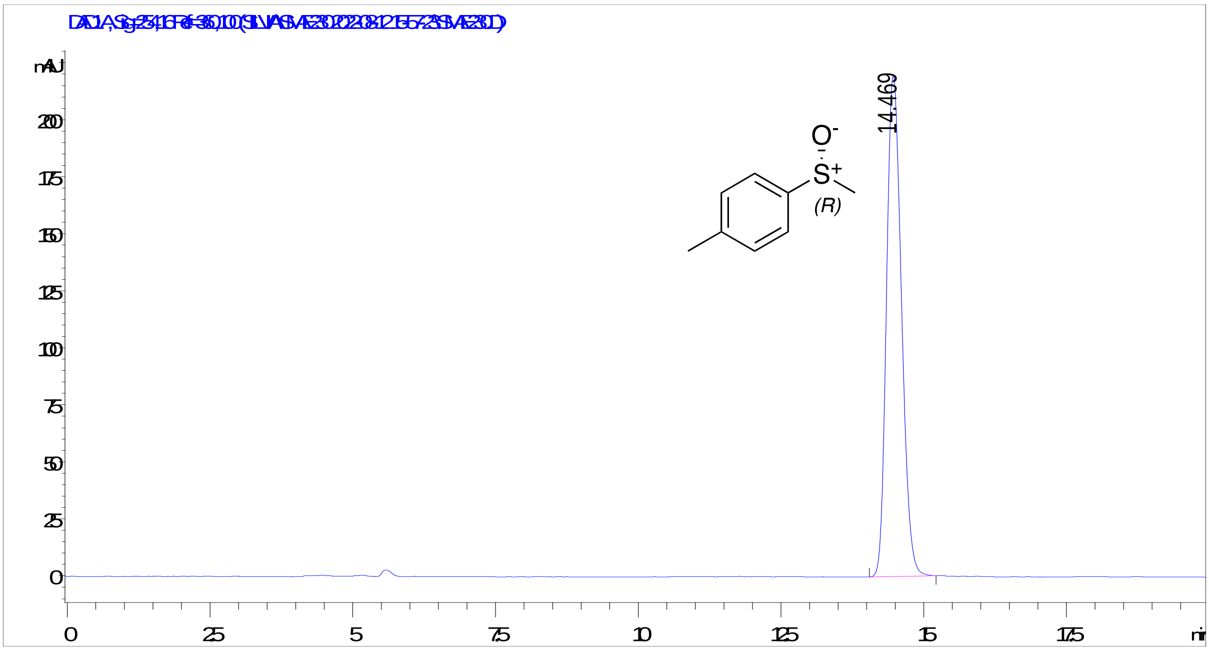

6.4.2 (Methylsulfinyl)benzene (1b)

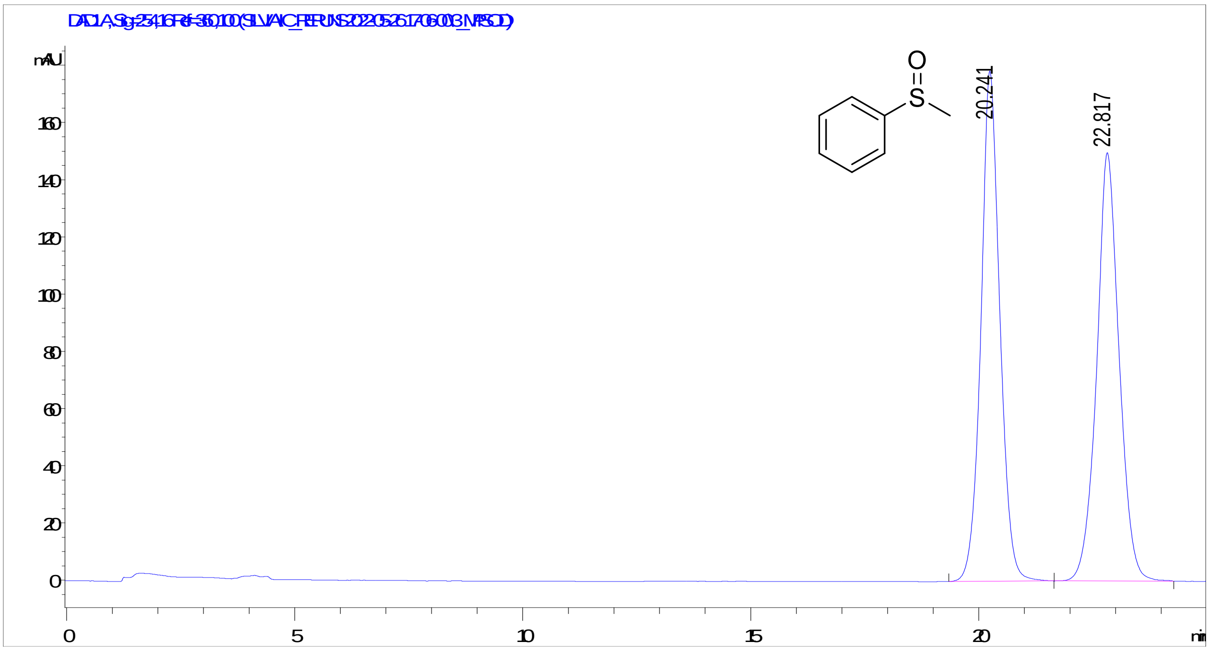

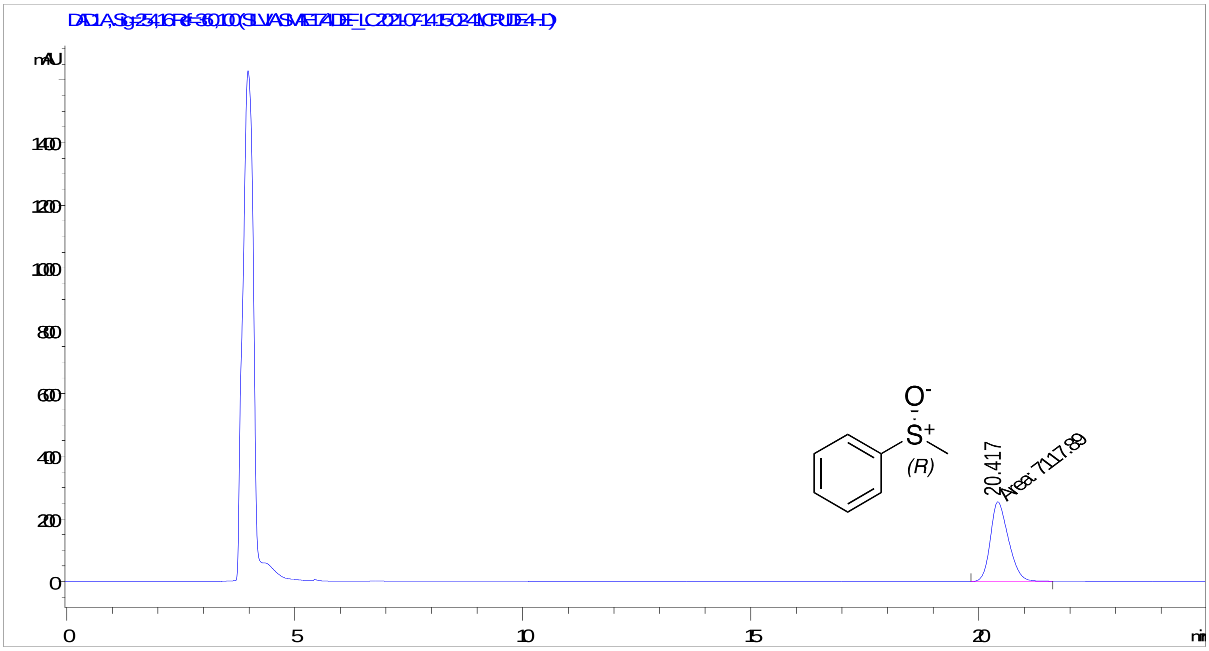

6.4.3 1-Fluoro-4-(methylsulfinyl)benzene (1c)

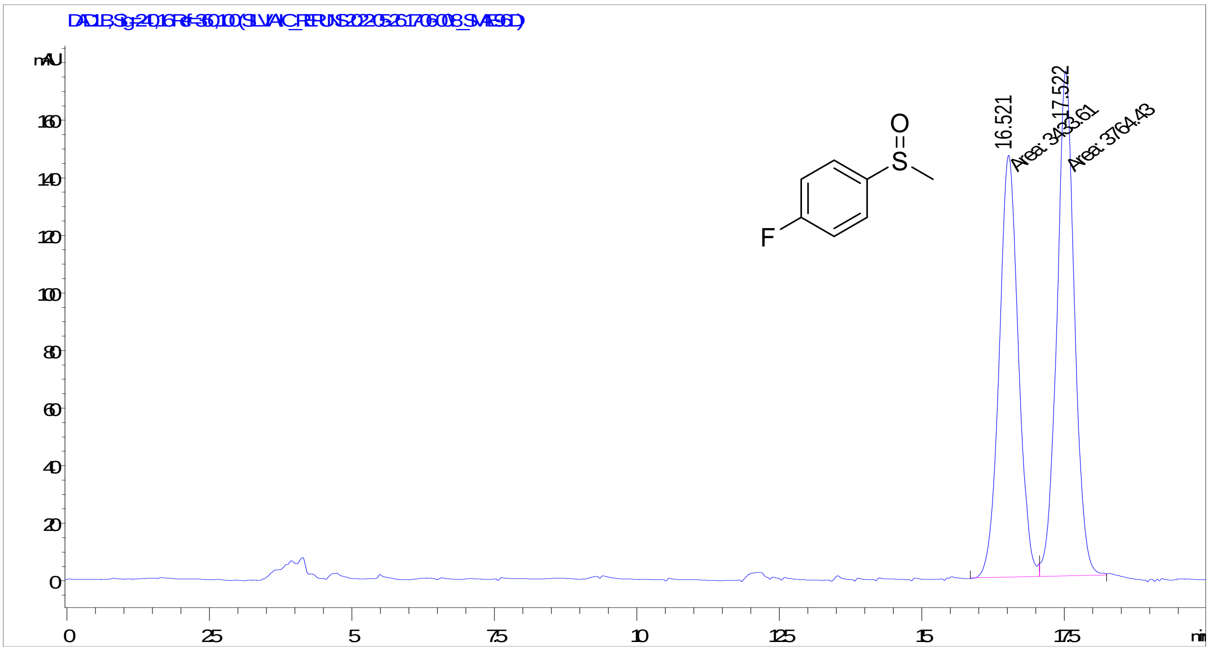

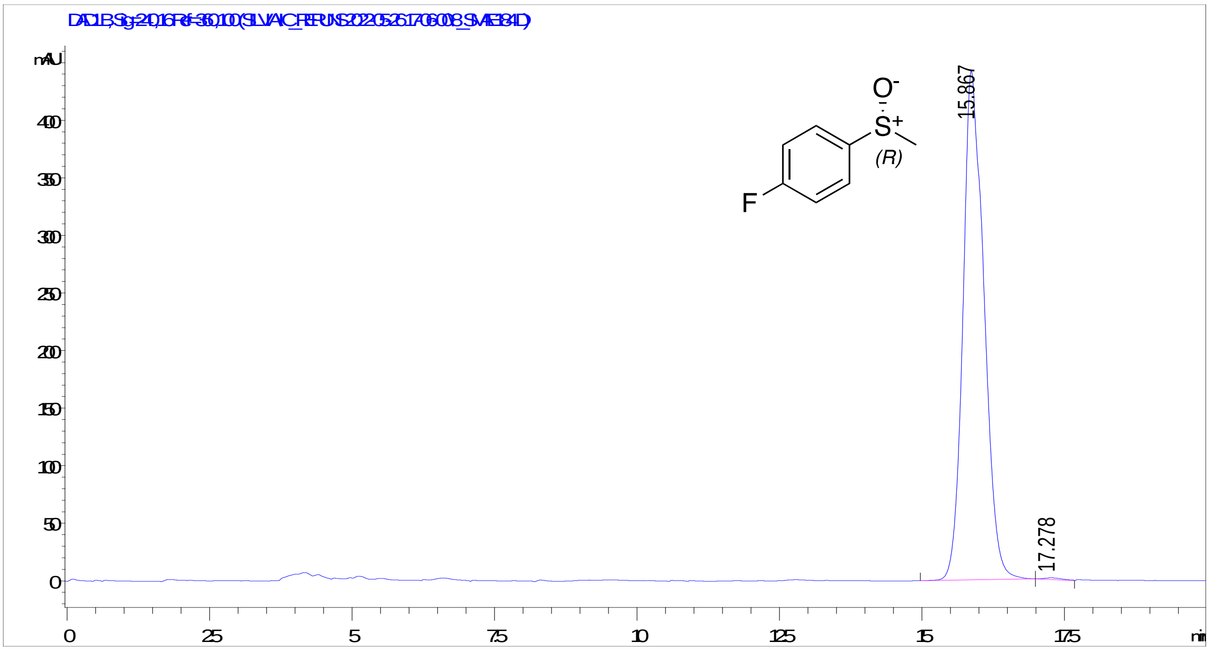

6.4.4 1-Bromo-4-(methylsulfinyl)benzene (1d)

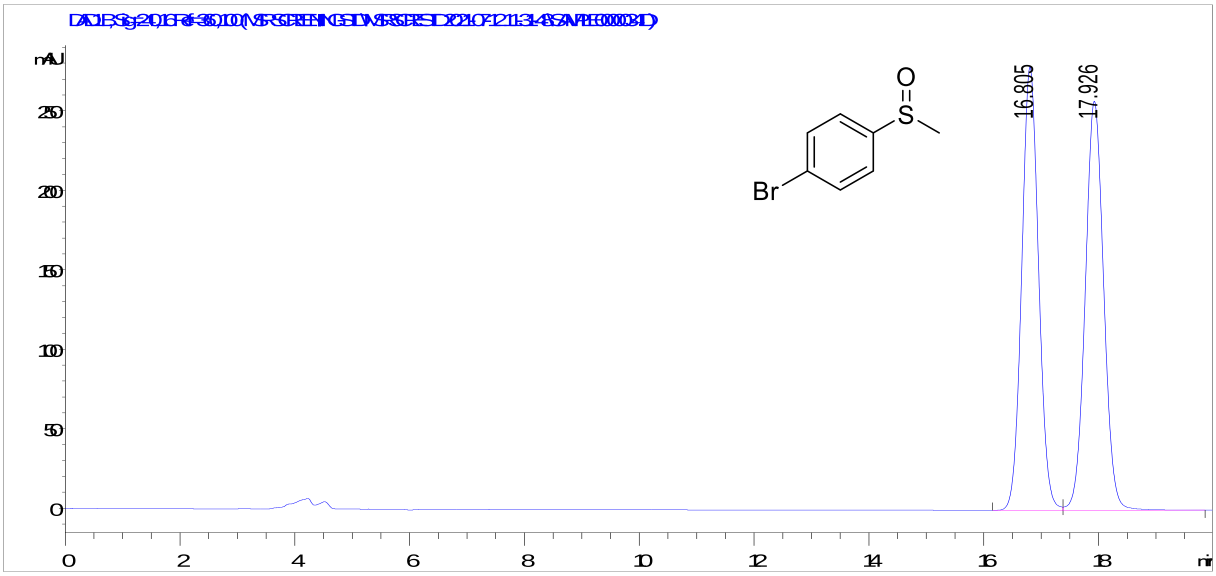

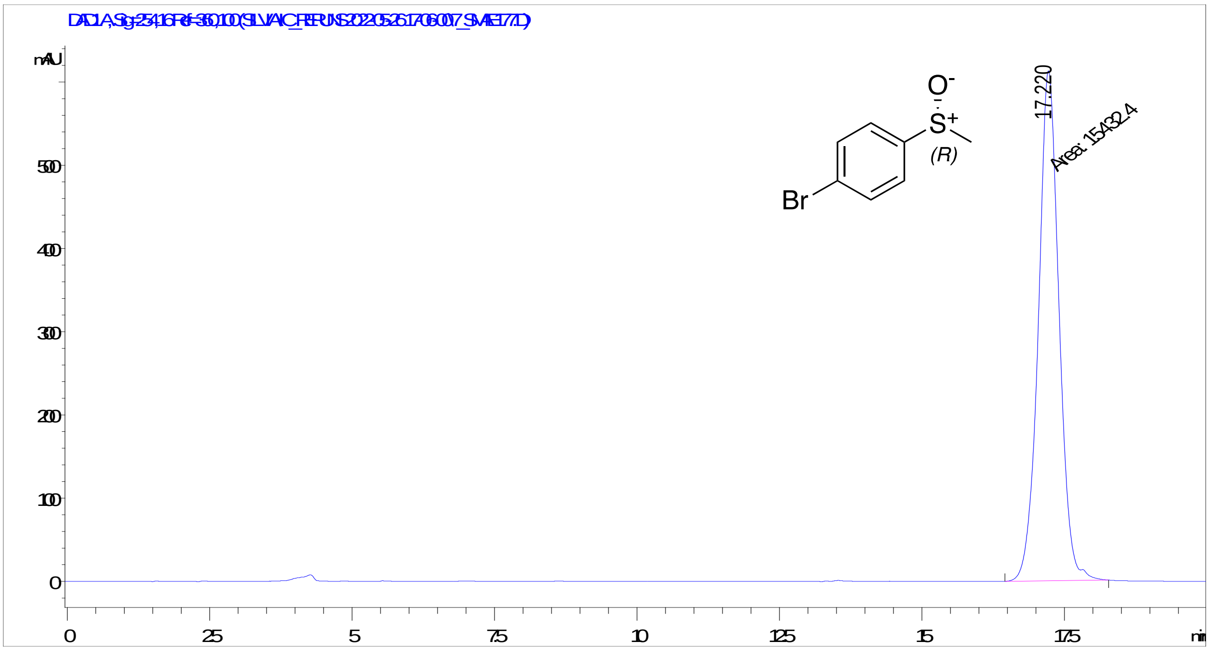

6.4.5 1-Chloro-4-(methylsulfinyl)benzene (1e)

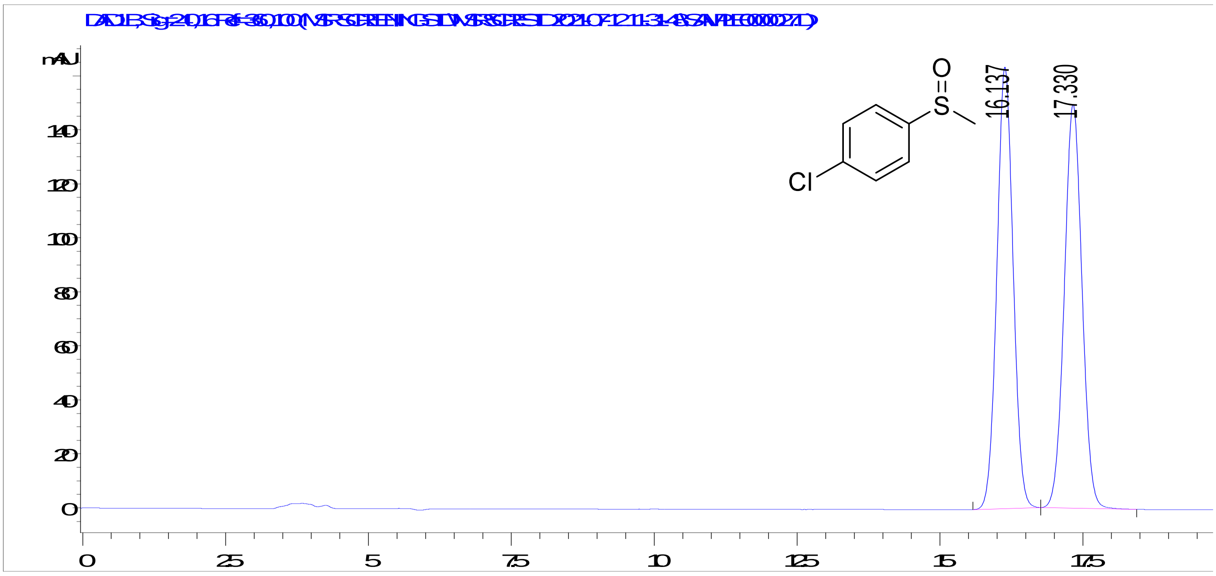

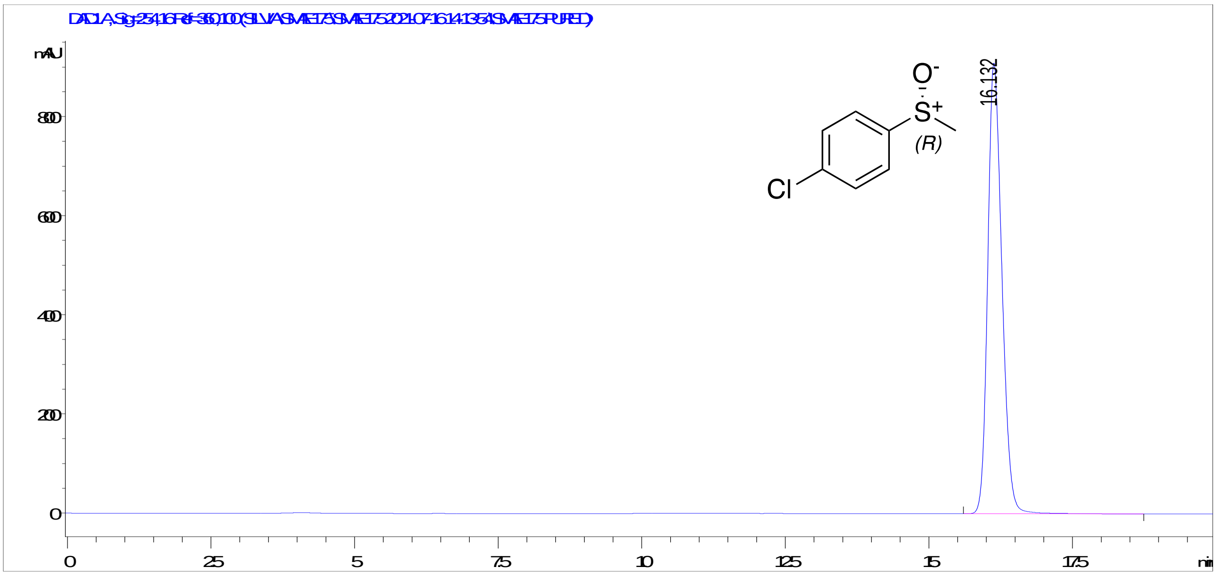

6.4.6 1-Chloro-3-(methylsulfinyl)benzene (1f)

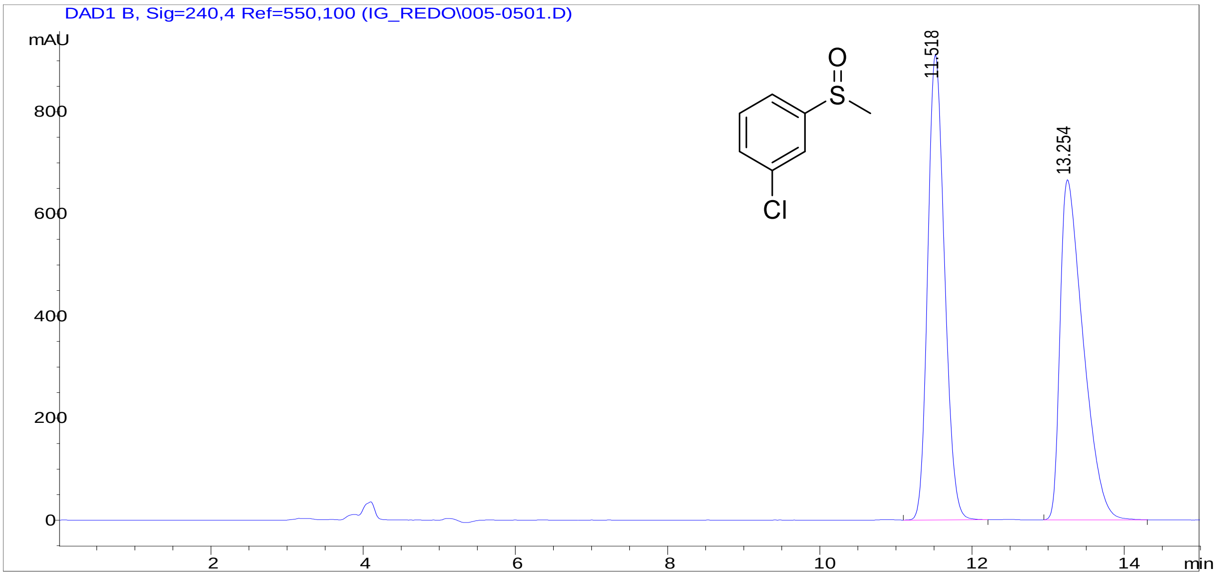

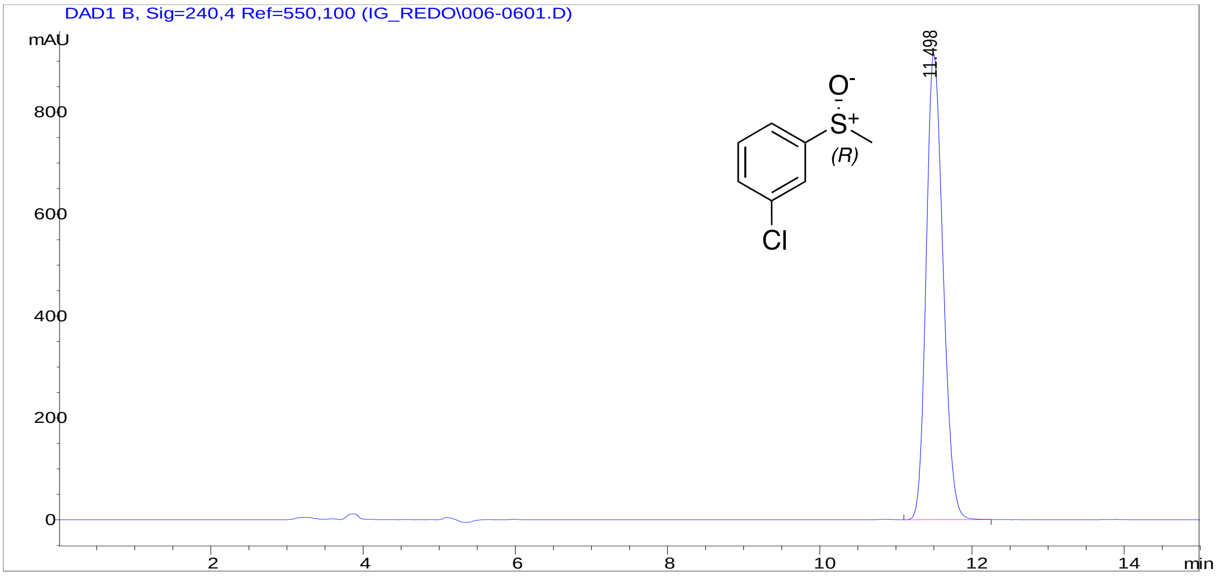

6.4.7 1-chloro-2-(methylsulfinyl)benzene (1g)

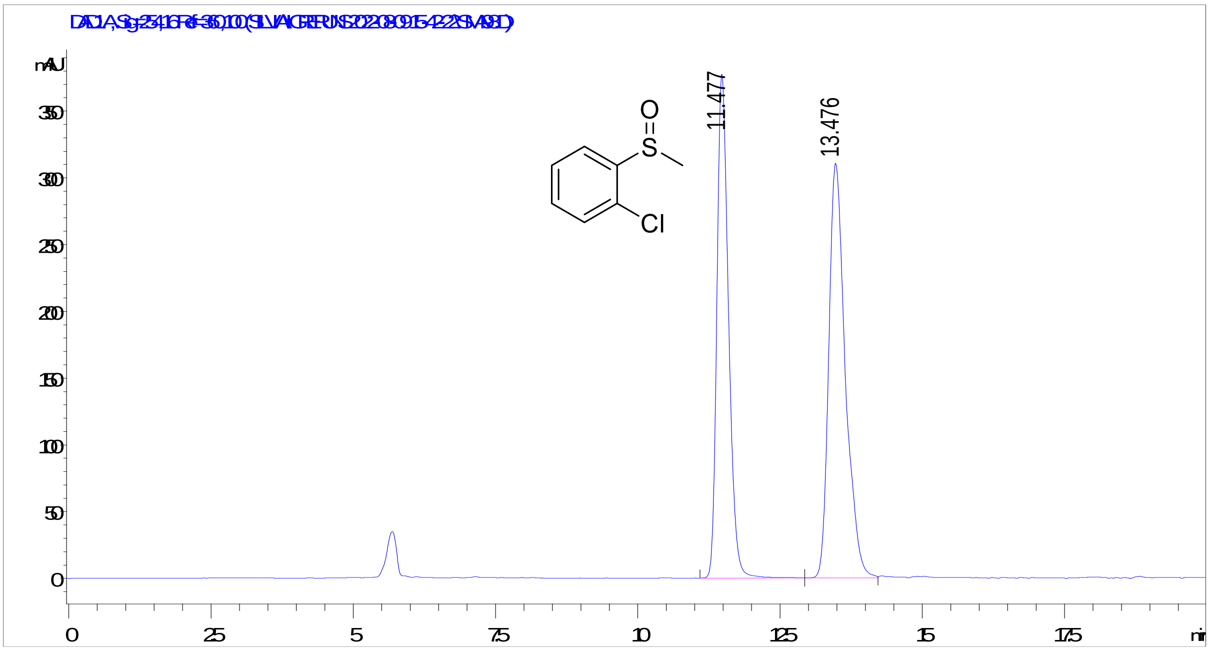

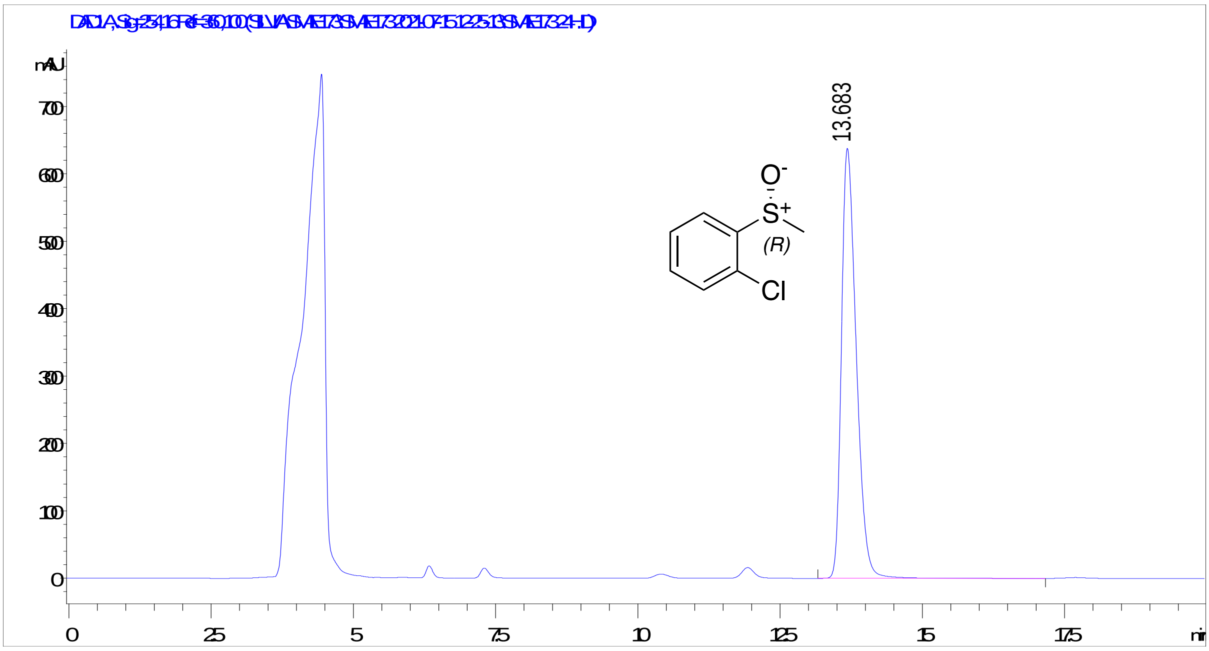

6.4.8 1-(4-(Methylsulfinyl)phenyl)ethan-1-one (1h)

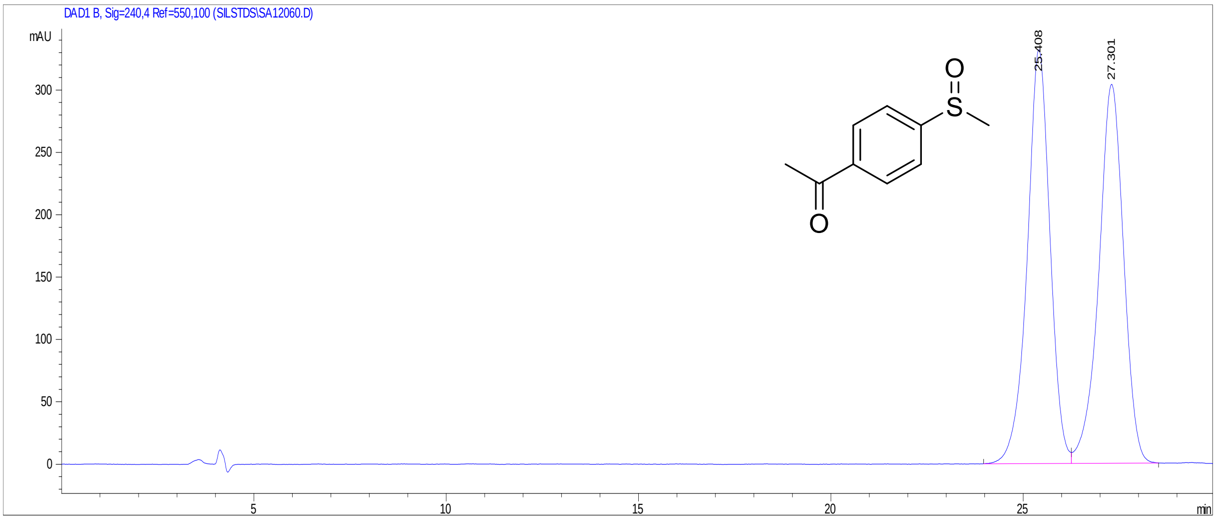

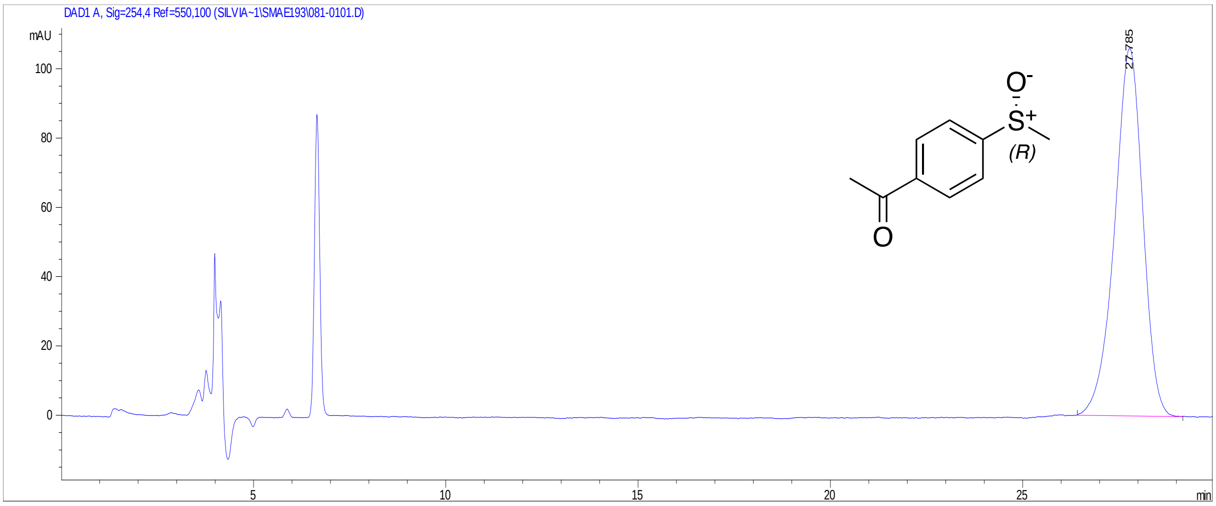

6.4.9 1-Methoxy-4-(methylsulfinyl)benzene (1i)

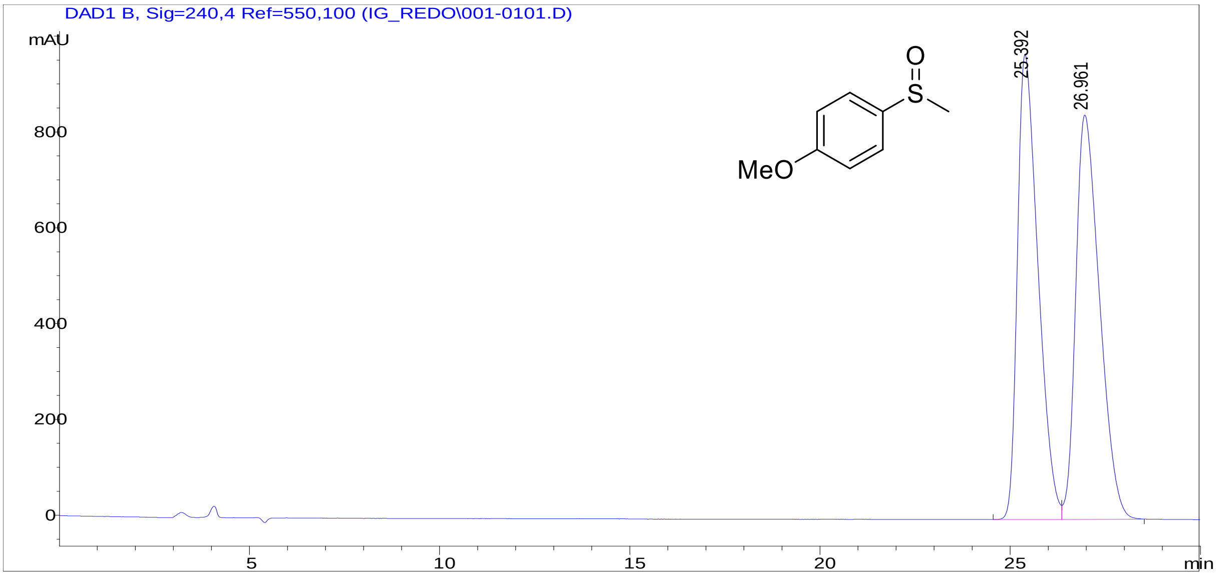

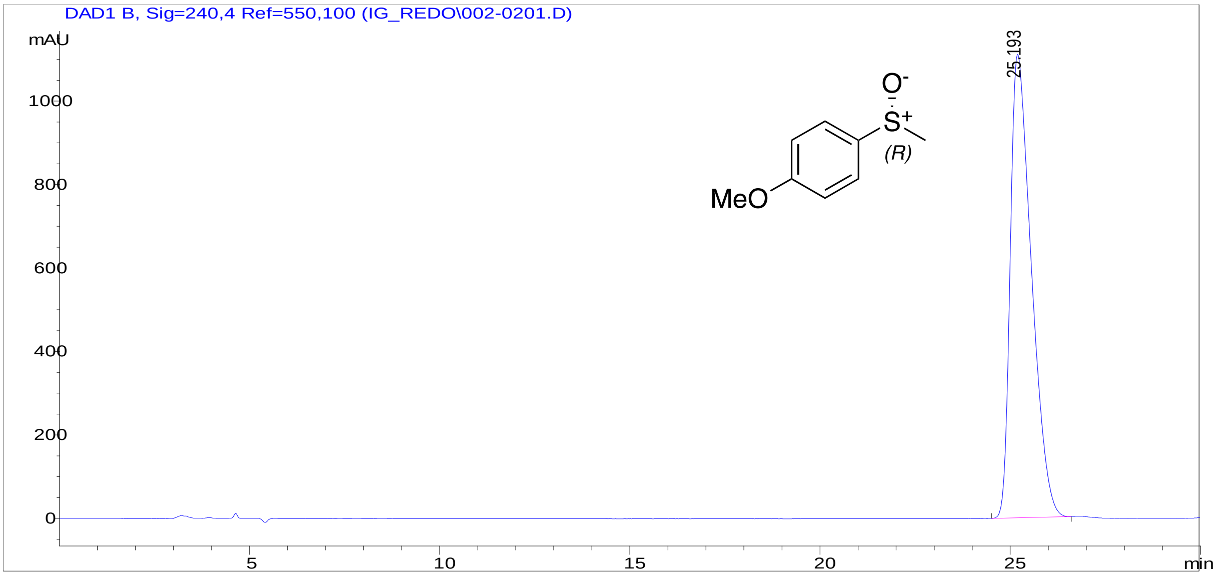

6.4.10 1-Methoxy-3-(methylsulfinyl)benzene (1j)

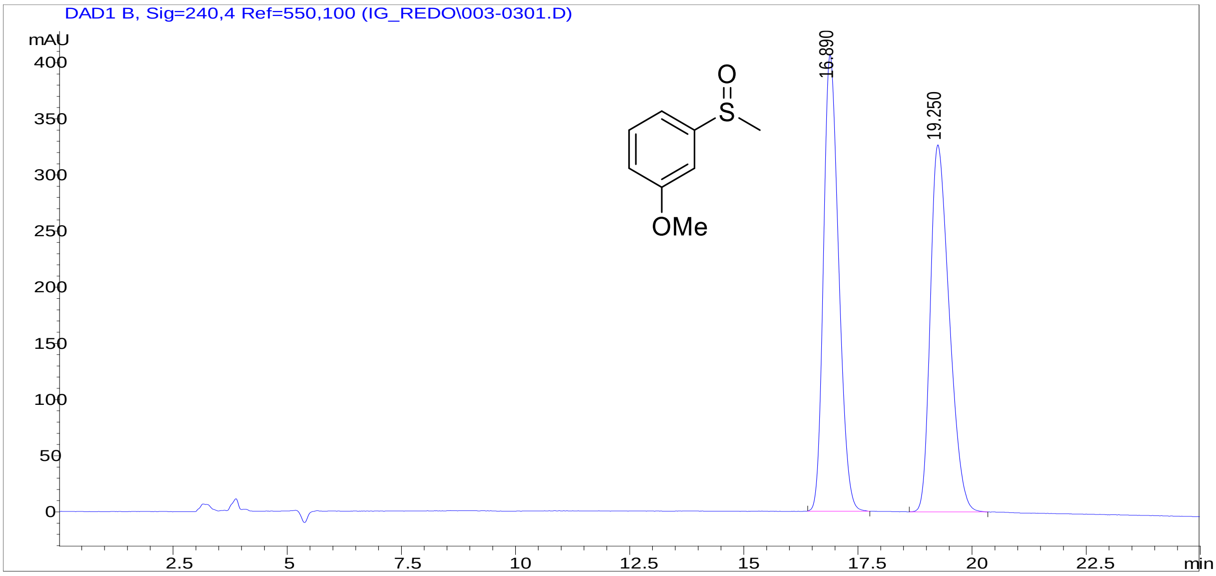

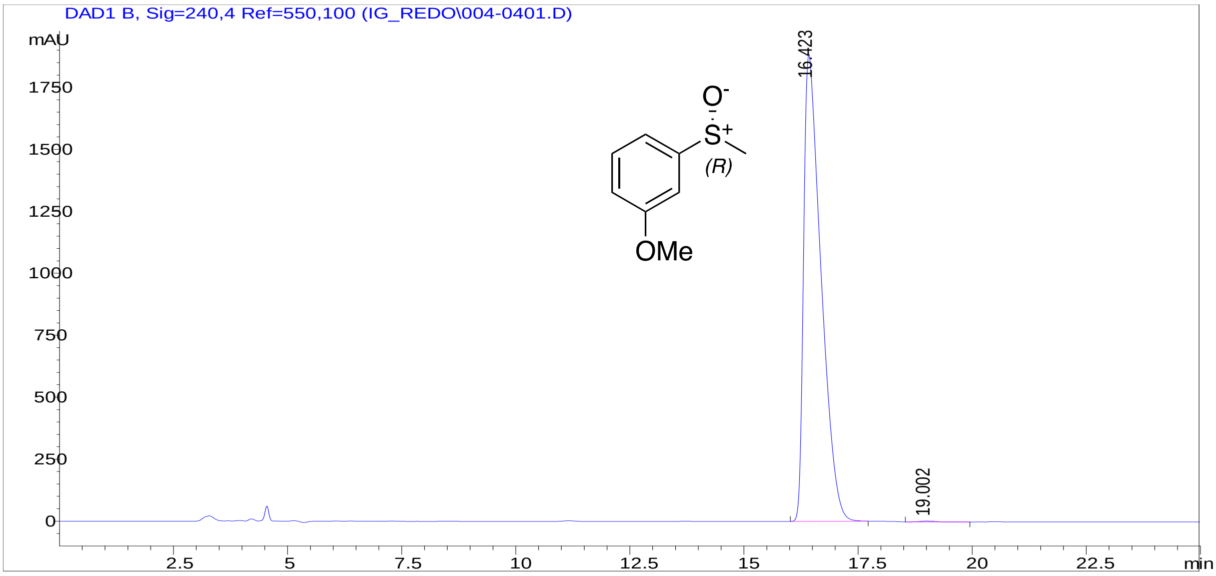

6.4.11 1-Methyl-3-(methylsulfinyl)benzene (1k)

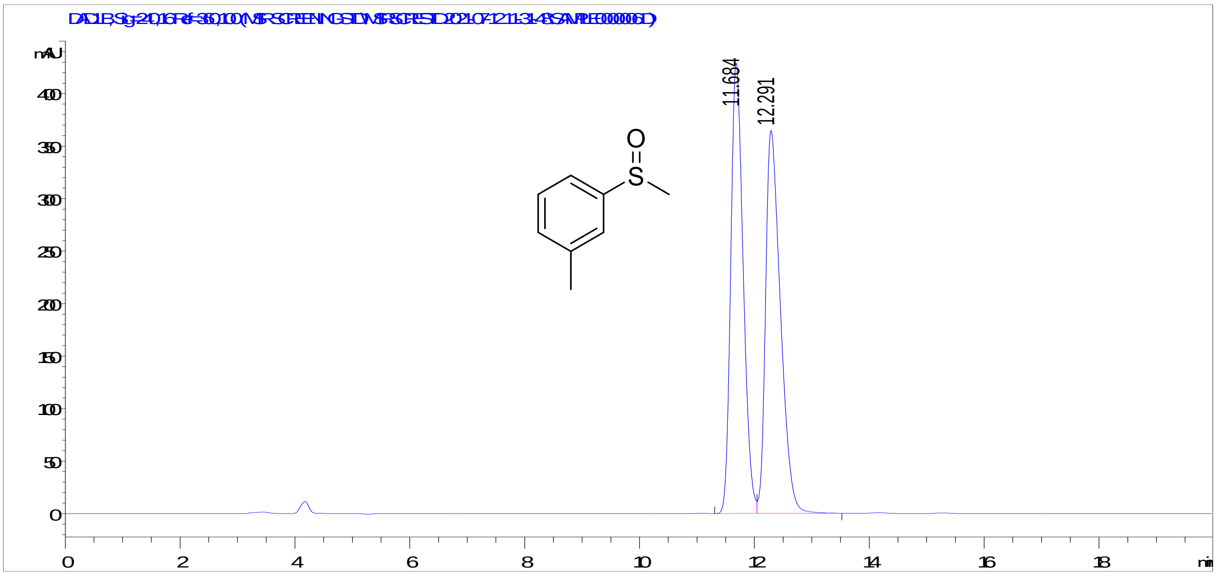

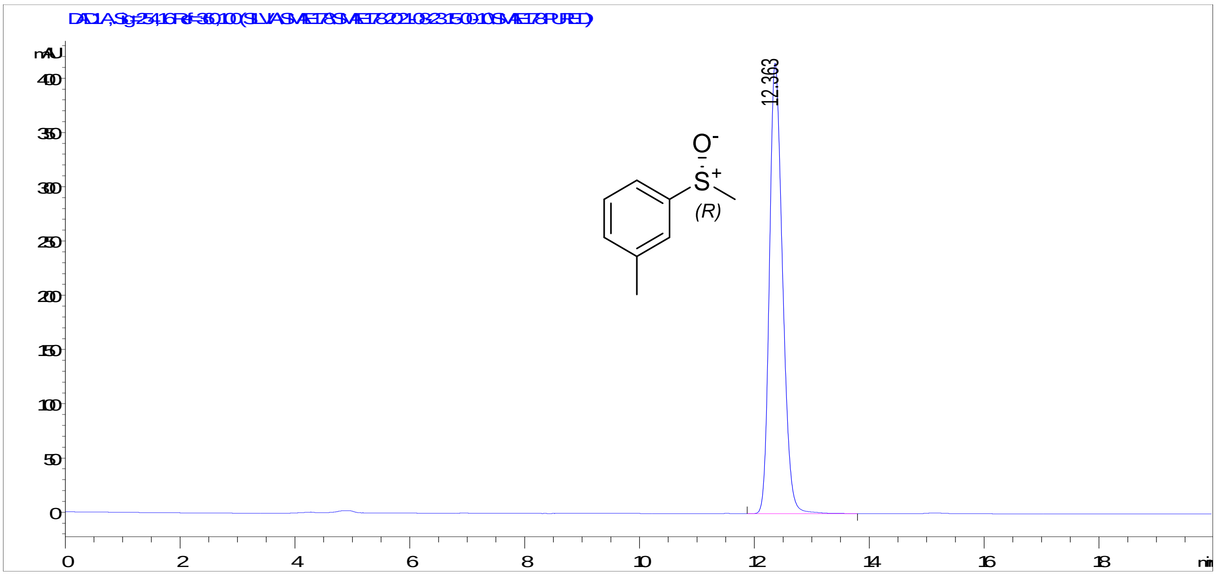

6.4.12 2,4-Dimethyl-1-(methylsulfinyl)benzene (1l)

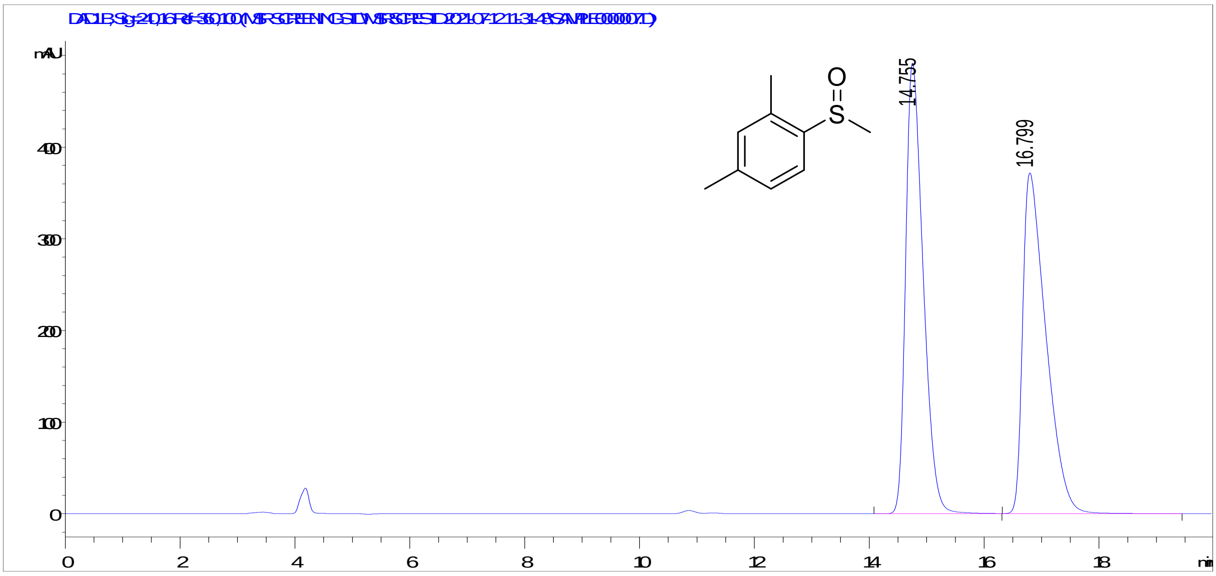

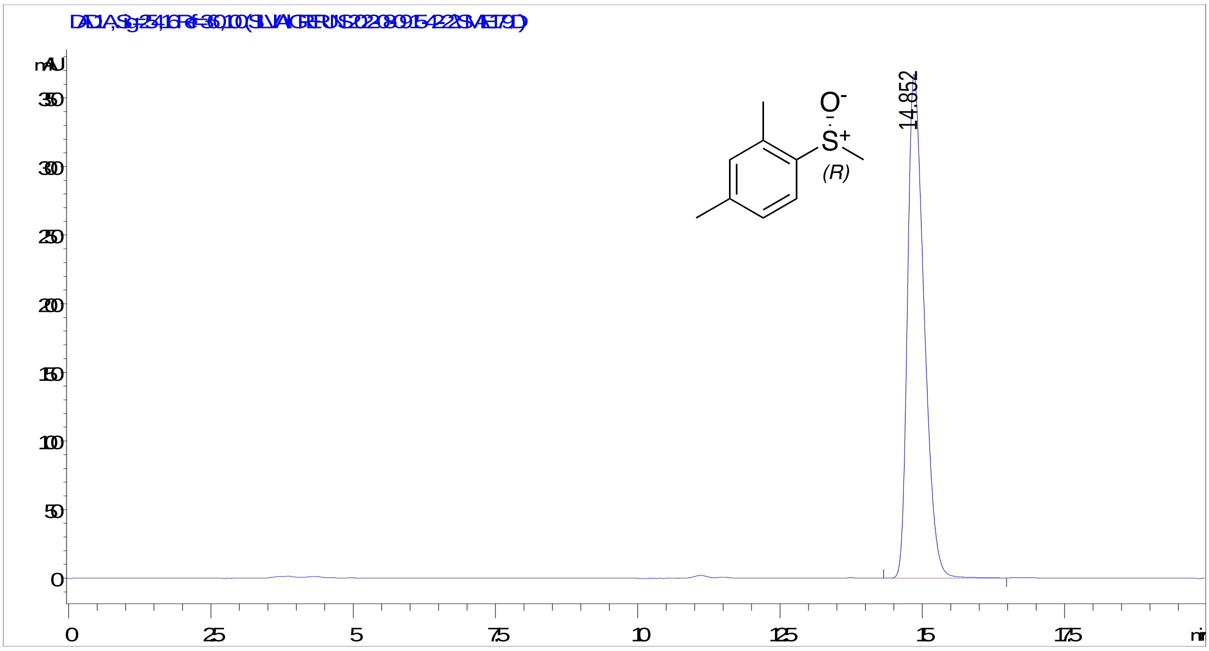

6.4.13 2-(Methylsulfinyl)naphthalene (1m)

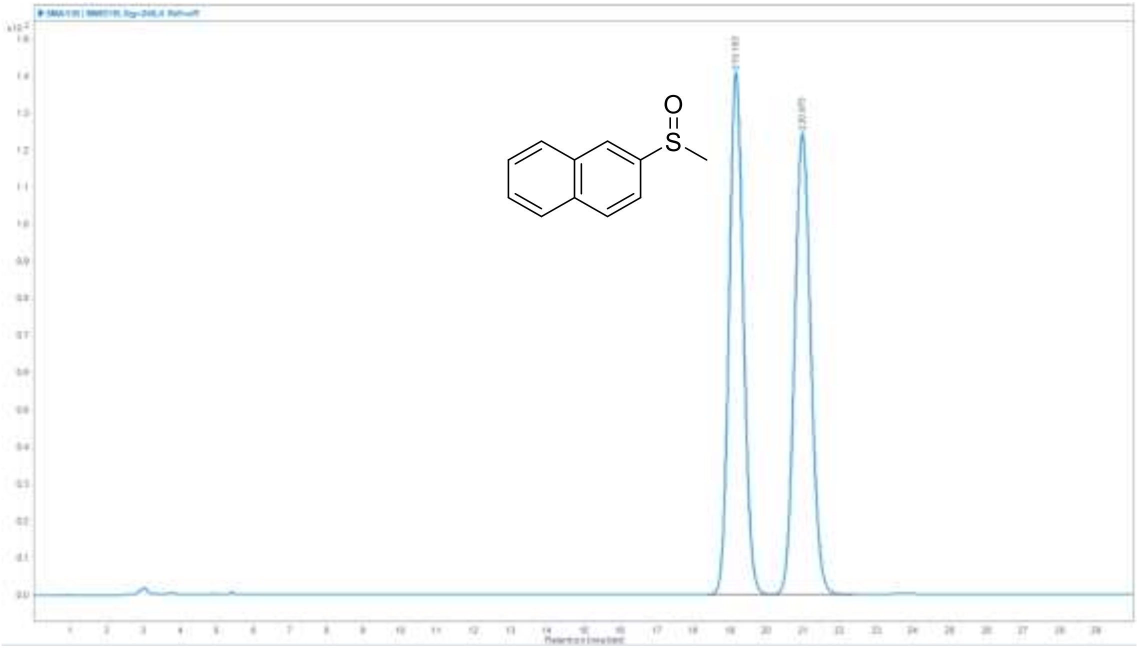

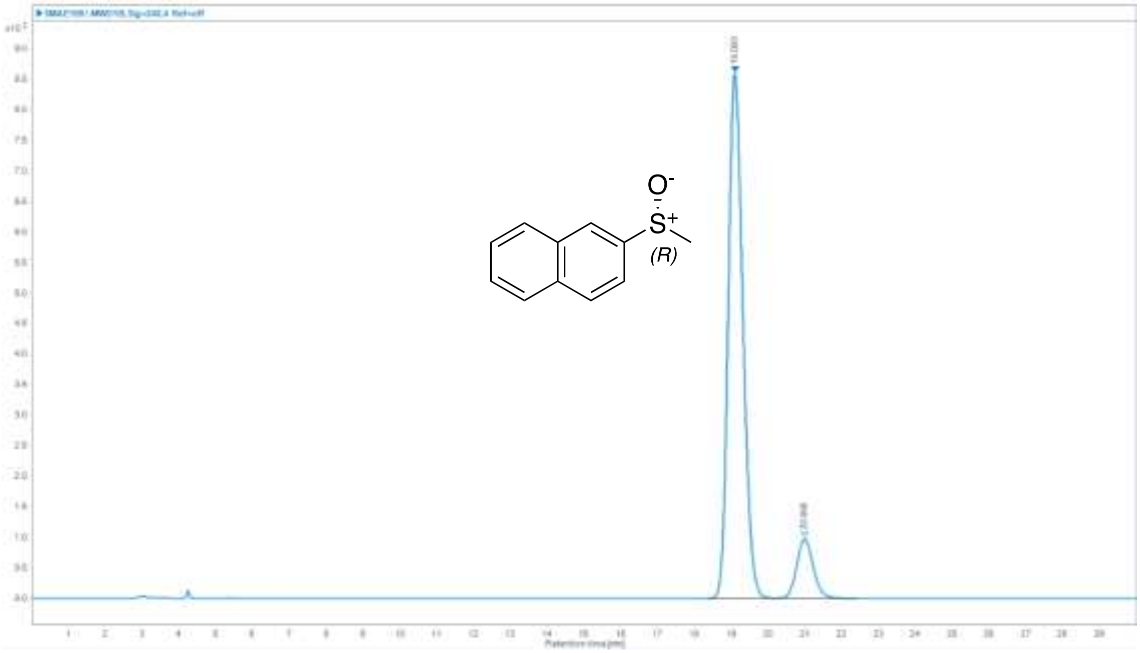

6.4.14 1-(Methylsulfinyl)naphthalene (1n)

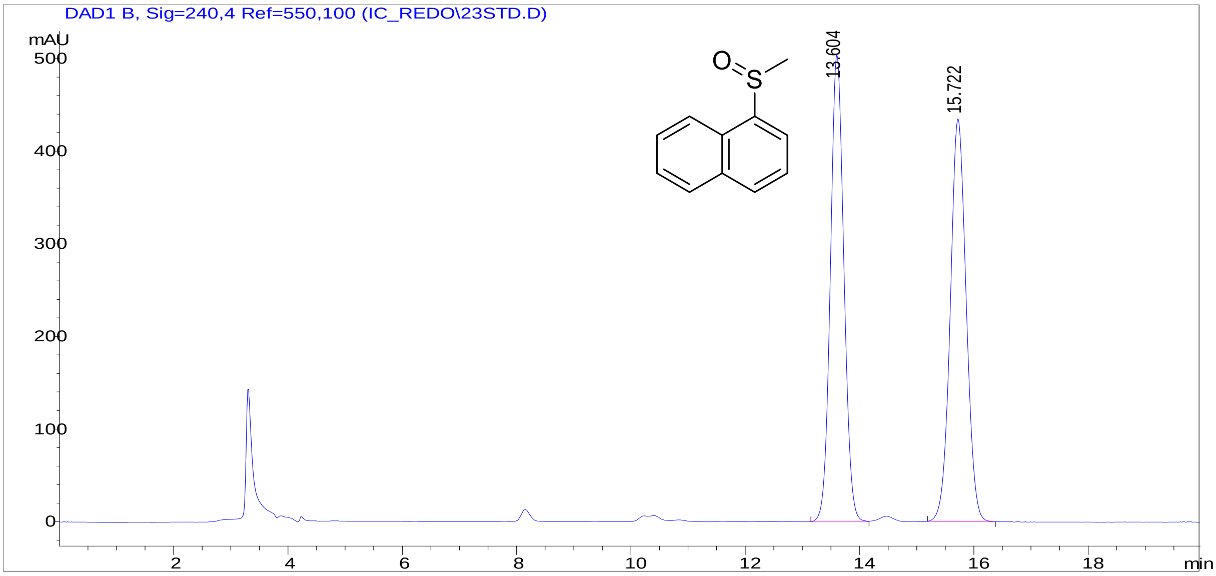

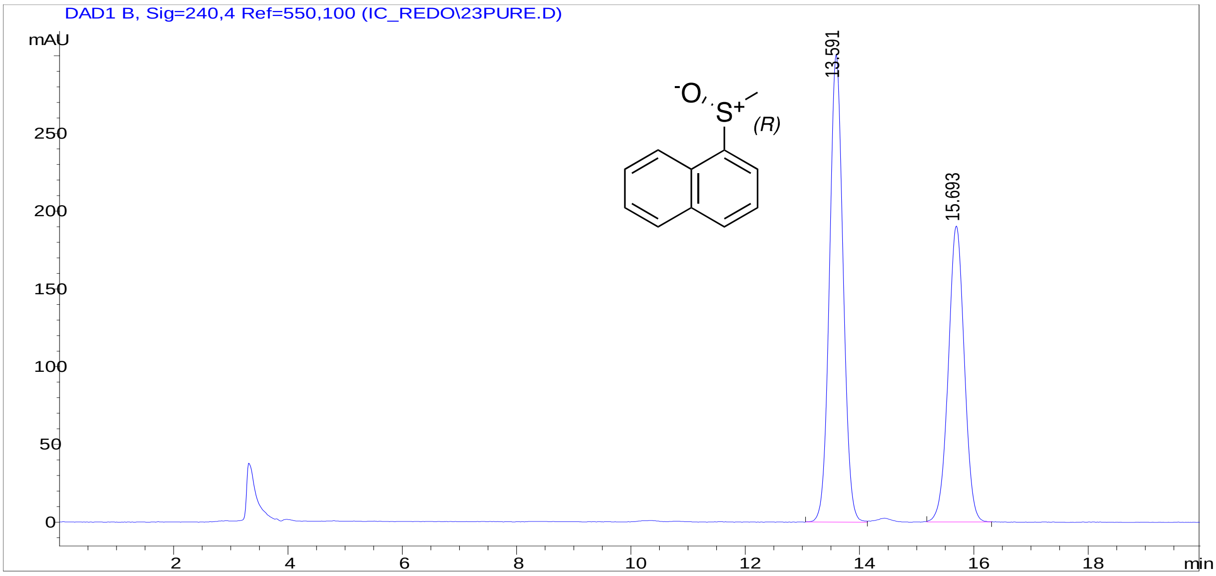

6.4.15 ((Methylsulfinyl)methyl)benzene (1o)

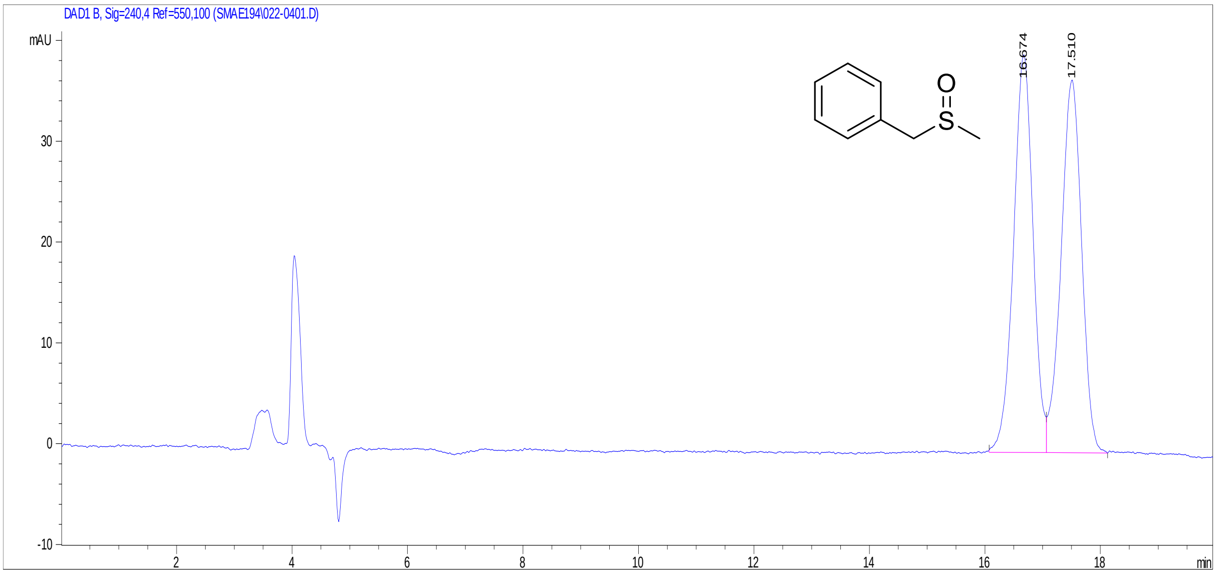

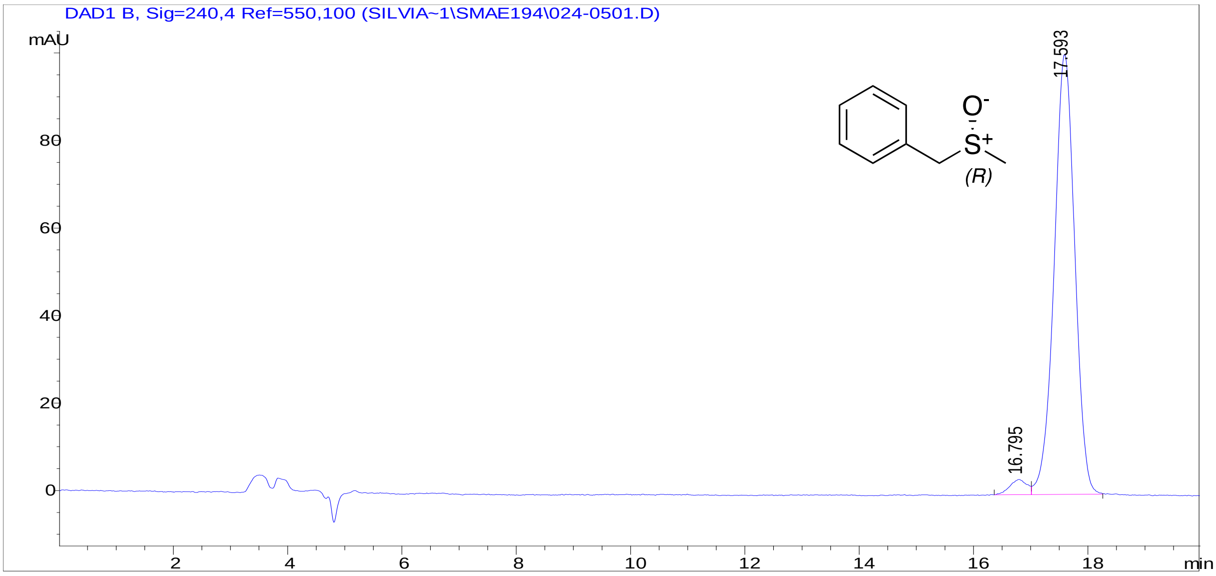

6.4.16 1-(Methylsulfinyl)dodecane (1p)

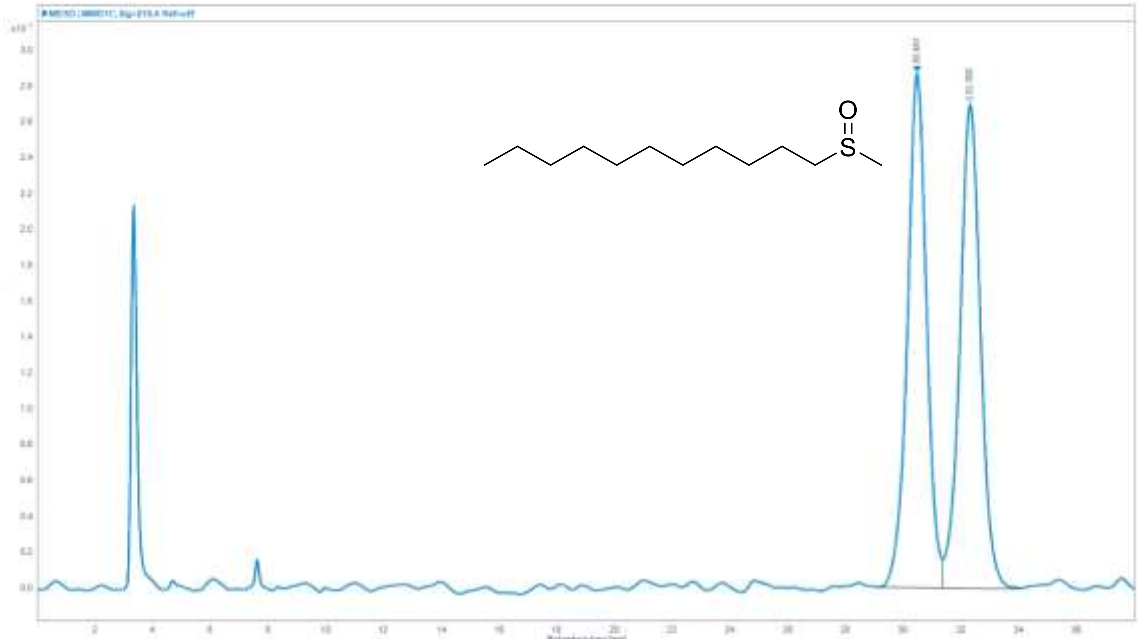

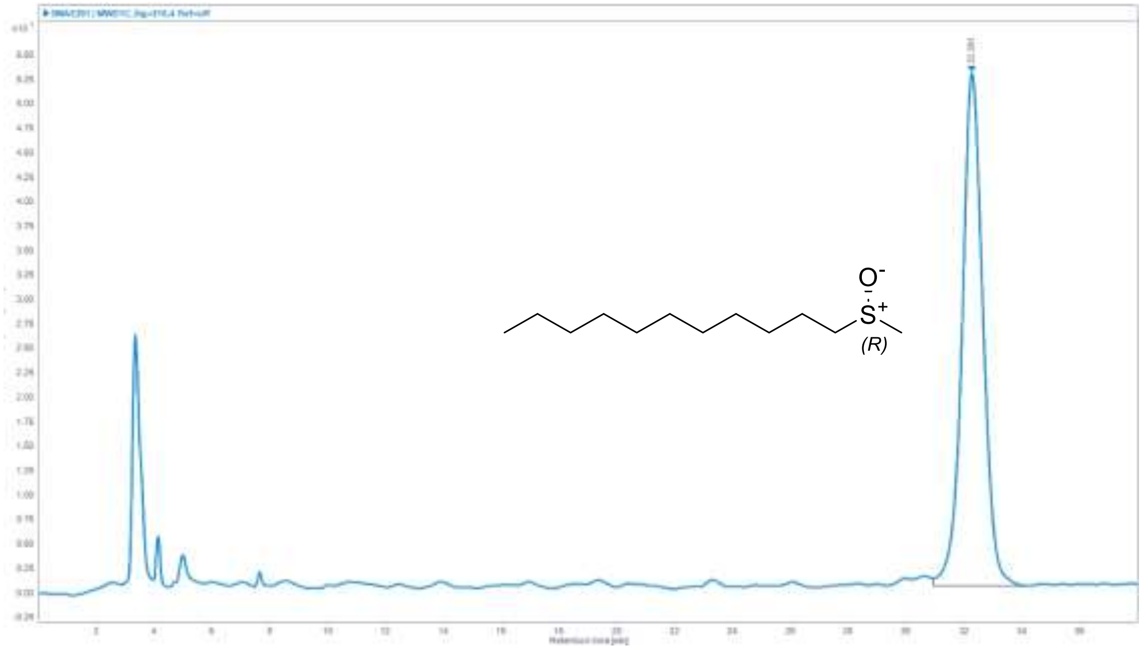

6.4.17 4-(Methylsulfinyl)butan-2-one (1q)

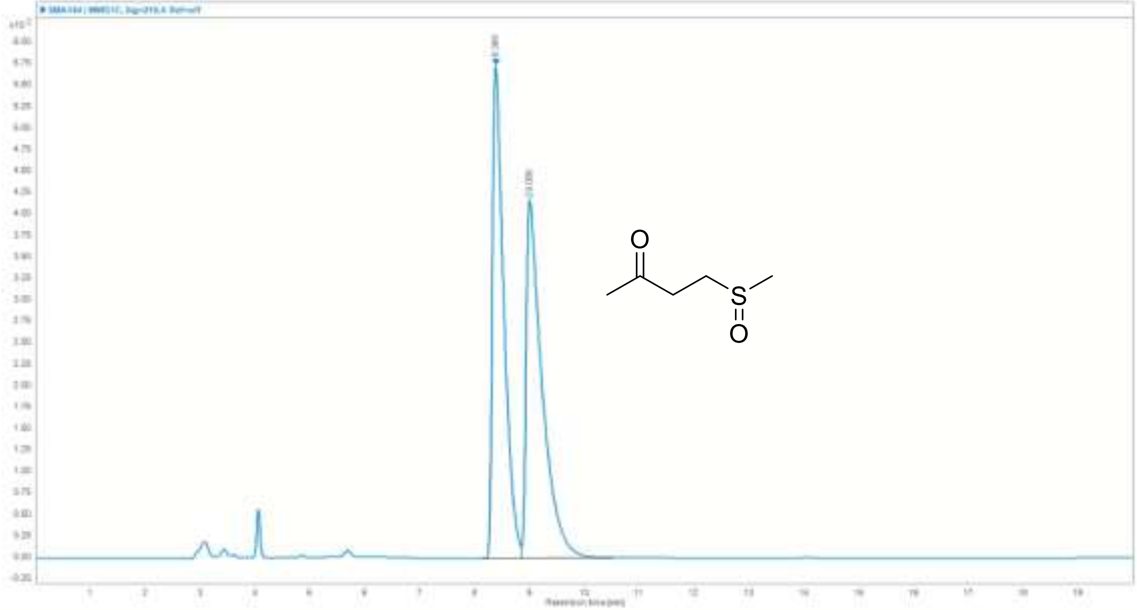

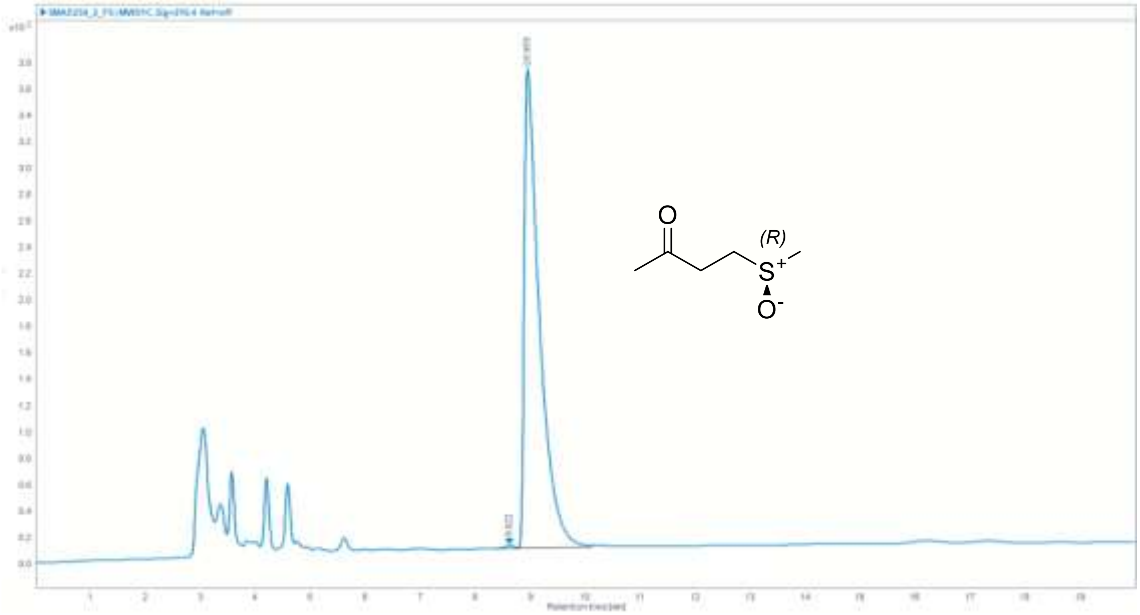

6.4.18 ((2-(Methylsulfinyl)ethyl)sulfinyl)benzene (1r)

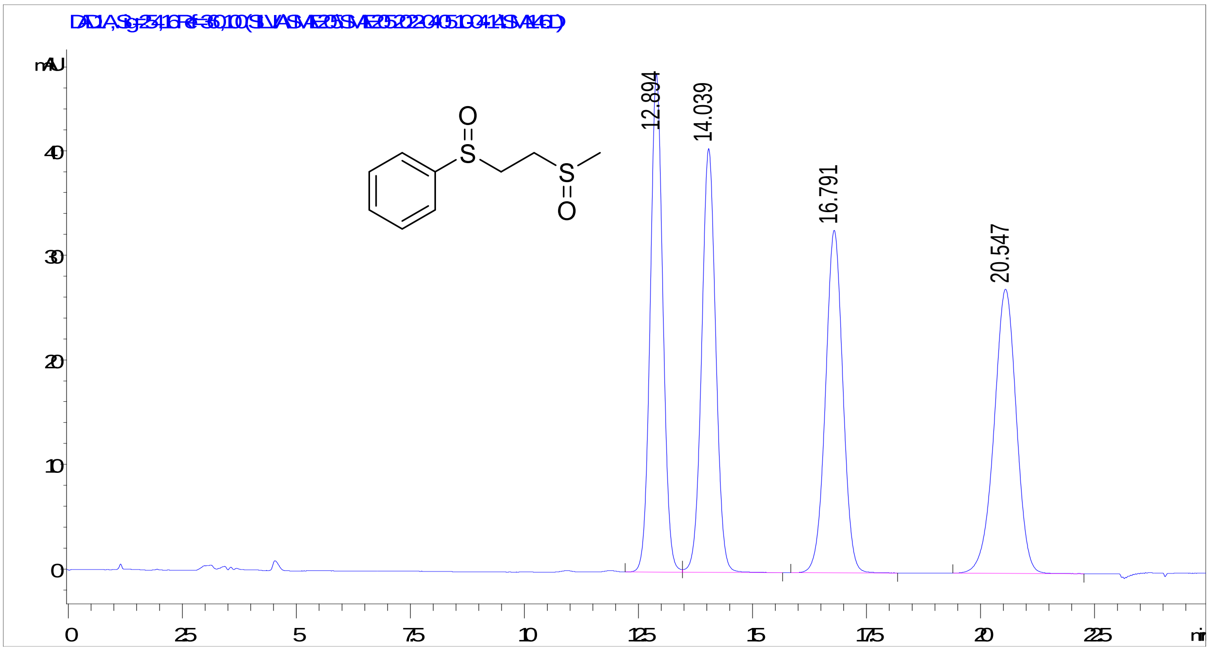

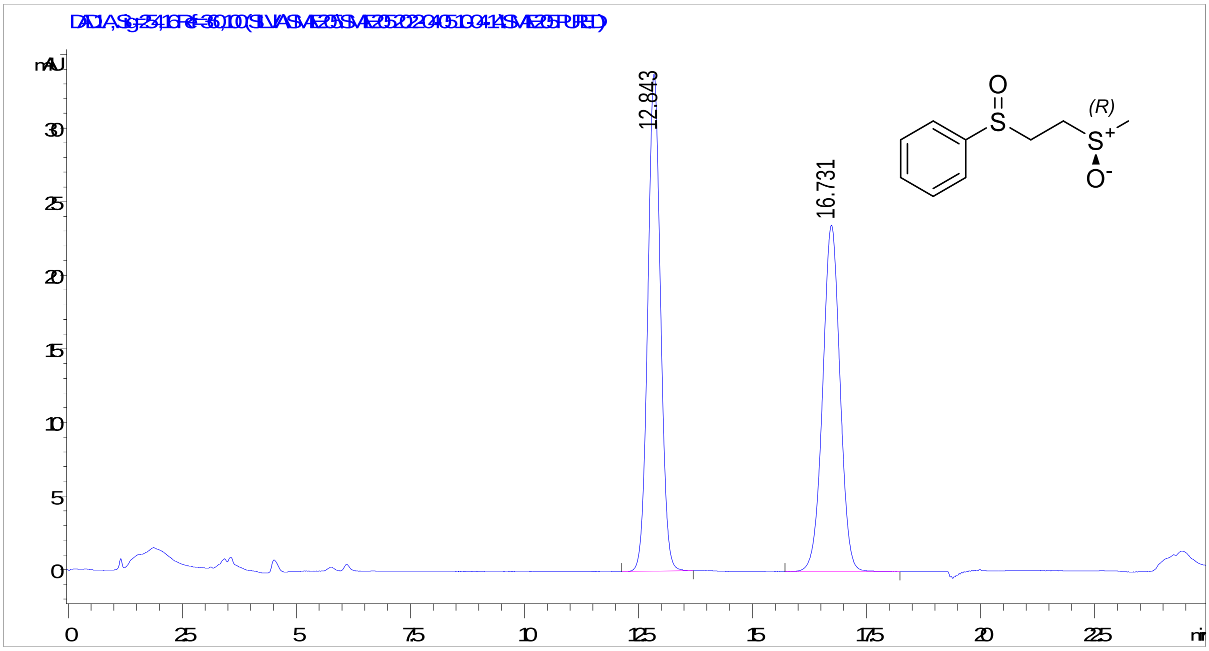

6.4.19 (Ethylsulfinyl)benzene (1s)

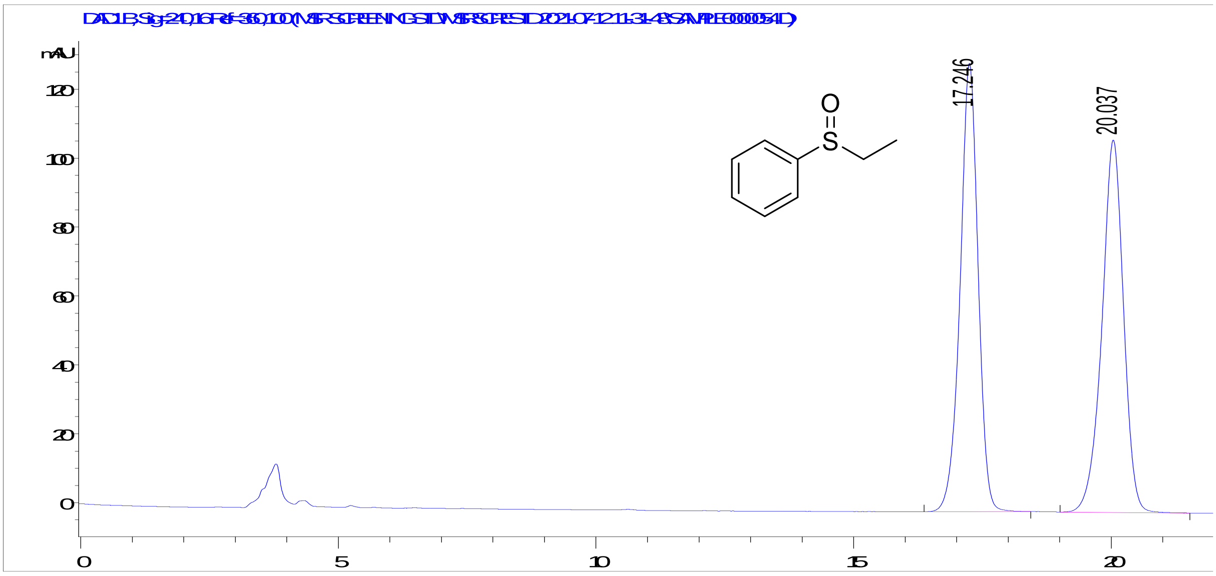

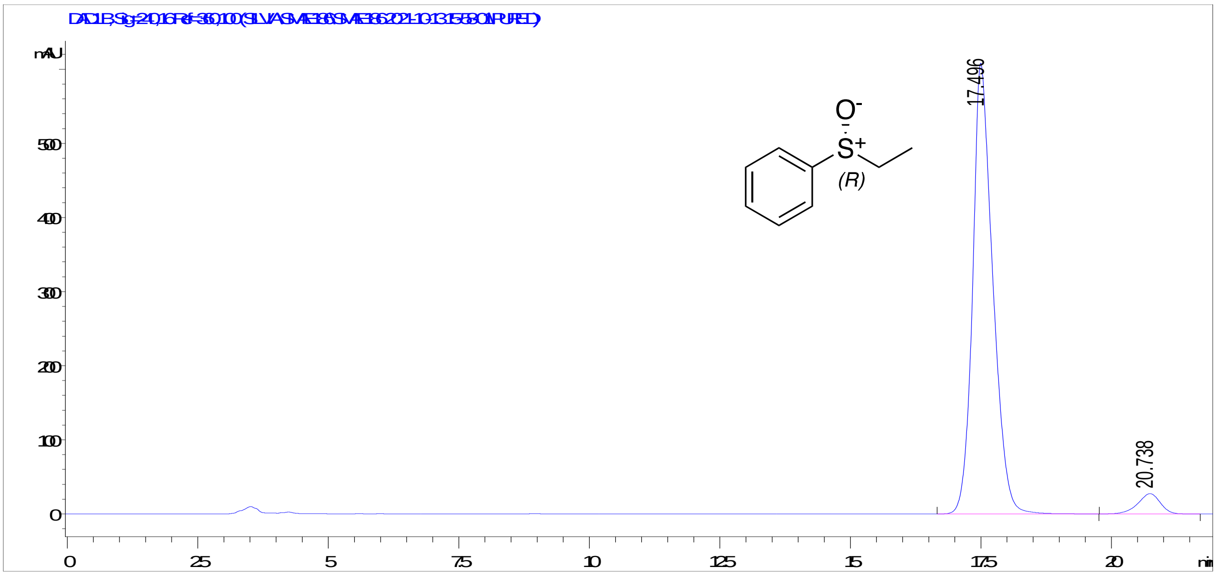

6.4.20 1-Methyl-4-(ethylsulfinyl)benzene (1t)

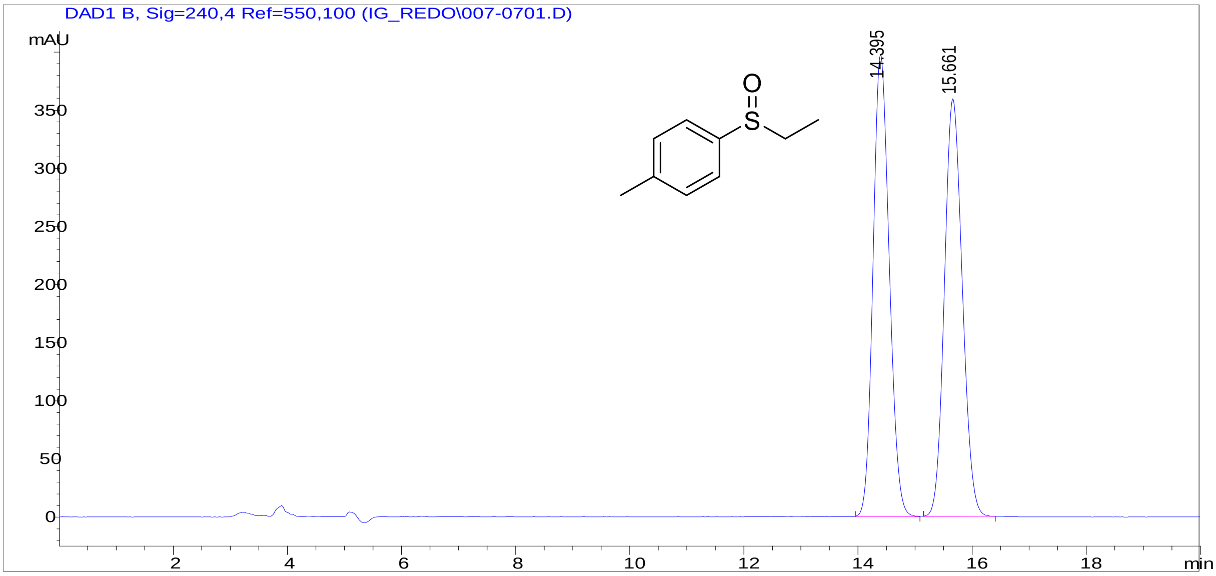

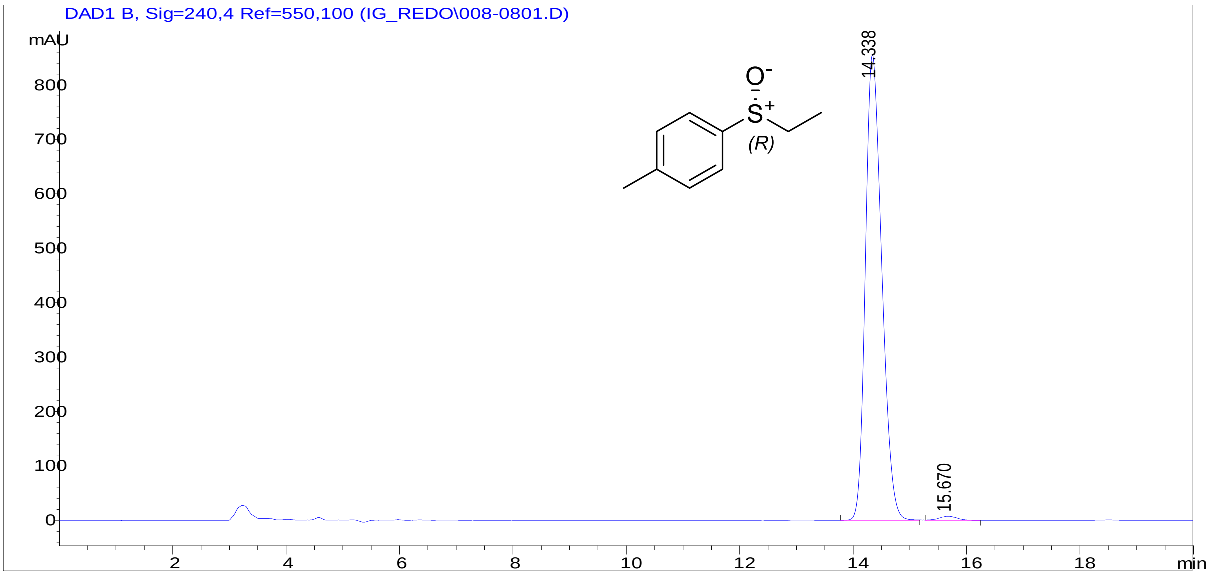

6.4.21 1-Bromo-4-(ethylsulfinyl)benzene (1u)

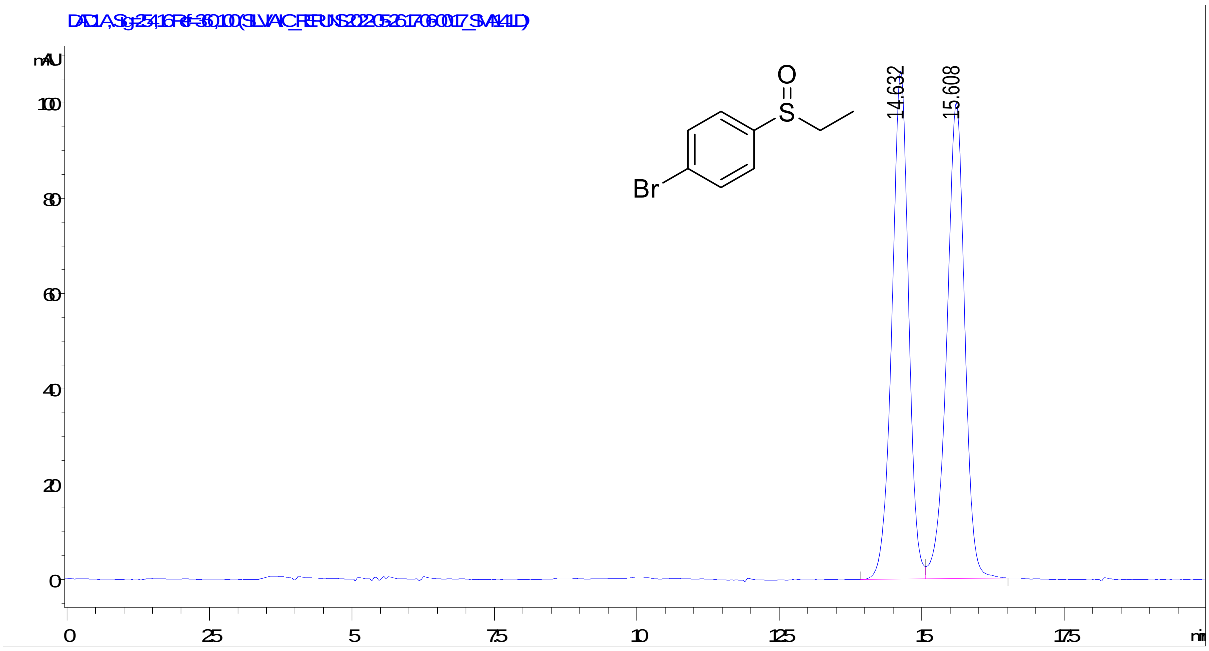

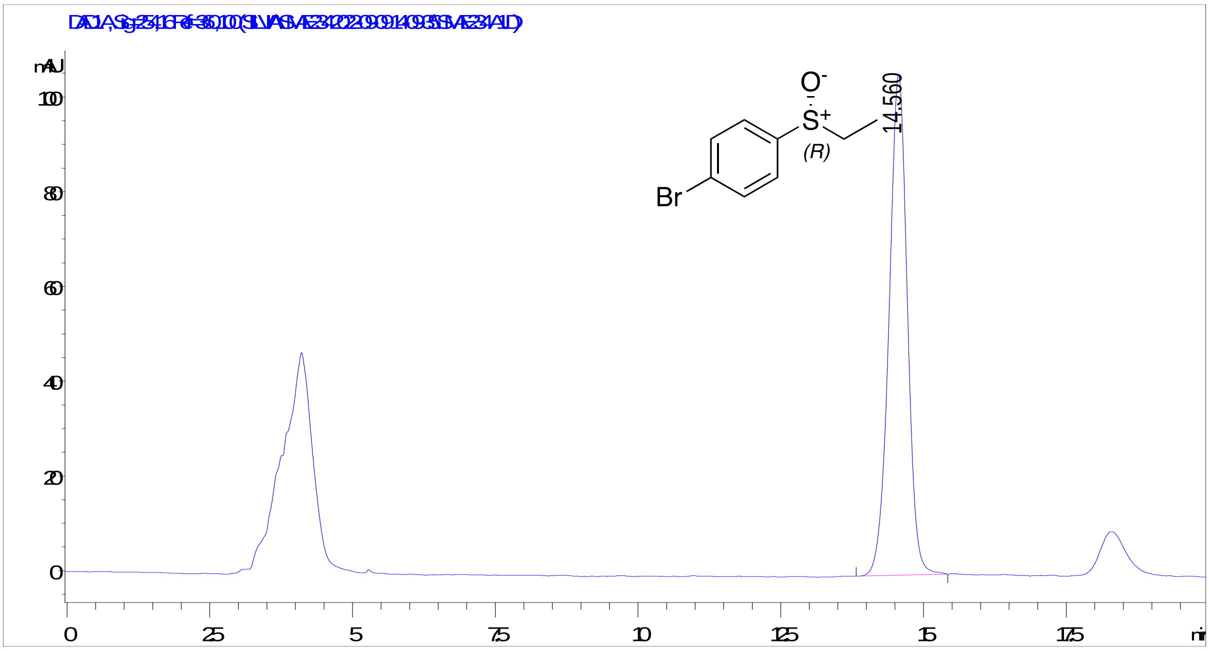

6.4.22 ((Ethylsulfinyl)methyl)benzene (1v)

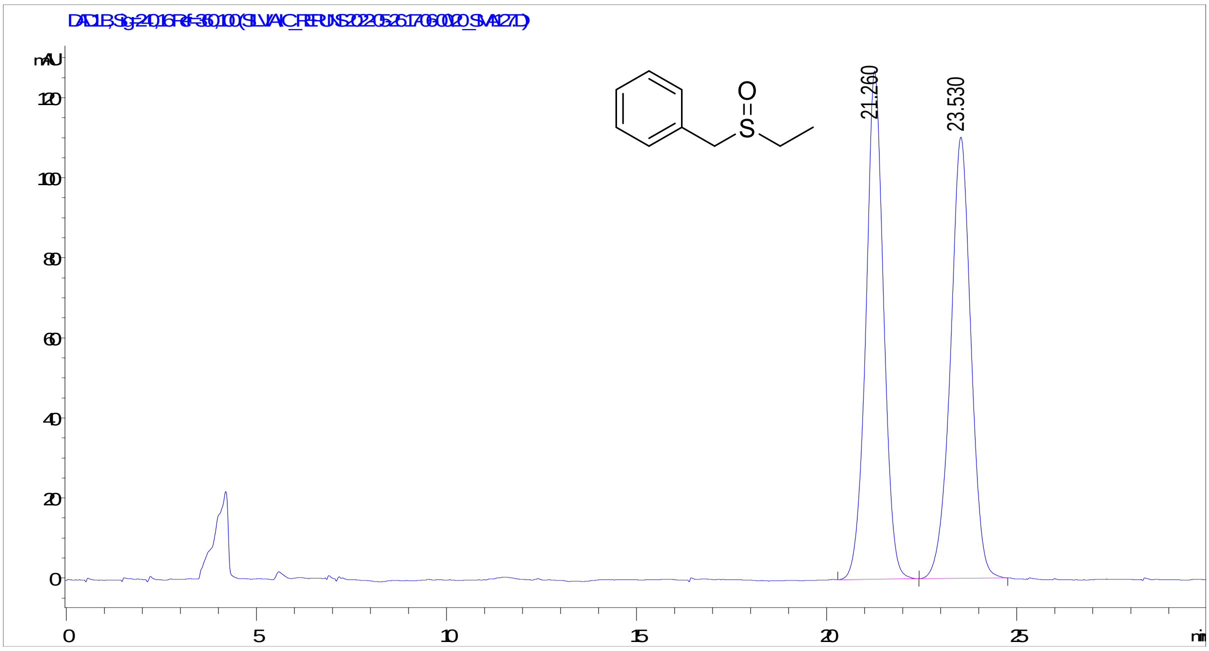

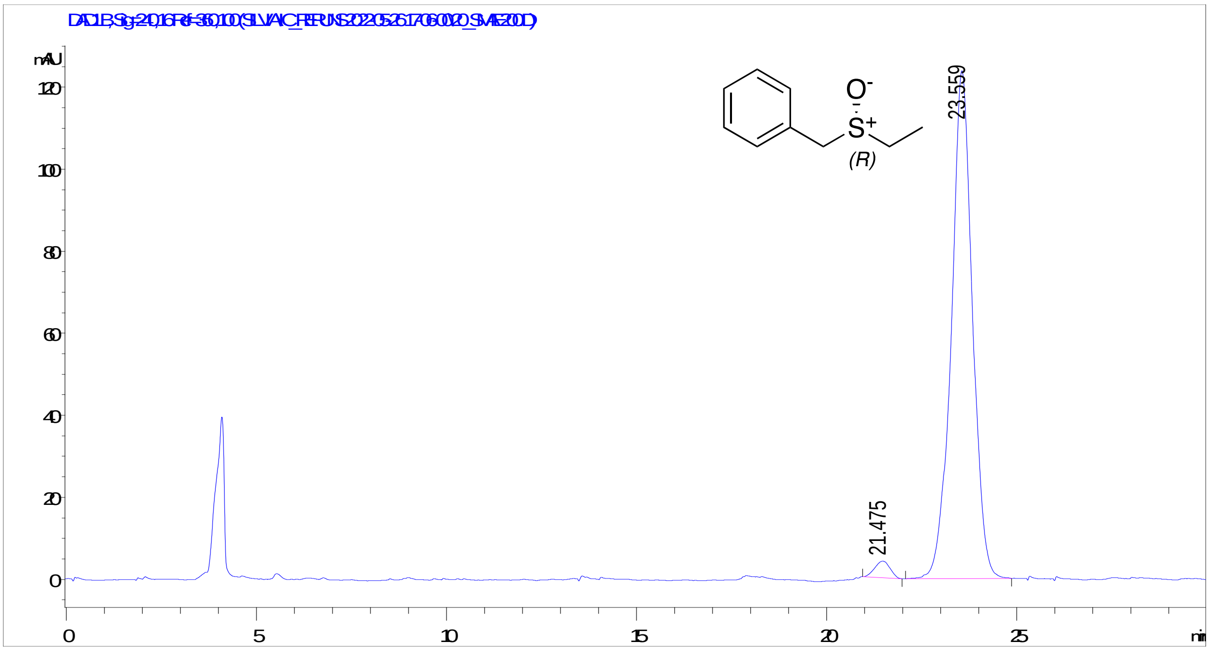

6.4.23 (2-(ethylsulfinyl)ethyl)benzene (1w)

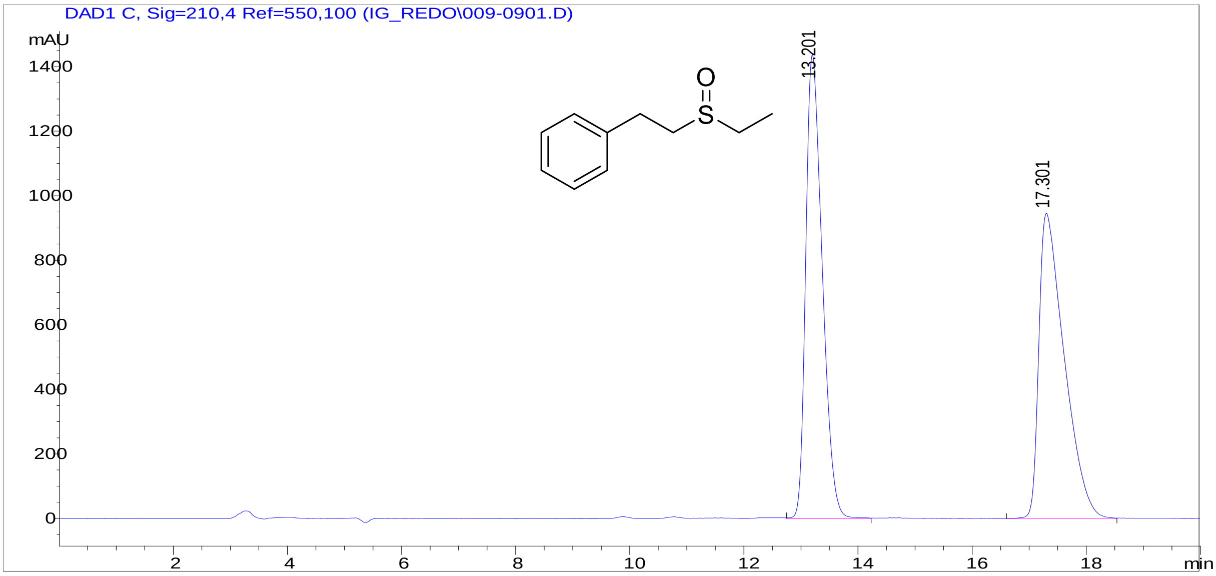

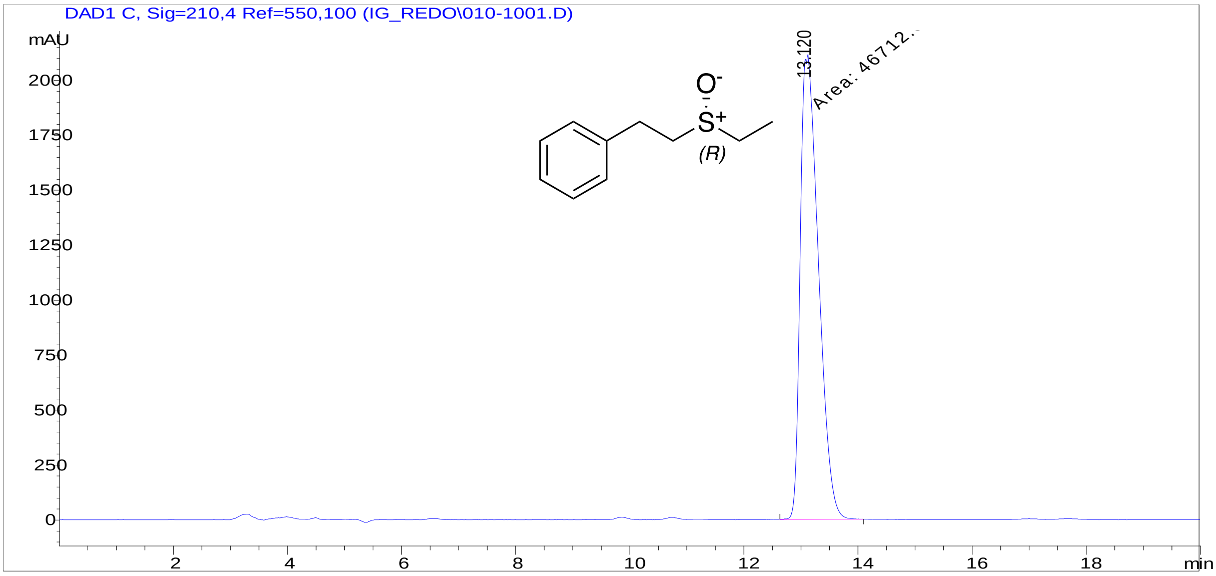

6.4.24 2-((Ethylsulfinyl)methyl)pyridine (1x)

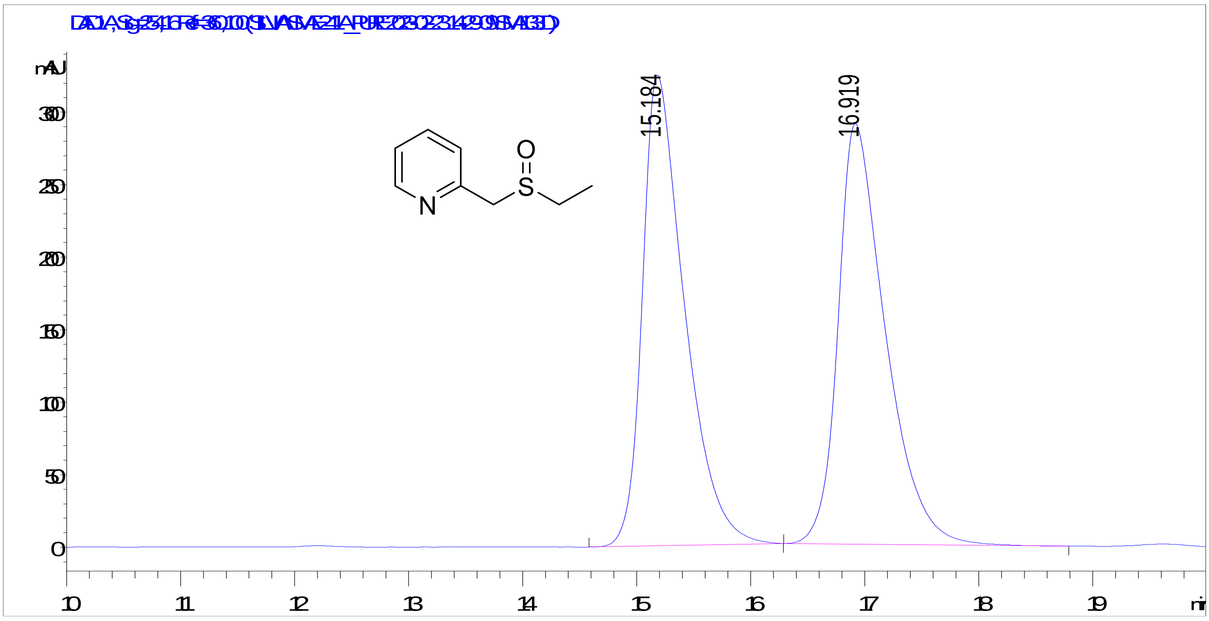

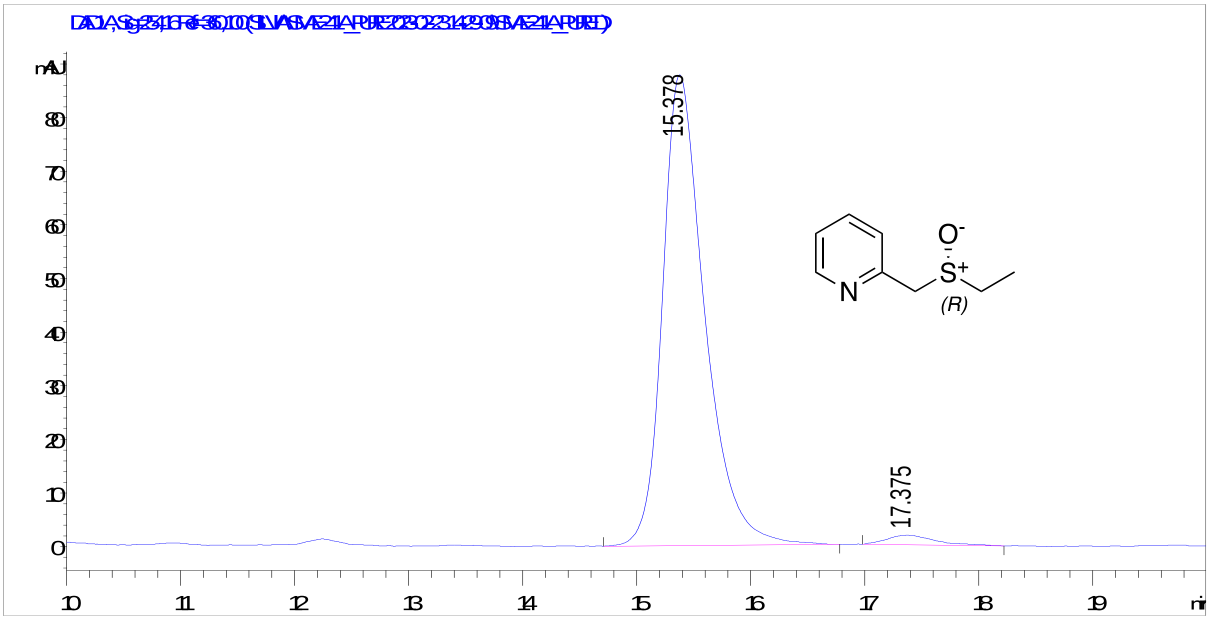

6.4.25 (Propylsulfinyl)benzene (1y)

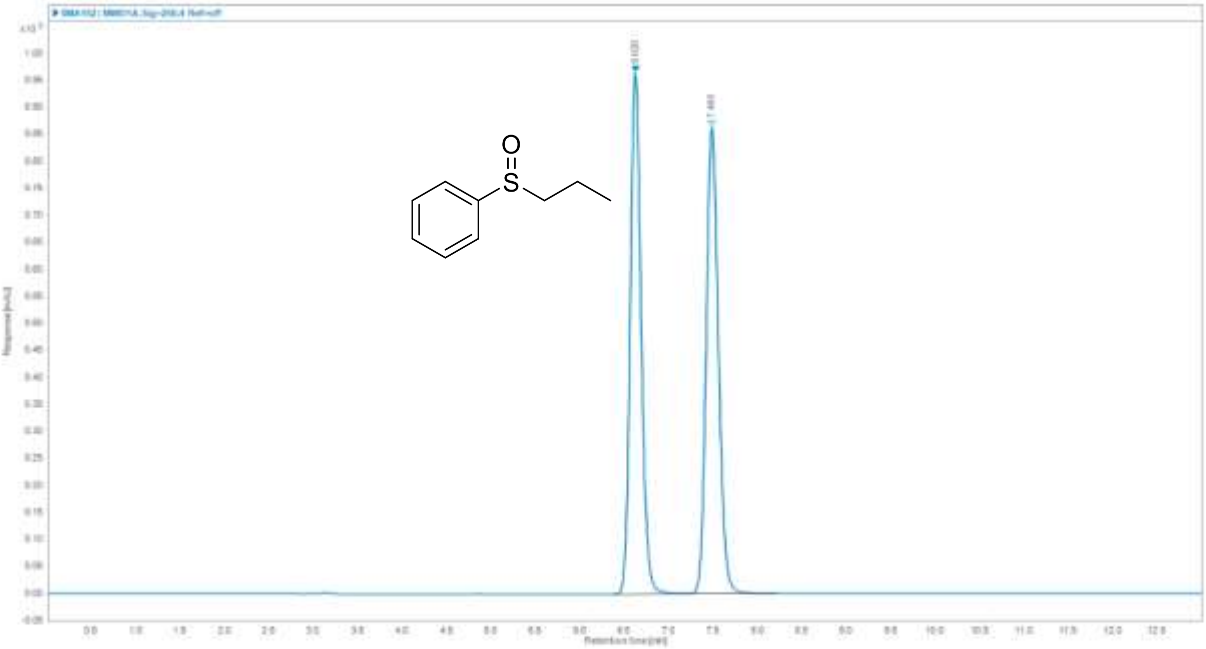

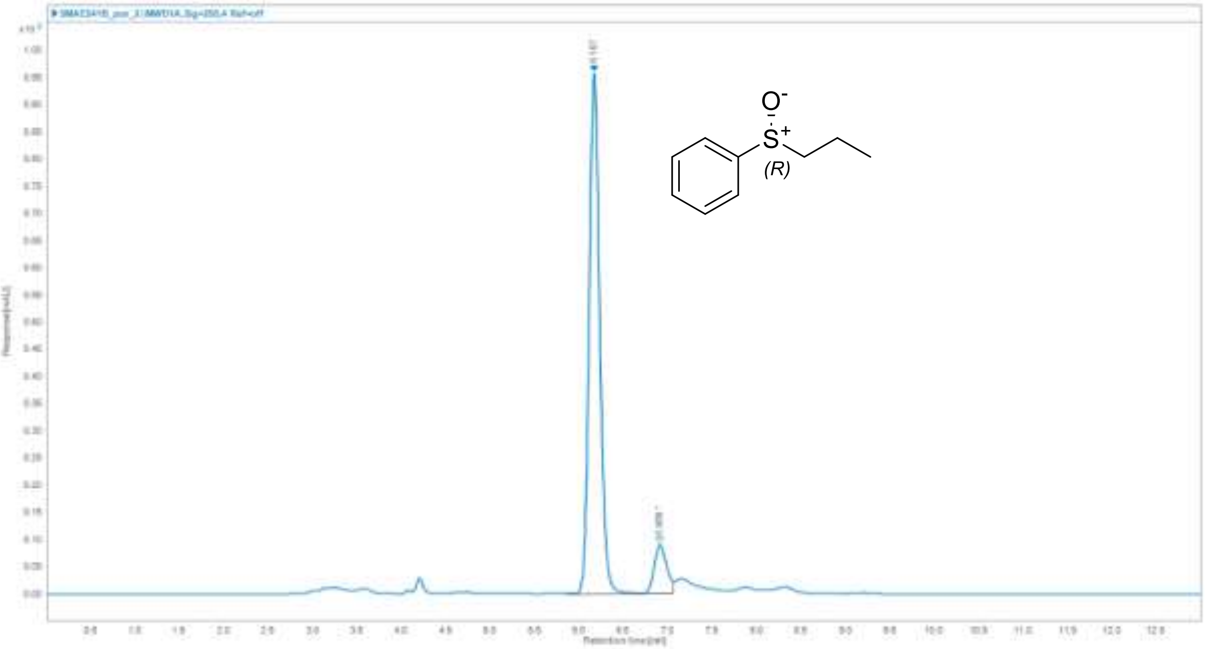

6.4.26 1-Bromo-4-(propylsulfinyl)benzene (1z)

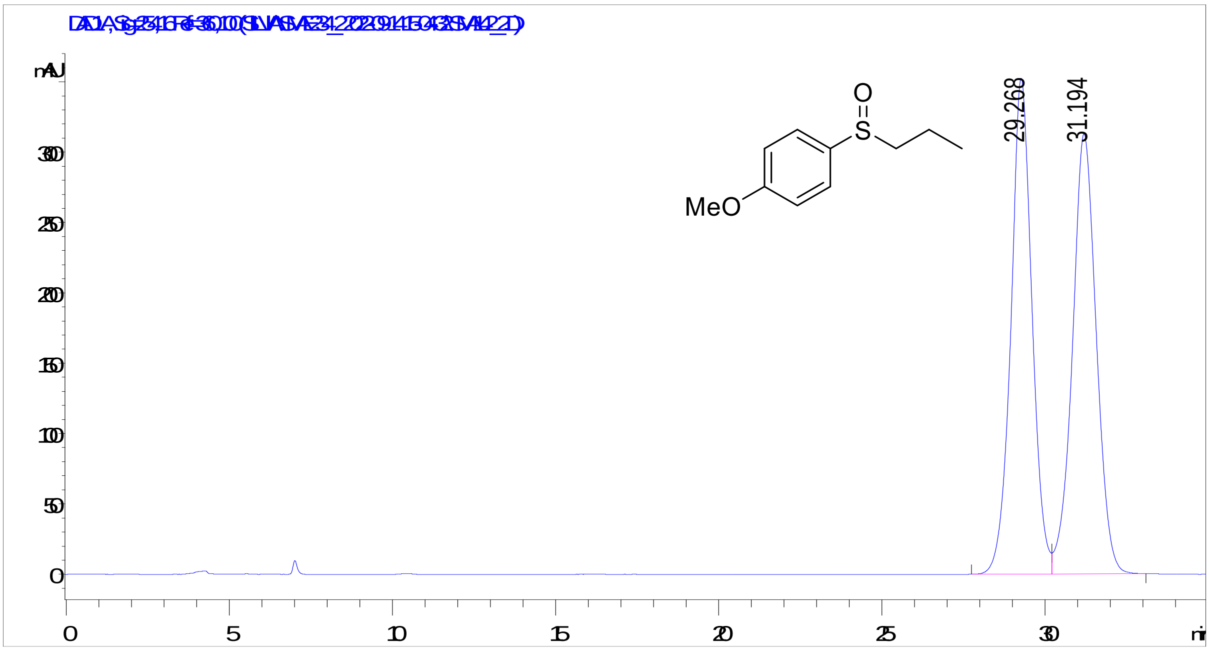

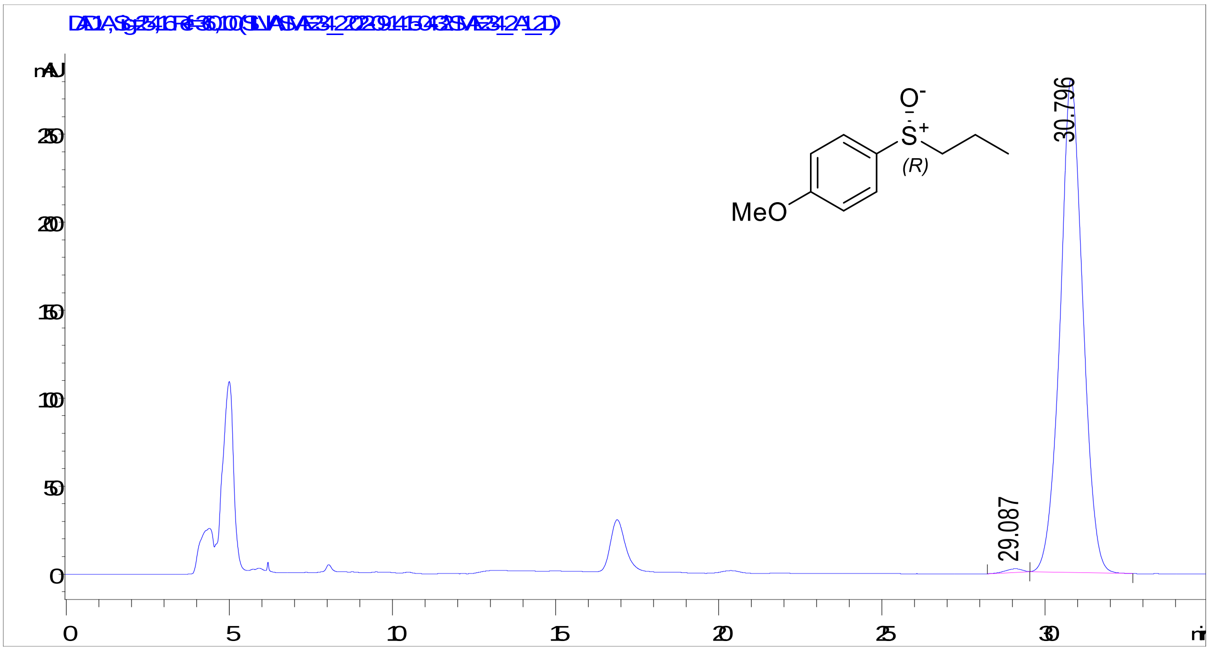

HPLC trace obtained from crude extract of the enzymatic reaction.

6.4.27 (Vinylsulfinyl)benzene (1aa)

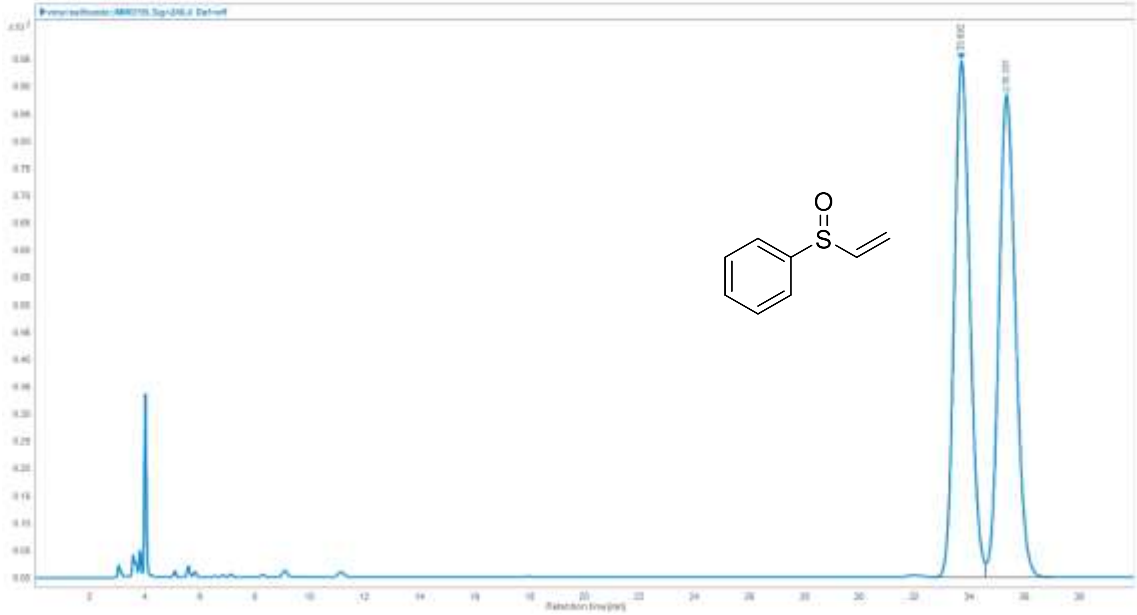

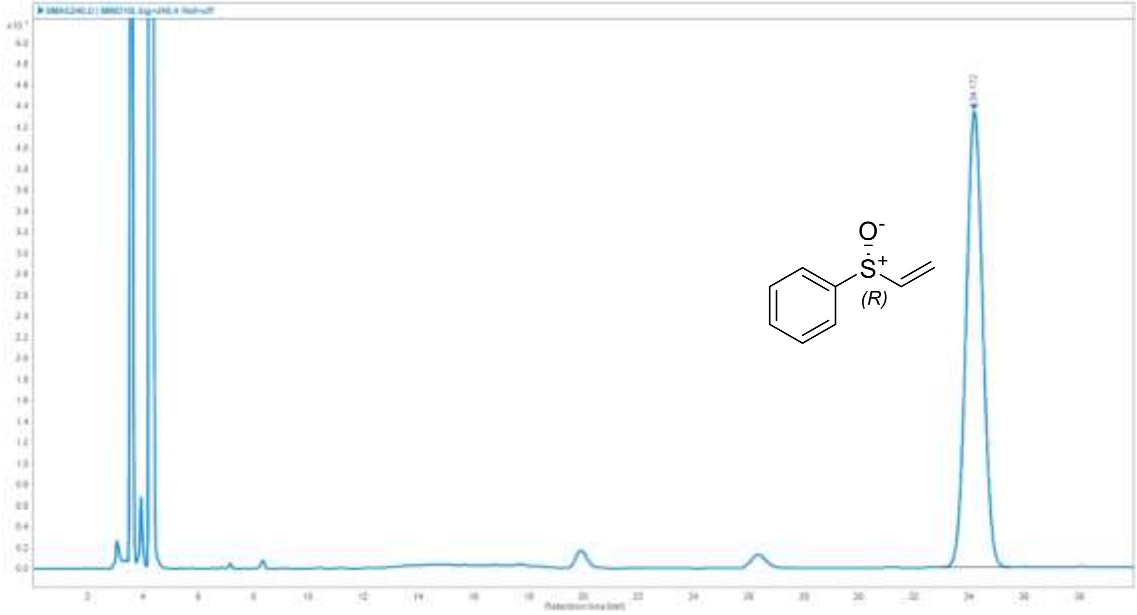

HPLC trace obtained from crude extract of the enzymatic reaction.

6.4.28 (Allylsulfinyl)benzene (1ab)

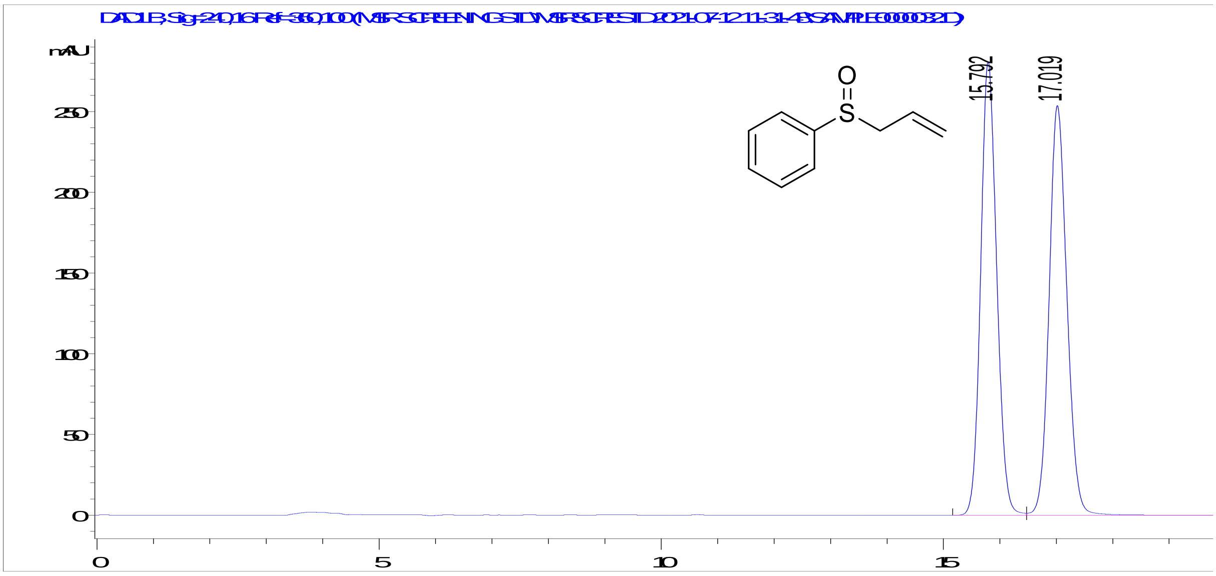

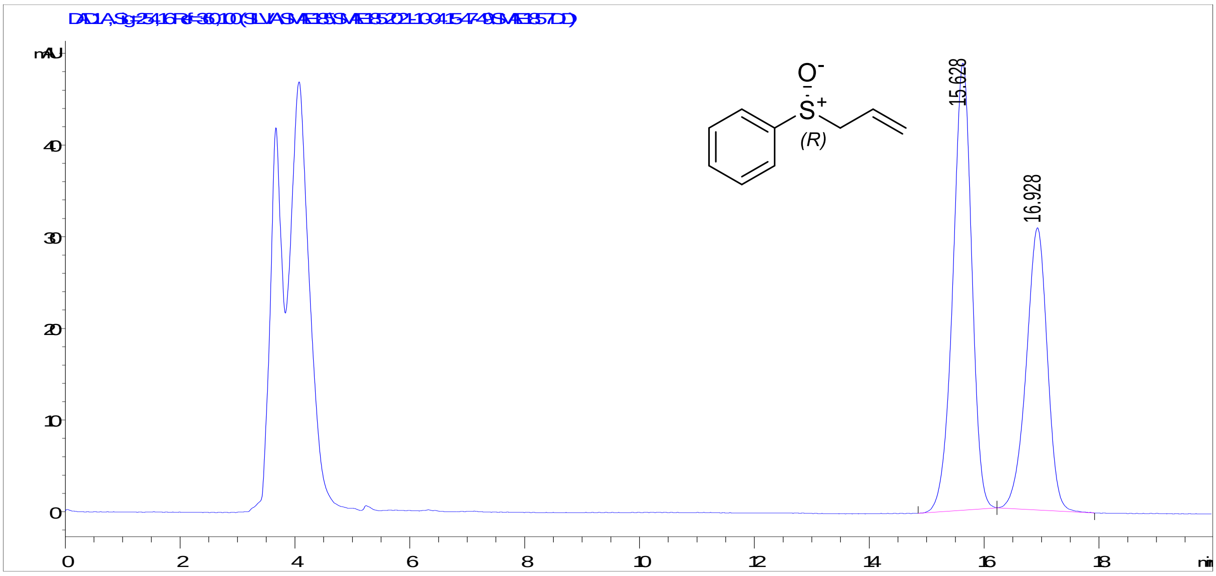

HPLC trace obtained from crude extract of the enzymatic reaction.

6.4.29 1-Methyl-4-(propylsulfinyl)benzene (1ac)

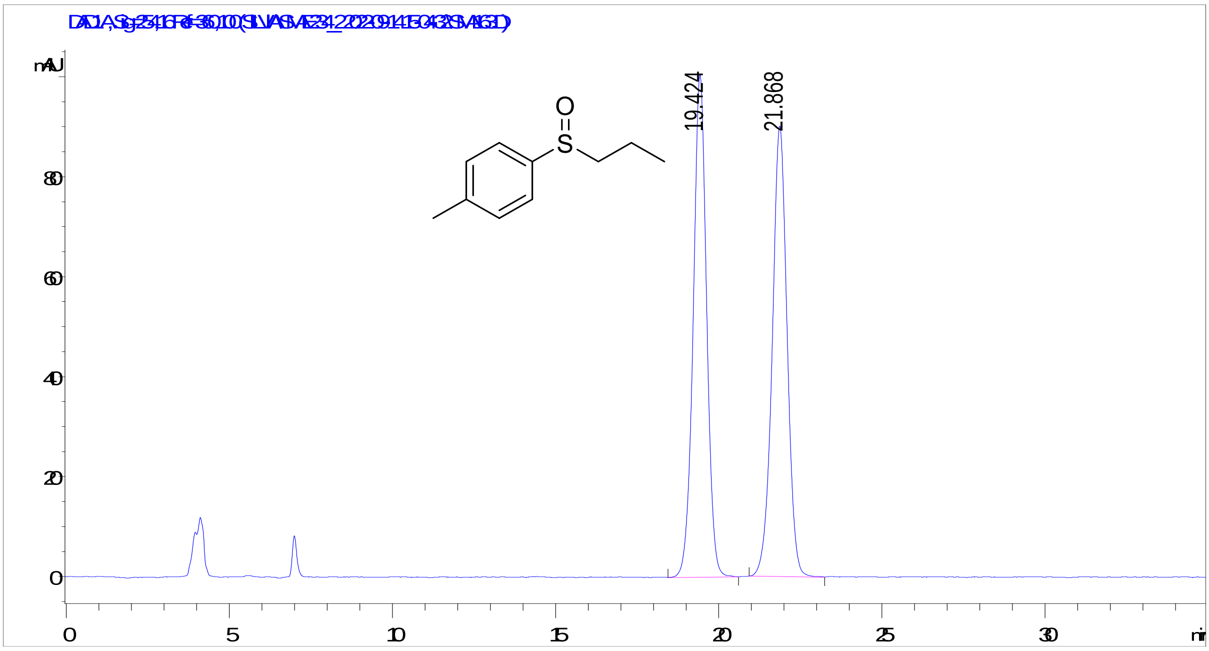

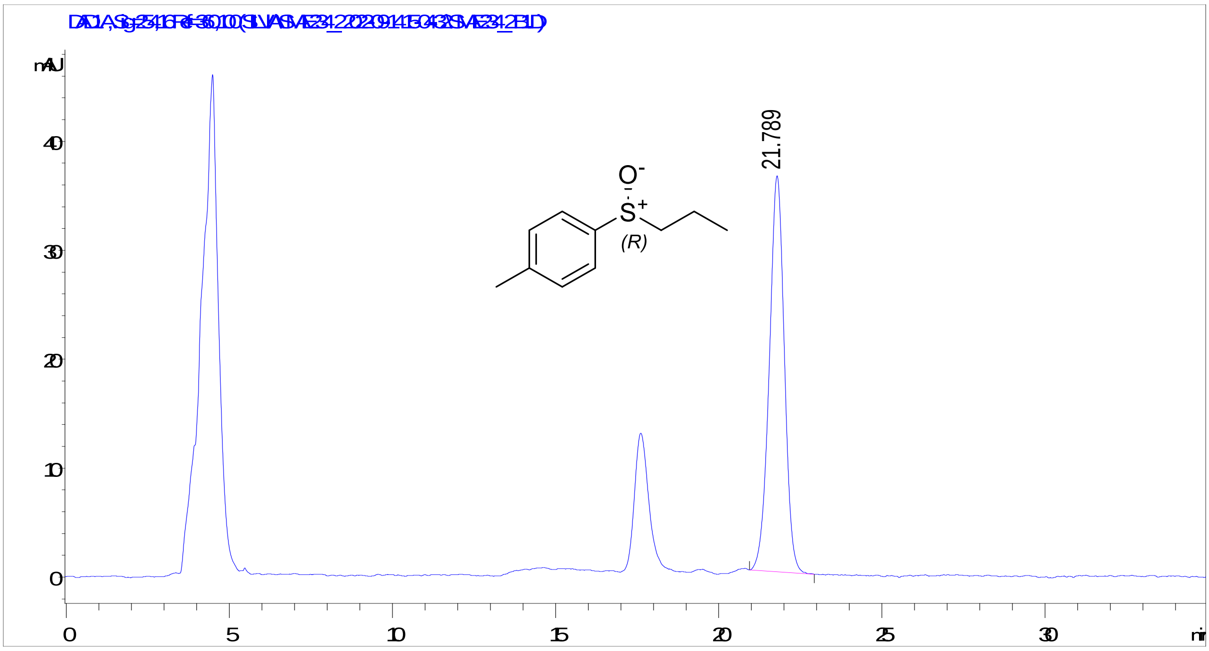

HPLC trace obtained from crude extract of the enzymatic reaction.

6.4.30 1-Chloro-4-(propylsulfinyl)benzene (1ad)

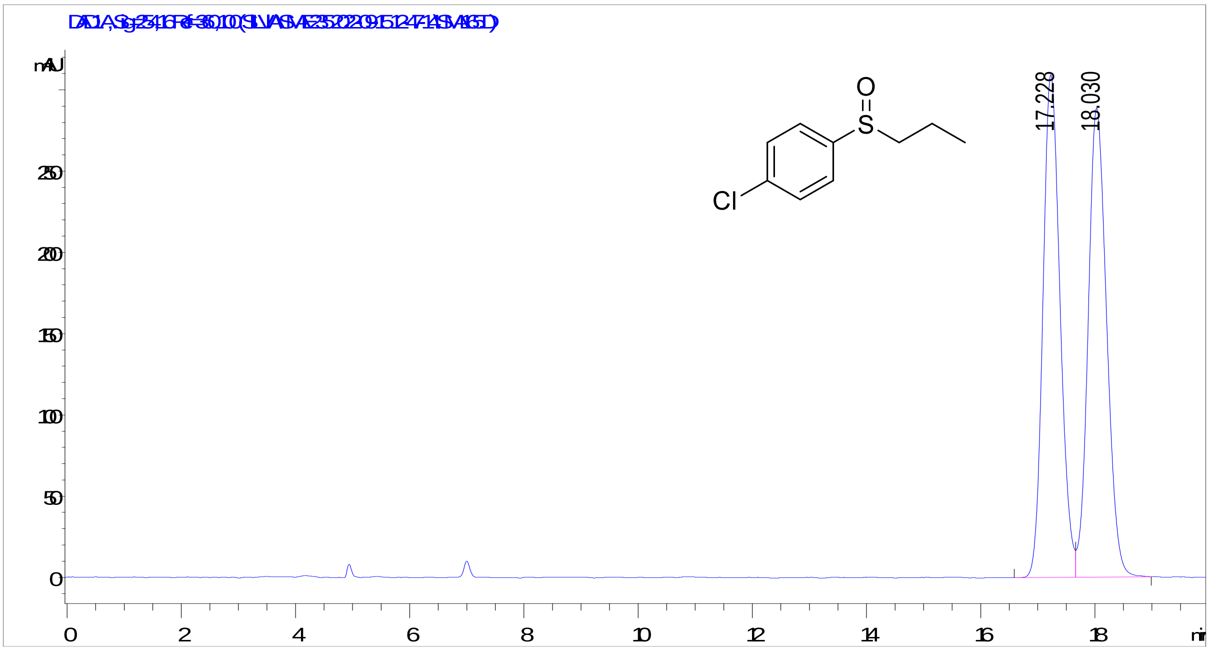

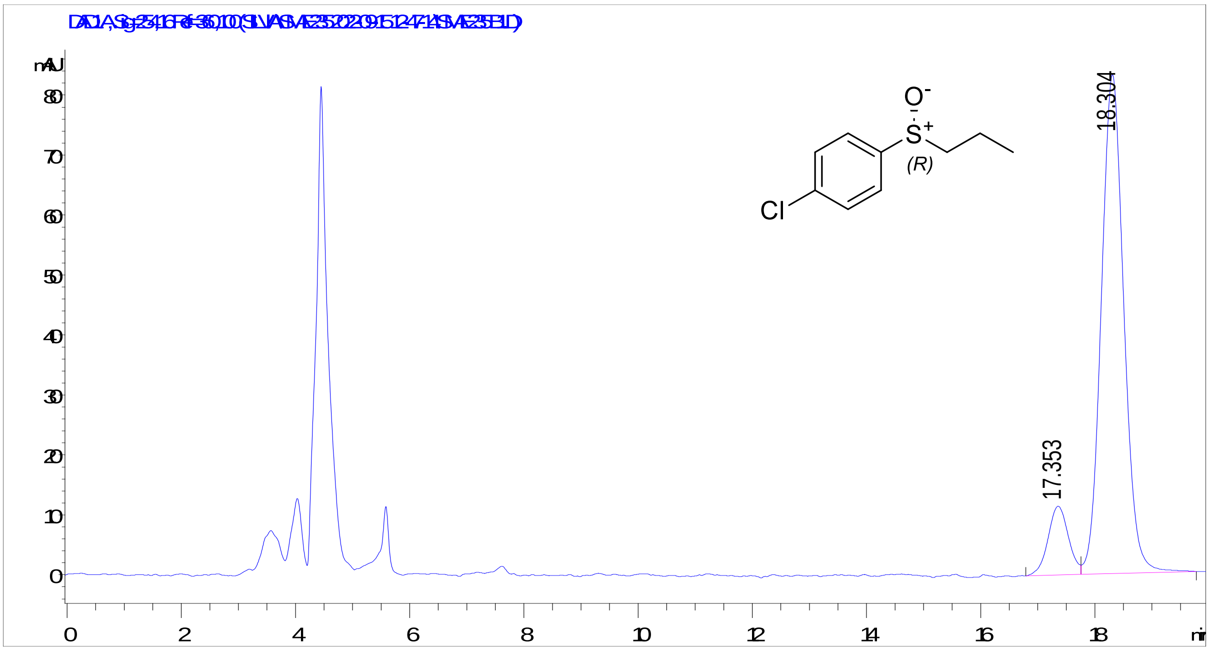

HPLC trace obtained from crude extract of the enzymatic reaction.

6.4.31 1-Bromo-4-(propylsulfinyl)benzene (1ae)

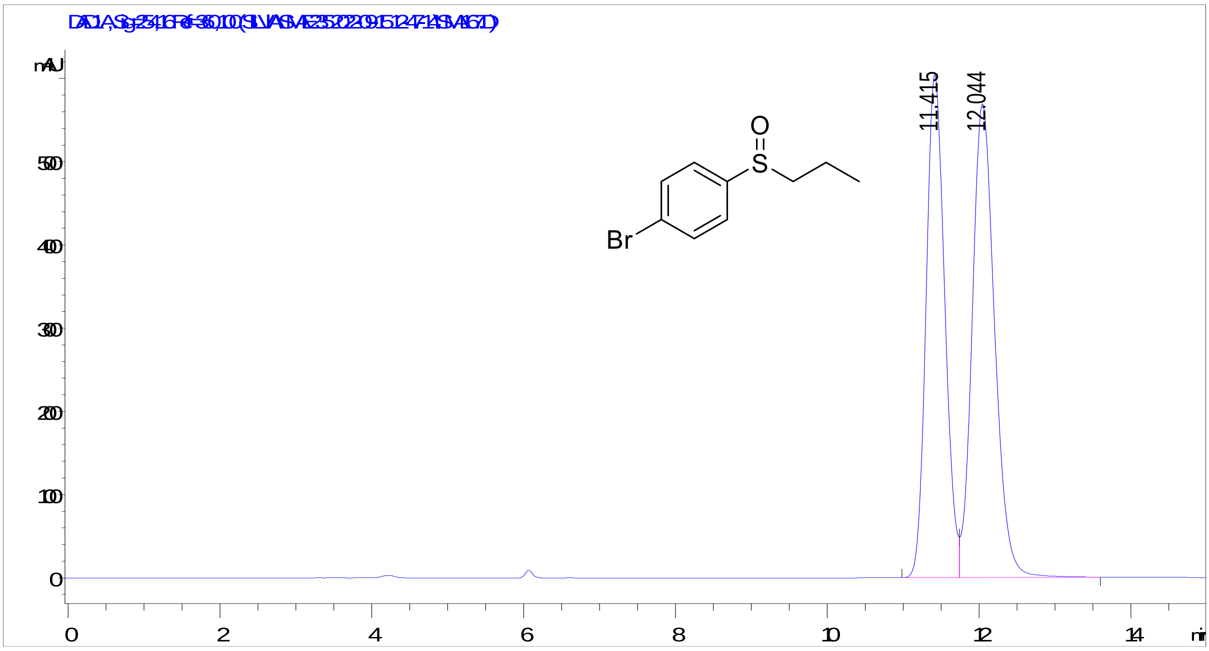

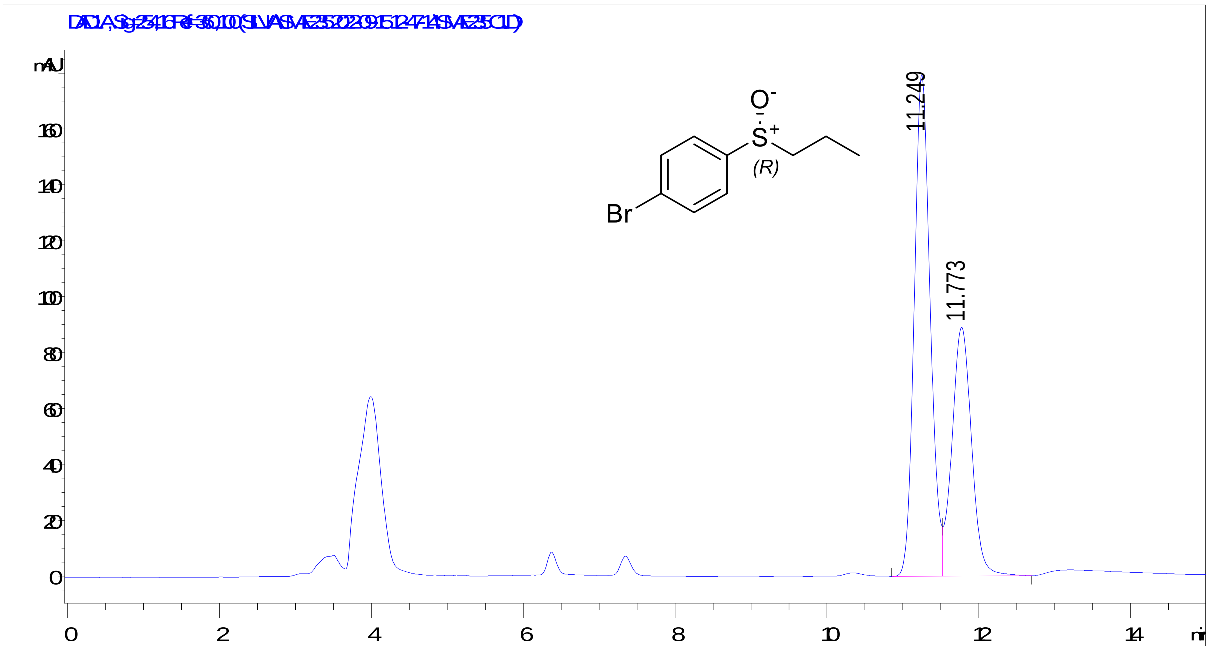

HPLC trace obtained from crude extract of the enzymatic reaction.

6.4.32 1-Methoxy-3-(propylsulfinyl)benzene (1af)

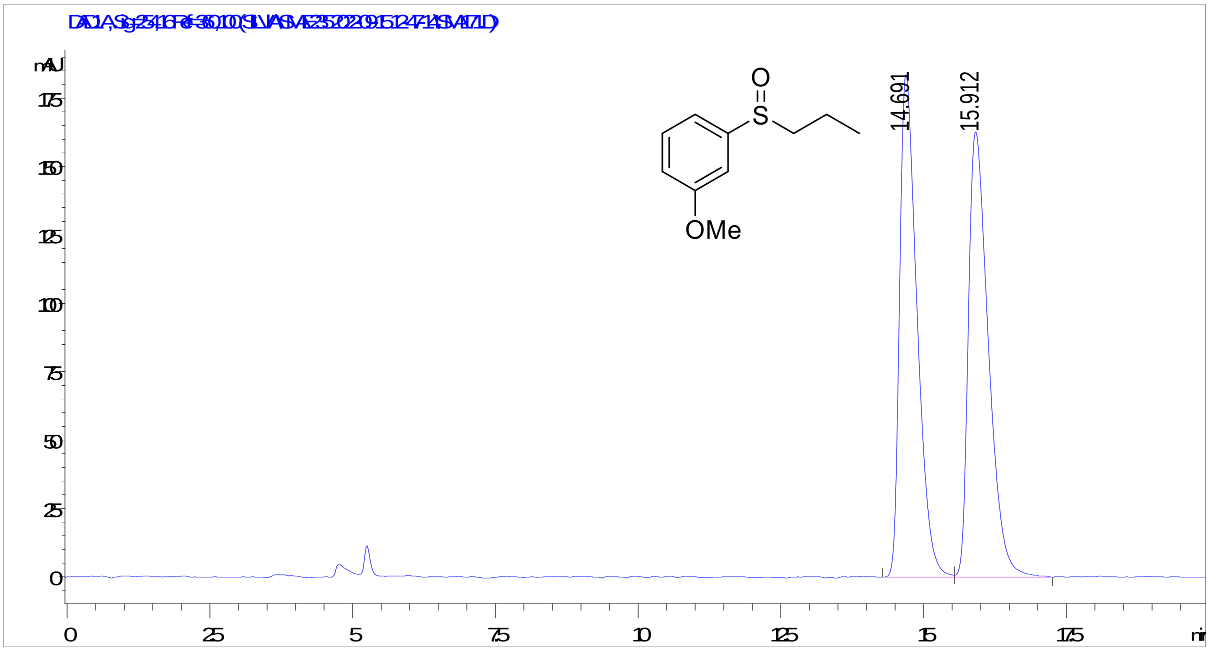

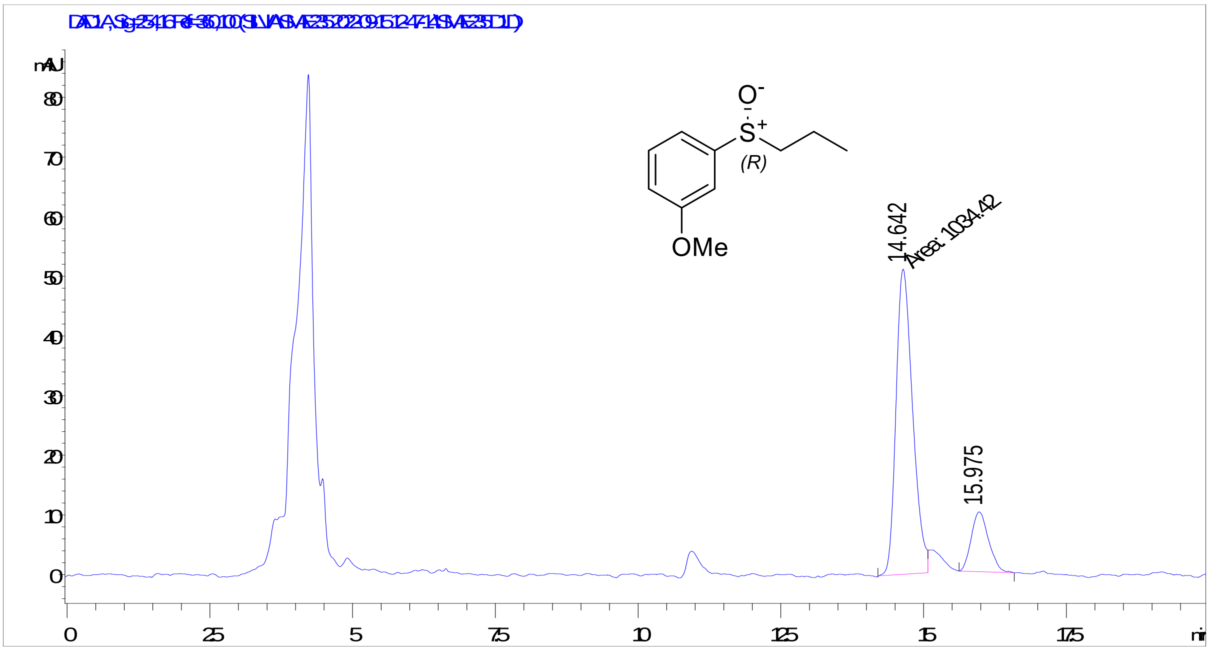

HPLC trace obtained from crude extract of the enzymatic reaction.

6.4.33 1-(Butylsulfinyl)-4-methoxybenzene (1ag)

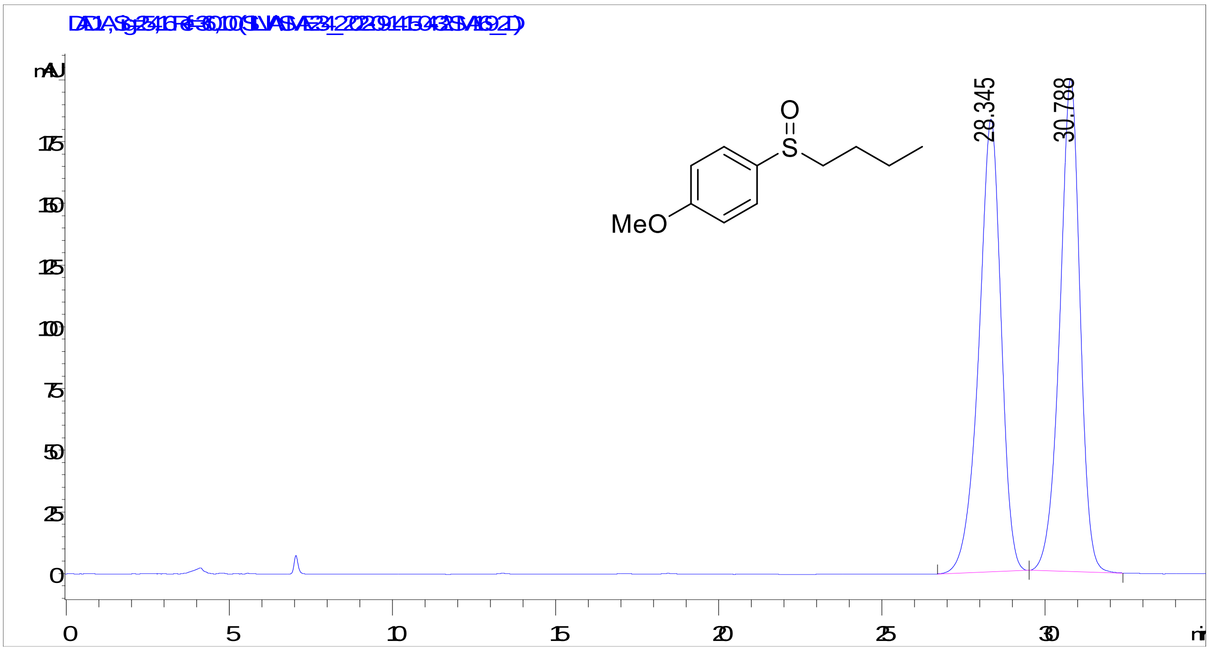

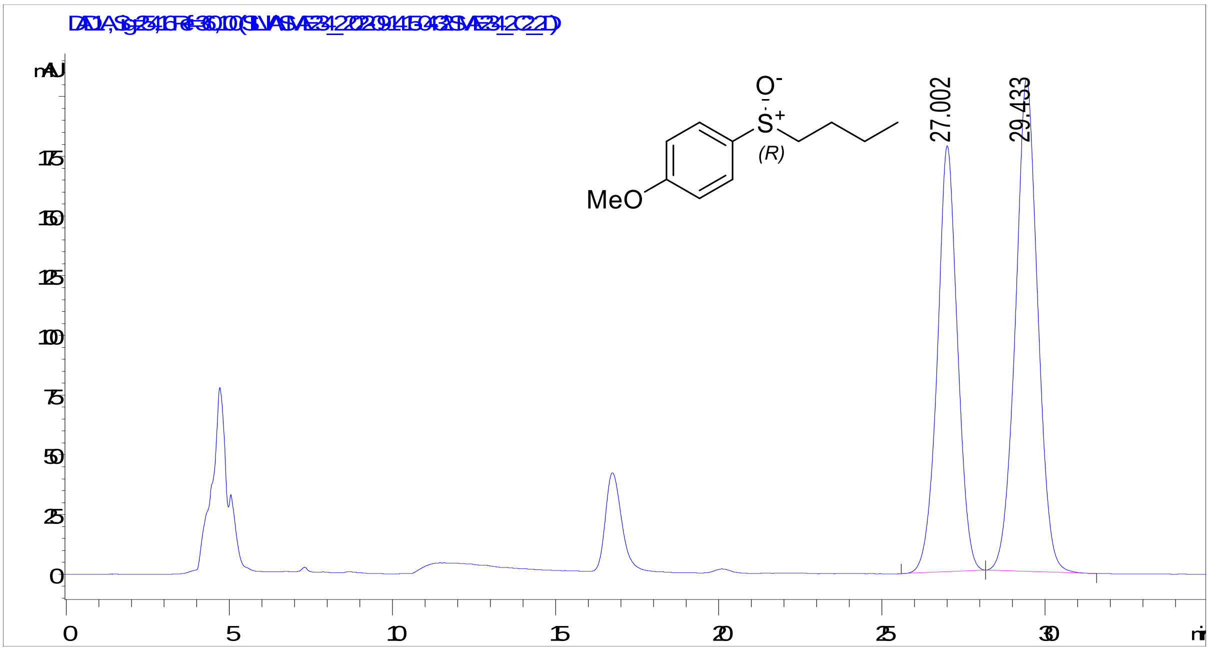

HPLC trace obtained from crude extract of the enzymatic reaction.

7 References

(1) Ma, Y. J.; Doni, A.; Skjoedt, M. O.; Honoré, C.; Arendrup, M.; Mantovani, A.; Garred, P. Heterocomplexes of Mannose-Binding Lectin and the Pentraxins PTX3 or Serum Amyloid P Component Trigger Cross-Activation of the Complement System. *J. Biol. Chem.* **2011**, *286* (5), 3405–3417.

(2) Wang, R. Y. R.; Song, Y.; Barad, B. A.; Cheng, Y.; Fraser, J. S.; DiMaio, F. Automated Structure Refinement of Macromolecular Assemblies from Cryo-EM Maps Using Rosetta. *Elife* **2016**, *5*, 1–22.

(3) Abraham, M. J.; Murtola, T.; Schulz, R.; Páll, S.; Smith, J. C.; Hess, B.; Lindah, E. GROMACS: High Performance Molecular Simulations through Multi-Level Parallelism from Laptops to Supercomputers. *SoftwareX* **2015**, *1–2*, 19–25. https://doi.org/10.1016/J.SOFTX.2015.06.001.

(4) Ashvar, C. S.; Devlin, F. J.; Bak, K. L.; Taylor, P. R.; Stephens, P. J. Ab Initio Calculation of Vibrational Absorption and Circular Dichroism Spectra: 6,8-Dioxabicyclo[3.2.1]Octane. *J. Phys. Chem.* **1996**, *100* (22), 9262–9270.

(5) Tomasi, J.; Mennucci, B.; Cammi, R. Quantum Mechanical Continuum Solvation Models. *Chem. Rev.* **2005**, *105* (8), 2999–3093.

(6) Morris, G. M.; Goodsell, D. S.; Halliday, R. S.; Huey, R.; Hart, W. E.; Belew, R. K.; Olson, A. J. Automated Docking Using a Lamarckian Genetic Algorithm and an Empirical Binding Free Energy Function. *J. Comput. Chem.* **1998**, *19*, 1639–1662.

(7) Hornak, V.; Abel, R.; Okur, A.; Strockbine, B.; Roitberg, A.; Simmerling, C. Comparison of Multiple Amber Force Fields and Development of Improved Protein Backbone Parameters. *Proteins: Struct., Funct., Bioinf.* **2006**, *65* (3), 712–725.

(8) Hess, B.; Bekker, H.; Berendsen, H. J. C.; Fraaije, J. G. E. M. LINCS: A Linear Constraint Solver for Molecular Simulations. *J. Comput. Chem.* **1997**, *18*, 14631472.

(9) Darden, T.; York, D.; Pedersen, L. Particle Mesh Ewald: An N·log(N) Method for Ewald Sums in Large Systems. *J. Chem. Phys.* **1998**, *98* (12), 10089.

(10) Delaglio, F.; Grzesiek, S.; Vuister, G. W.; Zhu, G.; Pfeifer, J.; Bax, A. NMRPipe: A Multidimensional Spectral Processing System Based on UNIX Pipes*. *J. Biomol. NMR* **1995**, *6*, 293.

(11) Johnson, B. A. Using NMRView to Visualize and Analyze the NMR Spectra of Macromolecules. In *Protein NMR Techniques*; Downing, K. A., Ed.; Humana Press, 2004; Vol. 278, pp 313–352.

(12) Blakemore, P. R.; Burge, M. S. Iterative Stereospecific Reagent-Controlled Homologation of Pinacol Boronates by Enantioenriched α-Chloroalkyllithium Reagents. *J. Am. Chem. Soc.* **2007**, *129* (11), 3068–3069.

(13) Anselmi, S.; Liu, S.; Kim, S.-H.; Barry, S. M.; Moody, T. S.; Castagnolo, D. A Mild and Chemoselective CALB Biocatalysed Synthesis of Sulfoxides Exploiting the Dual Role of AcOEt as Solvent and Reagent. *Org Biomol Chem* **2021**, *19* (1), 156–161.

(14) Nosek, V.; Míšek, J. Chemoenzymatic Deracemization of Chiral Sulfoxides. *Angew. Chem.* **2018**, *130* (31), 9849–9852.

(15) Rioz-Martínez, A.; Kopacz, M.; de Gonzalo, G.; Torres Pazmiño, D. E.; Gotor, V.; Fraaije, M. W. Exploring the Biocatalytic Scope of a Bacterial Flavin-Containing Monooxygenase. *Org. Biomol. Chem.* **2011**, *9* (5), 1337–1341.

(16) Amri, N.; Wirth, T. Flow Electrosynthesis of Sulfoxides, Sulfones, and Sulfoximines without Supporting Electrolytes. *J. Org. Chem.* **2021**, *86*, 15961–15972.

(17) Yang, J.; Wen, Y.; Peng, L.; Chan, Y.; Cheng, X.; Chen, Y. Identification of MsrA Homologues for the Preparation of (R)-Sulfoxides at High Substrate Concentrations. *Org. Biomol. Chem.* **2019**, *17* (13), 3381–3388.

(18) Davidson, S. C.; Gomes, G. dos P.; Kuhn, L. R.; Alabugin, I. v.; Kennedy, A. R.; Tomkinson, N. C. O. Organocatalytic Sulfoxidation. *Tetrahedron* **2021**, *78*, 1–10.

(19) Kim, S. S.; Nehru, K.; Kim, S. S.; Kim, D. W.; Jung, H. C. A Mild and Highly Efficient Oxidation of Sulfides to Sulfoxides with Periodic Acid Catalyzed by $FeCl_3$. *Synthesis (Stuttg)* **2002**, No. 17, 2484–2486.

(20) Park, J. K.; Lee, S. Sulfoxide and Sulfone Synthesis via Electrochemical Oxidation of Sulfides. *J. Org. Chem* **2021**, *86*, 13790–13799.

(21) Ruppenthal, S.; Brückner, R. Symmetric Diarylsulfoxides as Asymmetric Sulfinylating Reagents for Dialkylmagnesium Compounds. *J. Org. Chem.* **2015**, *80* (2), 897–910.

(22) Sato, T.; Nogi, K.; Yorimitsu, H. Palladium-Catalyzed Peri-Selective C−H Fluoroalkoxylation of Aryl Sulfoxides. *ChemCatChem* **2020**, *12* (13), 3467–3471.

(23) Gan, S.; Yin, J.; Yao, Y.; Liu, Y.; Chang, D.; Zhu, D.; Shi, L. Metal-and Additive-Free Oxygen-Atom Transfer Reaction: An Efficient and Chemoselective Oxidation of Sulfides to Sulfoxides with Cyclic Diacyl Peroxides. *Org. Biomol. Chem.* **2017**, *15*, 2647.

(24) Yadav, M.; Bhunia, A.; Jana, S. K.; Roesky, P. W. Manganese-and Lanthanide-Based 1D Chiral Coordination Polymers as an Enantioselective Catalyst for Sulfoxidation. *Inorg. Chem.* **2016**, *55*, 28.

(25) Petsi, M.; Zografos, A. L. 2,5-Diketopiperazine Catalysts as Activators of Dioxygen in Oxidative Processes. *ACS Catal.* **2020**, *10* (13), 7093–7099.

(26) Motoshima, K.; Sato, A.; Yorimitsu, H.; Oshima, K. Efficient Aerobic Oxidation of Phosphines, Phosphites, and Sulfides by Using Trialkylborane. *Bull. Chem. Soc. Jpn.* **2007**, *80*, 2229–2231.

(27) Liu, Q.; Wang, L.; Yue, H.; Li, J. S.; Luo, Z.; Wei, W. Catalyst-Free Visible-Light-Initiated Oxidative Coupling of Aryldiazo Sulfones with Thiols Leading to Unsymmetrical Sulfoxides in Air. *Green Chem.* **2019**, *21* (7), 1609–1613.

(28) Gogoi, P.; Kotipalli, T.; Indukuri, K.; Bondalapati, S.; Saha, P.; Saikia, A. K. Application of a Novel 1,3-Diol with a Benzyl Backbone as Chiral Ligand for Asymmetric Oxidation of Sulfides to Sulfoxides. *Tetrahedron Lett.* **2012**, *53* (22), 2726–2729.

(29) Jalba, A.; Régnier, N.; Ollevier, T. Enantioselective Aromatic Sulfide Oxidation and Tandem Kinetic Resolution Using Aqueous H_2O_2 and Chiral Iron–Bis(Oxazolinyl)Bipyridine Catalysts. *Eur. J. Org. Chem.* **2017**, *2017* (12), 1628–1637.

(30) Tang, J.; Huang, F.; Wei, Y.; Bian, H.; Zhang, W.; Liang, H. Bovine Serum Albumin–Cobalt(II) Schiff Base Complex Hybrid: An Efficient Artificial Metalloenzyme for Enantioselective Sulfoxidation Using Hydrogen Peroxide. *Dalton Trans.* **2016**, *45* (19), 8061–8072.

(31) Gelat, F.; Jayashankaran, J.; Lohier, J. F.; Gaumont, A. C.; Perrio, S. Organocatalytic Asymmetric Synthesis of Sulfoxides from Sulfenic Acid Anions Mediated by a Cinchona - Derived Phase-Transfer Reagent. *Org. Lett.* **2011**, *13* (12), 3170–3173.

(32) Sakuraba, H.; Maekawa, H. Enantioselective Oxidation of Sulfides Catalyzed by Chiral MoV and CuII Complexes of Catechol-Appended β-Cyclodextrin Derivatives in Water. *J. Inclusion Phenom. Macrocyclic Chem.* **2006**, *54* (1), 41–45.

(33) Gelat, F.; Gaumont, A. C.; Perrio, S. Chiral Non-Racemic Sulfoxides by Asymmetric Alkylation of Alkanesulfenates in the Presence of a Chiral Ammonium Phase-Transfer Catalyst Derived from Cinchona Alkaloid. *J. Sulfur Chem.* **2013**, *34* (6), 596–605.
